# Cortical structure and the risk for Alzheimer’s disease: a bidirectional Mendelian randomization study

**DOI:** 10.1038/s41398-021-01599-x

**Published:** 2021-09-15

**Authors:** Bang-Sheng Wu, Ya-Ru Zhang, Hong-Qi Li, Kevin Kuo, Shi-Dong Chen, Qiang Dong, Yong Liu, Jin-Tai Yu

**Affiliations:** 1 Department of Neurology and Institute of Neurology, Huashan Hospital, State Key Laboratory of Medical Neurobiology and MOE Frontiers Center for Brain Science, Shanghai Medical College, Fudan University, Shanghai, China; 2 School of Artificial Intelligence, Beijing University of Posts and Telecommunications, Beijing, China

**Keywords:** Medical genetics, Neuroscience

## Abstract

Progressive loss of neurons in a specific brain area is one of the manifestations of Alzheimer’s disease (AD). Much effort has been devoted to investigating brain atrophy and AD. However, the causal relationship between cortical structure and AD is not clear. We conducted a bidirectional two-sample Mendelian randomization analysis to investigate the causal relationship between cortical structure (surface area and thickness of the whole cortex and 34 cortical regions) and AD risk. Genetic variants used as instruments came from a large genome-wide association meta-analysis of cortical structure (33,992 participants of European ancestry) and AD (AD and AD-by-proxy, 71,880 cases, 383,378 controls). We found suggestive associations of the decreased surface area of the temporal pole (OR (95% CI): 0.95 (0.9, 0.997), *p* = 0.04), and decreased thickness of cuneus (OR (95% CI): 0.93 (0.89, 0.98), *p* = 0.006) with higher AD risk. We also found a suggestive association of vulnerability to AD with the decreased surface area of precentral (*β* (SE): –43.4 (21.3), *p* = 0.042) and isthmus cingulate (*β* (SE): –18.5 (7.3), *p* = 0.011). However, none of the Bonferroni-corrected *p* values of the causal relationship between cortical structure and AD met the threshold. We show suggestive evidence of an association of the atrophy of the temporal pole and cuneus with higher AD risk. In the other direction, there was a suggestive causal relationship between vulnerability to AD and the decreased surface area of the precentral and isthmus cingulate. Our findings shed light on the associations of cortical structure with the occurrence of AD.

## Introduction

Alzheimer’s disease (AD) is the main cause of dementia, which is characterized by the aggregation of amyloid-β (Aβ) peptides and neurofibrillary tangles [1]. In AD, excessive neuronal loss was observed in some brain regions, for example, the hippocampus [2]. And neurodegenerative disease shows selective neuronal loss mainly in the subcortical areas and cerebral cortex, resulting in abnormality in the cortical surface area and cortical thickness [3]. At present, the cortical surface area and cortical thickness of brain regions have been repeatedly reported to be associated with AD, including some typical and atypical atrophic regions. For instance, the atrophy of the hippocampus [4], entorhinal [5], medial temporal [6], the precuneus [7], and orbitofrontal [8] has been found in AD. Besides, a previous study also used different patterns of brain atrophy to distinguish subtypes of AD (typical, limbic-predominant, and hippocampal-sparing) [9]. Considering selectively vulnerable neurons in AD pathology [10], and the heterogeneity in the pattern of atrophy in AD [11], it is difficult to find out the relationship between AD and cortex.

As AD neuropathologic change emerges before the clinical symptoms start [12], cortical structural changes may appear before the occurrence of AD. Nevertheless, neurodegenerative diseases mainly affect the elderly whose brain structures differ from those of the young. As a confounding factor, aging made it difficult to elucidate the true causal relationship between neurodegenerative disease and the change in brain structure [13]. Moreover, it is not clear whether the changes in the surface area and thickness of the brain area are the cause of AD or the results of the disease. Although there have been some observational studies to explore the associations of cortical surface area and cortical thickness with AD, a correlation between a risk factor and an outcome cannot be reliably interpreted for a variety of confounding factors or reverse causation [14]. Therefore, a tailored approach is warranted to figure out the causal relationship between cortical structure and the occurrence of AD.

Mendelian randomization, using the single-nucleotide polymorphisms (SNPs) of genome-wide association study (GWAS) as an instrumental variable (IV) to deal with causal inference, widely used [15] for this model can provide accurate causal inference when the three assumptions are not violated. Mendelian randomization can be regarded as a natural randomized controlled trial for mutations that are randomly assigned to gametes when the cell undergoes meiosis [16–19]. Thus, reverse causation could be avoided since the occurrence of disease cannot affect the genotype.

In this study, we investigated the causal relationship between cortical structure and AD using bidirectional Mendelian randomization to further understand the etiology and progression of AD, as well as to better elucidate the potential interaction between cortical structure and AD risk.

## Materials and methods

**Fig. 1:**
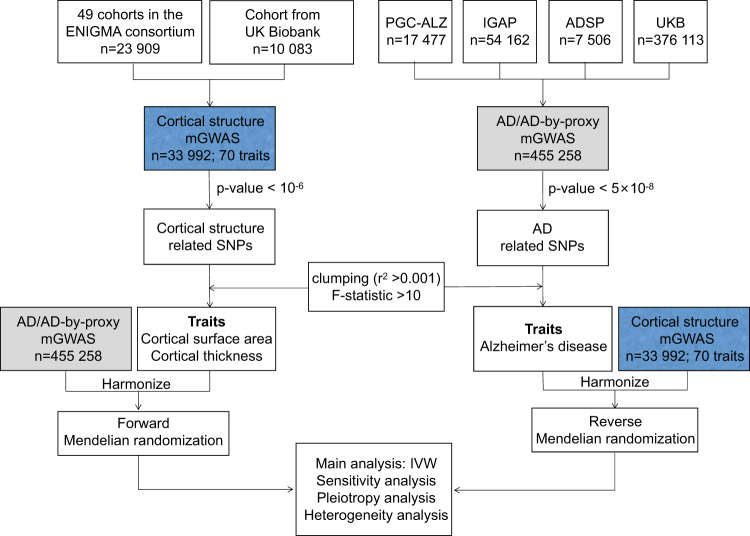
Flowchart of our bidirectional two-sample Mendelian randomization analysis. AD Alzheimer’s disease, AD-by-proxy based on parental diagnoses, ADSP the Alzheimer’s Disease Sequencing Project, ENIGMA the Enhancing NeuroImaging Genetics through Meta-Analysis, IGAP the International Genomics of Alzheimer’s Project, IVW inverse-variance weighted, mGWAS meta-analysis of GWAS, PGC-ALZ Alzheimer’s Disease Working Group of the Psychiatric Genomics Consortium, SNP single-nucleotide polymorphism, UKB the UK Biobank.

We conduct a bidirectional two-sample Mendelian randomization to investigate the causal associations of two cortical structure phenotypes (cortical surface area and cortical thickness) with AD (Fig. 1).

### Cortical structure phenotype data

The principal GWAS of the human cerebral cortex was based on 33,992 participants of European ancestry, including 23,909 from 49 cohorts participating in the Enhancing NeuroImaging Genetics through Meta-Analysis (ENIGMA) Consortium and 10,083 from the UK Biobank [20] (see Supplementary Table 1, Additional File 1). As for our study, the measured cortical surface area and cortical thickness of the whole cortex and 34 brain regions were defined by the Desikan–Killiany cortical atlas.

### Genetic data on AD

Genetic variants associated with AD were obtained from the meta-analysis of GWAS of participants of European ancestry who were clinically diagnosed with AD and AD-by-proxy (71,880 cases, 383,378 controls) (AD-by-proxy: based on parental diagnoses; the genetic correlation between AD and AD-by-proxy: *r*_*g*_ = 0.81) [21]. Four consortia (the Alzheimer’s Disease Sequencing Project (ADSP); the International Genomics of Alzheimer’s Project (IGAP); Alzheimer’s Disease Working Group of the Psychiatric Genomics Consortium (PGC-ALZ); the UK Biobank (UKB)) were included in the meta-analysis. The summary information can be found in Supplementary Table 1, Additional File 1.

### Mendelian randomization analysis

We used instrument variables from two different GWAS summary results to perform two-sample Mendelian randomization that could increase the estimated power.

We chose two sets of *p* values for genetic variants associated with the exposure in the bidirectional analysis. In the Mendelian randomization analysis for causal estimation of cortical structure on AD, the threshold of genome-wide significance was set at *p* < 10^–6^ for lack of significant SNPs available. And the threshold was set at *p* = 5 × 10^–8^ in the inverse analysis. Although the statistic power could increase if we increase the IVs in the Mendelian randomization analysis, those additional SNPs may violate the three core assumptions of Mendelian randomization and they could be weak instruments, thus biasing the causal estimate and decreasing the statistic power. To obtain independent SNPs associated with the exposure, we used linkage disequilibrium clumping (*r*^2^ > 0.001) and the SNPs with the most significant *p* values were retained. Then we harmonized the exposure and outcome data according to the same effect alleles and palindromic SNPs were removed. To identify the weak instruments, we calculated the variance explained by the instruments and the *F*-statistic.

Inverse-variance weighted (IVW) method [22] was implemented as a primary method in the following analysis. In the IVW method, we combined the ratios of SNP-exposure to SNP-outcome in a fixed-effects meta-analysis or random-effects meta-analysis to estimate the causal relationship between exposure and outcome. The estimates from the fixed-effects meta-analysis and random-effects meta-analysis were the same and if there was heterogeneity between SNPs, we would choose the random-effects model. The IVW method assumes that all the IVs are valid and could give a precise estimate if the core assumption of Mendelian randomization is not violated. However, if the genetic variations influence the outcome through a pathway other than through the exposure (horizontal pleiotropy), the estimate can be biased. Thus, using the MR-Egger [23] and weighted median [24] methods, we performed the sensitivity analysis, from which we inferred a causal relationship between exposure and outcome despite the existence of invalid SNPs. Unlike the IVW method, MR-Egger regression did not constrain the slope to pass through zero in the exposure–outcome estimate, where the intercept was used to identify the presence of directional pleiotropy. We also calculated *I*^2^_GX_ to find whether there was a violation of the NOME (no measurement error in the SNP-exposure effects) assumption [23, 25]. An *I*^2^_GX_ < 0.9 indicated that the causal estimate was inaccurate and should be interpreted with caution. The weighted median allowed half of the SNPs to be valid instruments for the causal estimate. Although its power might decrease, the weighted median would provide robust estimates when up to 50% of the invalid SNPs existed.

Besides, we detected the horizontal pleiotropy using MR-PRESSO global test and removed the outlying SNPs using the MR-PRESSO outlier test [26]. And we also investigated whether there was a statistically significant difference before and after removing the outlying SNPs. Finally, “leave-one-out” analysis and “single SNP” analysis were used to identify whether a single SNP was driving the main causal relationship and the Cochran *Q* test was used to detect the heterogeneity when we used IVW and MR-Egger methods to analyze the causal relationship [27].

To calculate OR per 1-SD change in the causal estimation of cortical structure on AD risk, we converted each beta-coefficient and corresponding SE reported in the original GWAS to SD units as reported by a previous study [28]. We also used a Bonferroni-corrected *p* value (that is 0.05/140 = 3.6 × 10^–4^) to take into account multiple testing. A *p* value larger than the Bonferroni-corrected *p* but lesser than 0.05 was considered suggestive of an association. Statistical power was calculated for the Mendelian randomization analysis using the online power calculator [29].

All statistical analyses were conducted using R version 3.6.3. The Mendelian randomization analysis was performed using the “TwoSampleMR” version 0.5.2.

## Results

We used bidirectional Mendelian randomization to explore the causal relationships of cortical surface area and cortical thickness of the whole cortex and 34 brain regions with AD. The summary information of SNPs used as genetic instruments is shown in Supplementary Tables 2–5 in Additional File 1.

### The causal effect of cortical surface area on AD

**Fig. 2:**
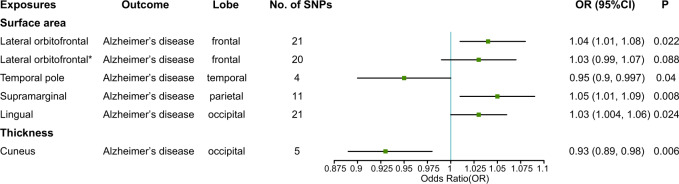
The causal effect of genetically predicted cortical structure on Alzheimer’s disease. SNP single-nucleotide polymorphism, OR odds ratio genetically predicted 1-SD unit increase in the cortical thickness, CI confidence interval, IVW inverse-variance weighted.

**Fig. 3:**
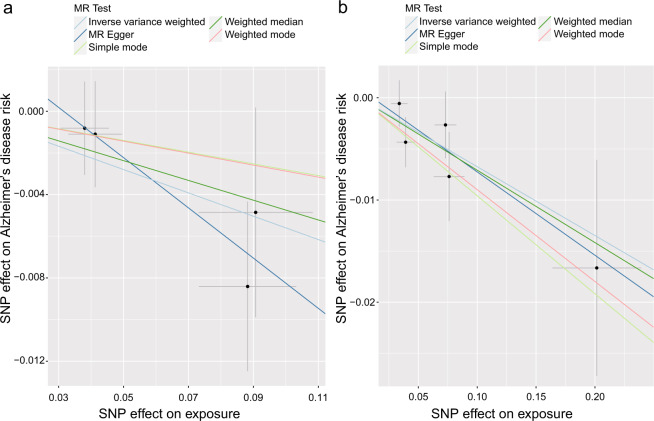
Scatterplot of single-nucleotide polymorphism (SNP) associated with the cortical structure and Alzheimer’s disease. **a** Scatterplot of SNP associated with the cortical surface area of the temporal pole versus AD. **b** Scatterplot of SNP associated with the cortical thickness of the cuneus versus AD. AD Alzheimer’s disease, SNP single-nucleotide polymorphism. Vertical and horizontal lines around each SNP show 95% confidence interval.

Concerning the causal effect of cortical surface area on AD, we conducted an estimation using the IVW method, and the suggestive causal association was shown in Fig. 2 and the full results of the causal estimates for all brain regions on AD are shown in Supplementary Fig. 1, Additional File 2. In the primary analyses, the cortical surface area (per 1 SD increase) of the temporal pole was suggestively associated with the risk of AD (OR (95% CI): 0.95 (0.90, 0.997), *p* = 0.04) (Figs. 2 and 3a). In the sensitivity analysis (Fig. 3a and see Supplementary Table 6, Additional File 1), although the 95% CIs of the temporal pole were wide, estimates in the MR-Egger and the weighted median were in the same direction. MR-PRESSO Global test and MR-Egger test indicated no notable horizontal or directional pleiotropy across SNPs in the causal estimates for the temporal pole on AD (*p* > 0.05) (see Supplementary Table 6, Additional File 1). Combining the leave-one-out analysis with the single SNP analysis, it was showed that rs6855246 had strong influences on the causal estimates for the temporal pole (see Supplementary Tables 7 and 8, Additional File 1 and Supplementary Figs. 2 and 3, Additional File 2). However, it has not been reported to be associated with AD, and its potential roles in biasing the causal estimate need further study. In addition, we also found suggestive associations of the cortical surface area (per 1 SD increase) of the lateral orbitofrontal, supramarginal, and lingual with the risk of AD (OR (95% CI): 1.04 (1.01, 1.08), *p* = 0.022; OR (95% CI): 1.05 (1.01, 1.09), *p* = 0.008; OR (95% CI): 1.03 (1.004, 1.06), *p* = 0.024) (Fig. 2 and see Supplementary Figs. 4–6, Additional File 2). However, the leave-one-out analysis found that SNP rs7252428 (in *CNN2* gene) and rs13208234 (in *RREB1* gene) had strong influences on the causal estimate for the lateral orbitofrontal (see Supplementary Tables 7 and 8, Additional File 1 and Supplementary Figs. 7 and 8, Additional File 2). Since the *CNN2* gene was previously reported to be associated with AD [30], we removed this SNP and found the suggestive causal relationship between the lateral orbitofrontal and AD disappeared after the removal (OR (95% CI): 1.03 (0.99, 1.07), *p* = 0.088). The results of the leave-one-out analysis and the single SNP analysis of the supramarginal and lingual can be found in Supplementary Tables 7 and 8, Additional File 1 and Supplementary Figs. 9–12, Additional File 2. Besides, no heterogeneity was found for the temporal pole, supramarginal, lateral orbitofrontal, and lingual, using the Cochran *Q* test (see Supplementary Table 9, Additional File 1).

### The causal effect of cortical thickness on AD

Using the information of genetic variants associated with cortical thickness, only one suggestive exposure–outcome pair was found (Figs. 2 and 3b) and the full results of the causal estimates for all brain regions on AD are shown in Supplementary Fig. 13, Additional File 2. Mendelian randomization analysis using IVW method showed that a 1-SD increase in the thickness of cuneus was suggestively associated with a decreased risk of AD, which was confirmed by a sensitivity analysis using the weighted median method (IVW: OR (95% CI): 0.93 (0.89, 0.98), *p* = 0.006; MR-Egger: OR (95% CI): 0.92 (0.83, 1.02), *p* = 0.219; weighted median: OR (95% CI): 0.93 (0.88, 0.99), *p* = 0.023) (Figs. 2 and 3b and see Supplementary Table 10, Additional File 1). In MR-PRESSO global test and MR-Egger test, no evidence of pleiotropy across SNPs was found as well (see Supplementary Table 10, Additional File 1). Using the leave-one-out analysis and the single SNP analysis, we observed that there was no outlying genetic variant that had a significant influence on the estimate (see Supplementary Tables 11 and 12, Additional File 1 and Supplementary Figs. 14 and 15, Additional File 2). There was also no heterogeneity observed in the causal estimate for the cuneus (see Supplementary Table 13, Additional File 1).

### The causal effect of AD on the cortical surface area

**Fig. 4:**
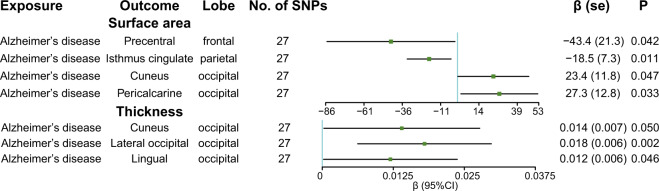
The causal effect of genetically predicted Alzheimer’s disease on cortical structure. SNP single-nucleotide polymorphism, *β* changes in cortical thickness in population with the disease compared with controls, SE standard error, CI confidence interval, IVW inverse-variance weighted.

**Fig. 5:**
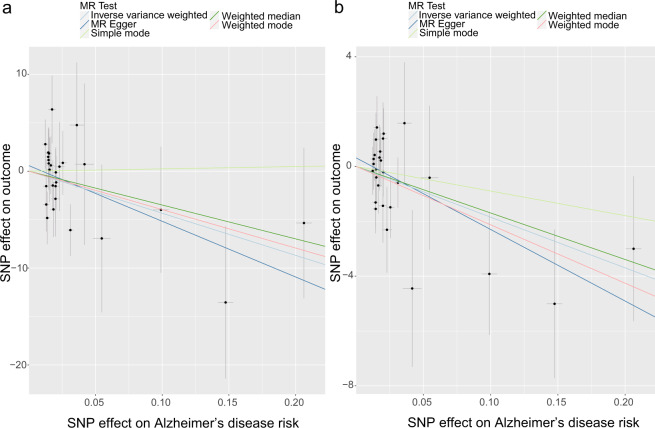
Scatterplot of single-nucleotide polymorphism (SNP) associated with Alzheimer’s disease and cortical structure. **a** Scatterplot of SNP associated with AD versus the cortical surface area of precentral. **b** Scatterplot of SNP associated with AD versus cortical surface area of isthmus cingulate. AD Alzheimer’s disease, SNP single-nucleotide polymorphism. Vertical and horizontal lines around each SNP show 95% confidence interval.

The results of the causal effect of AD on the cortical surface area are shown in Fig. 4. And the full results of the causal estimates for the vulnerability to AD on all brain regions are shown in Supplementary Fig. 16, Additional File 2. Our further investigation revealed that AD was suggestively associated with the decreased surface area of the precentral (*β* (SE): –43.4 (21.3), *p* = 0.042) and isthmus cingulate (*β* (SE): –18.5 (7.3); *p* = 0.011) (Figs. 4 and 5a, b) with no observed pleiotropy (see Supplementary Table 14, Additional File 1). The causal estimates of the isthmus cingulate was confirmed by the sensitivity analysis (MR-Egger: *β* (SE): –26.1 (10.4), *p* = 0.018) (Fig. 5b and see Supplementary Table 14, Additional File 1). In the leave-one-out analysis and the single SNP analysis, rs4663105 (near *BIN1* gene) was driving the main effect in the causal estimate for AD on the precentral and the results of the isthmus cingulate were not influenced by a single genetic variant (see Supplementary Tables 15 and 16, Additional File 1 and Supplementary Figs. 17–20, Additional File 2). In addition, we also found that AD was suggestively associated with the increased surface area of the cuneus (*β* (SE): 23.4 (11.8); *p* = 0.047) and pericalcarine (*β* (SE): 27.3 (12.8); *p* = 0.033) (see Supplementary Figs. 21 and 22, Additional File 2). However, rs11257238 (near *ECHDC3* gene) and rs4575098 (near *ADAMTS4* gene) were driving the main effect in the causal estimate for AD on the cuneus and pericalcarine respectively (see Supplementary Tables 15 and 16, Additional File 1 and Supplementary Figs. 23–26, Additional File 2). Besides, there was also evidence of potential heterogeneity in the genetic variants for the causal estimate for AD on the surface area of the cuneus (Cochran *Q* test *p* = 0.042 for MR-Egger) (see Supplementary Table 17, Additional File 1).

### The causal effect of AD on cortical thickness

The full results of the causal estimate for the vulnerability to AD on all brain regions are shown in Supplementary Fig. 27, Additional File 2. Genetically predicted AD was suggestively associated with increased cortical thickness of the cuneus (*β* (SE): 0.014 (0.007), *p* = 0.05), lateral occipital (*β* (SE): 0.018 (0.006), *p* = 0.002), and lingual (*β* (SE): 0.012 (0.006), *p* = 0.046) (Fig. 4 and see Supplementary Figs. 28–30, Additional File 2). Besides, the significant association between AD and cortical thickness of the lateral occipital remained in sensitivity analyses (MR-Egger: *β* (SE): 0.022 (0.008), *p* = 0.014; weighted median: *β* (SE): 0.021 (0.008), *p* = 0.008) without biased by horizontal pleiotropy (*p* > 0.05) (see Supplementary Table 18, Additional File 1). The leave-one-out analysis and the single SNP analysis showed that rs442495 (near *ADAM10* gene) played an important role in the causal estimate for AD on the cuneus and no influential single genetic variant was found in the results of the lateral occipital and lingual (see Supplementary Tables 19 and 20, Additional File 1 and Supplementary Figs. 31–36, Additional File 2). And there was no heterogeneity in the genetic variants (see Supplementary Table 21, Additional File 1).

For those suggestive exposure–outcome pairs (associations that are nonsignificant after correction for multiple testing), *I*^2^_GX_ was calculated and they were all larger than 0.9, indicating there was no evidence for violation of the NOME assumption (see Supplementary Tables 6, 10, 14, and 18 Additional File 1). We also calculated *F*-statistics, *R*^2^, and power for the causal estimate for the genetically predicted cortical structure on AD. Although the *F*-statistics were all greater than 10, the power was relatively low (see Supplementary Tables 22 and 23, Additional File 1).

## Discussion

In the present study, we investigated the causal relationships between cortical structure (cortical surface area and cortical thickness of whole cortex and 34 brain regions) and AD using the large-scale GWAS data that could provide reliable evidence for the causal relationship. It was shown that the atrophy of the temporal pole and cuneus is associated with an increased risk of AD. On the other hand, there was a suggestive causal association of the vulnerability to AD with a decrease in the surface area of precentral and isthmus cingulate. The vulnerability to AD might result in an increased volume of the occipital lobe was also found.

The inverse causal association between the surface area of the temporal pole and AD risk was consistent with previous studies showing that the temporal pole atrophy in AD patients [31, 32]. Parenthetically, the temporal pole, which was one of the regions with the greatest baseline cortical thickness in the cognitively normal stage, displayed the greatest degree of atrophy related to AD [33]. However, only four available SNPs were used as IV in the causal estimation between the surface area of the temporal pole and AD risk; the role of temporal pole atrophy in the course of AD needs further investigation.

Besides, the increase in the thickness of the cuneus was suggestively associated with a decreased risk of AD, which showed a similar pattern of disease progression in AD patients who suffered from posterior cortical atrophy. Posterior cortical atrophy was introduced by Benson et al. [34], and individuals with this syndrome display preserved episodic memory function in the early stage. Although multifarious pathologies may contribute to posterior cortical atrophy, AD is the most common pathologic cause [35]. Thus, it can be inferred that the cuneus atrophy occurs before the episodic memory loss and it may contribute to the AD risk.

Our findings showed that AD was causally associated with atrophy of the precentral gyrus. It is worth noting that motor impairments were reported both in the 5XFAD mouse model of AD [36] and in AD patients [37], and AD patients were found to possess a higher rate of gait disturbance than controls [38]. Besides, the precentral gyrus has been reported to be active in cognitive activity, such as short-term memory tasks [39, 40], which further support its connection with AD. The relationship between the precentral gyrus and AD was also reported in a previous study [41], and the precentral gyrus was found to have a reduction of gray matter volumes in carriers of the apolipoprotein E (APOE)-ε4 [42], which has been recognized to be a risk gene for AD. Thus, it is of great importance to figure out the role of the change of brain microstructural of the precentral gyrus in the progression of AD.

More and more studies demonstrated the limbic-predominant atrophy pattern of AD [43–45]: the thickness of the isthmus cingulate was reported to decrease in AD patients and it was also shown to be associated with the cognitive level [46, 47]. Our results further support the role of the isthmus cingulate in the progression of AD. However, the causal relationship between AD and the atrophy of isthmus cingulate was only found in the surface area instead of the thickness of the isthmus cingulate, which may be related to the pattern of neuronal loss. Further study is needed to elucidate the relationship between AD and the atrophy of isthmus cingulate.

Unexpectedly, we found a causal relationship between genetically predicted AD and increased volume of the occipital lobe (increased surface area of cuneus and pericalcarine, as well as increased thickness of cuneus, lateral occipital, and lingual). This was inconsistent with a previous study reporting occipital lobe atrophy with the development of AD [48]. However, a previous work reported that the uptake rates of ^18^F-florbetapir in the occipital lobe were higher in Aβ CSF-positive/PET-positive group compared with the CSF-negative/PET-negative group [49]; the increased volume of the occipital cortex may be explained by the space-occupying effects of amyloid plaques. In addition, the presenilin-1 messenger RNA, an important gene responsible for early-onset familial AD, was reported to preferentially express in immature neurons, and might play vital roles in rat neurogenesis [50]. Thus, the genetically predicted AD and the increased volume of occipital lobe could also result from the neurogenesis [51]. Besides, no evidence was found for a causal relationship between the entorhinal or parahippocampal cortex and AD risk. Since typical AD only had a pooled frequency of 55% among the four different atrophy patterns of AD [43], we could not fully exclude this possibility and the limited sample size of the cortical structure GWAS. Further analysis is needed to investigate the causal relationship between different atrophy patterns of AD and brain structure.

There are some limitations in our study. First, we only used cortical surface area and thickness to explore the causal relationship between brain structure and AD, and more evaluation indicators can be used to further evaluate the causal relationship between brain structure and AD risk. Second, as the accuracy of Mendelian randomization estimate relies on the three assumptions, one of which is that SNPs used as instrument variables should be strongly associated with the exposure, the fraction of the total variance explained by SNPs was low in our study. Besides, in the Mendelian randomization analysis for the causal estimation for cortical structure on AD risk, the genome-wide significance threshold was set at *p* < 10^–6^ for lack of significant SNPs available, which may introduce weak IVs. To address this issue, a larger population is needed. Third, in two-sample Mendelian randomization analyses, there may be sample overlapping between exposure and outcome population that may potentially bias the results. Fourth, as the population we used was mainly Europeans, the results cannot be generalized to other ethnicities and races.

In conclusion, using information from large genetic consortia, we provided suggestive evidence that the atrophy of the temporal pole and cuneus is associated with higher AD risk. In the other direction, there was a suggestive causal association between the vulnerability to AD and a decrease in the surface area of the precentral and isthmus cingulate. To figure out the causal relationship between brain structure and AD, further investigation into the biological functions of these brain regions is needed.

## Supplementary information

Additional File 1

Additional File 2

## Data Availability

The datasets of the human cerebral cortex analyzed during the current study are available from the ENIGMA Consortium website, http://enigma.ini.usc.edu/research/download-enigma-gwas-results/. The datasets of the Alzheimer’s disease analyzed during the current study are available from https://ctg.cncr.nl/.
