# Supplementary material for: Cortical structure and the risk for Alzheimer’s disease: a bidirectional Mendelian randomization study
Source: Transl Psychiatry. 2021 Sep 15;11:476. doi: 10.1038/s41398-021-01599-x (PMC8443658; doi:10.1038/s41398-021-01599-x)
Supplement: Supplementary file 1 — Additional File 1 [file 41398_2021_1599_MOESM1_ESM.docx]

**Supporting Information 1:** Supplementary tables

| **Table** | **Contents** | **Page** |
| --- | --- | --- |
| Table S1 | GWAS data sets used in the present MR analysis | 2 |
| Table S2 | Summary of genetic variants used to estimate the effect of cortical surface area on Alzheimer's disease (AD) risk | 3 |
| Table S3 | Summary of genetic variants used to estimate the effect of cortical thickness on Alzheimer's disease (AD) risk | 22 |
| Table S4 | Summary of genetic variants used to estimate the effect of Alzheimer's disease (AD) on cortical surface area | 32 |
| Table S5 | Summary of genetic variants used to estimate the effect of Alzheimer's disease (AD) on cortical thickness | 70 |
| Table S6 | Sensitivity analysis and pleiotropy analysis for causal effect of cortical surface area on Alzheimer’s Disease. | 108 |
| Table S7 | Leave-one-out(loo) analysis of association between genetically predicted cortical surface area and Alzheimer's disease (AD) risk. | 111 |
| Table S8 | Single SNP analysis of association between genetically predicted cortical surface area and Alzheimer's disease (AD) risk. | 122 |
| Table S9 | Heterogeneity analysis of association between genetically predicted cortical surface area and Alzheimer's disease (AD) risk. | 133 |
| Table S10 | Sensitivity analysis and pleiotropy analysis for causal effect of cortical thickness on Alzheimer’s Disease. | 135 |
| Table S11 | Leave-one-out(loo) analysis of association between genetically predicted cortical thickness and Alzheimer's disease (AD) risk. | 138 |
| Table S12 | Single SNP analysis of association between genetically predicted cortical thickness and Alzheimer's disease (AD) risk. | 144 |
| Table S13 | Heterogeneity analysis of association between genetically predicted cortical thickness and Alzheimer's disease (AD) risk. | 150 |
| Table S14 | Sensitivity analysis and pleiotropy analysis for causal effect of Alzheimer’s Disease on cortical surface area. | 152 |
| Table S15 | Leave-one-out(loo) analysis of association between genetically predicted Alzheimer's disease (AD) on cortical surface area. | 155 |
| Table S16 | Single SNP analysis of association between genetically predicted Alzheimer's disease (AD) and cortical surface area. | 178 |
| Table S17 | Heterogeneity analysis of association between genetically predicted Alzheimer's disease (AD) and cortical surface area. | 201 |
| Table S18 | Sensitivity analysis and pleiotropy analysis for causal effect of Alzheimer’s Disease on cortical thickness. | 204 |
| Table S19 | Leave-one-out(loo) analysis of association between genetically predicted Alzheimer's disease (AD) on cortical thickness. | 207 |
| Table S20 | Single SNP analysis of association between genetically predicted Alzheimer's disease (AD) and cortical thickness. | 230 |
| Table S21 | Heterogeneity analysis of association between genetically predicted Alzheimer's disease (AD) and cortical thickness. | 253 |
| Table S22 | The calculated F-statistics , R^2^ and power for causal effect of cortical structure on Alzheimer’s Disease. | 256 |
| Table S23 | The calculated F-statistics and R^2^ for causal effect of Alzheimer’s Disease on cortical structure. | 258 |

**Table S1.** GWAS data sets used in the present MR analysis

| **Traits** | **PubMed ID** | **Population** | **Sample size or Cases/Controls** |
| --- | --- | --- | --- |
| Cortical phenotype | 32193296 | European | 33992 |
| AD | 30617256 | European | 71880/383378 |
| PGC-ALZ |  | European | 2736/14741 |
| IGAP |  | European | 17008/37154 |
| ADSP |  | European | 4343/3163 |
| UKB |  | European | 47793/328320 |

AD, Alzheimer disease; ADSP, the Alzheimer’s Disease Sequencing Project; IGAP, the International Genomics of Alzheimer’s Project; PGC-ALZ, Alzheimer’s disease working group of the Psychiatric Genomics Consortium; UKB, the UK Biobank.

**Table S2.** Summary of genetic variants used to estimate the effect of cortical surface area on Alzheimer's disease (AD) risk

| **Exposure** | **lobe** | **outcome** | **SNP** | **effect allele** | **other allele** | **chr** | **beta exposure** | **se exposure** | **pval exposure** | **beta outcome** | **se outcome** | **pval outcome** |
| --- | --- | --- | --- | --- | --- | --- | --- | --- | --- | --- | --- | --- |
| caudal anterior cingulate | frontal | AD | rs10845985 | A | G | 12 | 0.03367 | 0.00647 | 1.98E-07 | -0.00155 | 0.00217 | 4.75E-01 |
| caudal anterior cingulate | frontal | AD | rs13021985 | A | G | 2 | 0.03790 | 0.00641 | 3.45E-09 | 0.00259 | 0.00220 | 2.40E-01 |
| caudal anterior cingulate | frontal | AD | rs2509765 | T | C | 8 | 0.04749 | 0.00915 | 2.10E-07 | 0.00129 | 0.00289 | 6.55E-01 |
| caudal anterior cingulate | frontal | AD | rs2647416 | T | C | 6 | -0.03229 | 0.00658 | 9.23E-07 | 0.00402 | 0.00221 | 6.94E-02 |
| caudal anterior cingulate | frontal | AD | rs4747503 | C | G | 10 | -0.03705 | 0.00651 | 1.29E-08 | -0.00445 | 0.00223 | 4.60E-02 |
| caudal anterior cingulate | frontal | AD | rs7728751 | A | G | 5 | 0.04471 | 0.00785 | 1.26E-08 | 0.00264 | 0.00265 | 3.18E-01 |
| caudal anterior cingulate | frontal | AD | rs80241863 | A | G | 16 | 0.22543 | 0.03793 | 2.79E-09 | 0.01121 | 0.01119 | 3.16E-01 |
| caudal middle frontal | frontal | AD | rs1261073 | A | C | 18 | 0.03229 | 0.00592 | 5.06E-08 | -0.00121 | 0.00220 | 5.83E-01 |
| caudal middle frontal | frontal | AD | rs2589230 | T | C | 15 | 0.06605 | 0.01322 | 5.80E-07 | 0.00340 | 0.00488 | 4.86E-01 |
| caudal middle frontal | frontal | AD | rs30641 | A | G | 5 | -0.04200 | 0.00678 | 5.80E-10 | -0.00356 | 0.00253 | 1.59E-01 |
| caudal middle frontal | frontal | AD | rs4273712 | A | G | 6 | -0.05773 | 0.00653 | 9.07E-19 | -0.00548 | 0.00242 | 2.35E-02 |
| caudal middle frontal | frontal | AD | rs448939 | T | C | 6 | 0.02923 | 0.00580 | 4.57E-07 | -0.00319 | 0.00216 | 1.40E-01 |
| caudal middle frontal | frontal | AD | rs655045 | T | C | 3 | -0.03510 | 0.00607 | 7.15E-09 | 0.00145 | 0.00220 | 5.10E-01 |
| caudal middle frontal | frontal | AD | rs7184835 | T | C | 16 | -0.03669 | 0.00578 | 2.19E-10 | 0.00161 | 0.00216 | 4.58E-01 |
| caudal middle frontal | frontal | AD | rs7356095 | A | G | 3 | -0.03228 | 0.00657 | 8.97E-07 | 0.00102 | 0.00237 | 6.67E-01 |
| caudal middle frontal | frontal | AD | rs7562708 | T | G | 2 | 0.03281 | 0.00591 | 2.80E-08 | 0.00099 | 0.00225 | 6.60E-01 |
| caudal middle frontal | frontal | AD | rs9345125 | A | G | 6 | 0.04897 | 0.00749 | 6.32E-11 | -0.00065 | 0.00279 | 8.17E-01 |
| caudal middle frontal | frontal | AD | rs9402979 | T | C | 6 | -0.03449 | 0.00669 | 2.51E-07 | -0.00345 | 0.00245 | 1.59E-01 |
| frontal pole | frontal | AD | rs1054442 | A | C | 12 | 0.03623 | 0.00722 | 5.25E-07 | -0.00241 | 0.00222 | 2.79E-01 |
| frontal pole | frontal | AD | rs113050298 | T | C | 16 | -0.08479 | 0.01577 | 7.58E-08 | -0.00296 | 0.00465 | 5.24E-01 |
| frontal pole | frontal | AD | rs114620601 | C | G | 5 | 0.16793 | 0.03315 | 4.06E-07 | -0.00037 | 0.00955 | 9.69E-01 |
| frontal pole | frontal | AD | rs17464221 | T | C | 2 | -0.04737 | 0.00775 | 9.83E-10 | -0.00413 | 0.00236 | 7.93E-02 |
| frontal pole | frontal | AD | rs403351 | A | G | 8 | 0.08304 | 0.01655 | 5.23E-07 | -0.00318 | 0.00402 | 4.29E-01 |
| frontal pole | frontal | AD | rs872505 | T | C | 1 | -0.03440 | 0.00696 | 7.87E-07 | 0.00287 | 0.00218 | 1.89E-01 |
| lateral orbitofrontal | frontal | AD | rs10179285 | C | G | 2 | 0.02601 | 0.00502 | 2.25E-07 | 0.00048 | 0.00222 | 8.30E-01 |
| lateral orbitofrontal | frontal | AD | rs10916258 | A | C | 1 | 0.03121 | 0.00617 | 4.24E-07 | 0.00083 | 0.00271 | 7.60E-01 |
| lateral orbitofrontal | frontal | AD | rs11012730 | A | G | 10 | -0.02590 | 0.00506 | 3.09E-07 | -0.00029 | 0.00225 | 8.99E-01 |
| lateral orbitofrontal | frontal | AD | rs12626790 | A | G | 21 | -0.02838 | 0.00498 | 1.18E-08 | -0.00089 | 0.00226 | 6.93E-01 |
| lateral orbitofrontal | frontal | AD | rs12652639 | T | C | 5 | 0.02425 | 0.00487 | 6.48E-07 | 0.00271 | 0.00530 | 6.09E-01 |
| lateral orbitofrontal | frontal | AD | rs13208234 | A | G | 6 | -0.02725 | 0.00497 | 4.25E-08 | -0.00523 | 0.00220 | 1.72E-02 |
| lateral orbitofrontal | frontal | AD | rs1822951 | A | G | 8 | -0.02751 | 0.00503 | 4.66E-08 | 0.00047 | 0.00222 | 8.34E-01 |
| lateral orbitofrontal | frontal | AD | rs2237133 | T | G | 6 | -0.02531 | 0.00498 | 3.80E-07 | -0.00723 | 0.01071 | 4.99E-01 |
| lateral orbitofrontal | frontal | AD | rs2358483 | T | C | 14 | -0.03930 | 0.00524 | 6.19E-14 | 0.00245 | 0.00243 | 3.13E-01 |
| lateral orbitofrontal | frontal | AD | rs4721802 | A | G | 7 | -0.04032 | 0.00508 | 2.06E-15 | -0.00180 | 0.00225 | 4.23E-01 |
| lateral orbitofrontal | frontal | AD | rs4897178 | T | G | 6 | -0.04352 | 0.00504 | 6.03E-18 | 0.00708 | 0.00530 | 1.82E-01 |
| lateral orbitofrontal | frontal | AD | rs56329255 | A | G | 11 | 0.02677 | 0.00526 | 3.52E-07 | 0.00008 | 0.00241 | 9.72E-01 |
| lateral orbitofrontal | frontal | AD | rs6729276 | A | G | 2 | 0.02466 | 0.00493 | 5.71E-07 | 0.00276 | 0.00222 | 2.14E-01 |
| lateral orbitofrontal | frontal | AD | rs6737150 | T | C | 2 | 0.03068 | 0.00581 | 1.28E-07 | 0.00027 | 0.00245 | 9.14E-01 |
| lateral orbitofrontal | frontal | AD | rs67616210 | A | G | 5 | -0.02659 | 0.00512 | 2.06E-07 | -0.00271 | 0.00231 | 2.40E-01 |
| lateral orbitofrontal | frontal | AD | rs6949868 | A | C | 7 | -0.02800 | 0.00567 | 7.81E-07 | -0.00174 | 0.00256 | 4.96E-01 |
| lateral orbitofrontal | frontal | AD | rs7252428 | C | G | 19 | 0.03209 | 0.00618 | 2.09E-07 | 0.00653 | 0.00244 | 7.52E-03 |
| lateral orbitofrontal | frontal | AD | rs7529542 | T | C | 1 | -0.03745 | 0.00639 | 4.54E-09 | -0.00137 | 0.00258 | 5.96E-01 |
| lateral orbitofrontal | frontal | AD | rs7621856 | T | C | 3 | 0.02465 | 0.00498 | 7.60E-07 | -0.00104 | 0.00222 | 6.39E-01 |
| lateral orbitofrontal | frontal | AD | rs77692431 | A | G | 11 | 0.04835 | 0.00914 | 1.22E-07 | 0.00356 | 0.00387 | 3.57E-01 |
| lateral orbitofrontal | frontal | AD | rs79487293 | T | C | 12 | -0.03093 | 0.00566 | 4.77E-08 | -0.00182 | 0.00234 | 4.39E-01 |
| medial orbitofrontal | frontal | AD | rs117833963 | T | C | 6 | 0.13938 | 0.02815 | 7.35E-07 | -0.00522 | 0.01204 | 6.65E-01 |
| medial orbitofrontal | frontal | AD | rs2446113 | A | C | 11 | 0.02785 | 0.00536 | 2.05E-07 | 0.00204 | 0.00236 | 3.89E-01 |
| medial orbitofrontal | frontal | AD | rs2609186 | A | G | 2 | -0.02598 | 0.00528 | 8.59E-07 | 0.00033 | 0.00233 | 8.88E-01 |
| medial orbitofrontal | frontal | AD | rs7097933 | A | G | 10 | 0.02752 | 0.00499 | 3.59E-08 | 0.00020 | 0.00224 | 9.29E-01 |
| medial orbitofrontal | frontal | AD | rs9375435 | T | C | 6 | 0.02553 | 0.00488 | 1.69E-07 | 0.00204 | 0.00216 | 3.45E-01 |
| paracentral | frontal | AD | rs10064431 | T | C | 5 | 0.03368 | 0.00656 | 2.83E-07 | -0.00122 | 0.00216 | 5.73E-01 |
| paracentral | frontal | AD | rs10850057 | T | C | 12 | 0.05559 | 0.01082 | 2.81E-07 | 0.00218 | 0.00355 | 5.40E-01 |
| paracentral | frontal | AD | rs12146713 | T | C | 12 | -0.05711 | 0.01073 | 1.02E-07 | 0.00144 | 0.00414 | 7.28E-01 |
| paracentral | frontal | AD | rs13147448 | T | C | 4 | -0.04889 | 0.00963 | 3.86E-07 | -0.00009 | 0.00316 | 9.78E-01 |
| paracentral | frontal | AD | rs2269084 | C | G | 10 | -0.04098 | 0.00735 | 2.43E-08 | 0.00201 | 0.00260 | 4.38E-01 |
| paracentral | frontal | AD | rs2346756 | C | G | 12 | 0.03531 | 0.00621 | 1.33E-08 | -0.00043 | 0.00534 | 9.36E-01 |
| paracentral | frontal | AD | rs2760751 | A | G | 17 | 0.03505 | 0.00678 | 2.38E-07 | 0.00019 | 0.00244 | 9.37E-01 |
| paracentral | frontal | AD | rs62515458 | A | G | 8 | 0.03615 | 0.00731 | 7.64E-07 | -0.00698 | 0.00257 | 6.60E-03 |
| paracentral | frontal | AD | rs6582653 | T | C | 12 | -0.03891 | 0.00764 | 3.55E-07 | 0.00851 | 0.01191 | 4.75E-01 |
| paracentral | frontal | AD | rs9565516 | A | T | 13 | -0.03359 | 0.00635 | 1.22E-07 | -0.00142 | 0.00242 | 5.58E-01 |
| parsopercularis | frontal | AD | rs11107546 | T | C | 12 | 0.08259 | 0.01613 | 3.06E-07 | -0.00106 | 0.00237 | 6.53E-01 |
| parsopercularis | frontal | AD | rs1159974 | T | C | 6 | -0.08777 | 0.01468 | 2.22E-09 | -0.00388 | 0.00216 | 7.22E-02 |
| parsopercularis | frontal | AD | rs12938190 | T | C | 17 | 0.08194 | 0.01494 | 4.13E-08 | -0.00130 | 0.00219 | 5.54E-01 |
| parsopercularis | frontal | AD | rs2033939 | A | G | 15 | -0.25728 | 0.02712 | 2.41E-21 | -0.00881 | 0.00404 | 2.93E-02 |
| parsopercularis | frontal | AD | rs2279829 | T | C | 3 | 0.08913 | 0.01780 | 5.55E-07 | -0.00537 | 0.00260 | 3.90E-02 |
| parsopercularis | frontal | AD | rs441890 | T | C | 8 | 0.08155 | 0.01483 | 3.86E-08 | 0.00166 | 0.00219 | 4.47E-01 |
| parsopercularis | frontal | AD | rs7550758 | T | C | 1 | -0.07794 | 0.01472 | 1.20E-07 | -0.00567 | 0.00216 | 8.74E-03 |
| parsorbitalis | frontal | AD | rs10901380 | A | G | 10 | -0.02771 | 0.00553 | 5.49E-07 | 0.00061 | 0.00231 | 7.93E-01 |
| parsorbitalis | frontal | AD | rs139214174 | A | C | 6 | 0.07887 | 0.01581 | 6.03E-07 | -0.00148 | 0.00586 | 8.01E-01 |
| parsorbitalis | frontal | AD | rs1503738 | A | G | 3 | 0.03303 | 0.00585 | 1.68E-08 | -0.00454 | 0.00228 | 4.66E-02 |
| parsorbitalis | frontal | AD | rs2287283 | T | C | 2 | 0.03494 | 0.00573 | 1.06E-09 | -0.00107 | 0.00225 | 6.34E-01 |
| parsorbitalis | frontal | AD | rs2396373 | A | G | 6 | -0.02963 | 0.00569 | 1.93E-07 | -0.00226 | 0.00221 | 3.06E-01 |
| parsorbitalis | frontal | AD | rs61901866 | T | C | 11 | 0.05563 | 0.00868 | 1.47E-10 | -0.00184 | 0.00321 | 5.66E-01 |
| parsorbitalis | frontal | AD | rs7147119 | A | G | 14 | -0.03839 | 0.00578 | 3.02E-11 | 0.00177 | 0.00230 | 4.42E-01 |
| parsorbitalis | frontal | AD | rs72673107 | A | G | 8 | -0.03909 | 0.00779 | 5.18E-07 | 0.00293 | 0.00303 | 3.34E-01 |
| parsorbitalis | frontal | AD | rs72691108 | A | G | 1 | -0.04247 | 0.00659 | 1.17E-10 | 0.00026 | 0.00254 | 9.20E-01 |
| parsorbitalis | frontal | AD | rs9329203 | A | G | 8 | -0.03214 | 0.00642 | 5.56E-07 | 0.00309 | 0.00245 | 2.07E-01 |
| parsorbitalis | frontal | AD | rs9875836 | T | C | 3 | -0.02897 | 0.00559 | 2.16E-07 | -0.00171 | 0.00217 | 4.31E-01 |
| parstriangularis | frontal | AD | rs10058365 | A | G | 5 | 0.09472 | 0.01838 | 2.56E-07 | -0.00742 | 0.00269 | 5.89E-03 |
| parstriangularis | frontal | AD | rs10278627 | A | G | 7 | -0.17128 | 0.01579 | 2.09E-27 | 0.00167 | 0.00226 | 4.60E-01 |
| parstriangularis | frontal | AD | rs1125867 | A | G | 19 | -0.09480 | 0.01753 | 6.36E-08 | 0.00114 | 0.00250 | 6.48E-01 |
| parstriangularis | frontal | AD | rs2144366 | C | G | 6 | 0.11152 | 0.02174 | 2.89E-07 | 0.00161 | 0.00337 | 6.32E-01 |
| parstriangularis | frontal | AD | rs2279829 | T | C | 3 | 0.23249 | 0.01784 | 8.35E-39 | -0.00537 | 0.00260 | 3.90E-02 |
| parstriangularis | frontal | AD | rs2287283 | T | C | 2 | 0.09852 | 0.01513 | 7.51E-11 | -0.00107 | 0.00225 | 6.34E-01 |
| parstriangularis | frontal | AD | rs2999980 | A | G | 10 | 0.09426 | 0.01866 | 4.36E-07 | -0.00159 | 0.00269 | 5.55E-01 |
| parstriangularis | frontal | AD | rs4920605 | A | G | 1 | -0.07499 | 0.01498 | 5.55E-07 | -0.00461 | 0.00216 | 3.29E-02 |
| parstriangularis | frontal | AD | rs4924345 | A | C | 15 | 0.17647 | 0.02721 | 8.80E-11 | 0.00835 | 0.00401 | 3.74E-02 |
| parstriangularis | frontal | AD | rs55818129 | T | G | 5 | -0.12868 | 0.02469 | 1.86E-07 | -0.00102 | 0.00348 | 7.70E-01 |
| parstriangularis | frontal | AD | rs5750482 | T | C | 22 | -0.07885 | 0.01517 | 2.01E-07 | 0.00374 | 0.00220 | 8.84E-02 |
| parstriangularis | frontal | AD | rs59614433 | T | C | 11 | -0.16669 | 0.02816 | 3.24E-09 | 0.00501 | 0.00397 | 2.07E-01 |
| parstriangularis | frontal | AD | rs6443469 | T | C | 3 | 0.07698 | 0.01527 | 4.62E-07 | -0.00096 | 0.00221 | 6.63E-01 |
| parstriangularis | frontal | AD | rs6867851 | C | G | 5 | -0.07521 | 0.01519 | 7.36E-07 | -0.00128 | 0.00222 | 5.65E-01 |
| parstriangularis | frontal | AD | rs75187227 | A | G | 4 | 0.18230 | 0.03572 | 3.33E-07 | 0.00420 | 0.00497 | 3.97E-01 |
| parstriangularis | frontal | AD | rs7967462 | A | T | 12 | 0.21379 | 0.04185 | 3.24E-07 | -0.00261 | 0.00553 | 6.37E-01 |
| parstriangularis | frontal | AD | rs7996803 | T | C | 13 | -0.09788 | 0.01651 | 3.09E-09 | -0.00301 | 0.00243 | 2.15E-01 |
| precentral | frontal | AD | rs10064431 | T | C | 5 | 0.03084 | 0.00522 | 3.50E-09 | -0.00122 | 0.00216 | 5.73E-01 |
| precentral | frontal | AD | rs1080066 | A | G | 15 | -0.21403 | 0.00859 | 3.81E-137 | 0.00872 | 0.00406 | 3.17E-02 |
| precentral | frontal | AD | rs17756000 | T | C | 14 | -0.02871 | 0.00568 | 4.24E-07 | 0.00248 | 0.00257 | 3.35E-01 |
| precentral | frontal | AD | rs189874581 | T | C | 15 | -0.08523 | 0.01387 | 7.91E-10 | 0.04158 | 0.02919 | 1.54E-01 |
| precentral | frontal | AD | rs2929680 | C | G | 15 | 0.02832 | 0.00511 | 2.96E-08 | -0.00619 | 0.00550 | 2.61E-01 |
| precentral | frontal | AD | rs4499967 | T | C | 6 | -0.04056 | 0.00810 | 5.61E-07 | 0.00214 | 0.00357 | 5.49E-01 |
| precentral | frontal | AD | rs4706392 | A | T | 6 | 0.05095 | 0.00630 | 6.22E-16 | -0.00049 | 0.00277 | 8.59E-01 |
| precentral | frontal | AD | rs4751614 | A | T | 10 | 0.03390 | 0.00577 | 4.18E-09 | 0.00458 | 0.00259 | 7.68E-02 |
| precentral | frontal | AD | rs4794859 | T | G | 17 | 0.02605 | 0.00490 | 1.04E-07 | 0.00376 | 0.00217 | 8.23E-02 |
| precentral | frontal | AD | rs7134627 | A | C | 12 | 0.02484 | 0.00503 | 7.67E-07 | 0.00152 | 0.00528 | 7.74E-01 |
| precentral | frontal | AD | rs7868648 | T | G | 9 | 0.03010 | 0.00571 | 1.37E-07 | -0.00182 | 0.00257 | 4.78E-01 |
| precentral | frontal | AD | rs853974 | T | C | 6 | 0.03023 | 0.00559 | 6.33E-08 | 0.00587 | 0.00244 | 1.61E-02 |
| rostral anterior cingulate | frontal | AD | rs1178101 | A | C | 7 | 0.04121 | 0.00745 | 3.14E-08 | -0.00068 | 0.00292 | 8.16E-01 |
| rostral anterior cingulate | frontal | AD | rs1986012 | T | C | 15 | -0.03302 | 0.00648 | 3.47E-07 | -0.00217 | 0.00245 | 3.76E-01 |
| rostral anterior cingulate | frontal | AD | rs2202895 | T | C | 17 | -0.03908 | 0.00678 | 8.22E-09 | -0.00086 | 0.00257 | 7.37E-01 |
| rostral anterior cingulate | frontal | AD | rs7874287 | T | C | 9 | -0.03306 | 0.00620 | 9.70E-08 | -0.00132 | 0.00237 | 5.79E-01 |
| rostral anterior cingulate | frontal | AD | rs797832 | T | C | 7 | -0.02915 | 0.00573 | 3.68E-07 | -0.00388 | 0.00218 | 7.61E-02 |
| rostral anterior cingulate | frontal | AD | rs9905914 | A | G | 17 | 0.02942 | 0.00587 | 5.44E-07 | 0.00316 | 0.00218 | 1.47E-01 |
| rostral middle frontal | frontal | AD | rs10139160 | T | C | 14 | 0.02112 | 0.00410 | 2.61E-07 | 0.00086 | 0.00218 | 6.92E-01 |
| rostral middle frontal | frontal | AD | rs10264709 | C | G | 7 | -0.02468 | 0.00493 | 5.69E-07 | 0.00120 | 0.00264 | 6.49E-01 |
| rostral middle frontal | frontal | AD | rs10283100 | A | G | 8 | 0.06934 | 0.01055 | 5.04E-11 | 0.01307 | 0.00487 | 7.20E-03 |
| rostral middle frontal | frontal | AD | rs1080066 | A | G | 15 | 0.04445 | 0.00724 | 8.16E-10 | 0.00872 | 0.00406 | 3.17E-02 |
| rostral middle frontal | frontal | AD | rs1165645 | A | G | 3 | 0.02395 | 0.00428 | 2.17E-08 | -0.00017 | 0.00220 | 9.38E-01 |
| rostral middle frontal | frontal | AD | rs1257415 | A | C | 14 | 0.02264 | 0.00417 | 5.61E-08 | -0.00349 | 0.00218 | 1.08E-01 |
| rostral middle frontal | frontal | AD | rs13019832 | A | G | 2 | -0.02172 | 0.00413 | 1.42E-07 | -0.00254 | 0.00221 | 2.52E-01 |
| rostral middle frontal | frontal | AD | rs1431272 | C | G | 13 | 0.02489 | 0.00475 | 1.59E-07 | -0.00150 | 0.00256 | 5.57E-01 |
| rostral middle frontal | frontal | AD | rs194242 | A | G | 2 | 0.02211 | 0.00438 | 4.52E-07 | -0.00051 | 0.00234 | 8.28E-01 |
| rostral middle frontal | frontal | AD | rs2276133 | A | G | 11 | -0.02528 | 0.00470 | 7.57E-08 | -0.00003 | 0.00249 | 9.89E-01 |
| rostral middle frontal | frontal | AD | rs2279829 | T | C | 3 | 0.02592 | 0.00496 | 1.72E-07 | -0.00537 | 0.00260 | 3.90E-02 |
| rostral middle frontal | frontal | AD | rs28645132 | A | G | 5 | -0.02364 | 0.00449 | 1.40E-07 | -0.00253 | 0.00246 | 3.04E-01 |
| rostral middle frontal | frontal | AD | rs35612915 | A | G | 2 | -0.03055 | 0.00483 | 2.52E-10 | -0.00306 | 0.00250 | 2.22E-01 |
| rostral middle frontal | frontal | AD | rs36006722 | A | G | 13 | 0.02299 | 0.00432 | 1.07E-07 | -0.00171 | 0.00225 | 4.47E-01 |
| rostral middle frontal | frontal | AD | rs40115 | T | C | 16 | -0.02357 | 0.00422 | 2.39E-08 | 0.00005 | 0.00228 | 9.82E-01 |
| rostral middle frontal | frontal | AD | rs4670555 | T | C | 2 | 0.02102 | 0.00409 | 2.69E-07 | -0.00332 | 0.00217 | 1.26E-01 |
| rostral middle frontal | frontal | AD | rs4722006 | T | C | 7 | -0.02031 | 0.00410 | 7.47E-07 | -0.00421 | 0.00219 | 5.43E-02 |
| rostral middle frontal | frontal | AD | rs6682671 | T | C | 1 | 0.02646 | 0.00436 | 1.27E-09 | -0.00452 | 0.00222 | 4.24E-02 |
| rostral middle frontal | frontal | AD | rs7529537 | T | C | 1 | 0.03618 | 0.00674 | 7.94E-08 | -0.00270 | 0.00336 | 4.21E-01 |
| rostral middle frontal | frontal | AD | rs77640487 | T | C | 3 | -0.05533 | 0.01090 | 3.86E-07 | 0.00371 | 0.00508 | 4.65E-01 |
| rostral middle frontal | frontal | AD | rs9971479 | C | G | 11 | -0.02327 | 0.00471 | 7.97E-07 | 0.00163 | 0.00254 | 5.21E-01 |
| superior frontal | frontal | AD | rs10835817 | A | G | 11 | -0.01887 | 0.00380 | 6.92E-07 | 0.00132 | 0.00219 | 5.48E-01 |
| superior frontal | frontal | AD | rs12989517 | A | G | 2 | -0.01922 | 0.00390 | 8.18E-07 | 0.00073 | 0.00225 | 7.46E-01 |
| superior frontal | frontal | AD | rs140562220 | T | C | 1 | -0.06431 | 0.01301 | 7.72E-07 | -0.00391 | 0.01404 | 7.81E-01 |
| superior frontal | frontal | AD | rs142301939 | A | G | 6 | 0.02512 | 0.00410 | 8.81E-10 | 0.00826 | 0.01104 | 4.55E-01 |
| superior frontal | frontal | AD | rs1473608 | T | G | 15 | -0.02433 | 0.00497 | 9.82E-07 | -0.00077 | 0.00283 | 7.86E-01 |
| superior frontal | frontal | AD | rs17669337 | T | C | 5 | -0.02095 | 0.00382 | 4.12E-08 | -0.00167 | 0.00220 | 4.48E-01 |
| superior frontal | frontal | AD | rs28704635 | A | T | 9 | 0.02336 | 0.00448 | 1.87E-07 | -0.00261 | 0.00260 | 3.15E-01 |
| superior frontal | frontal | AD | rs354085 | T | C | 7 | -0.02142 | 0.00434 | 7.93E-07 | -0.01488 | 0.01217 | 2.21E-01 |
| superior frontal | frontal | AD | rs4842266 | A | G | 12 | -0.02647 | 0.00409 | 1.01E-10 | 0.00361 | 0.00224 | 1.06E-01 |
| superior frontal | frontal | AD | rs4843560 | A | G | 16 | 0.01934 | 0.00392 | 8.01E-07 | -0.00112 | 0.00222 | 6.13E-01 |
| superior frontal | frontal | AD | rs4915928 | A | G | 1 | 0.03329 | 0.00537 | 5.82E-10 | -0.00235 | 0.00313 | 4.52E-01 |
| superior frontal | frontal | AD | rs628380 | C | G | 3 | 0.02212 | 0.00436 | 3.84E-07 | -0.00527 | 0.00258 | 4.12E-02 |
| superior frontal | frontal | AD | rs6912198 | A | G | 6 | -0.01967 | 0.00395 | 6.59E-07 | 0.00458 | 0.00529 | 3.87E-01 |
| superior frontal | frontal | AD | rs76696867 | A | G | 9 | -0.04318 | 0.00776 | 2.67E-08 | -0.00248 | 0.00489 | 6.13E-01 |
| bankssts | temporal | AD | rs160458 | T | C | 14 | -0.04308 | 0.00637 | 1.32E-11 | 0.00167 | 0.00218 | 4.41E-01 |
| bankssts | temporal | AD | rs2043294 | A | T | 19 | -0.04604 | 0.00864 | 9.91E-08 | 0.00405 | 0.00284 | 1.54E-01 |
| bankssts | temporal | AD | rs2573727 | A | G | 2 | -0.03483 | 0.00694 | 5.10E-07 | 0.00117 | 0.00230 | 6.09E-01 |
| bankssts | temporal | AD | rs62384380 | A | C | 5 | -0.13651 | 0.02741 | 6.35E-07 | 0.00540 | 0.00723 | 4.55E-01 |
| bankssts | temporal | AD | rs6565627 | T | C | 17 | -0.03742 | 0.00706 | 1.18E-07 | 0.00114 | 0.00238 | 6.32E-01 |
| bankssts | temporal | AD | rs73006822 | T | C | 19 | 0.05449 | 0.00958 | 1.28E-08 | 0.00325 | 0.00295 | 2.71E-01 |
| bankssts | temporal | AD | rs7862092 | T | G | 9 | 0.06547 | 0.01190 | 3.71E-08 | -0.00287 | 0.00396 | 4.69E-01 |
| bankssts | temporal | AD | rs9436222 | C | G | 1 | 0.03603 | 0.00696 | 2.27E-07 | -0.00589 | 0.00233 | 1.13E-02 |
| entorhinal | temporal | AD | rs12921392 | A | G | 16 | 0.03945 | 0.00709 | 2.59E-08 | -0.00011 | 0.00221 | 9.60E-01 |
| entorhinal | temporal | AD | rs141912254 | T | C | 3 | -0.12974 | 0.02487 | 1.83E-07 | 0.01108 | 0.00773 | 1.52E-01 |
| entorhinal | temporal | AD | rs17698176 | T | G | 17 | -0.05102 | 0.00960 | 1.05E-07 | 0.00103 | 0.00274 | 7.07E-01 |
| entorhinal | temporal | AD | rs2270027 | A | G | 7 | -0.04468 | 0.00892 | 5.47E-07 | 0.00590 | 0.00280 | 3.53E-02 |
| entorhinal | temporal | AD | rs247787 | A | G | 19 | -0.04772 | 0.00930 | 2.86E-07 | -0.00599 | 0.00285 | 3.54E-02 |
| entorhinal | temporal | AD | rs35004829 | A | G | 7 | -0.04238 | 0.00821 | 2.41E-07 | 0.00179 | 0.00256 | 4.85E-01 |
| entorhinal | temporal | AD | rs3743462 | A | G | 15 | -0.05826 | 0.01116 | 1.78E-07 | 0.00555 | 0.00728 | 4.46E-01 |
| entorhinal | temporal | AD | rs4147321 | C | G | 5 | 0.04761 | 0.00825 | 7.76E-09 | 0.00124 | 0.00262 | 6.36E-01 |
| entorhinal | temporal | AD | rs4888640 | A | T | 16 | 0.03750 | 0.00735 | 3.33E-07 | -0.00369 | 0.00227 | 1.04E-01 |
| entorhinal | temporal | AD | rs6693283 | A | C | 1 | -0.03694 | 0.00694 | 1.01E-07 | 0.00221 | 0.00219 | 3.13E-01 |
| entorhinal | temporal | AD | rs7141150 | A | C | 14 | 0.04487 | 0.00699 | 1.35E-10 | 0.00048 | 0.00217 | 8.26E-01 |
| entorhinal | temporal | AD | rs73205654 | T | C | 8 | -0.06069 | 0.01233 | 8.58E-07 | 0.00168 | 0.00374 | 6.53E-01 |
| fusiform | temporal | AD | rs10784446 | A | G | 12 | -0.02528 | 0.00512 | 7.72E-07 | 0.00361 | 0.00225 | 1.08E-01 |
| fusiform | temporal | AD | rs10940512 | C | G | 5 | 0.03616 | 0.00552 | 5.62E-11 | 0.00115 | 0.00237 | 6.25E-01 |
| fusiform | temporal | AD | rs17834032 | T | C | 14 | 0.03644 | 0.00491 | 1.13E-13 | 0.00045 | 0.00216 | 8.34E-01 |
| fusiform | temporal | AD | rs190064082 | C | G | 18 | -0.12321 | 0.02446 | 4.73E-07 | 0.00651 | 0.01112 | 5.58E-01 |
| fusiform | temporal | AD | rs2074404 | T | G | 17 | 0.03903 | 0.00595 | 5.58E-11 | 0.00947 | 0.00603 | 1.16E-01 |
| fusiform | temporal | AD | rs61784835 | T | C | 1 | 0.02964 | 0.00569 | 1.86E-07 | -0.00401 | 0.00225 | 7.42E-02 |
| fusiform | temporal | AD | rs6801733 | A | G | 3 | -0.05604 | 0.01126 | 6.47E-07 | 0.01161 | 0.00508 | 2.22E-02 |
| fusiform | temporal | AD | rs949279 | A | G | 11 | -0.03082 | 0.00526 | 4.67E-09 | -0.00444 | 0.00230 | 5.40E-02 |
| inferior temporal | temporal | AD | rs12351219 | A | G | 9 | -0.03720 | 0.00725 | 2.86E-07 | 0.00215 | 0.00306 | 4.83E-01 |
| inferior temporal | temporal | AD | rs2252655 | A | G | 10 | 0.02605 | 0.00518 | 4.93E-07 | 0.00435 | 0.00218 | 4.59E-02 |
| inferior temporal | temporal | AD | rs2664129 | T | G | 15 | 0.02968 | 0.00576 | 2.63E-07 | 0.00227 | 0.00248 | 3.61E-01 |
| inferior temporal | temporal | AD | rs4646623 | T | G | 15 | -0.02573 | 0.00503 | 3.08E-07 | 0.00333 | 0.00216 | 1.24E-01 |
| inferior temporal | temporal | AD | rs4895534 | T | C | 6 | 0.02858 | 0.00561 | 3.43E-07 | -0.00168 | 0.00233 | 4.71E-01 |
| inferior temporal | temporal | AD | rs62054449 | T | C | 17 | 0.03381 | 0.00656 | 2.53E-07 | -0.00189 | 0.00263 | 4.73E-01 |
| inferior temporal | temporal | AD | rs7155669 | A | G | 14 | 0.03007 | 0.00549 | 4.39E-08 | -0.00061 | 0.00236 | 7.96E-01 |
| inferior temporal | temporal | AD | rs71575448 | A | G | 5 | 0.04034 | 0.00787 | 2.96E-07 | -0.00016 | 0.00326 | 9.61E-01 |
| inferior temporal | temporal | AD | rs72775393 | T | C | 5 | -0.05132 | 0.01003 | 3.09E-07 | -0.00724 | 0.00413 | 7.94E-02 |
| inferior temporal | temporal | AD | rs73006822 | T | C | 19 | 0.03988 | 0.00754 | 1.22E-07 | 0.00325 | 0.00295 | 2.71E-01 |
| inferior temporal | temporal | AD | rs9309013 | A | G | 2 | -0.03403 | 0.00552 | 6.86E-10 | -0.00945 | 0.01159 | 4.15E-01 |
| insula | temporal | AD | rs10123515 | A | G | 9 | -0.05815 | 0.01184 | 8.96E-07 | -0.00090 | 0.00467 | 8.47E-01 |
| insula | temporal | AD | rs1122688 | T | C | 10 | -0.03783 | 0.00605 | 3.97E-10 | 0.00377 | 0.00248 | 1.29E-01 |
| insula | temporal | AD | rs11231598 | C | G | 11 | 0.06426 | 0.01268 | 4.01E-07 | 0.00350 | 0.00491 | 4.76E-01 |
| insula | temporal | AD | rs11901774 | T | C | 2 | -0.03101 | 0.00570 | 5.29E-08 | -0.00049 | 0.00230 | 8.30E-01 |
| insula | temporal | AD | rs139034643 | C | G | 4 | -0.07305 | 0.01410 | 2.19E-07 | -0.00611 | 0.00637 | 3.37E-01 |
| insula | temporal | AD | rs4291964 | A | G | 17 | -0.03902 | 0.00532 | 2.12E-13 | 0.01067 | 0.00532 | 4.51E-02 |
| insula | temporal | AD | rs4706392 | A | T | 6 | 0.03697 | 0.00682 | 5.81E-08 | -0.00049 | 0.00277 | 8.59E-01 |
| insula | temporal | AD | rs58066679 | A | G | 11 | 0.06298 | 0.00988 | 1.82E-10 | -0.00502 | 0.00394 | 2.02E-01 |
| insula | temporal | AD | rs6739199 | T | C | 2 | 0.02921 | 0.00581 | 4.97E-07 | 0.00380 | 0.00237 | 1.09E-01 |
| insula | temporal | AD | rs73166833 | A | T | 22 | 0.03687 | 0.00722 | 3.27E-07 | -0.00065 | 0.00295 | 8.24E-01 |
| insula | temporal | AD | rs7728751 | A | G | 5 | -0.04054 | 0.00644 | 3.01E-10 | 0.00264 | 0.00265 | 3.18E-01 |
| insula | temporal | AD | rs78986234 | A | T | 18 | -0.02959 | 0.00600 | 8.28E-07 | -0.00297 | 0.00260 | 2.53E-01 |
| insula | temporal | AD | rs80043648 | C | G | 4 | -0.03687 | 0.00736 | 5.44E-07 | 0.00328 | 0.00305 | 2.82E-01 |
| insula | temporal | AD | rs9375452 | T | C | 6 | 0.03334 | 0.00632 | 1.35E-07 | -0.00192 | 0.00255 | 4.51E-01 |
| insula | temporal | AD | rs976423 | A | T | 8 | -0.03281 | 0.00621 | 1.28E-07 | -0.00417 | 0.00258 | 1.06E-01 |
| middle temporal | temporal | AD | rs10045552 | T | C | 5 | -0.02672 | 0.00498 | 8.25E-08 | 0.00100 | 0.00234 | 6.69E-01 |
| middle temporal | temporal | AD | rs10048146 | A | G | 16 | -0.02966 | 0.00602 | 8.20E-07 | 0.00372 | 0.00284 | 1.90E-01 |
| middle temporal | temporal | AD | rs12794347 | A | G | 11 | -0.04976 | 0.00891 | 2.36E-08 | -0.00463 | 0.00397 | 2.43E-01 |
| middle temporal | temporal | AD | rs1344762 | T | C | 2 | -0.02647 | 0.00516 | 2.88E-07 | -0.00166 | 0.00229 | 4.69E-01 |
| middle temporal | temporal | AD | rs141834426 | C | G | 5 | -0.08127 | 0.01225 | 3.31E-11 | 0.00675 | 0.00549 | 2.18E-01 |
| middle temporal | temporal | AD | rs16971055 | A | G | 17 | -0.04585 | 0.00917 | 5.69E-07 | 0.00034 | 0.00380 | 9.30E-01 |
| middle temporal | temporal | AD | rs28711421 | T | C | 9 | -0.04152 | 0.00816 | 3.67E-07 | -0.01232 | 0.00372 | 9.26E-04 |
| middle temporal | temporal | AD | rs621952 | T | C | 2 | -0.02476 | 0.00474 | 1.81E-07 | -0.00081 | 0.00220 | 7.14E-01 |
| middle temporal | temporal | AD | rs62256903 | A | G | 3 | -0.02432 | 0.00475 | 3.14E-07 | -0.00067 | 0.00217 | 7.57E-01 |
| middle temporal | temporal | AD | rs72867280 | A | G | 6 | 0.02924 | 0.00575 | 3.73E-07 | 0.00148 | 0.00260 | 5.69E-01 |
| middle temporal | temporal | AD | rs73006822 | T | C | 19 | 0.04124 | 0.00711 | 6.73E-09 | 0.00325 | 0.00295 | 2.71E-01 |
| middle temporal | temporal | AD | rs7314572 | A | T | 12 | 0.04344 | 0.00860 | 4.42E-07 | 0.00023 | 0.00388 | 9.53E-01 |
| parahippocampal | temporal | AD | rs10117940 | A | G | 9 | 0.03814 | 0.00725 | 1.45E-07 | -0.00187 | 0.00243 | 4.41E-01 |
| parahippocampal | temporal | AD | rs10474080 | A | G | 5 | -0.03366 | 0.00646 | 1.88E-07 | 0.00270 | 0.00220 | 2.20E-01 |
| parahippocampal | temporal | AD | rs11919722 | C | G | 3 | -0.05422 | 0.01016 | 9.41E-08 | -0.00309 | 0.00338 | 3.61E-01 |
| parahippocampal | temporal | AD | rs1792354 | T | C | 11 | -0.04108 | 0.00684 | 1.96E-09 | 0.00040 | 0.00231 | 8.64E-01 |
| parahippocampal | temporal | AD | rs4870489 | C | G | 6 | -0.07063 | 0.01364 | 2.22E-07 | -0.00071 | 0.00472 | 8.80E-01 |
| parahippocampal | temporal | AD | rs5003492 | A | G | 8 | 0.03497 | 0.00646 | 6.05E-08 | 0.00642 | 0.00219 | 3.42E-03 |
| parahippocampal | temporal | AD | rs58131984 | T | G | 7 | 0.03904 | 0.00727 | 7.84E-08 | 0.00134 | 0.00249 | 5.89E-01 |
| parahippocampal | temporal | AD | rs58321169 | T | C | 6 | -0.04625 | 0.00778 | 2.70E-09 | -0.00205 | 0.00588 | 7.27E-01 |
| superior temporal | temporal | AD | rs115241741 | A | C | 15 | -0.07086 | 0.01180 | 1.91E-09 | 0.00301 | 0.00531 | 5.70E-01 |
| superior temporal | temporal | AD | rs143927182 | A | C | 1 | 0.03869 | 0.00787 | 8.88E-07 | -0.00666 | 0.00394 | 9.15E-02 |
| superior temporal | temporal | AD | rs17317075 | A | C | 4 | 0.04188 | 0.00837 | 5.69E-07 | -0.01454 | 0.00826 | 7.86E-02 |
| superior temporal | temporal | AD | rs1785181 | T | C | 18 | 0.02360 | 0.00482 | 9.58E-07 | 0.00136 | 0.00227 | 5.49E-01 |
| superior temporal | temporal | AD | rs2180127 | T | C | 6 | 0.03034 | 0.00584 | 2.08E-07 | -0.00069 | 0.00276 | 8.03E-01 |
| superior temporal | temporal | AD | rs389020 | A | G | 2 | 0.03212 | 0.00475 | 1.40E-11 | 0.00288 | 0.00231 | 2.12E-01 |
| superior temporal | temporal | AD | rs4515470 | C | G | 7 | 0.02649 | 0.00501 | 1.21E-07 | -0.00135 | 0.00242 | 5.77E-01 |
| superior temporal | temporal | AD | rs4690466 | A | C | 4 | -0.02786 | 0.00526 | 1.16E-07 | 0.00096 | 0.00265 | 7.17E-01 |
| superior temporal | temporal | AD | rs4841029 | A | G | 8 | 0.03567 | 0.00461 | 9.59E-15 | -0.00230 | 0.00222 | 3.00E-01 |
| superior temporal | temporal | AD | rs7107246 | A | C | 11 | -0.03861 | 0.00786 | 9.11E-07 | 0.00122 | 0.00371 | 7.42E-01 |
| superior temporal | temporal | AD | rs7601767 | A | G | 2 | 0.02958 | 0.00463 | 1.61E-10 | -0.00549 | 0.00223 | 1.39E-02 |
| superior temporal | temporal | AD | rs7874052 | A | T | 9 | 0.02522 | 0.00475 | 1.11E-07 | 0.00019 | 0.00228 | 9.32E-01 |
| temporal pole | temporal | AD | rs160472 | T | C | 14 | 0.03804 | 0.00744 | 3.20E-07 | -0.00082 | 0.00224 | 7.15E-01 |
| temporal pole | temporal | AD | rs2139446 | A | G | 15 | -0.04127 | 0.00830 | 6.68E-07 | 0.00110 | 0.00254 | 6.65E-01 |
| temporal pole | temporal | AD | rs6855246 | A | G | 4 | 0.08823 | 0.01500 | 4.01E-09 | -0.00841 | 0.00407 | 3.88E-02 |
| temporal pole | temporal | AD | rs73084102 | T | G | 3 | -0.09070 | 0.01760 | 2.54E-07 | 0.00485 | 0.00503 | 3.35E-01 |
| transverse temporal | temporal | AD | rs112315969 | A | T | 6 | -0.04827 | 0.00901 | 8.51E-08 | 0.00200 | 0.00271 | 4.60E-01 |
| transverse temporal | temporal | AD | rs11684511 | A | G | 2 | 0.05210 | 0.00658 | 2.42E-15 | 0.00082 | 0.00223 | 7.12E-01 |
| transverse temporal | temporal | AD | rs11785060 | T | C | 8 | 0.03713 | 0.00685 | 5.92E-08 | 0.00296 | 0.00227 | 1.91E-01 |
| transverse temporal | temporal | AD | rs2033939 | A | G | 15 | -0.06547 | 0.01184 | 3.22E-08 | -0.00881 | 0.00404 | 2.93E-02 |
| transverse temporal | temporal | AD | rs2409691 | T | C | 8 | 0.03591 | 0.00643 | 2.30E-08 | 0.00337 | 0.00217 | 1.20E-01 |
| transverse temporal | temporal | AD | rs2548226 | T | C | 5 | 0.03441 | 0.00657 | 1.60E-07 | -0.00151 | 0.00223 | 4.97E-01 |
| transverse temporal | temporal | AD | rs4706391 | A | T | 6 | -0.05363 | 0.00830 | 1.03E-10 | 0.00053 | 0.00278 | 8.49E-01 |
| transverse temporal | temporal | AD | rs72834200 | A | G | 6 | -0.07719 | 0.01544 | 5.80E-07 | 0.00854 | 0.00485 | 7.83E-02 |
| transverse temporal | temporal | AD | rs7315185 | A | T | 12 | 0.04552 | 0.00919 | 7.29E-07 | 0.00655 | 0.00314 | 3.74E-02 |
| transverse temporal | temporal | AD | rs75061235 | A | G | 9 | 0.06982 | 0.01336 | 1.74E-07 | 0.00920 | 0.00452 | 4.19E-02 |
| transverse temporal | temporal | AD | rs7714191 | C | G | 5 | -0.04154 | 0.00649 | 1.52E-10 | 0.00051 | 0.00222 | 8.19E-01 |
| transverse temporal | temporal | AD | rs949279 | A | G | 11 | 0.03419 | 0.00683 | 5.46E-07 | -0.00444 | 0.00230 | 5.40E-02 |
| transverse temporal | temporal | AD | rs9615351 | C | G | 22 | -0.04456 | 0.00837 | 1.00E-07 | 0.00113 | 0.00252 | 6.53E-01 |
| transverse temporal | temporal | AD | rs9844184 | A | G | 3 | -0.03779 | 0.00742 | 3.49E-07 | 0.00081 | 0.00251 | 7.47E-01 |
| inferior parietal | parietal | AD | rs118054914 | A | G | 10 | 0.09653 | 0.01780 | 5.87E-08 | 0.01364 | 0.00888 | 1.24E-01 |
| inferior parietal | parietal | AD | rs1413536 | T | C | 1 | 0.03572 | 0.00507 | 1.86E-12 | 0.00101 | 0.00217 | 6.41E-01 |
| inferior parietal | parietal | AD | rs148683230 | A | G | 11 | 0.03776 | 0.00724 | 1.79E-07 | -0.00125 | 0.00324 | 6.99E-01 |
| inferior parietal | parietal | AD | rs149940542 | T | C | 5 | 0.14999 | 0.03055 | 9.13E-07 | 0.00875 | 0.01120 | 4.34E-01 |
| inferior parietal | parietal | AD | rs17019370 | A | C | 4 | 0.03000 | 0.00559 | 7.78E-08 | -0.00084 | 0.00239 | 7.27E-01 |
| inferior parietal | parietal | AD | rs2218439 | A | G | 8 | 0.02709 | 0.00506 | 8.61E-08 | 0.00644 | 0.00220 | 3.39E-03 |
| inferior parietal | parietal | AD | rs2336714 | T | C | 12 | -0.04619 | 0.00523 | 1.09E-18 | 0.00391 | 0.00227 | 8.47E-02 |
| inferior parietal | parietal | AD | rs27540 | A | G | 5 | -0.03503 | 0.00509 | 6.14E-12 | 0.00093 | 0.00218 | 6.70E-01 |
| inferior parietal | parietal | AD | rs2779710 | A | G | 9 | -0.02536 | 0.00505 | 5.02E-07 | -0.00024 | 0.00218 | 9.14E-01 |
| inferior parietal | parietal | AD | rs34146683 | C | G | 15 | -0.04531 | 0.00864 | 1.56E-07 | -0.00810 | 0.00397 | 4.12E-02 |
| inferior parietal | parietal | AD | rs4437022 | A | C | 20 | -0.04048 | 0.00510 | 2.06E-15 | 0.00029 | 0.00218 | 8.94E-01 |
| inferior parietal | parietal | AD | rs4889898 | A | C | 17 | -0.02680 | 0.00532 | 4.65E-07 | 0.00176 | 0.00223 | 4.29E-01 |
| inferior parietal | parietal | AD | rs62399042 | C | G | 5 | 0.06337 | 0.00886 | 8.73E-13 | 0.00391 | 0.00385 | 3.10E-01 |
| inferior parietal | parietal | AD | rs639016 | C | G | 2 | 0.04444 | 0.00639 | 3.57E-12 | 0.00227 | 0.00272 | 4.03E-01 |
| inferior parietal | parietal | AD | rs68175985 | A | G | 19 | 0.05797 | 0.00743 | 6.06E-15 | 0.00343 | 0.00287 | 2.32E-01 |
| inferior parietal | parietal | AD | rs75911833 | A | C | 2 | -0.05340 | 0.01076 | 7.00E-07 | 0.00044 | 0.00445 | 9.21E-01 |
| inferior parietal | parietal | AD | rs7862092 | T | G | 9 | 0.04721 | 0.00947 | 6.10E-07 | -0.00287 | 0.00396 | 4.69E-01 |
| inferior parietal | parietal | AD | rs79272390 | T | C | 2 | -0.05135 | 0.00762 | 1.57E-11 | 0.00164 | 0.00326 | 6.15E-01 |
| inferior parietal | parietal | AD | rs9856782 | A | C | 3 | -0.03443 | 0.00600 | 9.82E-09 | -0.00572 | 0.00595 | 3.37E-01 |
| isthmus cingulate | parietal | AD | rs1123680 | A | G | 12 | -0.03617 | 0.00662 | 4.57E-08 | 0.00207 | 0.00258 | 4.22E-01 |
| isthmus cingulate | parietal | AD | rs12616022 | A | T | 2 | 0.03186 | 0.00642 | 7.09E-07 | -0.00236 | 0.00589 | 6.88E-01 |
| isthmus cingulate | parietal | AD | rs258035 | A | G | 5 | -0.03034 | 0.00565 | 7.72E-08 | -0.00166 | 0.00217 | 4.43E-01 |
| isthmus cingulate | parietal | AD | rs3770776 | A | G | 2 | -0.04139 | 0.00579 | 8.98E-13 | -0.00204 | 0.00218 | 3.49E-01 |
| isthmus cingulate | parietal | AD | rs625695 | T | C | 9 | 0.07132 | 0.01421 | 5.24E-07 | 0.01597 | 0.01336 | 2.32E-01 |
| isthmus cingulate | parietal | AD | rs78156452 | T | C | 12 | -0.03382 | 0.00686 | 8.25E-07 | 0.00051 | 0.00266 | 8.47E-01 |
| isthmus cingulate | parietal | AD | rs9392155 | A | G | 6 | 0.04261 | 0.00810 | 1.45E-07 | 0.00333 | 0.00288 | 2.47E-01 |
| postcentral | parietal | AD | rs11033898 | C | G | 11 | 0.02951 | 0.00484 | 1.09E-09 | -0.00054 | 0.00222 | 8.06E-01 |
| postcentral | parietal | AD | rs117623407 | A | G | 19 | 0.03488 | 0.00693 | 4.90E-07 | 0.00405 | 0.00308 | 1.89E-01 |
| postcentral | parietal | AD | rs11789773 | A | C | 9 | -0.03775 | 0.00638 | 3.32E-09 | 0.00087 | 0.00286 | 7.60E-01 |
| postcentral | parietal | AD | rs1884010 | A | G | 14 | -0.02501 | 0.00511 | 9.69E-07 | 0.00578 | 0.00535 | 2.79E-01 |
| postcentral | parietal | AD | rs2279829 | T | C | 3 | -0.06580 | 0.00574 | 1.82E-30 | -0.00537 | 0.00260 | 3.90E-02 |
| postcentral | parietal | AD | rs313135 | T | C | 4 | 0.02826 | 0.00472 | 2.15E-09 | 0.00169 | 0.00217 | 4.36E-01 |
| postcentral | parietal | AD | rs34322452 | A | G | 5 | 0.04350 | 0.00569 | 2.09E-14 | -0.00073 | 0.00255 | 7.75E-01 |
| postcentral | parietal | AD | rs344142 | A | G | 4 | -0.02384 | 0.00487 | 9.84E-07 | 0.00090 | 0.00221 | 6.85E-01 |
| postcentral | parietal | AD | rs4924346 | A | G | 15 | 0.13265 | 0.00842 | 5.83E-56 | -0.00898 | 0.00380 | 1.81E-02 |
| postcentral | parietal | AD | rs555720 | T | C | 6 | 0.03038 | 0.00588 | 2.44E-07 | -0.00193 | 0.00271 | 4.77E-01 |
| postcentral | parietal | AD | rs61842496 | T | C | 10 | -0.05444 | 0.01075 | 4.13E-07 | 0.00143 | 0.00402 | 7.22E-01 |
| postcentral | parietal | AD | rs7856501 | T | C | 9 | 0.02366 | 0.00471 | 5.00E-07 | -0.00013 | 0.00216 | 9.51E-01 |
| postcentral | parietal | AD | rs881743 | A | G | 4 | 0.02754 | 0.00515 | 8.72E-08 | -0.00195 | 0.00236 | 4.10E-01 |
| posterior cingulate | parietal | AD | rs11161942 | T | C | 1 | 0.05156 | 0.00830 | 5.29E-10 | -0.00112 | 0.00326 | 7.31E-01 |
| posterior cingulate | parietal | AD | rs11695609 | T | C | 2 | -0.03545 | 0.00553 | 1.47E-10 | -0.00231 | 0.00216 | 2.85E-01 |
| posterior cingulate | parietal | AD | rs12764880 | T | C | 10 | -0.03205 | 0.00558 | 9.38E-09 | -0.00066 | 0.00217 | 7.61E-01 |
| posterior cingulate | parietal | AD | rs2412771 | T | C | 4 | 0.02917 | 0.00565 | 2.41E-07 | -0.00502 | 0.00223 | 2.42E-02 |
| posterior cingulate | parietal | AD | rs66651545 | A | C | 8 | 0.03737 | 0.00716 | 1.80E-07 | 0.02686 | 0.01376 | 5.10E-02 |
| posterior cingulate | parietal | AD | rs72761270 | T | C | 5 | 0.03676 | 0.00580 | 2.32E-10 | 0.00417 | 0.00230 | 6.99E-02 |
| posterior cingulate | parietal | AD | rs7315284 | T | C | 12 | 0.03378 | 0.00687 | 8.65E-07 | 0.00004 | 0.00243 | 9.87E-01 |
| posterior cingulate | parietal | AD | rs77935092 | T | C | 14 | 0.09218 | 0.01744 | 1.26E-07 | -0.00207 | 0.00647 | 7.49E-01 |
| posterior cingulate | parietal | AD | rs841860 | T | G | 1 | 0.04725 | 0.00935 | 4.38E-07 | -0.01130 | 0.00743 | 1.28E-01 |
| precuneus | parietal | AD | rs10008615 | T | C | 4 | -0.02615 | 0.00530 | 7.89E-07 | 0.00314 | 0.00252 | 2.14E-01 |
| precuneus | parietal | AD | rs10749233 | C | G | 10 | -0.03354 | 0.00557 | 1.74E-09 | 0.00267 | 0.00249 | 2.84E-01 |
| precuneus | parietal | AD | rs12639074 | T | C | 3 | 0.02304 | 0.00467 | 8.21E-07 | -0.00143 | 0.00218 | 5.12E-01 |
| precuneus | parietal | AD | rs13060816 | T | C | 3 | 0.02313 | 0.00460 | 5.01E-07 | -0.00205 | 0.00216 | 3.44E-01 |
| precuneus | parietal | AD | rs13166639 | T | C | 5 | 0.02909 | 0.00562 | 2.31E-07 | 0.00339 | 0.00551 | 5.39E-01 |
| precuneus | parietal | AD | rs1451294 | A | T | 18 | -0.02700 | 0.00536 | 4.70E-07 | -0.00218 | 0.00272 | 4.23E-01 |
| precuneus | parietal | AD | rs2022932 | A | G | 8 | 0.02928 | 0.00561 | 1.82E-07 | -0.00157 | 0.00267 | 5.58E-01 |
| precuneus | parietal | AD | rs2432803 | T | C | 6 | -0.03142 | 0.00620 | 4.05E-07 | -0.00269 | 0.00285 | 3.45E-01 |
| precuneus | parietal | AD | rs4774220 | A | G | 15 | 0.02620 | 0.00530 | 7.63E-07 | 0.00144 | 0.00246 | 5.58E-01 |
| precuneus | parietal | AD | rs4811601 | T | C | 20 | -0.02550 | 0.00469 | 5.50E-08 | -0.00091 | 0.00220 | 6.78E-01 |
| precuneus | parietal | AD | rs56252769 | T | C | 11 | -0.02637 | 0.00532 | 7.30E-07 | 0.00559 | 0.00249 | 2.48E-02 |
| precuneus | parietal | AD | rs59373415 | C | G | 1 | 0.03970 | 0.00621 | 1.60E-10 | -0.00761 | 0.00294 | 9.65E-03 |
| precuneus | parietal | AD | rs7111565 | T | C | 11 | 0.02750 | 0.00528 | 1.93E-07 | -0.00013 | 0.00246 | 9.56E-01 |
| precuneus | parietal | AD | rs73313052 | A | G | 14 | 0.07105 | 0.00693 | 1.13E-24 | 0.00289 | 0.00328 | 3.79E-01 |
| precuneus | parietal | AD | rs7559976 | T | C | 2 | -0.03069 | 0.00481 | 1.70E-10 | -0.00031 | 0.00220 | 8.88E-01 |
| precuneus | parietal | AD | rs775718 | A | G | 3 | -0.02277 | 0.00465 | 9.75E-07 | 0.00205 | 0.00223 | 3.58E-01 |
| precuneus | parietal | AD | rs7782319 | A | G | 7 | 0.02411 | 0.00481 | 5.40E-07 | -0.00159 | 0.00535 | 7.66E-01 |
| precuneus | parietal | AD | rs888814 | T | G | 5 | 0.02655 | 0.00464 | 1.02E-08 | -0.00298 | 0.00229 | 1.94E-01 |
| precuneus | parietal | AD | rs905124 | A | T | 3 | 0.03246 | 0.00498 | 6.86E-11 | 0.00403 | 0.00225 | 7.38E-02 |
| precuneus | parietal | AD | rs9399245 | T | G | 6 | -0.03217 | 0.00508 | 2.46E-10 | 0.00168 | 0.00229 | 4.64E-01 |
| superior parietal | parietal | AD | rs10109434 | A | C | 8 | 0.03037 | 0.00562 | 6.48E-08 | -0.00060 | 0.00225 | 7.91E-01 |
| superior parietal | parietal | AD | rs114489117 | A | T | 5 | -0.05750 | 0.00859 | 2.11E-11 | 0.00022 | 0.00376 | 9.54E-01 |
| superior parietal | parietal | AD | rs115877304 | T | C | 5 | 0.09979 | 0.01575 | 2.37E-10 | 0.00521 | 0.00554 | 3.47E-01 |
| superior parietal | parietal | AD | rs142050688 | T | C | 6 | -0.10228 | 0.01952 | 1.61E-07 | -0.00157 | 0.00799 | 8.45E-01 |
| superior parietal | parietal | AD | rs17718831 | A | G | 3 | -0.02963 | 0.00533 | 2.68E-08 | -0.00019 | 0.00225 | 9.31E-01 |
| superior parietal | parietal | AD | rs1884368 | T | C | 1 | 0.03326 | 0.00635 | 1.61E-07 | -0.00134 | 0.00626 | 8.31E-01 |
| superior parietal | parietal | AD | rs2144366 | C | G | 6 | -0.04292 | 0.00752 | 1.17E-08 | 0.00161 | 0.00337 | 6.32E-01 |
| superior parietal | parietal | AD | rs40084 | T | C | 5 | -0.02626 | 0.00527 | 6.25E-07 | -0.00302 | 0.00223 | 1.77E-01 |
| superior parietal | parietal | AD | rs4924345 | A | C | 15 | 0.10130 | 0.00945 | 8.60E-27 | 0.00835 | 0.00401 | 3.74E-02 |
| superior parietal | parietal | AD | rs6022786 | A | G | 20 | 0.03076 | 0.00529 | 6.30E-09 | 0.00138 | 0.00219 | 5.29E-01 |
| superior parietal | parietal | AD | rs6059516 | T | C | 20 | 0.02967 | 0.00571 | 2.08E-07 | -0.00174 | 0.00239 | 4.67E-01 |
| superior parietal | parietal | AD | rs61872090 | A | G | 10 | -0.02836 | 0.00530 | 8.96E-08 | 0.00259 | 0.00221 | 2.40E-01 |
| superior parietal | parietal | AD | rs639016 | C | G | 2 | -0.05325 | 0.00649 | 2.35E-16 | 0.00227 | 0.00272 | 4.03E-01 |
| superior parietal | parietal | AD | rs6554054 | A | G | 4 | -0.04473 | 0.00729 | 8.42E-10 | 0.00280 | 0.00308 | 3.62E-01 |
| superior parietal | parietal | AD | rs68175985 | A | G | 19 | -0.07161 | 0.00754 | 2.23E-21 | 0.00343 | 0.00287 | 2.32E-01 |
| superior parietal | parietal | AD | rs6840242 | T | C | 4 | -0.03127 | 0.00519 | 1.72E-09 | -0.00078 | 0.00221 | 7.23E-01 |
| superior parietal | parietal | AD | rs79272390 | T | C | 2 | 0.04604 | 0.00777 | 3.14E-09 | 0.00164 | 0.00326 | 6.15E-01 |
| superior parietal | parietal | AD | rs7980991 | A | C | 12 | -0.03627 | 0.00635 | 1.13E-08 | 0.00055 | 0.00253 | 8.27E-01 |
| supramarginal | parietal | AD | rs1398859 | T | G | 1 | -0.02990 | 0.00563 | 1.06E-07 | 0.00112 | 0.00237 | 6.35E-01 |
| supramarginal | parietal | AD | rs17011924 | A | G | 4 | 0.02692 | 0.00523 | 2.63E-07 | 0.00138 | 0.00228 | 5.45E-01 |
| supramarginal | parietal | AD | rs2164950 | A | G | 14 | 0.06121 | 0.00743 | 1.67E-16 | 0.00389 | 0.00330 | 2.39E-01 |
| supramarginal | parietal | AD | rs2200225 | A | G | 4 | 0.04075 | 0.00668 | 1.04E-09 | -0.00314 | 0.00292 | 2.82E-01 |
| supramarginal | parietal | AD | rs2279829 | T | C | 3 | -0.05177 | 0.00599 | 5.52E-18 | -0.00537 | 0.00260 | 3.90E-02 |
| supramarginal | parietal | AD | rs28395235 | A | T | 15 | 0.03059 | 0.00546 | 2.07E-08 | 0.00475 | 0.00232 | 4.05E-02 |
| supramarginal | parietal | AD | rs28421555 | T | C | 2 | -0.03199 | 0.00651 | 8.99E-07 | 0.00166 | 0.00286 | 5.63E-01 |
| supramarginal | parietal | AD | rs35378400 | A | G | 8 | -0.03165 | 0.00632 | 5.51E-07 | -0.00273 | 0.00249 | 2.74E-01 |
| supramarginal | parietal | AD | rs4924345 | A | C | 15 | 0.12341 | 0.00911 | 7.56E-42 | 0.00835 | 0.00401 | 3.74E-02 |
| supramarginal | parietal | AD | rs61407096 | A | G | 4 | 0.04551 | 0.00882 | 2.51E-07 | -0.00024 | 0.00300 | 9.37E-01 |
| supramarginal | parietal | AD | rs724972 | A | T | 3 | -0.02577 | 0.00501 | 2.73E-07 | -0.00012 | 0.00222 | 9.57E-01 |
| cuneus | occipital | AD | rs10765918 | A | G | 11 | 0.04726 | 0.00745 | 2.29E-10 | 0.00153 | 0.00247 | 5.36E-01 |
| cuneus | occipital | AD | rs112484789 | T | C | 14 | 0.05648 | 0.01078 | 1.62E-07 | -0.00225 | 0.00337 | 5.04E-01 |
| cuneus | occipital | AD | rs115136616 | T | C | 5 | 0.07750 | 0.01468 | 1.31E-07 | 0.01093 | 0.00520 | 3.55E-02 |
| cuneus | occipital | AD | rs11652557 | A | G | 17 | -0.07429 | 0.01182 | 3.25E-10 | 0.00150 | 0.00416 | 7.19E-01 |
| cuneus | occipital | AD | rs12536836 | T | C | 7 | 0.03772 | 0.00622 | 1.33E-09 | 0.00232 | 0.00220 | 2.92E-01 |
| cuneus | occipital | AD | rs16829649 | A | G | 3 | 0.05023 | 0.00953 | 1.35E-07 | -0.00618 | 0.00349 | 7.64E-02 |
| cuneus | occipital | AD | rs2155645 | T | C | 11 | -0.03407 | 0.00681 | 5.73E-07 | -0.00179 | 0.00246 | 4.67E-01 |
| cuneus | occipital | AD | rs4895120 | T | C | 3 | -0.04195 | 0.00607 | 4.67E-12 | 0.00170 | 0.00216 | 4.31E-01 |
| cuneus | occipital | AD | rs71427711 | A | G | 2 | -0.04804 | 0.00868 | 3.09E-08 | -0.00393 | 0.00312 | 2.08E-01 |
| cuneus | occipital | AD | rs73092904 | T | C | 1 | -0.03955 | 0.00775 | 3.34E-07 | 0.00094 | 0.00273 | 7.30E-01 |
| cuneus | occipital | AD | rs73313052 | A | G | 14 | -0.07472 | 0.00906 | 1.62E-16 | 0.00289 | 0.00328 | 3.79E-01 |
| cuneus | occipital | AD | rs76470478 | T | C | 6 | -0.10028 | 0.01701 | 3.73E-09 | 0.00092 | 0.00591 | 8.76E-01 |
| cuneus | occipital | AD | rs9834227 | A | G | 3 | -0.03553 | 0.00655 | 5.81E-08 | -0.00200 | 0.00230 | 3.85E-01 |
| lateral occipital | occipital | AD | rs10438427 | A | G | 15 | -0.03784 | 0.00746 | 3.90E-07 | 0.00567 | 0.00721 | 4.32E-01 |
| lateral occipital | occipital | AD | rs12485668 | T | C | 3 | 0.07530 | 0.01511 | 6.24E-07 | 0.01252 | 0.00459 | 6.37E-03 |
| lateral occipital | occipital | AD | rs12609819 | A | G | 19 | 0.02634 | 0.00515 | 3.06E-07 | -0.00181 | 0.00217 | 4.04E-01 |
| lateral occipital | occipital | AD | rs16915479 | T | C | 12 | -0.04612 | 0.00903 | 3.24E-07 | 0.00297 | 0.00379 | 4.33E-01 |
| lateral occipital | occipital | AD | rs1781030 | T | C | 1 | 0.02553 | 0.00511 | 5.73E-07 | -0.00004 | 0.00218 | 9.84E-01 |
| lateral occipital | occipital | AD | rs2074404 | T | G | 17 | 0.03199 | 0.00612 | 1.72E-07 | 0.00947 | 0.00603 | 1.16E-01 |
| lateral occipital | occipital | AD | rs2282710 | T | C | 1 | 0.02673 | 0.00507 | 1.38E-07 | -0.00189 | 0.00217 | 3.84E-01 |
| lateral occipital | occipital | AD | rs28496034 | C | G | 9 | 0.04246 | 0.00545 | 6.28E-15 | -0.00307 | 0.00230 | 1.83E-01 |
| lateral occipital | occipital | AD | rs28514429 | A | C | 15 | -0.02997 | 0.00547 | 4.18E-08 | -0.00172 | 0.00529 | 7.46E-01 |
| lateral occipital | occipital | AD | rs4953152 | A | G | 2 | 0.02965 | 0.00544 | 5.03E-08 | 0.00051 | 0.00228 | 8.24E-01 |
| lateral occipital | occipital | AD | rs552305 | T | C | 3 | 0.03609 | 0.00524 | 5.78E-12 | 0.00098 | 0.00538 | 8.55E-01 |
| lateral occipital | occipital | AD | rs56007616 | A | G | 3 | 0.04493 | 0.00788 | 1.17E-08 | -0.00523 | 0.00330 | 1.14E-01 |
| lateral occipital | occipital | AD | rs76341705 | A | G | 14 | -0.06981 | 0.00762 | 5.34E-20 | 0.00375 | 0.00351 | 2.85E-01 |
| lateral occipital | occipital | AD | rs8076087 | A | G | 17 | -0.02767 | 0.00552 | 5.41E-07 | -0.00046 | 0.00229 | 8.40E-01 |
| lateral occipital | occipital | AD | rs9401907 | T | C | 6 | 0.03711 | 0.00593 | 3.86E-10 | 0.00230 | 0.00249 | 3.57E-01 |
| lateral occipital | occipital | AD | rs9863836 | T | C | 3 | -0.03812 | 0.00614 | 5.29E-10 | -0.00098 | 0.00271 | 7.17E-01 |
| lingual | occipital | AD | rs1014444 | A | G | 2 | 0.03569 | 0.00643 | 2.79E-08 | 0.00064 | 0.00227 | 7.80E-01 |
| lingual | occipital | AD | rs10237280 | T | C | 7 | 0.04258 | 0.00644 | 3.79E-11 | 0.00134 | 0.00223 | 5.47E-01 |
| lingual | occipital | AD | rs1078081 | T | C | 9 | 0.04419 | 0.00815 | 6.02E-08 | -0.00043 | 0.00287 | 8.80E-01 |
| lingual | occipital | AD | rs11929686 | A | G | 3 | -0.03919 | 0.00620 | 2.54E-10 | -0.00041 | 0.00218 | 8.51E-01 |
| lingual | occipital | AD | rs17419290 | A | G | 5 | 0.03545 | 0.00628 | 1.65E-08 | 0.00423 | 0.00218 | 5.24E-02 |
| lingual | occipital | AD | rs17690987 | T | C | 17 | 0.04890 | 0.00935 | 1.68E-07 | 0.00516 | 0.00267 | 5.34E-02 |
| lingual | occipital | AD | rs1905346 | T | C | 3 | 0.03199 | 0.00642 | 6.24E-07 | 0.00062 | 0.00225 | 7.83E-01 |
| lingual | occipital | AD | rs1934057 | T | C | 1 | -0.03891 | 0.00635 | 8.98E-10 | 0.00242 | 0.00220 | 2.70E-01 |
| lingual | occipital | AD | rs1952750 | T | C | 14 | -0.03730 | 0.00709 | 1.41E-07 | 0.00092 | 0.00564 | 8.71E-01 |
| lingual | occipital | AD | rs2022130 | T | C | 11 | 0.04500 | 0.00683 | 4.45E-11 | 0.00029 | 0.00237 | 9.01E-01 |
| lingual | occipital | AD | rs2043294 | A | T | 19 | 0.04269 | 0.00844 | 4.18E-07 | 0.00405 | 0.00284 | 1.54E-01 |
| lingual | occipital | AD | rs28410513 | T | G | 9 | -0.04266 | 0.00745 | 1.03E-08 | -0.00308 | 0.00261 | 2.37E-01 |
| lingual | occipital | AD | rs2999158 | T | C | 1 | -0.04430 | 0.00660 | 1.91E-11 | -0.00444 | 0.00232 | 5.54E-02 |
| lingual | occipital | AD | rs61508189 | A | C | 3 | 0.03899 | 0.00648 | 1.77E-09 | 0.00004 | 0.00528 | 9.94E-01 |
| lingual | occipital | AD | rs6603991 | T | C | 1 | 0.04436 | 0.00771 | 8.86E-09 | 0.00459 | 0.00271 | 9.06E-02 |
| lingual | occipital | AD | rs6812278 | C | G | 4 | 0.04218 | 0.00693 | 1.14E-09 | 0.00170 | 0.00236 | 4.72E-01 |
| lingual | occipital | AD | rs76341705 | A | G | 14 | -0.08213 | 0.00931 | 1.10E-18 | 0.00375 | 0.00351 | 2.85E-01 |
| lingual | occipital | AD | rs7809950 | T | C | 7 | 0.04146 | 0.00690 | 1.82E-09 | -0.00162 | 0.00239 | 4.98E-01 |
| lingual | occipital | AD | rs7914158 | T | C | 10 | 0.03519 | 0.00642 | 4.30E-08 | 0.00279 | 0.00222 | 2.09E-01 |
| lingual | occipital | AD | rs9401907 | T | C | 6 | 0.04699 | 0.00724 | 8.74E-11 | 0.00230 | 0.00249 | 3.57E-01 |
| lingual | occipital | AD | rs9545145 | A | G | 13 | -0.03466 | 0.00615 | 1.78E-08 | 0.00187 | 0.00216 | 3.86E-01 |
| pericalcarine | occipital | AD | rs1046953 | T | C | 3 | -0.03930 | 0.00734 | 8.77E-08 | -0.00711 | 0.00220 | 1.21E-03 |
| pericalcarine | occipital | AD | rs10765918 | A | G | 11 | 0.05717 | 0.00863 | 3.48E-11 | 0.00153 | 0.00247 | 5.36E-01 |
| pericalcarine | occipital | AD | rs11103221 | T | C | 9 | 0.04349 | 0.00806 | 6.84E-08 | 0.00008 | 0.00570 | 9.89E-01 |
| pericalcarine | occipital | AD | rs11252615 | T | C | 10 | 0.03464 | 0.00705 | 8.92E-07 | -0.00067 | 0.00217 | 7.57E-01 |
| pericalcarine | occipital | AD | rs117892760 | T | C | 6 | -0.15519 | 0.02085 | 9.85E-14 | -0.00059 | 0.00645 | 9.27E-01 |
| pericalcarine | occipital | AD | rs1223090 | A | G | 11 | 0.04957 | 0.00698 | 1.21E-12 | 0.00141 | 0.00218 | 5.17E-01 |
| pericalcarine | occipital | AD | rs13115025 | A | T | 4 | 0.10579 | 0.01800 | 4.20E-09 | 0.00199 | 0.00475 | 6.75E-01 |
| pericalcarine | occipital | AD | rs13726 | T | C | 17 | 0.03924 | 0.00784 | 5.57E-07 | 0.00278 | 0.00238 | 2.44E-01 |
| pericalcarine | occipital | AD | rs1420791 | A | G | 17 | 0.08017 | 0.01360 | 3.74E-09 | -0.00102 | 0.00416 | 8.06E-01 |
| pericalcarine | occipital | AD | rs147753572 | A | C | 1 | 0.13968 | 0.02551 | 4.35E-08 | -0.00103 | 0.00803 | 8.98E-01 |
| pericalcarine | occipital | AD | rs163499 | T | C | 2 | 0.03789 | 0.00730 | 2.08E-07 | -0.00102 | 0.00220 | 6.44E-01 |
| pericalcarine | occipital | AD | rs16822665 | T | C | 2 | -0.04091 | 0.00738 | 2.91E-08 | -0.00030 | 0.00231 | 8.98E-01 |
| pericalcarine | occipital | AD | rs16829649 | A | G | 3 | 0.06375 | 0.01102 | 7.20E-09 | -0.00618 | 0.00349 | 7.64E-02 |
| pericalcarine | occipital | AD | rs17179798 | A | G | 12 | 0.04814 | 0.00846 | 1.30E-08 | 0.00102 | 0.00266 | 7.02E-01 |
| pericalcarine | occipital | AD | rs28633576 | T | C | 9 | -0.04718 | 0.00857 | 3.63E-08 | -0.00328 | 0.00266 | 2.17E-01 |
| pericalcarine | occipital | AD | rs2999158 | T | C | 1 | -0.05315 | 0.00743 | 8.31E-13 | -0.00444 | 0.00232 | 5.54E-02 |
| pericalcarine | occipital | AD | rs35342371 | A | T | 14 | -0.04677 | 0.00790 | 3.20E-09 | 0.00309 | 0.00242 | 2.01E-01 |
| pericalcarine | occipital | AD | rs4811476 | T | C | 20 | 0.04665 | 0.00773 | 1.59E-09 | 0.00013 | 0.00216 | 9.51E-01 |
| pericalcarine | occipital | AD | rs4895532 | T | C | 6 | -0.04317 | 0.00728 | 3.07E-09 | 0.00055 | 0.00221 | 8.05E-01 |
| pericalcarine | occipital | AD | rs56111638 | T | C | 5 | -0.03801 | 0.00754 | 4.67E-07 | -0.00174 | 0.00226 | 4.40E-01 |
| pericalcarine | occipital | AD | rs57063427 | T | C | 5 | -0.06586 | 0.01190 | 3.11E-08 | -0.01034 | 0.00376 | 5.99E-03 |
| pericalcarine | occipital | AD | rs57334908 | T | C | 11 | 0.08402 | 0.01670 | 4.85E-07 | -0.00651 | 0.00425 | 1.26E-01 |
| pericalcarine | occipital | AD | rs62367903 | A | G | 5 | 0.05993 | 0.00704 | 1.66E-17 | 0.00455 | 0.00218 | 3.68E-02 |
| pericalcarine | occipital | AD | rs6461386 | A | G | 7 | 0.05062 | 0.00739 | 7.28E-12 | 0.00231 | 0.00226 | 3.06E-01 |
| pericalcarine | occipital | AD | rs6650695 | A | G | 18 | -0.04009 | 0.00819 | 9.93E-07 | 0.00105 | 0.00225 | 6.39E-01 |
| pericalcarine | occipital | AD | rs667801 | T | C | 11 | 0.03636 | 0.00706 | 2.61E-07 | -0.00360 | 0.00220 | 1.02E-01 |
| pericalcarine | occipital | AD | rs6741950 | C | G | 2 | 0.03679 | 0.00727 | 4.16E-07 | -0.00073 | 0.00226 | 7.47E-01 |
| pericalcarine | occipital | AD | rs6812278 | C | G | 4 | 0.05659 | 0.00780 | 4.08E-13 | 0.00170 | 0.00236 | 4.72E-01 |
| pericalcarine | occipital | AD | rs7188071 | T | C | 16 | 0.04067 | 0.00752 | 6.34E-08 | -0.00270 | 0.00227 | 2.35E-01 |
| pericalcarine | occipital | AD | rs73313052 | A | G | 14 | -0.10946 | 0.01047 | 1.46E-25 | 0.00289 | 0.00328 | 3.79E-01 |
| pericalcarine | occipital | AD | rs7364475 | T | C | 1 | -0.03858 | 0.00731 | 1.33E-07 | -0.00166 | 0.00240 | 4.90E-01 |
| pericalcarine | occipital | AD | rs7378179 | A | T | 4 | -0.04401 | 0.00776 | 1.39E-08 | -0.00152 | 0.00239 | 5.25E-01 |
| pericalcarine | occipital | AD | rs8034885 | T | C | 15 | 0.05998 | 0.01026 | 5.11E-09 | -0.00151 | 0.00295 | 6.09E-01 |
| pericalcarine | occipital | AD | rs8103241 | A | G | 19 | -0.03556 | 0.00713 | 6.06E-07 | 0.00211 | 0.00216 | 3.30E-01 |
| pericalcarine | occipital | AD | rs816328 | T | C | 3 | 0.04052 | 0.00815 | 6.57E-07 | 0.00554 | 0.00258 | 3.20E-02 |
| pericalcarine | occipital | AD | rs9545158 | A | G | 13 | 0.05149 | 0.00695 | 1.26E-13 | -0.00269 | 0.00216 | 2.15E-01 |
| pericalcarine | occipital | AD | rs961848 | A | C | 5 | -0.04043 | 0.00821 | 8.53E-07 | 0.00326 | 0.00256 | 2.03E-01 |
| pericalcarine | occipital | AD | rs971550 | A | T | 3 | 0.04589 | 0.00750 | 9.47E-10 | 0.00095 | 0.00235 | 6.85E-01 |
| whole cortex | global | AD | rs10496091 | A | G | 2 | -0.03894 | 0.00733 | 1.10E-07 | -0.00062 | 0.00241 | 7.98E-01 |
| whole cortex | global | AD | rs10876864 | A | G | 12 | -0.03809 | 0.00683 | 2.43E-08 | 0.00069 | 0.00219 | 7.52E-01 |
| whole cortex | global | AD | rs10878349 | A | G | 12 | 0.06302 | 0.00670 | 4.83E-21 | -0.00533 | 0.00217 | 1.39E-02 |
| whole cortex | global | AD | rs10927043 | T | C | 1 | 0.04358 | 0.00856 | 3.58E-07 | -0.00047 | 0.00273 | 8.64E-01 |
| whole cortex | global | AD | rs11759026 | A | G | 6 | -0.07887 | 0.00816 | 4.11E-22 | -0.00502 | 0.00252 | 4.67E-02 |
| whole cortex | global | AD | rs12357321 | A | G | 10 | -0.04234 | 0.00725 | 5.22E-09 | -0.00002 | 0.00237 | 9.93E-01 |
| whole cortex | global | AD | rs12452834 | T | C | 17 | -0.03796 | 0.00749 | 4.01E-07 | 0.00217 | 0.00551 | 6.94E-01 |
| whole cortex | global | AD | rs12630663 | T | C | 3 | -0.03835 | 0.00674 | 1.27E-08 | 0.00110 | 0.00221 | 6.18E-01 |
| whole cortex | global | AD | rs139849708 | T | C | 6 | 0.13561 | 0.02558 | 1.15E-07 | 0.00026 | 0.00704 | 9.70E-01 |
| whole cortex | global | AD | rs149352678 | T | C | 7 | 0.05864 | 0.01161 | 4.36E-07 | -0.01255 | 0.00370 | 7.02E-04 |
| whole cortex | global | AD | rs1628768 | T | C | 10 | -0.05896 | 0.00800 | 1.70E-13 | -0.00288 | 0.00251 | 2.51E-01 |
| whole cortex | global | AD | rs17543864 | A | G | 11 | -0.03781 | 0.00709 | 9.65E-08 | 0.00107 | 0.00238 | 6.54E-01 |
| whole cortex | global | AD | rs2066827 | T | G | 12 | 0.05226 | 0.00969 | 6.99E-08 | -0.00163 | 0.00252 | 5.16E-01 |
| whole cortex | global | AD | rs2195243 | C | G | 12 | -0.04662 | 0.00862 | 6.38E-08 | -0.00094 | 0.00266 | 7.24E-01 |
| whole cortex | global | AD | rs2301718 | A | G | 4 | 0.04468 | 0.00802 | 2.55E-08 | 0.00378 | 0.00256 | 1.40E-01 |
| whole cortex | global | AD | rs2802295 | A | G | 6 | -0.04330 | 0.00685 | 2.54E-10 | -0.00626 | 0.00220 | 4.49E-03 |
| whole cortex | global | AD | rs3217901 | A | G | 12 | -0.03597 | 0.00695 | 2.29E-07 | 0.00931 | 0.00537 | 8.29E-02 |
| whole cortex | global | AD | rs34464850 | C | G | 3 | 0.07473 | 0.00925 | 6.76E-16 | -0.00179 | 0.00302 | 5.54E-01 |
| whole cortex | global | AD | rs35227403 | A | T | 12 | -0.07707 | 0.01537 | 5.33E-07 | -0.00953 | 0.00470 | 4.25E-02 |
| whole cortex | global | AD | rs386424 | T | G | 5 | -0.03979 | 0.00727 | 4.52E-08 | -0.00370 | 0.00237 | 1.19E-01 |
| whole cortex | global | AD | rs41563 | A | G | 7 | 0.03555 | 0.00705 | 4.63E-07 | 0.00100 | 0.00228 | 6.63E-01 |
| whole cortex | global | AD | rs4846200 | T | C | 1 | 0.03810 | 0.00757 | 4.88E-07 | 0.00249 | 0.00533 | 6.40E-01 |
| whole cortex | global | AD | rs57415181 | C | G | 2 | -0.05058 | 0.00966 | 1.64E-07 | -0.00543 | 0.00302 | 7.25E-02 |
| whole cortex | global | AD | rs6463758 | A | G | 7 | -0.03400 | 0.00681 | 5.98E-07 | 0.00602 | 0.00218 | 5.79E-03 |
| whole cortex | global | AD | rs6572878 | T | C | 14 | 0.03484 | 0.00691 | 4.52E-07 | -0.00241 | 0.00539 | 6.55E-01 |
| whole cortex | global | AD | rs6673449 | A | G | 1 | 0.03351 | 0.00670 | 5.67E-07 | -0.00261 | 0.00221 | 2.37E-01 |
| whole cortex | global | AD | rs7715167 | T | C | 5 | -0.04016 | 0.00722 | 2.65E-08 | -0.00040 | 0.00221 | 8.57E-01 |
| whole cortex | global | AD | rs79600142 | T | C | 17 | 0.10283 | 0.00868 | 2.33E-32 | 0.00417 | 0.00267 | 1.18E-01 |
| whole cortex | global | AD | rs7975351 | A | G | 12 | 0.03703 | 0.00688 | 7.40E-08 | 0.00155 | 0.00218 | 4.78E-01 |

**Table S3.** Summary of genetic variants used to estimate the effect of cortical thickness on Alzheimer's disease (AD) risk

| **Exposure** | **lobe** | **outcome** | **SNP** | **effect allele** | **other allele** | **chr** | **beta exposure** | **se exposure** | **pval exposure** | **beta outcome** | **se outcome** | **pval outcome** |
| --- | --- | --- | --- | --- | --- | --- | --- | --- | --- | --- | --- | --- |
| caudal anterior cingulate | frontal | AD | rs111635253 | A | G | 15 | -0.09286 | 0.01810 | 2.10E-07 | -0.00130 | 0.00493 | 7.92E-01 |
| caudal anterior cingulate | frontal | AD | rs11778360 | T | C | 8 | -0.03810 | 0.00762 | 2.65E-07 | -0.00225 | 0.00217 | 2.98E-01 |
| caudal anterior cingulate | frontal | AD | rs73528979 | A | G | 6 | 0.05333 | 0.01048 | 2.30E-07 | 0.00036 | 0.00309 | 9.07E-01 |
| caudal anterior cingulate | frontal | AD | rs7737603 | T | C | 5 | 0.05476 | 0.01095 | 8.90E-07 | 0.00351 | 0.00326 | 2.83E-01 |
| caudal anterior cingulate | frontal | AD | rs9929528 | T | G | 16 | 0.05714 | 0.01048 | 7.67E-08 | -0.00888 | 0.00531 | 9.43E-02 |
| caudal middle frontal | frontal | AD | rs11748651 | A | T | 5 | 0.03313 | 0.00500 | 1.07E-11 | 0.00232 | 0.00236 | 3.27E-01 |
| caudal middle frontal | frontal | AD | rs13135092 | A | G | 4 | -0.04875 | 0.00875 | 4.80E-08 | -0.00750 | 0.00417 | 7.22E-02 |
| caudal middle frontal | frontal | AD | rs73004648 | A | G | 3 | 0.03500 | 0.00688 | 6.11E-07 | 0.00333 | 0.00314 | 2.90E-01 |
| caudal middle frontal | frontal | AD | rs73006822 | T | C | 19 | -0.03500 | 0.00688 | 8.18E-07 | 0.00325 | 0.00295 | 2.71E-01 |
| caudal middle frontal | frontal | AD | rs74700892 | T | C | 2 | -0.13063 | 0.02625 | 8.23E-07 | -0.00608 | 0.01037 | 5.58E-01 |
| frontal pole | frontal | AD | rs12463979 | C | G | 2 | 0.03231 | 0.00654 | 8.74E-07 | -0.00008 | 0.00218 | 9.72E-01 |
| frontal pole | frontal | AD | rs56023709 | A | C | 16 | 0.05346 | 0.00654 | 9.65E-16 | -0.00126 | 0.00221 | 5.71E-01 |
| frontal pole | frontal | AD | rs73596628 | T | C | 11 | -0.07192 | 0.01423 | 3.26E-07 | 0.00902 | 0.00427 | 3.44E-02 |
| frontal pole | frontal | AD | rs748319 | T | C | 21 | -0.04577 | 0.00923 | 7.96E-07 | -0.00191 | 0.00299 | 5.23E-01 |
| frontal pole | frontal | AD | rs77128055 | A | G | 12 | -0.06692 | 0.01346 | 8.51E-07 | 0.00404 | 0.00445 | 3.64E-01 |
| frontal pole | frontal | AD | rs78759106 | T | C | 16 | -0.04500 | 0.00923 | 7.14E-07 | 0.00320 | 0.00323 | 3.22E-01 |
| frontal pole | frontal | AD | rs9828792 | A | G | 3 | -0.03692 | 0.00769 | 8.68E-07 | -0.00017 | 0.00240 | 9.42E-01 |
| lateral orbitofrontal | frontal | AD | rs11609649 | A | G | 12 | 0.03813 | 0.00750 | 1.00E-07 | -0.00001 | 0.00282 | 9.96E-01 |
| lateral orbitofrontal | frontal | AD | rs59698523 | T | C | 6 | 0.03125 | 0.00563 | 2.63E-08 | 0.00507 | 0.00530 | 3.39E-01 |
| lateral orbitofrontal | frontal | AD | rs62394879 | A | G | 5 | 0.05313 | 0.01063 | 9.84E-07 | -0.00037 | 0.00383 | 9.23E-01 |
| lateral orbitofrontal | frontal | AD | rs6983605 | A | C | 8 | -0.03063 | 0.00625 | 5.60E-07 | 0.00348 | 0.00241 | 1.48E-01 |
| medial orbitofrontal | frontal | AD | rs177080 | A | G | 7 | -0.03438 | 0.00688 | 4.76E-07 | -0.00002 | 0.00236 | 9.94E-01 |
| medial orbitofrontal | frontal | AD | rs35515850 | A | G | 1 | -0.04000 | 0.00813 | 4.35E-07 | 0.00562 | 0.00272 | 3.87E-02 |
| medial orbitofrontal | frontal | AD | rs78728690 | A | G | 12 | -0.05125 | 0.00875 | 6.05E-09 | 0.00232 | 0.00301 | 4.40E-01 |
| paracentral | frontal | AD | rs11759026 | A | G | 6 | -0.04267 | 0.00667 | 4.07E-10 | -0.00502 | 0.00252 | 4.67E-02 |
| paracentral | frontal | AD | rs2003698 | T | C | 1 | 0.03267 | 0.00667 | 5.33E-07 | 0.00505 | 0.00264 | 5.53E-02 |
| paracentral | frontal | AD | rs2525990 | A | G | 17 | 0.03600 | 0.00600 | 1.66E-08 | -0.00365 | 0.00233 | 1.17E-01 |
| paracentral | frontal | AD | rs35288226 | A | G | 9 | 0.03467 | 0.00600 | 2.35E-08 | 0.00362 | 0.00235 | 1.22E-01 |
| paracentral | frontal | AD | rs7854681 | A | G | 9 | -0.02933 | 0.00600 | 3.77E-07 | -0.00180 | 0.00226 | 4.25E-01 |
| parsopercularis | frontal | AD | rs10987671 | T | C | 9 | -0.04333 | 0.00800 | 1.76E-07 | 0.01080 | 0.00940 | 2.50E-01 |
| parsopercularis | frontal | AD | rs117421027 | C | G | 16 | -0.07333 | 0.01400 | 1.48E-07 | -0.00037 | 0.00517 | 9.42E-01 |
| parsopercularis | frontal | AD | rs12517522 | T | C | 5 | -0.02467 | 0.00533 | 9.02E-07 | 0.00005 | 0.00231 | 9.83E-01 |
| parsopercularis | frontal | AD | rs143482636 | T | C | 11 | 0.09000 | 0.01733 | 2.21E-07 | 0.02210 | 0.00788 | 5.06E-03 |
| parsopercularis | frontal | AD | rs17694988 | T | C | 15 | 0.05333 | 0.00867 | 1.47E-10 | 0.00779 | 0.00377 | 3.85E-02 |
| parsopercularis | frontal | AD | rs3740221 | T | C | 10 | -0.03067 | 0.00600 | 4.80E-07 | 0.00060 | 0.00248 | 8.08E-01 |
| parsopercularis | frontal | AD | rs3904916 | A | G | 1 | 0.03467 | 0.00733 | 9.70E-07 | -0.00177 | 0.00327 | 5.88E-01 |
| parsopercularis | frontal | AD | rs77303563 | A | T | 6 | 0.09533 | 0.01867 | 4.66E-07 | -0.00902 | 0.00822 | 2.72E-01 |
| parsopercularis | frontal | AD | rs9459027 | T | C | 6 | 0.03333 | 0.00667 | 5.55E-07 | -0.00193 | 0.00274 | 4.82E-01 |
| parsorbitalis | frontal | AD | rs10871753 | T | G | 18 | -0.03150 | 0.00550 | 3.17E-08 | -0.00080 | 0.00217 | 7.12E-01 |
| parsorbitalis | frontal | AD | rs34754191 | A | C | 11 | 0.03050 | 0.00600 | 9.45E-07 | -0.00215 | 0.00242 | 3.73E-01 |
| parsorbitalis | frontal | AD | rs72766503 | T | G | 16 | -0.04350 | 0.00700 | 4.05E-10 | 0.00566 | 0.00634 | 3.72E-01 |
| parstriangularis | frontal | AD | rs11577200 | A | G | 1 | 0.03938 | 0.00750 | 1.79E-07 | 0.00243 | 0.00308 | 4.30E-01 |
| parstriangularis | frontal | AD | rs35320047 | T | G | 5 | -0.02938 | 0.00563 | 3.69E-07 | 0.00039 | 0.00247 | 8.75E-01 |
| parstriangularis | frontal | AD | rs4243289 | C | G | 18 | 0.02375 | 0.00500 | 6.71E-07 | 0.00012 | 0.00218 | 9.56E-01 |
| parstriangularis | frontal | AD | rs6965709 | A | T | 7 | -0.02750 | 0.00500 | 1.48E-07 | -0.01060 | 0.01207 | 3.80E-01 |
| parstriangularis | frontal | AD | rs76782422 | A | C | 11 | -0.07563 | 0.01500 | 5.69E-07 | 0.00005 | 0.00618 | 9.93E-01 |
| precentral | frontal | AD | rs112216293 | A | C | 12 | 0.07400 | 0.01467 | 5.88E-07 | -0.00934 | 0.00596 | 1.17E-01 |
| precentral | frontal | AD | rs117753404 | C | G | 16 | 0.10200 | 0.02067 | 8.07E-07 | -0.00378 | 0.00808 | 6.40E-01 |
| precentral | frontal | AD | rs149866019 | T | C | 1 | -0.08533 | 0.01667 | 4.28E-07 | -0.00316 | 0.00775 | 6.84E-01 |
| precentral | frontal | AD | rs1880694 | A | G | 11 | -0.04267 | 0.00733 | 2.12E-08 | -0.00349 | 0.00328 | 2.88E-01 |
| precentral | frontal | AD | rs2277403 | A | G | 12 | -0.02333 | 0.00467 | 8.50E-07 | -0.00427 | 0.00216 | 4.79E-02 |
| precentral | frontal | AD | rs2298485 | C | G | 11 | -0.02800 | 0.00533 | 1.10E-07 | -0.00231 | 0.00220 | 2.94E-01 |
| precentral | frontal | AD | rs3752962 | T | C | 17 | -0.02667 | 0.00533 | 5.79E-07 | 0.00376 | 0.00237 | 1.13E-01 |
| precentral | frontal | AD | rs3852000 | A | G | 3 | -0.02533 | 0.00467 | 1.11E-07 | 0.00355 | 0.00224 | 1.13E-01 |
| precentral | frontal | AD | rs73150969 | A | G | 3 | -0.07600 | 0.01467 | 1.28E-07 | 0.00005 | 0.00543 | 9.92E-01 |
| rostral anterior cingulate | frontal | AD | rs116956554 | A | G | 17 | 0.05900 | 0.01100 | 8.35E-08 | -0.01127 | 0.00638 | 7.73E-02 |
| rostral anterior cingulate | frontal | AD | rs12698894 | C | G | 7 | 0.06000 | 0.01150 | 2.86E-07 | -0.00149 | 0.00349 | 6.70E-01 |
| rostral anterior cingulate | frontal | AD | rs192834490 | T | G | 12 | -0.05100 | 0.00900 | 1.93E-08 | 0.00439 | 0.00303 | 1.48E-01 |
| rostral anterior cingulate | frontal | AD | rs323483 | T | G | 18 | -0.03800 | 0.00750 | 3.09E-07 | 0.00044 | 0.00231 | 8.48E-01 |
| rostral anterior cingulate | frontal | AD | rs72768970 | T | C | 10 | -0.11250 | 0.02250 | 5.03E-07 | -0.01504 | 0.00736 | 4.09E-02 |
| rostral anterior cingulate | frontal | AD | rs77479170 | A | G | 2 | 0.03750 | 0.00750 | 6.16E-07 | 0.00505 | 0.00530 | 3.41E-01 |
| rostral anterior cingulate | frontal | AD | rs79927507 | A | G | 20 | -0.12500 | 0.02500 | 6.88E-07 | -0.00407 | 0.00684 | 5.52E-01 |
| rostral middle frontal | frontal | AD | rs10103320 | T | G | 8 | 0.03214 | 0.00643 | 1.90E-07 | 0.00506 | 0.00707 | 4.74E-01 |
| rostral middle frontal | frontal | AD | rs111297085 | T | C | 6 | 0.05643 | 0.01143 | 7.28E-07 | -0.00735 | 0.00608 | 2.27E-01 |
| rostral middle frontal | frontal | AD | rs13164785 | T | G | 5 | -0.03000 | 0.00571 | 6.94E-08 | -0.00178 | 0.00273 | 5.14E-01 |
| rostral middle frontal | frontal | AD | rs1741344 | T | C | 20 | -0.02357 | 0.00500 | 8.59E-07 | -0.00177 | 0.00225 | 4.32E-01 |
| rostral middle frontal | frontal | AD | rs2959992 | C | G | 17 | 0.03714 | 0.00714 | 4.07E-07 | -0.01277 | 0.00662 | 5.36E-02 |
| rostral middle frontal | frontal | AD | rs62320715 | T | C | 4 | -0.03571 | 0.00714 | 5.73E-07 | 0.00138 | 0.01564 | 9.30E-01 |
| rostral middle frontal | frontal | AD | rs6671321 | T | C | 1 | -0.05786 | 0.01143 | 4.32E-07 | 0.00936 | 0.00472 | 4.75E-02 |
| rostral middle frontal | frontal | AD | rs7236701 | T | G | 18 | -0.02643 | 0.00500 | 6.21E-07 | -0.00472 | 0.00548 | 3.90E-01 |
| superior frontal | frontal | AD | rs11748651 | A | T | 5 | 0.02625 | 0.00438 | 4.89E-09 | 0.00232 | 0.00236 | 3.27E-01 |
| superior frontal | frontal | AD | rs12091574 | A | G | 1 | 0.08750 | 0.01688 | 1.73E-07 | 0.00055 | 0.00797 | 9.45E-01 |
| superior frontal | frontal | AD | rs12779836 | T | C | 10 | 0.02188 | 0.00438 | 8.50E-07 | 0.00151 | 0.00227 | 5.06E-01 |
| superior frontal | frontal | AD | rs12826251 | A | G | 12 | -0.02188 | 0.00438 | 9.31E-07 | 0.00147 | 0.00222 | 5.09E-01 |
| superior frontal | frontal | AD | rs12956276 | A | G | 18 | 0.02188 | 0.00438 | 9.08E-07 | 0.00403 | 0.00227 | 7.62E-02 |
| superior frontal | frontal | AD | rs7199462 | T | G | 16 | 0.02125 | 0.00375 | 1.37E-07 | -0.00182 | 0.00217 | 4.01E-01 |
| superior frontal | frontal | AD | rs7305141 | A | G | 12 | 0.03000 | 0.00625 | 9.29E-07 | -0.00169 | 0.00284 | 5.52E-01 |
| superior frontal | frontal | AD | rs75098304 | A | G | 12 | -0.04438 | 0.00813 | 1.21E-08 | 0.00713 | 0.00409 | 8.08E-02 |
| bankssts | temporal | AD | rs11259736 | A | G | 10 | -0.04500 | 0.00875 | 4.24E-07 | 0.00097 | 0.00729 | 8.94E-01 |
| bankssts | temporal | AD | rs11710662 | A | G | 3 | -0.04188 | 0.00813 | 3.44E-07 | 0.00877 | 0.00649 | 1.77E-01 |
| bankssts | temporal | AD | rs13311548 | T | C | 7 | 0.04063 | 0.00813 | 3.16E-07 | 0.00138 | 0.00285 | 6.28E-01 |
| bankssts | temporal | AD | rs149142 | T | C | 14 | -0.05063 | 0.00625 | 2.02E-17 | 0.00050 | 0.00222 | 8.20E-01 |
| bankssts | temporal | AD | rs2416560 | A | C | 9 | 0.03250 | 0.00563 | 3.64E-08 | 0.00443 | 0.00221 | 4.55E-02 |
| entorhinal | temporal | AD | rs111658633 | T | C | 17 | -0.08091 | 0.01515 | 7.19E-08 | 0.00186 | 0.00414 | 6.54E-01 |
| entorhinal | temporal | AD | rs17790394 | T | C | 5 | 0.04939 | 0.00939 | 1.49E-07 | -0.00209 | 0.00275 | 4.47E-01 |
| entorhinal | temporal | AD | rs72812170 | A | C | 2 | -0.06394 | 0.01121 | 1.43E-08 | 0.00522 | 0.00342 | 1.27E-01 |
| entorhinal | temporal | AD | rs8044158 | A | G | 16 | 0.05455 | 0.00879 | 3.92E-10 | -0.00117 | 0.00259 | 6.51E-01 |
| fusiform | temporal | AD | rs3742960 | T | C | 15 | -0.02929 | 0.00571 | 2.72E-07 | -0.00253 | 0.00216 | 2.43E-01 |
| fusiform | temporal | AD | rs62308301 | A | G | 4 | -0.09786 | 0.02000 | 8.13E-07 | -0.01729 | 0.00816 | 3.41E-02 |
| fusiform | temporal | AD | rs6658111 | T | G | 1 | -0.04214 | 0.00643 | 1.13E-10 | 0.00318 | 0.00224 | 1.56E-01 |
| inferior temporal | temporal | AD | rs111735741 | A | G | 17 | 0.04000 | 0.00750 | 5.47E-08 | -0.00983 | 0.00620 | 1.13E-01 |
| inferior temporal | temporal | AD | rs7507079 | A | G | 18 | -0.03063 | 0.00625 | 4.88E-07 | -0.00187 | 0.00248 | 4.51E-01 |
| insula | temporal | AD | rs10980625 | T | C | 9 | 0.06313 | 0.00938 | 1.94E-11 | -0.00087 | 0.00349 | 8.04E-01 |
| insula | temporal | AD | rs11086605 | A | G | 20 | 0.08188 | 0.01563 | 2.39E-07 | -0.00787 | 0.00522 | 1.31E-01 |
| insula | temporal | AD | rs1533034 | C | G | 17 | 0.04313 | 0.00813 | 8.97E-08 | 0.00577 | 0.00676 | 3.94E-01 |
| insula | temporal | AD | rs4396807 | C | G | 22 | -0.03125 | 0.00625 | 7.92E-07 | 0.00360 | 0.00225 | 1.09E-01 |
| insula | temporal | AD | rs4843226 | T | C | 16 | -0.03250 | 0.00563 | 2.14E-08 | 0.00092 | 0.00221 | 6.76E-01 |
| insula | temporal | AD | rs72848575 | C | G | 6 | 0.03625 | 0.00750 | 6.05E-07 | 0.00252 | 0.00257 | 3.27E-01 |
| middle temporal | temporal | AD | rs10762574 | A | C | 10 | 0.02625 | 0.00563 | 9.45E-07 | 0.00299 | 0.00221 | 1.76E-01 |
| middle temporal | temporal | AD | rs1344762 | T | C | 2 | 0.03000 | 0.00563 | 2.80E-08 | -0.00166 | 0.00229 | 4.69E-01 |
| middle temporal | temporal | AD | rs1378358 | T | C | 17 | 0.03250 | 0.00625 | 4.54E-07 | -0.00387 | 0.00291 | 1.83E-01 |
| middle temporal | temporal | AD | rs2793626 | T | C | 1 | 0.03063 | 0.00625 | 6.05E-07 | 0.00084 | 0.00265 | 7.50E-01 |
| middle temporal | temporal | AD | rs4839254 | T | C | 1 | -0.02438 | 0.00500 | 6.00E-07 | 0.00078 | 0.00216 | 7.18E-01 |
| middle temporal | temporal | AD | rs6483830 | A | C | 11 | -0.03375 | 0.00625 | 3.11E-07 | 0.00396 | 0.00273 | 1.47E-01 |
| middle temporal | temporal | AD | rs7155669 | A | G | 14 | -0.03813 | 0.00563 | 4.99E-12 | -0.00061 | 0.00236 | 7.96E-01 |
| middle temporal | temporal | AD | rs7563883 | T | G | 2 | -0.03000 | 0.00625 | 9.12E-07 | 0.00400 | 0.00254 | 1.15E-01 |
| middle temporal | temporal | AD | rs7707060 | T | C | 5 | 0.02938 | 0.00563 | 1.13E-07 | 0.00148 | 0.00232 | 5.22E-01 |
| middle temporal | temporal | AD | rs79887807 | C | G | 1 | 0.04313 | 0.00813 | 1.78E-07 | 0.00075 | 0.00362 | 8.35E-01 |
| parahippocampal | temporal | AD | rs115914417 | A | G | 4 | 0.08741 | 0.01741 | 4.02E-07 | -0.00376 | 0.00566 | 5.07E-01 |
| parahippocampal | temporal | AD | rs12257242 | C | G | 10 | -0.04370 | 0.00815 | 1.07E-07 | -0.00062 | 0.00238 | 7.96E-01 |
| parahippocampal | temporal | AD | rs16909919 | T | C | 9 | -0.05185 | 0.00778 | 5.12E-11 | 0.00223 | 0.00230 | 3.32E-01 |
| parahippocampal | temporal | AD | rs17400875 | T | G | 2 | 0.04407 | 0.00889 | 4.65E-07 | 0.00048 | 0.00250 | 8.48E-01 |
| parahippocampal | temporal | AD | rs206517 | C | G | 18 | 0.04000 | 0.00815 | 9.91E-07 | 0.00332 | 0.00231 | 1.51E-01 |
| parahippocampal | temporal | AD | rs224697 | A | T | 10 | -0.05556 | 0.00889 | 2.12E-10 | -0.00283 | 0.00247 | 2.51E-01 |
| parahippocampal | temporal | AD | rs352359 | C | G | 5 | -0.04519 | 0.00852 | 1.23E-07 | -0.00072 | 0.00229 | 7.52E-01 |
| parahippocampal | temporal | AD | rs4795529 | A | G | 17 | -0.03852 | 0.00741 | 3.07E-07 | -0.00333 | 0.00216 | 1.23E-01 |
| parahippocampal | temporal | AD | rs5174 | T | C | 1 | 0.03852 | 0.00778 | 7.27E-07 | 0.00283 | 0.00222 | 2.01E-01 |
| parahippocampal | temporal | AD | rs62348867 | T | C | 4 | 0.06926 | 0.01148 | 1.66E-09 | 0.00716 | 0.00325 | 2.77E-02 |
| parahippocampal | temporal | AD | rs8028503 | A | G | 15 | -0.05889 | 0.01111 | 1.72E-07 | 0.00230 | 0.00329 | 4.84E-01 |
| parahippocampal | temporal | AD | rs8183037 | T | C | 20 | -0.03926 | 0.00778 | 3.97E-07 | 0.00156 | 0.00218 | 4.75E-01 |
| superior temporal | temporal | AD | rs11706205 | T | G | 3 | -0.04375 | 0.00813 | 1.27E-07 | -0.00359 | 0.00322 | 2.66E-01 |
| superior temporal | temporal | AD | rs1344762 | T | C | 2 | 0.02938 | 0.00563 | 8.08E-08 | -0.00166 | 0.00229 | 4.69E-01 |
| superior temporal | temporal | AD | rs150356945 | A | G | 17 | 0.08688 | 0.01750 | 5.79E-07 | 0.00019 | 0.00662 | 9.77E-01 |
| superior temporal | temporal | AD | rs199441 | A | G | 17 | -0.03688 | 0.00625 | 1.65E-08 | -0.00432 | 0.00267 | 1.06E-01 |
| superior temporal | temporal | AD | rs4843227 | T | C | 16 | -0.03313 | 0.00563 | 9.20E-10 | 0.00112 | 0.00222 | 6.15E-01 |
| superior temporal | temporal | AD | rs4857715 | A | T | 3 | 0.02813 | 0.00563 | 2.07E-07 | 0.00166 | 0.00223 | 4.58E-01 |
| superior temporal | temporal | AD | rs61784835 | T | C | 1 | -0.03875 | 0.00563 | 2.97E-11 | -0.00401 | 0.00225 | 7.42E-02 |
| superior temporal | temporal | AD | rs9472314 | T | C | 6 | -0.02688 | 0.00563 | 7.00E-07 | 0.00311 | 0.00224 | 1.64E-01 |
| superior temporal | temporal | AD | rs9844757 | T | G | 3 | 0.04688 | 0.00813 | 1.90E-08 | 0.00240 | 0.00359 | 5.04E-01 |
| temporal pole | temporal | AD | rs113981818 | A | T | 12 | -0.06645 | 0.01323 | 3.89E-07 | -0.00813 | 0.00431 | 5.91E-02 |
| temporal pole | temporal | AD | rs13344413 | A | G | 19 | -0.03742 | 0.00677 | 3.53E-08 | -0.00598 | 0.00219 | 6.31E-03 |
| temporal pole | temporal | AD | rs1948948 | T | C | 16 | 0.03710 | 0.00677 | 4.56E-08 | -0.00243 | 0.00217 | 2.64E-01 |
| temporal pole | temporal | AD | rs36004933 | A | G | 2 | -0.04903 | 0.00903 | 7.85E-08 | 0.00378 | 0.00288 | 1.89E-01 |
| temporal pole | temporal | AD | rs3858678 | A | T | 12 | 0.03806 | 0.00774 | 9.92E-07 | -0.00166 | 0.00240 | 4.90E-01 |
| transverse temporal | temporal | AD | rs117236536 | A | G | 17 | -0.09000 | 0.01810 | 6.62E-07 | 0.00207 | 0.00681 | 7.61E-01 |
| transverse temporal | temporal | AD | rs12221335 | A | T | 10 | 0.05714 | 0.01143 | 4.29E-07 | 0.00077 | 0.00374 | 8.37E-01 |
| transverse temporal | temporal | AD | rs68044532 | T | C | 9 | 0.04619 | 0.00905 | 3.34E-07 | -0.00110 | 0.00286 | 7.01E-01 |
| transverse temporal | temporal | AD | rs72831258 | T | C | 2 | -0.13143 | 0.02667 | 7.53E-07 | 0.00119 | 0.00671 | 8.59E-01 |
| inferior parietal | parietal | AD | rs111915190 | A | C | 3 | 0.08500 | 0.01714 | 4.60E-07 | -0.00204 | 0.00901 | 8.21E-01 |
| inferior parietal | parietal | AD | rs4506714 | T | C | 12 | -0.02143 | 0.00429 | 5.53E-07 | -0.00028 | 0.00240 | 9.06E-01 |
| inferior parietal | parietal | AD | rs7124343 | T | G | 11 | -0.02143 | 0.00429 | 2.21E-07 | -0.00038 | 0.00217 | 8.62E-01 |
| inferior parietal | parietal | AD | rs76659172 | T | C | 5 | -0.09357 | 0.01857 | 5.67E-07 | -0.01404 | 0.00835 | 9.27E-02 |
| inferior parietal | parietal | AD | rs77103055 | A | G | 4 | -0.03929 | 0.00786 | 5.05E-07 | -0.00256 | 0.00408 | 5.29E-01 |
| isthmus cingulate | parietal | AD | rs10067618 | A | G | 5 | 0.04263 | 0.00684 | 5.33E-10 | 0.00303 | 0.00221 | 1.71E-01 |
| isthmus cingulate | parietal | AD | rs12412476 | T | C | 10 | -0.03579 | 0.00737 | 9.47E-07 | -0.00085 | 0.00233 | 7.15E-01 |
| isthmus cingulate | parietal | AD | rs34929358 | A | G | 20 | -0.05105 | 0.01053 | 6.73E-07 | 0.00380 | 0.00333 | 2.53E-01 |
| isthmus cingulate | parietal | AD | rs3820823 | A | G | 2 | -0.04105 | 0.00684 | 2.56E-09 | 0.00024 | 0.00222 | 9.15E-01 |
| isthmus cingulate | parietal | AD | rs4962691 | T | C | 10 | -0.04895 | 0.00684 | 1.07E-12 | 0.00054 | 0.00219 | 8.04E-01 |
| isthmus cingulate | parietal | AD | rs6947897 | A | G | 7 | -0.04632 | 0.00789 | 3.07E-09 | -0.00024 | 0.00251 | 9.25E-01 |
| isthmus cingulate | parietal | AD | rs9783497 | A | G | 12 | 0.04105 | 0.00737 | 1.58E-08 | 0.00383 | 0.00227 | 9.13E-02 |
| isthmus cingulate | parietal | AD | rs9870832 | T | C | 3 | 0.03579 | 0.00684 | 2.13E-07 | -0.00089 | 0.00217 | 6.82E-01 |
| postcentral | parietal | AD | rs10498193 | A | G | 2 | -0.07000 | 0.01083 | 6.44E-11 | -0.00691 | 0.00406 | 8.85E-02 |
| postcentral | parietal | AD | rs11639391 | T | C | 15 | 0.20917 | 0.01000 | 2.07E-97 | -0.00886 | 0.00379 | 1.94E-02 |
| postcentral | parietal | AD | rs12128305 | A | T | 1 | -0.02833 | 0.00583 | 9.44E-07 | 0.00086 | 0.00566 | 8.79E-01 |
| postcentral | parietal | AD | rs13056230 | T | C | 22 | -0.03083 | 0.00583 | 7.28E-08 | -0.00091 | 0.00221 | 6.79E-01 |
| postcentral | parietal | AD | rs13118125 | T | C | 4 | -0.03250 | 0.00583 | 2.23E-08 | -0.00307 | 0.00231 | 1.84E-01 |
| postcentral | parietal | AD | rs149625242 | T | C | 17 | 0.13583 | 0.02500 | 8.87E-08 | 0.00025 | 0.00972 | 9.79E-01 |
| postcentral | parietal | AD | rs1668120 | A | C | 18 | -0.03917 | 0.00667 | 1.29E-09 | -0.00005 | 0.00242 | 9.84E-01 |
| postcentral | parietal | AD | rs1918352 | A | G | 7 | 0.03083 | 0.00583 | 6.21E-08 | -0.02834 | 0.00932 | 2.35E-03 |
| postcentral | parietal | AD | rs1938573 | T | C | 1 | -0.03833 | 0.00750 | 1.74E-07 | -0.00088 | 0.00292 | 7.64E-01 |
| postcentral | parietal | AD | rs2489009 | T | C | 10 | -0.02917 | 0.00583 | 5.42E-07 | -0.02333 | 0.01075 | 3.00E-02 |
| postcentral | parietal | AD | rs4778990 | A | C | 15 | 0.02833 | 0.00583 | 5.86E-07 | 0.00471 | 0.00216 | 2.92E-02 |
| postcentral | parietal | AD | rs4823878 | T | C | 22 | 0.04583 | 0.00667 | 7.55E-13 | 0.01102 | 0.01233 | 3.71E-01 |
| postcentral | parietal | AD | rs55686521 | T | C | 15 | 0.03500 | 0.00583 | 1.56E-09 | -0.00186 | 0.00235 | 4.30E-01 |
| postcentral | parietal | AD | rs5769797 | T | C | 22 | -0.03250 | 0.00667 | 8.97E-07 | 0.00202 | 0.00250 | 4.20E-01 |
| postcentral | parietal | AD | rs62521928 | A | G | 8 | 0.08500 | 0.01417 | 3.18E-09 | 0.00177 | 0.00516 | 7.32E-01 |
| posterior cingulate | parietal | AD | rs10051155 | A | T | 5 | 0.03133 | 0.00600 | 4.87E-07 | -0.00134 | 0.00221 | 5.45E-01 |
| posterior cingulate | parietal | AD | rs10117809 | T | C | 9 | 0.04933 | 0.00933 | 2.16E-07 | -0.00201 | 0.00328 | 5.40E-01 |
| posterior cingulate | parietal | AD | rs143550882 | A | G | 3 | -0.03733 | 0.00733 | 1.41E-07 | 0.00478 | 0.00587 | 4.16E-01 |
| posterior cingulate | parietal | AD | rs2415142 | T | G | 15 | 0.03400 | 0.00600 | 2.50E-08 | -0.00148 | 0.00217 | 4.97E-01 |
| posterior cingulate | parietal | AD | rs3111626 | A | G | 5 | -0.04133 | 0.00600 | 4.43E-11 | 0.00602 | 0.00232 | 9.55E-03 |
| posterior cingulate | parietal | AD | rs4924345 | A | C | 15 | 0.05867 | 0.01133 | 1.74E-07 | 0.00835 | 0.00401 | 3.74E-02 |
| posterior cingulate | parietal | AD | rs7700617 | A | C | 5 | 0.03000 | 0.00600 | 8.48E-07 | 0.00123 | 0.00217 | 5.70E-01 |
| precuneus | parietal | AD | rs11641011 | T | C | 16 | -0.02857 | 0.00429 | 7.22E-11 | -0.00216 | 0.00217 | 3.20E-01 |
| precuneus | parietal | AD | rs11738990 | A | C | 5 | 0.02786 | 0.00571 | 7.05E-07 | 0.00398 | 0.00296 | 1.79E-01 |
| precuneus | parietal | AD | rs12485175 | A | G | 22 | 0.02929 | 0.00500 | 2.80E-09 | 0.00361 | 0.00231 | 1.18E-01 |
| precuneus | parietal | AD | rs1452628 | A | T | 1 | -0.02214 | 0.00429 | 3.80E-07 | -0.00306 | 0.00223 | 1.69E-01 |
| precuneus | parietal | AD | rs150000053 | A | G | 2 | 0.11357 | 0.01857 | 6.71E-10 | -0.01469 | 0.00827 | 7.58E-02 |
| precuneus | parietal | AD | rs4326243 | A | C | 6 | 0.03000 | 0.00571 | 5.02E-07 | 0.00083 | 0.00270 | 7.58E-01 |
| precuneus | parietal | AD | rs623640 | A | G | 9 | 0.04714 | 0.00857 | 1.22E-08 | -0.00381 | 0.00396 | 3.36E-01 |
| precuneus | parietal | AD | rs6658111 | T | G | 1 | 0.03429 | 0.00500 | 2.41E-12 | 0.00318 | 0.00224 | 1.56E-01 |
| precuneus | parietal | AD | rs79583401 | A | G | 12 | 0.07643 | 0.01500 | 6.65E-07 | -0.00071 | 0.00699 | 9.19E-01 |
| superior parietal | parietal | AD | rs11641011 | T | C | 16 | -0.03000 | 0.00462 | 2.23E-10 | -0.00216 | 0.00217 | 3.20E-01 |
| superior parietal | parietal | AD | rs1452628 | A | T | 1 | -0.03462 | 0.00462 | 4.82E-13 | -0.00306 | 0.00223 | 1.69E-01 |
| superior parietal | parietal | AD | rs216527 | T | C | 14 | 0.02308 | 0.00462 | 8.14E-07 | -0.00435 | 0.00222 | 5.04E-02 |
| superior parietal | parietal | AD | rs4843226 | T | C | 16 | 0.04923 | 0.00462 | 1.93E-25 | 0.00092 | 0.00221 | 6.76E-01 |
| superior parietal | parietal | AD | rs61784835 | T | C | 1 | -0.02769 | 0.00538 | 1.34E-07 | -0.00401 | 0.00225 | 7.42E-02 |
| superior parietal | parietal | AD | rs72748146 | A | G | 9 | 0.03846 | 0.00615 | 1.06E-10 | -0.00991 | 0.01364 | 4.67E-01 |
| superior parietal | parietal | AD | rs73215353 | T | C | 7 | 0.04000 | 0.00769 | 7.35E-07 | -0.00262 | 0.00380 | 4.90E-01 |
| supramarginal | parietal | AD | rs11250157 | A | G | 8 | 0.01933 | 0.00400 | 9.30E-07 | -0.00071 | 0.00216 | 7.41E-01 |
| supramarginal | parietal | AD | rs2282910 | T | C | 7 | -0.01933 | 0.00400 | 8.31E-07 | -0.00410 | 0.00228 | 7.14E-02 |
| supramarginal | parietal | AD | rs72794732 | C | G | 16 | -0.04467 | 0.00933 | 9.38E-07 | 0.01027 | 0.00535 | 5.52E-02 |
| supramarginal | parietal | AD | rs7685753 | A | T | 4 | 0.02133 | 0.00400 | 6.35E-08 | 0.00278 | 0.00233 | 2.34E-01 |
| supramarginal | parietal | AD | rs78138595 | T | C | 7 | -0.03600 | 0.00733 | 9.84E-07 | -0.00306 | 0.00387 | 4.30E-01 |
| supramarginal | parietal | AD | rs865010 | T | C | 3 | 0.02267 | 0.00467 | 2.85E-07 | 0.00025 | 0.00231 | 9.12E-01 |
| cuneus | occipital | AD | rs117923045 | T | G | 8 | 0.20154 | 0.03769 | 7.69E-08 | -0.01665 | 0.01058 | 1.16E-01 |
| cuneus | occipital | AD | rs12810533 | A | G | 12 | 0.03923 | 0.00769 | 7.16E-07 | -0.00434 | 0.00245 | 7.63E-02 |
| cuneus | occipital | AD | rs13107325 | T | C | 4 | -0.07615 | 0.01308 | 6.25E-09 | 0.00770 | 0.00437 | 7.77E-02 |
| cuneus | occipital | AD | rs34163390 | T | C | 8 | 0.03385 | 0.00692 | 4.02E-07 | -0.00056 | 0.00227 | 8.05E-01 |
| cuneus | occipital | AD | rs8005394 | C | G | 14 | 0.07308 | 0.00923 | 1.54E-14 | -0.00265 | 0.00326 | 4.16E-01 |
| lateral occipital | occipital | AD | rs12953229 | A | G | 17 | 0.03308 | 0.00615 | 1.88E-08 | -0.00064 | 0.00229 | 7.80E-01 |
| lateral occipital | occipital | AD | rs13107325 | T | C | 4 | -0.06385 | 0.01077 | 1.80E-09 | 0.00770 | 0.00437 | 7.77E-02 |
| lateral occipital | occipital | AD | rs1411164 | A | C | 9 | 0.05692 | 0.01000 | 2.53E-08 | -0.00239 | 0.00404 | 5.53E-01 |
| lateral occipital | occipital | AD | rs245105 | T | C | 5 | -0.03385 | 0.00615 | 3.94E-08 | -0.00886 | 0.00251 | 4.24E-04 |
| lateral occipital | occipital | AD | rs256376 | A | G | 5 | -0.02692 | 0.00538 | 7.85E-07 | 0.00185 | 0.00227 | 4.16E-01 |
| lateral occipital | occipital | AD | rs5747112 | A | G | 22 | -0.02692 | 0.00538 | 4.73E-07 | -0.00333 | 0.00217 | 1.25E-01 |
| lateral occipital | occipital | AD | rs595789 | T | C | 2 | 0.02615 | 0.00538 | 8.42E-07 | 0.00038 | 0.00218 | 8.62E-01 |
| lateral occipital | occipital | AD | rs62200349 | A | G | 20 | 0.03692 | 0.00692 | 2.10E-08 | -0.00349 | 0.00274 | 2.03E-01 |
| lingual | occipital | AD | rs10495963 | T | C | 2 | 0.04000 | 0.00667 | 6.06E-10 | 0.00441 | 0.00248 | 7.47E-02 |
| lingual | occipital | AD | rs11760588 | A | G | 7 | 0.04750 | 0.00917 | 3.17E-07 | 0.00394 | 0.00316 | 2.13E-01 |
| lingual | occipital | AD | rs12591705 | T | C | 15 | 0.02917 | 0.00583 | 3.56E-07 | 0.00037 | 0.00217 | 8.66E-01 |
| lingual | occipital | AD | rs13135092 | A | G | 4 | 0.06167 | 0.01167 | 1.77E-07 | -0.00750 | 0.00417 | 7.22E-02 |
| lingual | occipital | AD | rs13180395 | T | C | 5 | 0.03917 | 0.00750 | 1.31E-07 | -0.00567 | 0.00596 | 3.42E-01 |
| lingual | occipital | AD | rs1391550 | A | G | 1 | 0.03167 | 0.00667 | 6.21E-07 | 0.00079 | 0.00237 | 7.39E-01 |
| lingual | occipital | AD | rs73239710 | A | C | 7 | -0.03333 | 0.00667 | 1.96E-07 | -0.00270 | 0.00241 | 2.62E-01 |
| lingual | occipital | AD | rs9537915 | T | C | 13 | -0.03583 | 0.00583 | 8.64E-10 | -0.00206 | 0.00528 | 6.97E-01 |
| pericalcarine | occipital | AD | rs13262416 | C | G | 8 | -0.05000 | 0.00917 | 1.42E-08 | 0.00108 | 0.00255 | 6.73E-01 |
| pericalcarine | occipital | AD | rs2164950 | A | G | 14 | -0.06250 | 0.01000 | 5.38E-10 | 0.00389 | 0.00330 | 2.39E-01 |
| pericalcarine | occipital | AD | rs34324736 | T | C | 3 | -0.04167 | 0.00750 | 5.71E-09 | 0.00938 | 0.00531 | 7.71E-02 |
| pericalcarine | occipital | AD | rs4682946 | T | C | 3 | -0.04250 | 0.00833 | 5.36E-07 | -0.02741 | 0.01514 | 7.02E-02 |
| pericalcarine | occipital | AD | rs629404 | A | T | 12 | 0.03833 | 0.00750 | 7.73E-07 | -0.00245 | 0.00255 | 3.37E-01 |
| whole cortex | global | AD | rs11656696 | A | C | 17 | 0.03636 | 0.00727 | 2.12E-07 | 0.00625 | 0.00535 | 2.43E-01 |
| whole cortex | global | AD | rs11692435 | A | G | 2 | -0.08273 | 0.01364 | 3.18E-10 | 0.00217 | 0.00392 | 5.80E-01 |
| whole cortex | global | AD | rs117826338 | T | C | 19 | 0.05636 | 0.01091 | 9.90E-08 | -0.01044 | 0.00321 | 1.15E-03 |
| whole cortex | global | AD | rs1180331 | A | G | 1 | 0.03545 | 0.00727 | 5.30E-07 | -0.00173 | 0.00216 | 4.23E-01 |
| whole cortex | global | AD | rs12938775 | A | G | 17 | -0.03909 | 0.00818 | 6.87E-07 | -0.00036 | 0.00216 | 8.69E-01 |
| whole cortex | global | AD | rs13107325 | T | C | 4 | -0.06909 | 0.01364 | 5.05E-07 | 0.00770 | 0.00437 | 7.77E-02 |
| whole cortex | global | AD | rs1742401 | A | G | 16 | -0.03455 | 0.00727 | 7.05E-07 | -0.00002 | 0.00221 | 9.93E-01 |
| whole cortex | global | AD | rs2316766 | T | G | 17 | 0.06273 | 0.01000 | 2.90E-10 | -0.01540 | 0.00617 | 1.25E-02 |
| whole cortex | global | AD | rs3200031 | T | C | 8 | 0.06455 | 0.01273 | 5.53E-07 | -0.00157 | 0.00439 | 7.21E-01 |
| whole cortex | global | AD | rs35021943 | A | C | 4 | -0.04636 | 0.00818 | 2.98E-09 | 0.00158 | 0.00252 | 5.31E-01 |
| whole cortex | global | AD | rs3770776 | A | G | 2 | -0.03545 | 0.00727 | 3.17E-07 | -0.00204 | 0.00218 | 3.49E-01 |
| whole cortex | global | AD | rs3816046 | T | C | 19 | -0.03727 | 0.00727 | 8.46E-07 | -0.00418 | 0.00231 | 7.04E-02 |
| whole cortex | global | AD | rs40565 | T | C | 5 | 0.04364 | 0.00909 | 5.91E-07 | -0.00005 | 0.00271 | 9.85E-01 |
| whole cortex | global | AD | rs533577 | T | C | 3 | -0.04545 | 0.00727 | 8.43E-11 | -0.00125 | 0.00216 | 5.62E-01 |
| whole cortex | global | AD | rs5994871 | T | C | 22 | 0.03818 | 0.00818 | 8.82E-07 | -0.00019 | 0.00238 | 9.37E-01 |
| whole cortex | global | AD | rs6738528 | A | T | 2 | 0.04091 | 0.00727 | 7.32E-09 | -0.00009 | 0.00221 | 9.66E-01 |
| whole cortex | global | AD | rs724265 | A | G | 8 | 0.03727 | 0.00727 | 1.01E-07 | -0.00130 | 0.00230 | 5.73E-01 |
| whole cortex | global | AD | rs7531555 | T | C | 1 | 0.04273 | 0.00818 | 7.66E-08 | 0.00184 | 0.00251 | 4.62E-01 |
| whole cortex | global | AD | rs7657284 | A | C | 4 | -0.04000 | 0.00818 | 2.68E-07 | -0.00072 | 0.00256 | 7.79E-01 |
| whole cortex | global | AD | rs7824177 | A | G | 8 | 0.05364 | 0.00909 | 8.92E-09 | 0.00092 | 0.00285 | 7.48E-01 |

**Table S4.** Summary of genetic variants used to estimate the effect of Alzheimer's disease (AD) on cortical surface area

| **Exposure** | **outcome** | **SNP** | **effect allele** | **other allele** | **beta exposure** | **se exposure** | **pval exposure** | **beta outcome** | **se outcome** | **pval outcome** |
| --- | --- | --- | --- | --- | --- | --- | --- | --- | --- | --- |
| AD | Caudal anterior cingulate | rs10933431 | G | C | -0.01544 | 0.00251 | 7.62E-10 | 0.4726 | 0.9992 | 6.36E-01 |
| AD | Caudal anterior cingulate | rs111278892 | G | C | 0.01991 | 0.00305 | 6.67E-11 | -1.1529 | 1.1735 | 3.26E-01 |
| AD | Caudal anterior cingulate | rs11218343 | C | T | -0.03593 | 0.00526 | 8.12E-12 | 2.0161 | 1.9256 | 2.95E-01 |
| AD | Caudal anterior cingulate | rs11257238 | C | T | 0.01294 | 0.00226 | 1.04E-08 | -0.8206 | 0.8164 | 3.15E-01 |
| AD | Caudal anterior cingulate | rs113260531 | A | G | 0.01999 | 0.00325 | 7.91E-10 | -0.157 | 1.1472 | 8.91E-01 |
| AD | Caudal anterior cingulate | rs118170342 | C | T | 0.14754 | 0.00570 | 7.93E-148 | -3.2298 | 2.3716 | 1.73E-01 |
| AD | Caudal anterior cingulate | rs12590654 | A | G | -0.01483 | 0.00231 | 1.32E-10 | 1.0226 | 0.8189 | 2.12E-01 |
| AD | Caudal anterior cingulate | rs1859788 | A | G | -0.01840 | 0.00231 | 1.80E-15 | 0.1793 | 0.8477 | 8.33E-01 |
| AD | Caudal anterior cingulate | rs204473 | A | G | -0.04167 | 0.00700 | 2.58E-09 | -1.0028 | 2.5077 | 6.89E-01 |
| AD | Caudal anterior cingulate | rs2081545 | A | C | -0.01787 | 0.00223 | 1.11E-15 | -0.0676 | 0.7694 | 9.30E-01 |
| AD | Caudal anterior cingulate | rs28394864 | A | G | 0.01230 | 0.00218 | 1.68E-08 | 0.489 | 0.7581 | 5.19E-01 |
| AD | Caudal anterior cingulate | rs28399657 | G | A | -0.05464 | 0.00658 | 9.82E-17 | -3.3925 | 2.3086 | 1.42E-01 |
| AD | Caudal anterior cingulate | rs41289512 | G | C | 0.20630 | 0.00578 | 1.00E-200 | -2.6055 | 2.3255 | 2.63E-01 |
| AD | Caudal anterior cingulate | rs41290120 | A | G | -0.09905 | 0.00578 | 7.14E-66 | 1.832 | 1.9451 | 3.46E-01 |
| AD | Caudal anterior cingulate | rs4236673 | A | G | -0.02016 | 0.00223 | 1.48E-19 | 0.2355 | 0.7777 | 7.62E-01 |
| AD | Caudal anterior cingulate | rs442495 | C | T | -0.01372 | 0.00226 | 1.22E-09 | -1.3016 | 0.8091 | 1.08E-01 |
| AD | Caudal anterior cingulate | rs4575098 | A | G | 0.01641 | 0.00258 | 1.90E-10 | 0.6003 | 0.8885 | 4.99E-01 |
| AD | Caudal anterior cingulate | rs4663105 | C | A | 0.03110 | 0.00222 | 1.45E-44 | -0.7919 | 0.7968 | 3.20E-01 |
| AD | Caudal anterior cingulate | rs59735493 | A | G | -0.01299 | 0.00236 | 3.73E-08 | -1.2272 | 0.8278 | 1.38E-01 |
| AD | Caudal anterior cingulate | rs6014724 | G | A | -0.02289 | 0.00369 | 5.38E-10 | -1.1955 | 1.3622 | 3.80E-01 |
| AD | Caudal anterior cingulate | rs6448453 | A | G | 0.01470 | 0.00245 | 1.98E-09 | -0.3241 | 0.8497 | 7.03E-01 |
| AD | Caudal anterior cingulate | rs679515 | T | C | 0.02542 | 0.00286 | 6.83E-19 | 0.866 | 0.9789 | 3.76E-01 |
| AD | Caudal anterior cingulate | rs755951 | C | A | 0.01500 | 0.00221 | 1.13E-11 | -1.4582 | 0.769 | 5.79E-02 |
| AD | Caudal anterior cingulate | rs7810606 | T | C | -0.01452 | 0.00218 | 2.89E-11 | 0.0886 | 0.7742 | 9.09E-01 |
| AD | Caudal anterior cingulate | rs846881 | C | A | -0.01737 | 0.00269 | 9.89E-11 | 0.3902 | 1.0483 | 7.10E-01 |
| AD | Caudal anterior cingulate | rs867611 | G | A | -0.02043 | 0.00232 | 1.48E-18 | -0.3128 | 0.8076 | 6.99E-01 |
| AD | Caudal anterior cingulate | rs9381563 | C | T | 0.01445 | 0.00227 | 1.99E-10 | 0.1056 | 0.7922 | 8.94E-01 |
| AD | Caudal middle frontal | rs10933431 | G | C | -0.01544 | 0.00251 | 7.62E-10 | -0.3283 | 2.6258 | 9.01E-01 |
| AD | Caudal middle frontal | rs111278892 | G | C | 0.01991 | 0.00305 | 6.67E-11 | 1.1216 | 3.0623 | 7.14E-01 |
| AD | Caudal middle frontal | rs11218343 | C | T | -0.03593 | 0.00526 | 8.12E-12 | -8.1536 | 5.1133 | 1.11E-01 |
| AD | Caudal middle frontal | rs11257238 | C | T | 0.01294 | 0.00226 | 1.04E-08 | 0.1217 | 2.1516 | 9.55E-01 |
| AD | Caudal middle frontal | rs113260531 | A | G | 0.01999 | 0.00325 | 7.91E-10 | -4.8963 | 3.0238 | 1.05E-01 |
| AD | Caudal middle frontal | rs118170342 | C | T | 0.14754 | 0.00570 | 7.93E-148 | -7.6891 | 6.1847 | 2.14E-01 |
| AD | Caudal middle frontal | rs12590654 | A | G | -0.01483 | 0.00231 | 1.32E-10 | 1.0982 | 2.1586 | 6.11E-01 |
| AD | Caudal middle frontal | rs1859788 | A | G | -0.01840 | 0.00231 | 1.80E-15 | 0.3385 | 2.2321 | 8.80E-01 |
| AD | Caudal middle frontal | rs204473 | A | G | -0.04167 | 0.00700 | 2.58E-09 | 7.5798 | 6.5553 | 2.48E-01 |
| AD | Caudal middle frontal | rs2081545 | A | C | -0.01787 | 0.00223 | 1.11E-15 | 0.4073 | 2.0324 | 8.41E-01 |
| AD | Caudal middle frontal | rs28394864 | A | G | 0.01230 | 0.00218 | 1.68E-08 | 2.3443 | 2.0018 | 2.42E-01 |
| AD | Caudal middle frontal | rs28399657 | G | A | -0.05464 | 0.00658 | 9.82E-17 | 3.4366 | 6.0226 | 5.68E-01 |
| AD | Caudal middle frontal | rs41289512 | G | C | 0.20630 | 0.00578 | 1.00E-200 | -1.1227 | 6.0661 | 8.53E-01 |
| AD | Caudal middle frontal | rs41290120 | A | G | -0.09905 | 0.00578 | 7.14E-66 | 6.6725 | 5.1192 | 1.92E-01 |
| AD | Caudal middle frontal | rs4236673 | A | G | -0.02016 | 0.00223 | 1.48E-19 | -2.357 | 2.0519 | 2.51E-01 |
| AD | Caudal middle frontal | rs442495 | C | T | -0.01372 | 0.00226 | 1.22E-09 | 2.3829 | 2.1418 | 2.66E-01 |
| AD | Caudal middle frontal | rs4575098 | A | G | 0.01641 | 0.00258 | 1.90E-10 | -3.9146 | 2.3483 | 9.55E-02 |
| AD | Caudal middle frontal | rs4663105 | C | A | 0.03110 | 0.00222 | 1.45E-44 | 1.7792 | 2.0965 | 3.96E-01 |
| AD | Caudal middle frontal | rs59735493 | A | G | -0.01299 | 0.00236 | 3.73E-08 | 2.2453 | 2.188 | 3.05E-01 |
| AD | Caudal middle frontal | rs6014724 | G | A | -0.02289 | 0.00369 | 5.38E-10 | -2.2996 | 3.6039 | 5.23E-01 |
| AD | Caudal middle frontal | rs6448453 | A | G | 0.01470 | 0.00245 | 1.98E-09 | 1.5822 | 2.2445 | 4.81E-01 |
| AD | Caudal middle frontal | rs679515 | T | C | 0.02542 | 0.00286 | 6.83E-19 | 2.0062 | 2.5898 | 4.39E-01 |
| AD | Caudal middle frontal | rs755951 | C | A | 0.01500 | 0.00221 | 1.13E-11 | -1.4411 | 2.0297 | 4.78E-01 |
| AD | Caudal middle frontal | rs7810606 | T | C | -0.01452 | 0.00218 | 2.89E-11 | -0.9929 | 2.0449 | 6.27E-01 |
| AD | Caudal middle frontal | rs846881 | C | A | -0.01737 | 0.00269 | 9.89E-11 | 1.9519 | 2.7427 | 4.77E-01 |
| AD | Caudal middle frontal | rs867611 | G | A | -0.02043 | 0.00232 | 1.48E-18 | -2.9055 | 2.1315 | 1.73E-01 |
| AD | Caudal middle frontal | rs9381563 | C | T | 0.01445 | 0.00227 | 1.99E-10 | 4.9846 | 2.0941 | 1.73E-02 |
| AD | Frontal pole | rs10933431 | G | C | -0.01544 | 0.00251 | 7.62E-10 | -0.0524 | 0.2958 | 8.60E-01 |
| AD | Frontal pole | rs111278892 | G | C | 0.01991 | 0.00305 | 6.67E-11 | 0.1392 | 0.3457 | 6.87E-01 |
| AD | Frontal pole | rs11218343 | C | T | -0.03593 | 0.00526 | 8.12E-12 | 1.2742 | 0.577 | 2.72E-02 |
| AD | Frontal pole | rs11257238 | C | T | 0.01294 | 0.00226 | 1.04E-08 | -0.0303 | 0.2426 | 9.01E-01 |
| AD | Frontal pole | rs113260531 | A | G | 0.01999 | 0.00325 | 7.91E-10 | -0.4176 | 0.342 | 2.22E-01 |
| AD | Frontal pole | rs118170342 | C | T | 0.14754 | 0.00570 | 7.93E-148 | 0.1897 | 0.698 | 7.86E-01 |
| AD | Frontal pole | rs12590654 | A | G | -0.01483 | 0.00231 | 1.32E-10 | 0.176 | 0.2439 | 4.71E-01 |
| AD | Frontal pole | rs1859788 | A | G | -0.01840 | 0.00231 | 1.80E-15 | -0.152 | 0.252 | 5.46E-01 |
| AD | Frontal pole | rs204473 | A | G | -0.04167 | 0.00700 | 2.58E-09 | -0.2657 | 0.7401 | 7.20E-01 |
| AD | Frontal pole | rs2081545 | A | C | -0.01787 | 0.00223 | 1.11E-15 | 0.2877 | 0.2293 | 2.10E-01 |
| AD | Frontal pole | rs28394864 | A | G | 0.01230 | 0.00218 | 1.68E-08 | -0.1693 | 0.226 | 4.54E-01 |
| AD | Frontal pole | rs28399657 | G | A | -0.05464 | 0.00658 | 9.82E-17 | 0.1024 | 0.6831 | 8.81E-01 |
| AD | Frontal pole | rs41289512 | G | C | 0.20630 | 0.00578 | 1.00E-200 | 0.7763 | 0.6874 | 2.59E-01 |
| AD | Frontal pole | rs41290120 | A | G | -0.09905 | 0.00578 | 7.14E-66 | -0.4989 | 0.5744 | 3.85E-01 |
| AD | Frontal pole | rs4236673 | A | G | -0.02016 | 0.00223 | 1.48E-19 | -0.3774 | 0.232 | 1.04E-01 |
| AD | Frontal pole | rs442495 | C | T | -0.01372 | 0.00226 | 1.22E-09 | -0.0697 | 0.2413 | 7.73E-01 |
| AD | Frontal pole | rs4575098 | A | G | 0.01641 | 0.00258 | 1.90E-10 | 0.1641 | 0.2652 | 5.36E-01 |
| AD | Frontal pole | rs4663105 | C | A | 0.03110 | 0.00222 | 1.45E-44 | -0.0059 | 0.2369 | 9.80E-01 |
| AD | Frontal pole | rs59735493 | A | G | -0.01299 | 0.00236 | 3.73E-08 | 0.082 | 0.2469 | 7.40E-01 |
| AD | Frontal pole | rs6014724 | G | A | -0.02289 | 0.00369 | 5.38E-10 | -0.1481 | 0.4059 | 7.15E-01 |
| AD | Frontal pole | rs6448453 | A | G | 0.01470 | 0.00245 | 1.98E-09 | -0.2641 | 0.2534 | 2.97E-01 |
| AD | Frontal pole | rs679515 | T | C | 0.02542 | 0.00286 | 6.83E-19 | 0.1631 | 0.2922 | 5.77E-01 |
| AD | Frontal pole | rs755951 | C | A | 0.01500 | 0.00221 | 1.13E-11 | -0.2015 | 0.2291 | 3.79E-01 |
| AD | Frontal pole | rs7810606 | T | C | -0.01452 | 0.00218 | 2.89E-11 | -0.0361 | 0.2309 | 8.76E-01 |
| AD | Frontal pole | rs846881 | C | A | -0.01737 | 0.00269 | 9.89E-11 | 0.117 | 0.3095 | 7.05E-01 |
| AD | Frontal pole | rs867611 | G | A | -0.02043 | 0.00232 | 1.48E-18 | -0.5093 | 0.2404 | 3.42E-02 |
| AD | Frontal pole | rs9381563 | C | T | 0.01445 | 0.00227 | 1.99E-10 | 0.2046 | 0.2363 | 3.87E-01 |
| AD | Lateral orbitofrontal | rs10933431 | G | C | -0.01544 | 0.00251 | 7.62E-10 | 2.5393 | 1.8247 | 1.64E-01 |
| AD | Lateral orbitofrontal | rs111278892 | G | C | 0.01991 | 0.00305 | 6.67E-11 | -1.4689 | 2.1272 | 4.90E-01 |
| AD | Lateral orbitofrontal | rs11218343 | C | T | -0.03593 | 0.00526 | 8.12E-12 | 1.8962 | 3.5666 | 5.95E-01 |
| AD | Lateral orbitofrontal | rs11257238 | C | T | 0.01294 | 0.00226 | 1.04E-08 | 0.9732 | 1.5002 | 5.17E-01 |
| AD | Lateral orbitofrontal | rs113260531 | A | G | 0.01999 | 0.00325 | 7.91E-10 | 1.4272 | 2.1081 | 4.98E-01 |
| AD | Lateral orbitofrontal | rs118170342 | C | T | 0.14754 | 0.00570 | 7.93E-148 | -2.8642 | 4.2522 | 5.01E-01 |
| AD | Lateral orbitofrontal | rs12590654 | A | G | -0.01483 | 0.00231 | 1.32E-10 | -1.1148 | 1.5095 | 4.60E-01 |
| AD | Lateral orbitofrontal | rs1859788 | A | G | -0.01840 | 0.00231 | 1.80E-15 | 2.1698 | 1.5579 | 1.64E-01 |
| AD | Lateral orbitofrontal | rs204473 | A | G | -0.04167 | 0.00700 | 2.58E-09 | -5.3727 | 4.5451 | 2.37E-01 |
| AD | Lateral orbitofrontal | rs2081545 | A | C | -0.01787 | 0.00223 | 1.11E-15 | 0.652 | 1.4196 | 6.46E-01 |
| AD | Lateral orbitofrontal | rs28394864 | A | G | 0.01230 | 0.00218 | 1.68E-08 | 0.5699 | 1.3984 | 6.84E-01 |
| AD | Lateral orbitofrontal | rs28399657 | G | A | -0.05464 | 0.00658 | 9.82E-17 | 3.9156 | 4.1909 | 3.50E-01 |
| AD | Lateral orbitofrontal | rs41289512 | G | C | 0.20630 | 0.00578 | 1.00E-200 | -8.1488 | 4.1651 | 5.04E-02 |
| AD | Lateral orbitofrontal | rs41290120 | A | G | -0.09905 | 0.00578 | 7.14E-66 | -2.594 | 3.5691 | 4.67E-01 |
| AD | Lateral orbitofrontal | rs4236673 | A | G | -0.02016 | 0.00223 | 1.48E-19 | 1.1704 | 1.4296 | 4.13E-01 |
| AD | Lateral orbitofrontal | rs442495 | C | T | -0.01372 | 0.00226 | 1.22E-09 | 1.0598 | 1.4927 | 4.78E-01 |
| AD | Lateral orbitofrontal | rs4575098 | A | G | 0.01641 | 0.00258 | 1.90E-10 | 2.2347 | 1.6392 | 1.73E-01 |
| AD | Lateral orbitofrontal | rs4663105 | C | A | 0.03110 | 0.00222 | 1.45E-44 | 1.9928 | 1.4627 | 1.73E-01 |
| AD | Lateral orbitofrontal | rs59735493 | A | G | -0.01299 | 0.00236 | 3.73E-08 | -1.5339 | 1.5274 | 3.15E-01 |
| AD | Lateral orbitofrontal | rs6014724 | G | A | -0.02289 | 0.00369 | 5.38E-10 | 3.0069 | 2.5155 | 2.32E-01 |
| AD | Lateral orbitofrontal | rs6448453 | A | G | 0.01470 | 0.00245 | 1.98E-09 | 0.9313 | 1.5705 | 5.53E-01 |
| AD | Lateral orbitofrontal | rs679515 | T | C | 0.02542 | 0.00286 | 6.83E-19 | -2.9361 | 1.8068 | 1.04E-01 |
| AD | Lateral orbitofrontal | rs755951 | C | A | 0.01500 | 0.00221 | 1.13E-11 | -0.2996 | 1.4187 | 8.33E-01 |
| AD | Lateral orbitofrontal | rs7810606 | T | C | -0.01452 | 0.00218 | 2.89E-11 | -0.521 | 1.4232 | 7.14E-01 |
| AD | Lateral orbitofrontal | rs846881 | C | A | -0.01737 | 0.00269 | 9.89E-11 | 1.1752 | 1.9059 | 5.38E-01 |
| AD | Lateral orbitofrontal | rs867611 | G | A | -0.02043 | 0.00232 | 1.48E-18 | 1.4648 | 1.4894 | 3.25E-01 |
| AD | Lateral orbitofrontal | rs9381563 | C | T | 0.01445 | 0.00227 | 1.99E-10 | -1.1796 | 1.4607 | 4.19E-01 |
| AD | Medial orbitofrontal | rs10933431 | G | C | -0.01544 | 0.00251 | 7.62E-10 | 0.8391 | 1.3606 | 5.37E-01 |
| AD | Medial orbitofrontal | rs111278892 | G | C | 0.01991 | 0.00305 | 6.67E-11 | -3.1614 | 1.5883 | 4.65E-02 |
| AD | Medial orbitofrontal | rs11218343 | C | T | -0.03593 | 0.00526 | 8.12E-12 | 1.0807 | 2.6403 | 6.82E-01 |
| AD | Medial orbitofrontal | rs11257238 | C | T | 0.01294 | 0.00226 | 1.04E-08 | -0.5864 | 1.1173 | 6.00E-01 |
| AD | Medial orbitofrontal | rs113260531 | A | G | 0.01999 | 0.00325 | 7.91E-10 | 0.408 | 1.5667 | 7.95E-01 |
| AD | Medial orbitofrontal | rs118170342 | C | T | 0.14754 | 0.00570 | 7.93E-148 | 4.704 | 3.1857 | 1.40E-01 |
| AD | Medial orbitofrontal | rs12590654 | A | G | -0.01483 | 0.00231 | 1.32E-10 | 1.2267 | 1.1205 | 2.74E-01 |
| AD | Medial orbitofrontal | rs1859788 | A | G | -0.01840 | 0.00231 | 1.80E-15 | 0.9463 | 1.159 | 4.14E-01 |
| AD | Medial orbitofrontal | rs204473 | A | G | -0.04167 | 0.00700 | 2.58E-09 | -2.7238 | 3.3901 | 4.22E-01 |
| AD | Medial orbitofrontal | rs2081545 | A | C | -0.01787 | 0.00223 | 1.11E-15 | -0.489 | 1.0541 | 6.43E-01 |
| AD | Medial orbitofrontal | rs28394864 | A | G | 0.01230 | 0.00218 | 1.68E-08 | 0.4168 | 1.0383 | 6.88E-01 |
| AD | Medial orbitofrontal | rs28399657 | G | A | -0.05464 | 0.00658 | 9.82E-17 | 1.3366 | 3.1397 | 6.70E-01 |
| AD | Medial orbitofrontal | rs41289512 | G | C | 0.20630 | 0.00578 | 1.00E-200 | -2.7988 | 3.1421 | 3.73E-01 |
| AD | Medial orbitofrontal | rs41290120 | A | G | -0.09905 | 0.00578 | 7.14E-66 | 3.4725 | 2.6655 | 1.93E-01 |
| AD | Medial orbitofrontal | rs4236673 | A | G | -0.02016 | 0.00223 | 1.48E-19 | -0.6475 | 1.0625 | 5.42E-01 |
| AD | Medial orbitofrontal | rs442495 | C | T | -0.01372 | 0.00226 | 1.22E-09 | 1.0285 | 1.1075 | 3.53E-01 |
| AD | Medial orbitofrontal | rs4575098 | A | G | 0.01641 | 0.00258 | 1.90E-10 | 1.8171 | 1.2173 | 1.36E-01 |
| AD | Medial orbitofrontal | rs4663105 | C | A | 0.03110 | 0.00222 | 1.45E-44 | -1.4251 | 1.0873 | 1.90E-01 |
| AD | Medial orbitofrontal | rs59735493 | A | G | -0.01299 | 0.00236 | 3.73E-08 | -0.9075 | 1.133 | 4.23E-01 |
| AD | Medial orbitofrontal | rs6014724 | G | A | -0.02289 | 0.00369 | 5.38E-10 | 1.381 | 1.8675 | 4.60E-01 |
| AD | Medial orbitofrontal | rs6448453 | A | G | 0.01470 | 0.00245 | 1.98E-09 | 1.8848 | 1.1632 | 1.05E-01 |
| AD | Medial orbitofrontal | rs679515 | T | C | 0.02542 | 0.00286 | 6.83E-19 | 2.6236 | 1.341 | 5.04E-02 |
| AD | Medial orbitofrontal | rs755951 | C | A | 0.01500 | 0.00221 | 1.13E-11 | 0.6374 | 1.052 | 5.45E-01 |
| AD | Medial orbitofrontal | rs7810606 | T | C | -0.01452 | 0.00218 | 2.89E-11 | -0.6311 | 1.0583 | 5.51E-01 |
| AD | Medial orbitofrontal | rs846881 | C | A | -0.01737 | 0.00269 | 9.89E-11 | 1.537 | 1.4208 | 2.79E-01 |
| AD | Medial orbitofrontal | rs867611 | G | A | -0.02043 | 0.00232 | 1.48E-18 | 0.9346 | 1.1048 | 3.98E-01 |
| AD | Medial orbitofrontal | rs9381563 | C | T | 0.01445 | 0.00227 | 1.99E-10 | -0.6923 | 1.0852 | 5.24E-01 |
| AD | paracentral | rs10933431 | G | C | -0.01544 | 0.00251 | 7.62E-10 | 0.8906 | 1.4947 | 5.51E-01 |
| AD | paracentral | rs111278892 | G | C | 0.01991 | 0.00305 | 6.67E-11 | -2.6799 | 1.7369 | 1.23E-01 |
| AD | paracentral | rs11218343 | C | T | -0.03593 | 0.00526 | 8.12E-12 | -1.0489 | 2.8743 | 7.15E-01 |
| AD | paracentral | rs11257238 | C | T | 0.01294 | 0.00226 | 1.04E-08 | 0.424 | 1.2139 | 7.27E-01 |
| AD | paracentral | rs113260531 | A | G | 0.01999 | 0.00325 | 7.91E-10 | -1.5563 | 1.7043 | 3.61E-01 |
| AD | paracentral | rs118170342 | C | T | 0.14754 | 0.00570 | 7.93E-148 | 3.9422 | 3.504 | 2.61E-01 |
| AD | paracentral | rs12590654 | A | G | -0.01483 | 0.00231 | 1.32E-10 | 0.7305 | 1.2181 | 5.49E-01 |
| AD | paracentral | rs1859788 | A | G | -0.01840 | 0.00231 | 1.80E-15 | 0.1186 | 1.2615 | 9.25E-01 |
| AD | paracentral | rs204473 | A | G | -0.04167 | 0.00700 | 2.58E-09 | 0.4647 | 3.6902 | 9.00E-01 |
| AD | paracentral | rs2081545 | A | C | -0.01787 | 0.00223 | 1.11E-15 | 1.3712 | 1.1447 | 2.31E-01 |
| AD | paracentral | rs28394864 | A | G | 0.01230 | 0.00218 | 1.68E-08 | -1.6647 | 1.1277 | 1.40E-01 |
| AD | paracentral | rs28399657 | G | A | -0.05464 | 0.00658 | 9.82E-17 | -2.4983 | 3.4017 | 4.63E-01 |
| AD | paracentral | rs41289512 | G | C | 0.20630 | 0.00578 | 1.00E-200 | 5.6205 | 3.4529 | 1.04E-01 |
| AD | paracentral | rs41290120 | A | G | -0.09905 | 0.00578 | 7.14E-66 | -2.0925 | 2.8878 | 4.69E-01 |
| AD | paracentral | rs4236673 | A | G | -0.02016 | 0.00223 | 1.48E-19 | -0.9004 | 1.1548 | 4.36E-01 |
| AD | paracentral | rs442495 | C | T | -0.01372 | 0.00226 | 1.22E-09 | -1.3421 | 1.2066 | 2.66E-01 |
| AD | paracentral | rs4575098 | A | G | 0.01641 | 0.00258 | 1.90E-10 | -1.1784 | 1.3208 | 3.72E-01 |
| AD | paracentral | rs4663105 | C | A | 0.03110 | 0.00222 | 1.45E-44 | -1.3011 | 1.1831 | 2.72E-01 |
| AD | paracentral | rs59735493 | A | G | -0.01299 | 0.00236 | 3.73E-08 | 1.3561 | 1.2324 | 2.71E-01 |
| AD | paracentral | rs6014724 | G | A | -0.02289 | 0.00369 | 5.38E-10 | -0.6443 | 2.031 | 7.51E-01 |
| AD | paracentral | rs6448453 | A | G | 0.01470 | 0.00245 | 1.98E-09 | 0.3118 | 1.2646 | 8.05E-01 |
| AD | paracentral | rs679515 | T | C | 0.02542 | 0.00286 | 6.83E-19 | -1.1905 | 1.4578 | 4.14E-01 |
| AD | paracentral | rs755951 | C | A | 0.01500 | 0.00221 | 1.13E-11 | -0.2982 | 1.1432 | 7.94E-01 |
| AD | paracentral | rs7810606 | T | C | -0.01452 | 0.00218 | 2.89E-11 | 0.2788 | 1.1521 | 8.09E-01 |
| AD | paracentral | rs846881 | C | A | -0.01737 | 0.00269 | 9.89E-11 | -0.3791 | 1.5581 | 8.08E-01 |
| AD | paracentral | rs867611 | G | A | -0.02043 | 0.00232 | 1.48E-18 | 0.986 | 1.2008 | 4.12E-01 |
| AD | paracentral | rs9381563 | C | T | 0.01445 | 0.00227 | 1.99E-10 | -0.5817 | 1.1792 | 6.22E-01 |
| AD | Pars opercularis | rs10933431 | G | C | -0.01544 | 0.00251 | 7.62E-10 | -0.6491 | 1.7456 | 7.10E-01 |
| AD | Pars opercularis | rs111278892 | G | C | 0.01991 | 0.00305 | 6.67E-11 | 0.8884 | 2.0386 | 6.63E-01 |
| AD | Pars opercularis | rs11218343 | C | T | -0.03593 | 0.00526 | 8.12E-12 | -2.5315 | 3.4131 | 4.58E-01 |
| AD | Pars opercularis | rs11257238 | C | T | 0.01294 | 0.00226 | 1.04E-08 | 0.2926 | 1.435 | 8.38E-01 |
| AD | Pars opercularis | rs113260531 | A | G | 0.01999 | 0.00325 | 7.91E-10 | -0.0094 | 2.0217 | 9.96E-01 |
| AD | Pars opercularis | rs118170342 | C | T | 0.14754 | 0.00570 | 7.93E-148 | -5.0385 | 4.0888 | 2.18E-01 |
| AD | Pars opercularis | rs12590654 | A | G | -0.01483 | 0.00231 | 1.32E-10 | -2.6734 | 1.4422 | 6.38E-02 |
| AD | Pars opercularis | rs1859788 | A | G | -0.01840 | 0.00231 | 1.80E-15 | 0.5228 | 1.4904 | 7.26E-01 |
| AD | Pars opercularis | rs204473 | A | G | -0.04167 | 0.00700 | 2.58E-09 | 3.6257 | 4.3741 | 4.07E-01 |
| AD | Pars opercularis | rs2081545 | A | C | -0.01787 | 0.00223 | 1.11E-15 | 2.3918 | 1.3569 | 7.80E-02 |
| AD | Pars opercularis | rs28394864 | A | G | 0.01230 | 0.00218 | 1.68E-08 | 1.6193 | 1.3377 | 2.26E-01 |
| AD | Pars opercularis | rs28399657 | G | A | -0.05464 | 0.00658 | 9.82E-17 | -0.0418 | 4.039 | 9.92E-01 |
| AD | Pars opercularis | rs41289512 | G | C | 0.20630 | 0.00578 | 1.00E-200 | 0.1694 | 4.0155 | 9.66E-01 |
| AD | Pars opercularis | rs41290120 | A | G | -0.09905 | 0.00578 | 7.14E-66 | -0.9433 | 3.4134 | 7.82E-01 |
| AD | Pars opercularis | rs4236673 | A | G | -0.02016 | 0.00223 | 1.48E-19 | 1.5401 | 1.37 | 2.61E-01 |
| AD | Pars opercularis | rs442495 | C | T | -0.01372 | 0.00226 | 1.22E-09 | 1.5029 | 1.4284 | 2.93E-01 |
| AD | Pars opercularis | rs4575098 | A | G | 0.01641 | 0.00258 | 1.90E-10 | 0.9389 | 1.5695 | 5.50E-01 |
| AD | Pars opercularis | rs4663105 | C | A | 0.03110 | 0.00222 | 1.45E-44 | -1.5888 | 1.4 | 2.56E-01 |
| AD | Pars opercularis | rs59735493 | A | G | -0.01299 | 0.00236 | 3.73E-08 | -0.7308 | 1.4618 | 6.17E-01 |
| AD | Pars opercularis | rs6014724 | G | A | -0.02289 | 0.00369 | 5.38E-10 | 2.1226 | 2.4041 | 3.77E-01 |
| AD | Pars opercularis | rs6448453 | A | G | 0.01470 | 0.00245 | 1.98E-09 | 0.7394 | 1.5025 | 6.23E-01 |
| AD | Pars opercularis | rs679515 | T | C | 0.02542 | 0.00286 | 6.83E-19 | 0.9487 | 1.7322 | 5.84E-01 |
| AD | Pars opercularis | rs755951 | C | A | 0.01500 | 0.00221 | 1.13E-11 | 0.118 | 1.3563 | 9.31E-01 |
| AD | Pars opercularis | rs7810606 | T | C | -0.01452 | 0.00218 | 2.89E-11 | 1.317 | 1.3645 | 3.34E-01 |
| AD | Pars opercularis | rs846881 | C | A | -0.01737 | 0.00269 | 9.89E-11 | -3.1921 | 1.8276 | 8.07E-02 |
| AD | Pars opercularis | rs867611 | G | A | -0.02043 | 0.00232 | 1.48E-18 | 0.6944 | 1.4244 | 6.26E-01 |
| AD | Pars opercularis | rs9381563 | C | T | 0.01445 | 0.00227 | 1.99E-10 | -0.987 | 1.3968 | 4.80E-01 |
| AD | Pars orbitalis | rs10933431 | G | C | -0.01544 | 0.00251 | 7.62E-10 | 0.611 | 0.6184 | 3.23E-01 |
| AD | Pars orbitalis | rs111278892 | G | C | 0.01991 | 0.00305 | 6.67E-11 | -0.4005 | 0.7196 | 5.78E-01 |
| AD | Pars orbitalis | rs11218343 | C | T | -0.03593 | 0.00526 | 8.12E-12 | -0.549 | 1.1981 | 6.47E-01 |
| AD | Pars orbitalis | rs11257238 | C | T | 0.01294 | 0.00226 | 1.04E-08 | -0.1466 | 0.5053 | 7.72E-01 |
| AD | Pars orbitalis | rs113260531 | A | G | 0.01999 | 0.00325 | 7.91E-10 | -0.0133 | 0.7101 | 9.85E-01 |
| AD | Pars orbitalis | rs118170342 | C | T | 0.14754 | 0.00570 | 7.93E-148 | 0.0264 | 1.4474 | 9.85E-01 |
| AD | Pars orbitalis | rs12590654 | A | G | -0.01483 | 0.00231 | 1.32E-10 | 0.0203 | 0.507 | 9.68E-01 |
| AD | Pars orbitalis | rs1859788 | A | G | -0.01840 | 0.00231 | 1.80E-15 | -1.3306 | 0.5239 | 1.11E-02 |
| AD | Pars orbitalis | rs204473 | A | G | -0.04167 | 0.00700 | 2.58E-09 | -2.9975 | 1.5343 | 5.08E-02 |
| AD | Pars orbitalis | rs2081545 | A | C | -0.01787 | 0.00223 | 1.11E-15 | 0.1221 | 0.4777 | 7.98E-01 |
| AD | Pars orbitalis | rs28394864 | A | G | 0.01230 | 0.00218 | 1.68E-08 | -0.6289 | 0.47 | 1.81E-01 |
| AD | Pars orbitalis | rs28399657 | G | A | -0.05464 | 0.00658 | 9.82E-17 | 1.8227 | 1.4155 | 1.98E-01 |
| AD | Pars orbitalis | rs41289512 | G | C | 0.20630 | 0.00578 | 1.00E-200 | -0.9472 | 1.4213 | 5.05E-01 |
| AD | Pars orbitalis | rs41290120 | A | G | -0.09905 | 0.00578 | 7.14E-66 | -1.9952 | 1.202 | 9.69E-02 |
| AD | Pars orbitalis | rs4236673 | A | G | -0.02016 | 0.00223 | 1.48E-19 | -0.4814 | 0.4816 | 3.18E-01 |
| AD | Pars orbitalis | rs442495 | C | T | -0.01372 | 0.00226 | 1.22E-09 | -0.1998 | 0.503 | 6.91E-01 |
| AD | Pars orbitalis | rs4575098 | A | G | 0.01641 | 0.00258 | 1.90E-10 | -0.3394 | 0.5515 | 5.38E-01 |
| AD | Pars orbitalis | rs4663105 | C | A | 0.03110 | 0.00222 | 1.45E-44 | -0.2011 | 0.4927 | 6.83E-01 |
| AD | Pars orbitalis | rs59735493 | A | G | -0.01299 | 0.00236 | 3.73E-08 | -0.715 | 0.5141 | 1.64E-01 |
| AD | Pars orbitalis | rs6014724 | G | A | -0.02289 | 0.00369 | 5.38E-10 | -0.032 | 0.8469 | 9.70E-01 |
| AD | Pars orbitalis | rs6448453 | A | G | 0.01470 | 0.00245 | 1.98E-09 | -0.5318 | 0.5271 | 3.13E-01 |
| AD | Pars orbitalis | rs679515 | T | C | 0.02542 | 0.00286 | 6.83E-19 | -0.2009 | 0.6064 | 7.40E-01 |
| AD | Pars orbitalis | rs755951 | C | A | 0.01500 | 0.00221 | 1.13E-11 | 0.7059 | 0.4767 | 1.39E-01 |
| AD | Pars orbitalis | rs7810606 | T | C | -0.01452 | 0.00218 | 2.89E-11 | 0.2252 | 0.4798 | 6.39E-01 |
| AD | Pars orbitalis | rs846881 | C | A | -0.01737 | 0.00269 | 9.89E-11 | -0.1318 | 0.6426 | 8.38E-01 |
| AD | Pars orbitalis | rs867611 | G | A | -0.02043 | 0.00232 | 1.48E-18 | 0.4637 | 0.5003 | 3.54E-01 |
| AD | Pars orbitalis | rs9381563 | C | T | 0.01445 | 0.00227 | 1.99E-10 | 0.3096 | 0.4921 | 5.29E-01 |
| AD | Pars triangularis | rs10933431 | G | C | -0.01544 | 0.00251 | 7.62E-10 | 0.8579 | 1.6235 | 5.97E-01 |
| AD | Pars triangularis | rs111278892 | G | C | 0.01991 | 0.00305 | 6.67E-11 | -1.7489 | 1.8913 | 3.55E-01 |
| AD | Pars triangularis | rs11218343 | C | T | -0.03593 | 0.00526 | 8.12E-12 | -3.2007 | 3.1653 | 3.12E-01 |
| AD | Pars triangularis | rs11257238 | C | T | 0.01294 | 0.00226 | 1.04E-08 | 0.4775 | 1.3349 | 7.21E-01 |
| AD | Pars triangularis | rs113260531 | A | G | 0.01999 | 0.00325 | 7.91E-10 | 0.6197 | 1.8758 | 7.41E-01 |
| AD | Pars triangularis | rs118170342 | C | T | 0.14754 | 0.00570 | 7.93E-148 | 2.4061 | 3.7989 | 5.27E-01 |
| AD | Pars triangularis | rs12590654 | A | G | -0.01483 | 0.00231 | 1.32E-10 | -1.7807 | 1.3386 | 1.83E-01 |
| AD | Pars triangularis | rs1859788 | A | G | -0.01840 | 0.00231 | 1.80E-15 | 0.77 | 1.3826 | 5.78E-01 |
| AD | Pars triangularis | rs204473 | A | G | -0.04167 | 0.00700 | 2.58E-09 | -3.8585 | 4.0561 | 3.42E-01 |
| AD | Pars triangularis | rs2081545 | A | C | -0.01787 | 0.00223 | 1.11E-15 | 1.1424 | 1.2617 | 3.65E-01 |
| AD | Pars triangularis | rs28394864 | A | G | 0.01230 | 0.00218 | 1.68E-08 | -0.7866 | 1.2419 | 5.27E-01 |
| AD | Pars triangularis | rs28399657 | G | A | -0.05464 | 0.00658 | 9.82E-17 | 0.4944 | 3.7367 | 8.95E-01 |
| AD | Pars triangularis | rs41289512 | G | C | 0.20630 | 0.00578 | 1.00E-200 | 2.3233 | 3.7233 | 5.33E-01 |
| AD | Pars triangularis | rs41290120 | A | G | -0.09905 | 0.00578 | 7.14E-66 | -1.6685 | 3.1627 | 5.98E-01 |
| AD | Pars triangularis | rs4236673 | A | G | -0.02016 | 0.00223 | 1.48E-19 | 1.259 | 1.2727 | 3.23E-01 |
| AD | Pars triangularis | rs442495 | C | T | -0.01372 | 0.00226 | 1.22E-09 | 1.1006 | 1.3296 | 4.08E-01 |
| AD | Pars triangularis | rs4575098 | A | G | 0.01641 | 0.00258 | 1.90E-10 | 1.2467 | 1.4582 | 3.93E-01 |
| AD | Pars triangularis | rs4663105 | C | A | 0.03110 | 0.00222 | 1.45E-44 | -2.0224 | 1.2992 | 1.20E-01 |
| AD | Pars triangularis | rs59735493 | A | G | -0.01299 | 0.00236 | 3.73E-08 | 1.1505 | 1.3579 | 3.97E-01 |
| AD | Pars triangularis | rs6014724 | G | A | -0.02289 | 0.00369 | 5.38E-10 | 1.1557 | 2.2368 | 6.05E-01 |
| AD | Pars triangularis | rs6448453 | A | G | 0.01470 | 0.00245 | 1.98E-09 | -0.3836 | 1.3942 | 7.83E-01 |
| AD | Pars triangularis | rs679515 | T | C | 0.02542 | 0.00286 | 6.83E-19 | -2.3438 | 1.6034 | 1.44E-01 |
| AD | Pars triangularis | rs755951 | C | A | 0.01500 | 0.00221 | 1.13E-11 | 0.4652 | 1.261 | 7.12E-01 |
| AD | Pars triangularis | rs7810606 | T | C | -0.01452 | 0.00218 | 2.89E-11 | 0.8499 | 1.2674 | 5.03E-01 |
| AD | Pars triangularis | rs846881 | C | A | -0.01737 | 0.00269 | 9.89E-11 | -4.2645 | 1.6932 | 1.18E-02 |
| AD | Pars triangularis | rs867611 | G | A | -0.02043 | 0.00232 | 1.48E-18 | 1.5589 | 1.3226 | 2.39E-01 |
| AD | Pars triangularis | rs9381563 | C | T | 0.01445 | 0.00227 | 1.99E-10 | -0.3908 | 1.2985 | 7.64E-01 |
| AD | precentral | rs10933431 | G | C | -0.01544 | 0.00251 | 7.62E-10 | -0.152 | 3.34 | 9.64E-01 |
| AD | precentral | rs111278892 | G | C | 0.01991 | 0.00305 | 6.67E-11 | -2.8524 | 3.892 | 4.64E-01 |
| AD | precentral | rs11218343 | C | T | -0.03593 | 0.00526 | 8.12E-12 | -4.7586 | 6.4556 | 4.61E-01 |
| AD | precentral | rs11257238 | C | T | 0.01294 | 0.00226 | 1.04E-08 | -1.5453 | 2.7332 | 5.72E-01 |
| AD | precentral | rs113260531 | A | G | 0.01999 | 0.00325 | 7.91E-10 | -1.5466 | 3.8505 | 6.88E-01 |
| AD | precentral | rs118170342 | C | T | 0.14754 | 0.00570 | 7.93E-148 | -13.5486 | 7.8523 | 8.45E-02 |
| AD | precentral | rs12590654 | A | G | -0.01483 | 0.00231 | 1.32E-10 | -0.8053 | 2.7494 | 7.70E-01 |
| AD | precentral | rs1859788 | A | G | -0.01840 | 0.00231 | 1.80E-15 | 3.9431 | 2.8375 | 1.65E-01 |
| AD | precentral | rs204473 | A | G | -0.04167 | 0.00700 | 2.58E-09 | -0.7163 | 8.3263 | 9.31E-01 |
| AD | precentral | rs2081545 | A | C | -0.01787 | 0.00223 | 1.11E-15 | 1.4747 | 2.5781 | 5.67E-01 |
| AD | precentral | rs28394864 | A | G | 0.01230 | 0.00218 | 1.68E-08 | 2.7863 | 2.5458 | 2.74E-01 |
| AD | precentral | rs28399657 | G | A | -0.05464 | 0.00658 | 9.82E-17 | 6.9403 | 7.6303 | 3.63E-01 |
| AD | precentral | rs41289512 | G | C | 0.20630 | 0.00578 | 1.00E-200 | -5.3502 | 7.7782 | 4.92E-01 |
| AD | precentral | rs41290120 | A | G | -0.09905 | 0.00578 | 7.14E-66 | 3.9904 | 6.4939 | 5.39E-01 |
| AD | precentral | rs4236673 | A | G | -0.02016 | 0.00223 | 1.48E-19 | 0.1223 | 2.6026 | 9.63E-01 |
| AD | precentral | rs442495 | C | T | -0.01372 | 0.00226 | 1.22E-09 | 4.8273 | 2.712 | 7.51E-02 |
| AD | precentral | rs4575098 | A | G | 0.01641 | 0.00258 | 1.90E-10 | 0.6012 | 2.9759 | 8.40E-01 |
| AD | precentral | rs4663105 | C | A | 0.03110 | 0.00222 | 1.45E-44 | -6.0803 | 2.6638 | 2.25E-02 |
| AD | precentral | rs59735493 | A | G | -0.01299 | 0.00236 | 3.73E-08 | 3.436 | 2.7772 | 2.16E-01 |
| AD | precentral | rs6014724 | G | A | -0.02289 | 0.00369 | 5.38E-10 | -0.4718 | 4.5791 | 9.18E-01 |
| AD | precentral | rs6448453 | A | G | 0.01470 | 0.00245 | 1.98E-09 | 1.1508 | 2.8554 | 6.87E-01 |
| AD | precentral | rs679515 | T | C | 0.02542 | 0.00286 | 6.83E-19 | 0.8576 | 3.2972 | 7.95E-01 |
| AD | precentral | rs755951 | C | A | 0.01500 | 0.00221 | 1.13E-11 | 1.8295 | 2.5783 | 4.78E-01 |
| AD | precentral | rs7810606 | T | C | -0.01452 | 0.00218 | 2.89E-11 | -1.9262 | 2.6 | 4.59E-01 |
| AD | precentral | rs846881 | C | A | -0.01737 | 0.00269 | 9.89E-11 | -6.3763 | 3.5004 | 6.85E-02 |
| AD | precentral | rs867611 | G | A | -0.02043 | 0.00232 | 1.48E-18 | 1.1563 | 2.712 | 6.70E-01 |
| AD | precentral | rs9381563 | C | T | 0.01445 | 0.00227 | 1.99E-10 | 1.4767 | 2.6596 | 5.79E-01 |
| AD | Rostral anterior cingulate | rs10933431 | G | C | -0.01544 | 0.00251 | 7.62E-10 | 0.9149 | 0.9669 | 3.44E-01 |
| AD | Rostral anterior cingulate | rs111278892 | G | C | 0.01991 | 0.00305 | 6.67E-11 | -1.743 | 1.1298 | 1.23E-01 |
| AD | Rostral anterior cingulate | rs11218343 | C | T | -0.03593 | 0.00526 | 8.12E-12 | 1.358 | 1.8716 | 4.68E-01 |
| AD | Rostral anterior cingulate | rs11257238 | C | T | 0.01294 | 0.00226 | 1.04E-08 | -0.7465 | 0.7907 | 3.45E-01 |
| AD | Rostral anterior cingulate | rs113260531 | A | G | 0.01999 | 0.00325 | 7.91E-10 | -2.0413 | 1.1135 | 6.68E-02 |
| AD | Rostral anterior cingulate | rs118170342 | C | T | 0.14754 | 0.00570 | 7.93E-148 | -0.3763 | 2.2853 | 8.69E-01 |
| AD | Rostral anterior cingulate | rs12590654 | A | G | -0.01483 | 0.00231 | 1.32E-10 | -0.3854 | 0.794 | 6.27E-01 |
| AD | Rostral anterior cingulate | rs1859788 | A | G | -0.01840 | 0.00231 | 1.80E-15 | 0.8218 | 0.8224 | 3.18E-01 |
| AD | Rostral anterior cingulate | rs204473 | A | G | -0.04167 | 0.00700 | 2.58E-09 | -1.19 | 2.4207 | 6.23E-01 |
| AD | Rostral anterior cingulate | rs2081545 | A | C | -0.01787 | 0.00223 | 1.11E-15 | 0.9962 | 0.747 | 1.82E-01 |
| AD | Rostral anterior cingulate | rs28394864 | A | G | 0.01230 | 0.00218 | 1.68E-08 | 1.8141 | 0.7365 | 1.38E-02 |
| AD | Rostral anterior cingulate | rs28399657 | G | A | -0.05464 | 0.00658 | 9.82E-17 | 0.193 | 2.219 | 9.31E-01 |
| AD | Rostral anterior cingulate | rs41289512 | G | C | 0.20630 | 0.00578 | 1.00E-200 | -3.9927 | 2.2401 | 7.47E-02 |
| AD | Rostral anterior cingulate | rs41290120 | A | G | -0.09905 | 0.00578 | 7.14E-66 | 2.1601 | 1.8832 | 2.51E-01 |
| AD | Rostral anterior cingulate | rs4236673 | A | G | -0.02016 | 0.00223 | 1.48E-19 | 0.2718 | 0.7541 | 7.19E-01 |
| AD | Rostral anterior cingulate | rs442495 | C | T | -0.01372 | 0.00226 | 1.22E-09 | -0.3139 | 0.7852 | 6.89E-01 |
| AD | Rostral anterior cingulate | rs4575098 | A | G | 0.01641 | 0.00258 | 1.90E-10 | 0.535 | 0.8624 | 5.35E-01 |
| AD | Rostral anterior cingulate | rs4663105 | C | A | 0.03110 | 0.00222 | 1.45E-44 | -0.2889 | 0.7717 | 7.08E-01 |
| AD | Rostral anterior cingulate | rs59735493 | A | G | -0.01299 | 0.00236 | 3.73E-08 | -0.4791 | 0.8038 | 5.51E-01 |
| AD | Rostral anterior cingulate | rs6014724 | G | A | -0.02289 | 0.00369 | 5.38E-10 | -0.6238 | 1.3229 | 6.37E-01 |
| AD | Rostral anterior cingulate | rs6448453 | A | G | 0.01470 | 0.00245 | 1.98E-09 | 1.1784 | 0.8243 | 1.53E-01 |
| AD | Rostral anterior cingulate | rs679515 | T | C | 0.02542 | 0.00286 | 6.83E-19 | -0.4206 | 0.9507 | 6.58E-01 |
| AD | Rostral anterior cingulate | rs755951 | C | A | 0.01500 | 0.00221 | 1.13E-11 | -1.4359 | 0.7457 | 5.41E-02 |
| AD | Rostral anterior cingulate | rs7810606 | T | C | -0.01452 | 0.00218 | 2.89E-11 | 0.1266 | 0.7507 | 8.66E-01 |
| AD | Rostral anterior cingulate | rs846881 | C | A | -0.01737 | 0.00269 | 9.89E-11 | -0.7212 | 1.0095 | 4.75E-01 |
| AD | Rostral anterior cingulate | rs867611 | G | A | -0.02043 | 0.00232 | 1.48E-18 | -1.2407 | 0.7834 | 1.13E-01 |
| AD | Rostral anterior cingulate | rs9381563 | C | T | 0.01445 | 0.00227 | 1.99E-10 | -0.2418 | 0.7698 | 7.53E-01 |
| AD | Rostral middle frontal | rs10933431 | G | C | -0.01544 | 0.00251 | 7.62E-10 | 4.2175 | 3.9754 | 2.89E-01 |
| AD | Rostral middle frontal | rs111278892 | G | C | 0.01991 | 0.00305 | 6.67E-11 | -0.7271 | 4.65 | 8.76E-01 |
| AD | Rostral middle frontal | rs11218343 | C | T | -0.03593 | 0.00526 | 8.12E-12 | 0.9493 | 7.7596 | 9.03E-01 |
| AD | Rostral middle frontal | rs11257238 | C | T | 0.01294 | 0.00226 | 1.04E-08 | -0.6286 | 3.2795 | 8.48E-01 |
| AD | Rostral middle frontal | rs113260531 | A | G | 0.01999 | 0.00325 | 7.91E-10 | -2.6864 | 4.6075 | 5.60E-01 |
| AD | Rostral middle frontal | rs118170342 | C | T | 0.14754 | 0.00570 | 7.93E-148 | -10.542 | 9.2933 | 2.57E-01 |
| AD | Rostral middle frontal | rs12590654 | A | G | -0.01483 | 0.00231 | 1.32E-10 | 6.2463 | 3.2965 | 5.81E-02 |
| AD | Rostral middle frontal | rs1859788 | A | G | -0.01840 | 0.00231 | 1.80E-15 | -2.6855 | 3.3998 | 4.30E-01 |
| AD | Rostral middle frontal | rs204473 | A | G | -0.04167 | 0.00700 | 2.58E-09 | -5.7795 | 9.9503 | 5.61E-01 |
| AD | Rostral middle frontal | rs2081545 | A | C | -0.01787 | 0.00223 | 1.11E-15 | -4.9075 | 3.1005 | 1.14E-01 |
| AD | Rostral middle frontal | rs28394864 | A | G | 0.01230 | 0.00218 | 1.68E-08 | -5.3551 | 3.0554 | 7.97E-02 |
| AD | Rostral middle frontal | rs28399657 | G | A | -0.05464 | 0.00658 | 9.82E-17 | 6.3592 | 9.1963 | 4.89E-01 |
| AD | Rostral middle frontal | rs41289512 | G | C | 0.20630 | 0.00578 | 1.00E-200 | 2.9869 | 9.1367 | 7.44E-01 |
| AD | Rostral middle frontal | rs41290120 | A | G | -0.09905 | 0.00578 | 7.14E-66 | 6.5452 | 7.813 | 4.02E-01 |
| AD | Rostral middle frontal | rs4236673 | A | G | -0.02016 | 0.00223 | 1.48E-19 | -1.5569 | 3.1255 | 6.18E-01 |
| AD | Rostral middle frontal | rs442495 | C | T | -0.01372 | 0.00226 | 1.22E-09 | 2.7972 | 3.258 | 3.91E-01 |
| AD | Rostral middle frontal | rs4575098 | A | G | 0.01641 | 0.00258 | 1.90E-10 | -3.3896 | 3.5879 | 3.45E-01 |
| AD | Rostral middle frontal | rs4663105 | C | A | 0.03110 | 0.00222 | 1.45E-44 | 1.2264 | 3.1941 | 7.01E-01 |
| AD | Rostral middle frontal | rs59735493 | A | G | -0.01299 | 0.00236 | 3.73E-08 | -0.1568 | 3.3361 | 9.63E-01 |
| AD | Rostral middle frontal | rs6014724 | G | A | -0.02289 | 0.00369 | 5.38E-10 | 4.5277 | 5.4909 | 4.10E-01 |
| AD | Rostral middle frontal | rs6448453 | A | G | 0.01470 | 0.00245 | 1.98E-09 | 2.0699 | 3.4232 | 5.45E-01 |
| AD | Rostral middle frontal | rs679515 | T | C | 0.02542 | 0.00286 | 6.83E-19 | -0.5414 | 3.9519 | 8.91E-01 |
| AD | Rostral middle frontal | rs755951 | C | A | 0.01500 | 0.00221 | 1.13E-11 | -3.0139 | 3.0968 | 3.30E-01 |
| AD | Rostral middle frontal | rs7810606 | T | C | -0.01452 | 0.00218 | 2.89E-11 | -3.4576 | 3.1137 | 2.67E-01 |
| AD | Rostral middle frontal | rs846881 | C | A | -0.01737 | 0.00269 | 9.89E-11 | -3.1222 | 4.1605 | 4.53E-01 |
| AD | Rostral middle frontal | rs867611 | G | A | -0.02043 | 0.00232 | 1.48E-18 | 1.8542 | 3.2535 | 5.69E-01 |
| AD | Rostral middle frontal | rs9381563 | C | T | 0.01445 | 0.00227 | 1.99E-10 | 1.7129 | 3.1916 | 5.92E-01 |
| AD | Superior frontal | rs10933431 | G | C | -0.01544 | 0.00251 | 7.62E-10 | 2.8821 | 4.0337 | 4.75E-01 |
| AD | Superior frontal | rs111278892 | G | C | 0.01991 | 0.00305 | 6.67E-11 | 1.9996 | 4.701 | 6.71E-01 |
| AD | Superior frontal | rs11218343 | C | T | -0.03593 | 0.00526 | 8.12E-12 | 6.954 | 7.7535 | 3.70E-01 |
| AD | Superior frontal | rs11257238 | C | T | 0.01294 | 0.00226 | 1.04E-08 | -2.4446 | 3.2949 | 4.58E-01 |
| AD | Superior frontal | rs113260531 | A | G | 0.01999 | 0.00325 | 7.91E-10 | -2.1504 | 4.6153 | 6.41E-01 |
| AD | Superior frontal | rs118170342 | C | T | 0.14754 | 0.00570 | 7.93E-148 | -1.8965 | 9.4533 | 8.41E-01 |
| AD | Superior frontal | rs12590654 | A | G | -0.01483 | 0.00231 | 1.32E-10 | 2.4092 | 3.3032 | 4.66E-01 |
| AD | Superior frontal | rs1859788 | A | G | -0.01840 | 0.00231 | 1.80E-15 | 1.6345 | 3.4068 | 6.31E-01 |
| AD | Superior frontal | rs204473 | A | G | -0.04167 | 0.00700 | 2.58E-09 | 4.6254 | 9.9824 | 6.43E-01 |
| AD | Superior frontal | rs2081545 | A | C | -0.01787 | 0.00223 | 1.11E-15 | 5.4815 | 3.1053 | 7.75E-02 |
| AD | Superior frontal | rs28394864 | A | G | 0.01230 | 0.00218 | 1.68E-08 | 0.3485 | 3.0589 | 9.09E-01 |
| AD | Superior frontal | rs28399657 | G | A | -0.05464 | 0.00658 | 9.82E-17 | 2.8062 | 9.2392 | 7.61E-01 |
| AD | Superior frontal | rs41289512 | G | C | 0.20630 | 0.00578 | 1.00E-200 | 14.5562 | 9.2959 | 1.17E-01 |
| AD | Superior frontal | rs41290120 | A | G | -0.09905 | 0.00578 | 7.14E-66 | 9.5296 | 7.8621 | 2.26E-01 |
| AD | Superior frontal | rs4236673 | A | G | -0.02016 | 0.00223 | 1.48E-19 | -2.1374 | 3.1269 | 4.94E-01 |
| AD | Superior frontal | rs442495 | C | T | -0.01372 | 0.00226 | 1.22E-09 | 1.2695 | 3.2687 | 6.98E-01 |
| AD | Superior frontal | rs4575098 | A | G | 0.01641 | 0.00258 | 1.90E-10 | -0.4196 | 3.5849 | 9.07E-01 |
| AD | Superior frontal | rs4663105 | C | A | 0.03110 | 0.00222 | 1.45E-44 | 5.4496 | 3.1987 | 8.84E-02 |
| AD | Superior frontal | rs59735493 | A | G | -0.01299 | 0.00236 | 3.73E-08 | 5.3566 | 3.3416 | 1.09E-01 |
| AD | Superior frontal | rs6014724 | G | A | -0.02289 | 0.00369 | 5.38E-10 | -4.4319 | 5.5049 | 4.21E-01 |
| AD | Superior frontal | rs6448453 | A | G | 0.01470 | 0.00245 | 1.98E-09 | 2.3138 | 3.4271 | 5.00E-01 |
| AD | Superior frontal | rs679515 | T | C | 0.02542 | 0.00286 | 6.83E-19 | -12.2766 | 3.9494 | 1.88E-03 |
| AD | Superior frontal | rs755951 | C | A | 0.01500 | 0.00221 | 1.13E-11 | 4.2475 | 3.1008 | 1.71E-01 |
| AD | Superior frontal | rs7810606 | T | C | -0.01452 | 0.00218 | 2.89E-11 | -2.2343 | 3.1202 | 4.74E-01 |
| AD | Superior frontal | rs846881 | C | A | -0.01737 | 0.00269 | 9.89E-11 | 1.3041 | 4.1918 | 7.56E-01 |
| AD | Superior frontal | rs867611 | G | A | -0.02043 | 0.00232 | 1.48E-18 | 0.236 | 3.2568 | 9.42E-01 |
| AD | Superior frontal | rs9381563 | C | T | 0.01445 | 0.00227 | 1.99E-10 | 1.3725 | 3.1976 | 6.68E-01 |
| AD | banks of the superior temporal sulcus | rs10933431 | G | C | -0.01544 | 0.00251 | 7.62E-10 | 0.1163 | 1.1181 | 9.17E-01 |
| AD | banks of the superior temporal sulcus | rs111278892 | G | C | 0.01991 | 0.00305 | 6.67E-11 | 2.4729 | 1.3084 | 5.88E-02 |
| AD | banks of the superior temporal sulcus | rs11218343 | C | T | -0.03593 | 0.00526 | 8.12E-12 | 0.4257 | 2.1738 | 8.45E-01 |
| AD | banks of the superior temporal sulcus | rs11257238 | C | T | 0.01294 | 0.00226 | 1.04E-08 | -0.3586 | 0.9156 | 6.95E-01 |
| AD | banks of the superior temporal sulcus | rs113260531 | A | G | 0.01999 | 0.00325 | 7.91E-10 | 1.5118 | 1.2883 | 2.41E-01 |
| AD | banks of the superior temporal sulcus | rs118170342 | C | T | 0.14754 | 0.00570 | 7.93E-148 | 1.0926 | 2.657 | 6.81E-01 |
| AD | banks of the superior temporal sulcus | rs12590654 | A | G | -0.01483 | 0.00231 | 1.32E-10 | 1.6586 | 0.9192 | 7.12E-02 |
| AD | banks of the superior temporal sulcus | rs1859788 | A | G | -0.01840 | 0.00231 | 1.80E-15 | -0.8791 | 0.9512 | 3.55E-01 |
| AD | banks of the superior temporal sulcus | rs204473 | A | G | -0.04167 | 0.00700 | 2.58E-09 | -1.1736 | 2.7957 | 6.75E-01 |
| AD | banks of the superior temporal sulcus | rs2081545 | A | C | -0.01787 | 0.00223 | 1.11E-15 | -0.263 | 0.8629 | 7.61E-01 |
| AD | banks of the superior temporal sulcus | rs28394864 | A | G | 0.01230 | 0.00218 | 1.68E-08 | 1.5782 | 0.8504 | 6.35E-02 |
| AD | banks of the superior temporal sulcus | rs28399657 | G | A | -0.05464 | 0.00658 | 9.82E-17 | 1.3619 | 2.5867 | 5.99E-01 |
| AD | banks of the superior temporal sulcus | rs41289512 | G | C | 0.20630 | 0.00578 | 1.00E-200 | 0.5197 | 2.5842 | 8.41E-01 |
| AD | banks of the superior temporal sulcus | rs41290120 | A | G | -0.09905 | 0.00578 | 7.14E-66 | 1.0567 | 2.1793 | 6.28E-01 |
| AD | banks of the superior temporal sulcus | rs4236673 | A | G | -0.02016 | 0.00223 | 1.48E-19 | -0.845 | 0.8734 | 3.33E-01 |
| AD | banks of the superior temporal sulcus | rs442495 | C | T | -0.01372 | 0.00226 | 1.22E-09 | 1.9221 | 0.9091 | 3.45E-02 |
| AD | banks of the superior temporal sulcus | rs4575098 | A | G | 0.01641 | 0.00258 | 1.90E-10 | -2.2857 | 0.9987 | 2.21E-02 |
| AD | banks of the superior temporal sulcus | rs4663105 | C | A | 0.03110 | 0.00222 | 1.45E-44 | 0.2966 | 0.892 | 7.40E-01 |
| AD | banks of the superior temporal sulcus | rs59735493 | A | G | -0.01299 | 0.00236 | 3.73E-08 | 0.27 | 0.9297 | 7.72E-01 |
| AD | banks of the superior temporal sulcus | rs6014724 | G | A | -0.02289 | 0.00369 | 5.38E-10 | -0.4144 | 1.5333 | 7.87E-01 |
| AD | banks of the superior temporal sulcus | rs6448453 | A | G | 0.01470 | 0.00245 | 1.98E-09 | -0.7 | 0.9559 | 4.64E-01 |
| AD | banks of the superior temporal sulcus | rs679515 | T | C | 0.02542 | 0.00286 | 6.83E-19 | 0.2342 | 1.0993 | 8.31E-01 |
| AD | banks of the superior temporal sulcus | rs755951 | C | A | 0.01500 | 0.00221 | 1.13E-11 | 0.7462 | 0.8644 | 3.88E-01 |
| AD | banks of the superior temporal sulcus | rs7810606 | T | C | -0.01452 | 0.00218 | 2.89E-11 | 0.3463 | 0.8697 | 6.91E-01 |
| AD | banks of the superior temporal sulcus | rs846881 | C | A | -0.01737 | 0.00269 | 9.89E-11 | 0.4466 | 1.1698 | 7.03E-01 |
| AD | banks of the superior temporal sulcus | rs867611 | G | A | -0.02043 | 0.00232 | 1.48E-18 | 0.6911 | 0.9065 | 4.46E-01 |
| AD | banks of the superior temporal sulcus | rs9381563 | C | T | 0.01445 | 0.00227 | 1.99E-10 | -0.1571 | 0.8893 | 8.60E-01 |
| AD | entorhinal | rs10933431 | G | C | -0.01544 | 0.00251 | 7.62E-10 | -1 | 0.6268 | 1.11E-01 |
| AD | entorhinal | rs111278892 | G | C | 0.01991 | 0.00305 | 6.67E-11 | -0.2431 | 0.7327 | 7.40E-01 |
| AD | entorhinal | rs11218343 | C | T | -0.03593 | 0.00526 | 8.12E-12 | -0.3283 | 1.2256 | 7.89E-01 |
| AD | entorhinal | rs11257238 | C | T | 0.01294 | 0.00226 | 1.04E-08 | 0.4556 | 0.5178 | 3.79E-01 |
| AD | entorhinal | rs113260531 | A | G | 0.01999 | 0.00325 | 7.91E-10 | 0.6481 | 0.7286 | 3.74E-01 |
| AD | entorhinal | rs118170342 | C | T | 0.14754 | 0.00570 | 7.93E-148 | -0.6544 | 1.4524 | 6.52E-01 |
| AD | entorhinal | rs12590654 | A | G | -0.01483 | 0.00231 | 1.32E-10 | 0.1019 | 0.5185 | 8.44E-01 |
| AD | entorhinal | rs1859788 | A | G | -0.01840 | 0.00231 | 1.80E-15 | 0.1871 | 0.5344 | 7.26E-01 |
| AD | entorhinal | rs204473 | A | G | -0.04167 | 0.00700 | 2.58E-09 | -0.8677 | 1.5674 | 5.80E-01 |
| AD | entorhinal | rs2081545 | A | C | -0.01787 | 0.00223 | 1.11E-15 | -0.5335 | 0.488 | 2.74E-01 |
| AD | entorhinal | rs28394864 | A | G | 0.01230 | 0.00218 | 1.68E-08 | -0.6921 | 0.481 | 1.50E-01 |
| AD | entorhinal | rs28399657 | G | A | -0.05464 | 0.00658 | 9.82E-17 | 1.4313 | 1.4324 | 3.18E-01 |
| AD | entorhinal | rs41289512 | G | C | 0.20630 | 0.00578 | 1.00E-200 | -0.0957 | 1.4261 | 9.47E-01 |
| AD | entorhinal | rs41290120 | A | G | -0.09905 | 0.00578 | 7.14E-66 | 1.5908 | 1.2275 | 1.95E-01 |
| AD | entorhinal | rs4236673 | A | G | -0.02016 | 0.00223 | 1.48E-19 | 0.0754 | 0.4923 | 8.78E-01 |
| AD | entorhinal | rs442495 | C | T | -0.01372 | 0.00226 | 1.22E-09 | 0.2451 | 0.5132 | 6.33E-01 |
| AD | entorhinal | rs4575098 | A | G | 0.01641 | 0.00258 | 1.90E-10 | -0.6652 | 0.5648 | 2.39E-01 |
| AD | entorhinal | rs4663105 | C | A | 0.03110 | 0.00222 | 1.45E-44 | 0.2012 | 0.5024 | 6.89E-01 |
| AD | entorhinal | rs59735493 | A | G | -0.01299 | 0.00236 | 3.73E-08 | 0.8669 | 0.5257 | 9.91E-02 |
| AD | entorhinal | rs6014724 | G | A | -0.02289 | 0.00369 | 5.38E-10 | 1.2913 | 0.8631 | 1.35E-01 |
| AD | entorhinal | rs6448453 | A | G | 0.01470 | 0.00245 | 1.98E-09 | -0.3649 | 0.5412 | 5.00E-01 |
| AD | entorhinal | rs679515 | T | C | 0.02542 | 0.00286 | 6.83E-19 | 0.5021 | 0.6241 | 4.21E-01 |
| AD | entorhinal | rs755951 | C | A | 0.01500 | 0.00221 | 1.13E-11 | 0.6358 | 0.4885 | 1.93E-01 |
| AD | entorhinal | rs7810606 | T | C | -0.01452 | 0.00218 | 2.89E-11 | -0.5444 | 0.4891 | 2.66E-01 |
| AD | entorhinal | rs846881 | C | A | -0.01737 | 0.00269 | 9.89E-11 | -1.0755 | 0.6539 | 1.00E-01 |
| AD | entorhinal | rs867611 | G | A | -0.02043 | 0.00232 | 1.48E-18 | 0.1332 | 0.5129 | 7.95E-01 |
| AD | entorhinal | rs9381563 | C | T | 0.01445 | 0.00227 | 1.99E-10 | 0.0187 | 0.5021 | 9.70E-01 |
| AD | fusiform | rs10933431 | G | C | -0.01544 | 0.00251 | 7.62E-10 | -2.9934 | 2.5021 | 2.32E-01 |
| AD | fusiform | rs111278892 | G | C | 0.01991 | 0.00305 | 6.67E-11 | 6.1062 | 2.9243 | 3.68E-02 |
| AD | fusiform | rs11218343 | C | T | -0.03593 | 0.00526 | 8.12E-12 | -1.9258 | 4.8426 | 6.91E-01 |
| AD | fusiform | rs11257238 | C | T | 0.01294 | 0.00226 | 1.04E-08 | 1.1986 | 2.0507 | 5.59E-01 |
| AD | fusiform | rs113260531 | A | G | 0.01999 | 0.00325 | 7.91E-10 | 1.406 | 2.8797 | 6.25E-01 |
| AD | fusiform | rs118170342 | C | T | 0.14754 | 0.00570 | 7.93E-148 | 5.2403 | 5.8813 | 3.73E-01 |
| AD | fusiform | rs12590654 | A | G | -0.01483 | 0.00231 | 1.32E-10 | 1.7439 | 2.0552 | 3.96E-01 |
| AD | fusiform | rs1859788 | A | G | -0.01840 | 0.00231 | 1.80E-15 | -2.0021 | 2.1253 | 3.46E-01 |
| AD | fusiform | rs204473 | A | G | -0.04167 | 0.00700 | 2.58E-09 | 5.954 | 6.208 | 3.38E-01 |
| AD | fusiform | rs2081545 | A | C | -0.01787 | 0.00223 | 1.11E-15 | 0.2334 | 1.9321 | 9.04E-01 |
| AD | fusiform | rs28394864 | A | G | 0.01230 | 0.00218 | 1.68E-08 | -0.663 | 1.9038 | 7.28E-01 |
| AD | fusiform | rs28399657 | G | A | -0.05464 | 0.00658 | 9.82E-17 | 2.8686 | 5.775 | 6.19E-01 |
| AD | fusiform | rs41289512 | G | C | 0.20630 | 0.00578 | 1.00E-200 | -3.2085 | 5.8034 | 5.80E-01 |
| AD | fusiform | rs41290120 | A | G | -0.09905 | 0.00578 | 7.14E-66 | 1.1959 | 4.9027 | 8.07E-01 |
| AD | fusiform | rs4236673 | A | G | -0.02016 | 0.00223 | 1.48E-19 | 2.0244 | 1.9471 | 2.99E-01 |
| AD | fusiform | rs442495 | C | T | -0.01372 | 0.00226 | 1.22E-09 | 2.1743 | 2.0285 | 2.84E-01 |
| AD | fusiform | rs4575098 | A | G | 0.01641 | 0.00258 | 1.90E-10 | 0.0875 | 2.2319 | 9.69E-01 |
| AD | fusiform | rs4663105 | C | A | 0.03110 | 0.00222 | 1.45E-44 | -0.549 | 1.9952 | 7.83E-01 |
| AD | fusiform | rs59735493 | A | G | -0.01299 | 0.00236 | 3.73E-08 | 0.7282 | 2.0796 | 7.26E-01 |
| AD | fusiform | rs6014724 | G | A | -0.02289 | 0.00369 | 5.38E-10 | -5.1962 | 3.4298 | 1.30E-01 |
| AD | fusiform | rs6448453 | A | G | 0.01470 | 0.00245 | 1.98E-09 | -1.5992 | 2.1338 | 4.54E-01 |
| AD | fusiform | rs679515 | T | C | 0.02542 | 0.00286 | 6.83E-19 | 0.4174 | 2.4608 | 8.65E-01 |
| AD | fusiform | rs755951 | C | A | 0.01500 | 0.00221 | 1.13E-11 | 1.6113 | 1.9272 | 4.03E-01 |
| AD | fusiform | rs7810606 | T | C | -0.01452 | 0.00218 | 2.89E-11 | 1.4224 | 1.9414 | 4.64E-01 |
| AD | fusiform | rs846881 | C | A | -0.01737 | 0.00269 | 9.89E-11 | 1.595 | 2.6115 | 5.41E-01 |
| AD | fusiform | rs867611 | G | A | -0.02043 | 0.00232 | 1.48E-18 | 0.8639 | 2.027 | 6.70E-01 |
| AD | fusiform | rs9381563 | C | T | 0.01445 | 0.00227 | 1.99E-10 | -0.9638 | 1.99 | 6.28E-01 |
| AD | Inferior temporal | rs10933431 | G | C | -0.01544 | 0.00251 | 7.62E-10 | 3.5929 | 2.8524 | 2.08E-01 |
| AD | Inferior temporal | rs111278892 | G | C | 0.01991 | 0.00305 | 6.67E-11 | 0.9204 | 3.3127 | 7.81E-01 |
| AD | Inferior temporal | rs11218343 | C | T | -0.03593 | 0.00526 | 8.12E-12 | 9.8868 | 5.5226 | 7.34E-02 |
| AD | Inferior temporal | rs11257238 | C | T | 0.01294 | 0.00226 | 1.04E-08 | 0.9019 | 2.3282 | 6.99E-01 |
| AD | Inferior temporal | rs113260531 | A | G | 0.01999 | 0.00325 | 7.91E-10 | 2.7107 | 3.2656 | 4.07E-01 |
| AD | Inferior temporal | rs118170342 | C | T | 0.14754 | 0.00570 | 7.93E-148 | -1.9791 | 6.6598 | 7.66E-01 |
| AD | Inferior temporal | rs12590654 | A | G | -0.01483 | 0.00231 | 1.32E-10 | 0.5052 | 2.3355 | 8.29E-01 |
| AD | Inferior temporal | rs1859788 | A | G | -0.01840 | 0.00231 | 1.80E-15 | -6.8122 | 2.4151 | 4.79E-03 |
| AD | Inferior temporal | rs204473 | A | G | -0.04167 | 0.00700 | 2.58E-09 | 2.5788 | 7.0512 | 7.15E-01 |
| AD | Inferior temporal | rs2081545 | A | C | -0.01787 | 0.00223 | 1.11E-15 | -1.2548 | 2.1989 | 5.68E-01 |
| AD | Inferior temporal | rs28394864 | A | G | 0.01230 | 0.00218 | 1.68E-08 | -2.4971 | 2.1642 | 2.49E-01 |
| AD | Inferior temporal | rs28399657 | G | A | -0.05464 | 0.00658 | 9.82E-17 | 0.1563 | 6.5428 | 9.81E-01 |
| AD | Inferior temporal | rs41289512 | G | C | 0.20630 | 0.00578 | 1.00E-200 | -2.1509 | 6.5323 | 7.42E-01 |
| AD | Inferior temporal | rs41290120 | A | G | -0.09905 | 0.00578 | 7.14E-66 | -10.029 | 5.5498 | 7.08E-02 |
| AD | Inferior temporal | rs4236673 | A | G | -0.02016 | 0.00223 | 1.48E-19 | -1.5474 | 2.2156 | 4.85E-01 |
| AD | Inferior temporal | rs442495 | C | T | -0.01372 | 0.00226 | 1.22E-09 | 1.9147 | 2.3136 | 4.08E-01 |
| AD | Inferior temporal | rs4575098 | A | G | 0.01641 | 0.00258 | 1.90E-10 | -1.227 | 2.5376 | 6.29E-01 |
| AD | Inferior temporal | rs4663105 | C | A | 0.03110 | 0.00222 | 1.45E-44 | 3.9443 | 2.2657 | 8.17E-02 |
| AD | Inferior temporal | rs59735493 | A | G | -0.01299 | 0.00236 | 3.73E-08 | -2.6963 | 2.3644 | 2.54E-01 |
| AD | Inferior temporal | rs6014724 | G | A | -0.02289 | 0.00369 | 5.38E-10 | -6.2226 | 3.893 | 1.10E-01 |
| AD | Inferior temporal | rs6448453 | A | G | 0.01470 | 0.00245 | 1.98E-09 | -0.5654 | 2.4298 | 8.16E-01 |
| AD | Inferior temporal | rs679515 | T | C | 0.02542 | 0.00286 | 6.83E-19 | -0.8639 | 2.7952 | 7.57E-01 |
| AD | Inferior temporal | rs755951 | C | A | 0.01500 | 0.00221 | 1.13E-11 | 1.3127 | 2.1938 | 5.50E-01 |
| AD | Inferior temporal | rs7810606 | T | C | -0.01452 | 0.00218 | 2.89E-11 | 2.5046 | 2.2033 | 2.56E-01 |
| AD | Inferior temporal | rs846881 | C | A | -0.01737 | 0.00269 | 9.89E-11 | 0.8388 | 2.9595 | 7.77E-01 |
| AD | Inferior temporal | rs867611 | G | A | -0.02043 | 0.00232 | 1.48E-18 | -1.0284 | 2.3052 | 6.56E-01 |
| AD | Inferior temporal | rs9381563 | C | T | 0.01445 | 0.00227 | 1.99E-10 | 1.0638 | 2.2592 | 6.38E-01 |
| AD | insula | rs10933431 | G | C | -0.01544 | 0.00251 | 7.62E-10 | 4.1931 | 1.7036 | 1.38E-02 |
| AD | insula | rs111278892 | G | C | 0.01991 | 0.00305 | 6.67E-11 | -2.5575 | 1.971 | 1.94E-01 |
| AD | insula | rs11218343 | C | T | -0.03593 | 0.00526 | 8.12E-12 | -2.3739 | 3.2976 | 4.72E-01 |
| AD | insula | rs11257238 | C | T | 0.01294 | 0.00226 | 1.04E-08 | 1.205 | 1.3872 | 3.85E-01 |
| AD | insula | rs113260531 | A | G | 0.01999 | 0.00325 | 7.91E-10 | 0.4538 | 1.9573 | 8.17E-01 |
| AD | insula | rs118170342 | C | T | 0.14754 | 0.00570 | 7.93E-148 | 4.5665 | 3.9579 | 2.49E-01 |
| AD | insula | rs12590654 | A | G | -0.01483 | 0.00231 | 1.32E-10 | -1.3652 | 1.3982 | 3.29E-01 |
| AD | insula | rs1859788 | A | G | -0.01840 | 0.00231 | 1.80E-15 | 3.5744 | 1.4427 | 1.32E-02 |
| AD | insula | rs204473 | A | G | -0.04167 | 0.00700 | 2.58E-09 | 6.0968 | 4.2166 | 1.48E-01 |
| AD | insula | rs2081545 | A | C | -0.01787 | 0.00223 | 1.11E-15 | 0.5651 | 1.3174 | 6.68E-01 |
| AD | insula | rs28394864 | A | G | 0.01230 | 0.00218 | 1.68E-08 | -2.0763 | 1.2965 | 1.09E-01 |
| AD | insula | rs28399657 | G | A | -0.05464 | 0.00658 | 9.82E-17 | -1.4264 | 3.8735 | 7.13E-01 |
| AD | insula | rs41289512 | G | C | 0.20630 | 0.00578 | 1.00E-200 | 3.5745 | 3.893 | 3.59E-01 |
| AD | insula | rs41290120 | A | G | -0.09905 | 0.00578 | 7.14E-66 | -0.3946 | 3.3022 | 9.05E-01 |
| AD | insula | rs4236673 | A | G | -0.02016 | 0.00223 | 1.48E-19 | 0.0085 | 1.3266 | 9.95E-01 |
| AD | insula | rs442495 | C | T | -0.01372 | 0.00226 | 1.22E-09 | -1.4563 | 1.3881 | 2.94E-01 |
| AD | insula | rs4575098 | A | G | 0.01641 | 0.00258 | 1.90E-10 | 3.1171 | 1.5191 | 4.02E-02 |
| AD | insula | rs4663105 | C | A | 0.03110 | 0.00222 | 1.45E-44 | -0.4778 | 1.3547 | 7.24E-01 |
| AD | insula | rs59735493 | A | G | -0.01299 | 0.00236 | 3.73E-08 | 2.7669 | 1.4147 | 5.05E-02 |
| AD | insula | rs6014724 | G | A | -0.02289 | 0.00369 | 5.38E-10 | 3.4518 | 2.319 | 1.37E-01 |
| AD | insula | rs6448453 | A | G | 0.01470 | 0.00245 | 1.98E-09 | 1.2351 | 1.4557 | 3.96E-01 |
| AD | insula | rs679515 | T | C | 0.02542 | 0.00286 | 6.83E-19 | -3.3778 | 1.6743 | 4.37E-02 |
| AD | insula | rs755951 | C | A | 0.01500 | 0.00221 | 1.13E-11 | 1.6141 | 1.3157 | 2.20E-01 |
| AD | insula | rs7810606 | T | C | -0.01452 | 0.00218 | 2.89E-11 | 3.2828 | 1.3196 | 1.29E-02 |
| AD | insula | rs846881 | C | A | -0.01737 | 0.00269 | 9.89E-11 | 0.882 | 1.7627 | 6.17E-01 |
| AD | insula | rs867611 | G | A | -0.02043 | 0.00232 | 1.48E-18 | -2.2271 | 1.3812 | 1.07E-01 |
| AD | insula | rs9381563 | C | T | 0.01445 | 0.00227 | 1.99E-10 | -1.809 | 1.3569 | 1.83E-01 |
| AD | Middle temporal | rs10933431 | G | C | -0.01544 | 0.00251 | 7.62E-10 | -0.1904 | 2.4509 | 9.38E-01 |
| AD | Middle temporal | rs111278892 | G | C | 0.01991 | 0.00305 | 6.67E-11 | 2.103 | 2.8565 | 4.62E-01 |
| AD | Middle temporal | rs11218343 | C | T | -0.03593 | 0.00526 | 8.12E-12 | 2.8047 | 4.7418 | 5.54E-01 |
| AD | Middle temporal | rs11257238 | C | T | 0.01294 | 0.00226 | 1.04E-08 | 0.392 | 2.0038 | 8.45E-01 |
| AD | Middle temporal | rs113260531 | A | G | 0.01999 | 0.00325 | 7.91E-10 | 6.0835 | 2.814 | 3.06E-02 |
| AD | Middle temporal | rs118170342 | C | T | 0.14754 | 0.00570 | 7.93E-148 | 7.1374 | 5.7445 | 2.14E-01 |
| AD | Middle temporal | rs12590654 | A | G | -0.01483 | 0.00231 | 1.32E-10 | 0.6173 | 2.0176 | 7.60E-01 |
| AD | Middle temporal | rs1859788 | A | G | -0.01840 | 0.00231 | 1.80E-15 | -7.0585 | 2.0783 | 6.83E-04 |
| AD | Middle temporal | rs204473 | A | G | -0.04167 | 0.00700 | 2.58E-09 | 8.3444 | 6.0535 | 1.68E-01 |
| AD | Middle temporal | rs2081545 | A | C | -0.01787 | 0.00223 | 1.11E-15 | 0.5282 | 1.8925 | 7.80E-01 |
| AD | Middle temporal | rs28394864 | A | G | 0.01230 | 0.00218 | 1.68E-08 | 2.0291 | 1.8634 | 2.76E-01 |
| AD | Middle temporal | rs28399657 | G | A | -0.05464 | 0.00658 | 9.82E-17 | 1.9113 | 5.6535 | 7.35E-01 |
| AD | Middle temporal | rs41289512 | G | C | 0.20630 | 0.00578 | 1.00E-200 | -4.4194 | 5.6151 | 4.31E-01 |
| AD | Middle temporal | rs41290120 | A | G | -0.09905 | 0.00578 | 7.14E-66 | -1.5113 | 4.7897 | 7.52E-01 |
| AD | Middle temporal | rs4236673 | A | G | -0.02016 | 0.00223 | 1.48E-19 | -0.4804 | 1.9079 | 8.01E-01 |
| AD | Middle temporal | rs442495 | C | T | -0.01372 | 0.00226 | 1.22E-09 | 1.7109 | 1.9861 | 3.89E-01 |
| AD | Middle temporal | rs4575098 | A | G | 0.01641 | 0.00258 | 1.90E-10 | -5.5666 | 2.1854 | 1.09E-02 |
| AD | Middle temporal | rs4663105 | C | A | 0.03110 | 0.00222 | 1.45E-44 | 3.7145 | 1.952 | 5.71E-02 |
| AD | Middle temporal | rs59735493 | A | G | -0.01299 | 0.00236 | 3.73E-08 | -2.4653 | 2.0349 | 2.26E-01 |
| AD | Middle temporal | rs6014724 | G | A | -0.02289 | 0.00369 | 5.38E-10 | -1.4341 | 3.3506 | 6.69E-01 |
| AD | Middle temporal | rs6448453 | A | G | 0.01470 | 0.00245 | 1.98E-09 | -0.9722 | 2.0928 | 6.42E-01 |
| AD | Middle temporal | rs679515 | T | C | 0.02542 | 0.00286 | 6.83E-19 | -2.4738 | 2.4093 | 3.05E-01 |
| AD | Middle temporal | rs755951 | C | A | 0.01500 | 0.00221 | 1.13E-11 | -2.2823 | 1.8892 | 2.27E-01 |
| AD | Middle temporal | rs7810606 | T | C | -0.01452 | 0.00218 | 2.89E-11 | 4.3142 | 1.8989 | 2.31E-02 |
| AD | Middle temporal | rs846881 | C | A | -0.01737 | 0.00269 | 9.89E-11 | 3.1144 | 2.5502 | 2.22E-01 |
| AD | Middle temporal | rs867611 | G | A | -0.02043 | 0.00232 | 1.48E-18 | -1.8437 | 1.9853 | 3.53E-01 |
| AD | Middle temporal | rs9381563 | C | T | 0.01445 | 0.00227 | 1.99E-10 | 2.0761 | 1.9438 | 2.86E-01 |
| AD | parahippocampal | rs10933431 | G | C | -0.01544 | 0.00251 | 7.62E-10 | -0.6006 | 0.768 | 4.34E-01 |
| AD | parahippocampal | rs111278892 | G | C | 0.01991 | 0.00305 | 6.67E-11 | -0.462 | 0.897 | 6.07E-01 |
| AD | parahippocampal | rs11218343 | C | T | -0.03593 | 0.00526 | 8.12E-12 | 0.7045 | 1.4851 | 6.35E-01 |
| AD | parahippocampal | rs11257238 | C | T | 0.01294 | 0.00226 | 1.04E-08 | 0.8485 | 0.6321 | 1.80E-01 |
| AD | parahippocampal | rs113260531 | A | G | 0.01999 | 0.00325 | 7.91E-10 | 0.8238 | 0.8824 | 3.51E-01 |
| AD | parahippocampal | rs118170342 | C | T | 0.14754 | 0.00570 | 7.93E-148 | -0.8068 | 1.8288 | 6.59E-01 |
| AD | parahippocampal | rs12590654 | A | G | -0.01483 | 0.00231 | 1.32E-10 | 1.1817 | 0.631 | 6.11E-02 |
| AD | parahippocampal | rs1859788 | A | G | -0.01840 | 0.00231 | 1.80E-15 | -0.9919 | 0.6523 | 1.28E-01 |
| AD | parahippocampal | rs204473 | A | G | -0.04167 | 0.00700 | 2.58E-09 | 2.281 | 1.9277 | 2.37E-01 |
| AD | parahippocampal | rs2081545 | A | C | -0.01787 | 0.00223 | 1.11E-15 | -0.8649 | 0.5929 | 1.45E-01 |
| AD | parahippocampal | rs28394864 | A | G | 0.01230 | 0.00218 | 1.68E-08 | -0.393 | 0.5846 | 5.02E-01 |
| AD | parahippocampal | rs28399657 | G | A | -0.05464 | 0.00658 | 9.82E-17 | -0.5841 | 1.7791 | 7.43E-01 |
| AD | parahippocampal | rs41289512 | G | C | 0.20630 | 0.00578 | 1.00E-200 | 2.9427 | 1.7954 | 1.01E-01 |
| AD | parahippocampal | rs41290120 | A | G | -0.09905 | 0.00578 | 7.14E-66 | -2.8655 | 1.4963 | 5.55E-02 |
| AD | parahippocampal | rs4236673 | A | G | -0.02016 | 0.00223 | 1.48E-19 | -0.7341 | 0.5983 | 2.20E-01 |
| AD | parahippocampal | rs442495 | C | T | -0.01372 | 0.00226 | 1.22E-09 | -0.5313 | 0.6251 | 3.95E-01 |
| AD | parahippocampal | rs4575098 | A | G | 0.01641 | 0.00258 | 1.90E-10 | -0.5058 | 0.6867 | 4.61E-01 |
| AD | parahippocampal | rs4663105 | C | A | 0.03110 | 0.00222 | 1.45E-44 | -0.2094 | 0.6142 | 7.33E-01 |
| AD | parahippocampal | rs59735493 | A | G | -0.01299 | 0.00236 | 3.73E-08 | -1.0745 | 0.6383 | 9.23E-02 |
| AD | parahippocampal | rs6014724 | G | A | -0.02289 | 0.00369 | 5.38E-10 | -0.806 | 1.0434 | 4.40E-01 |
| AD | parahippocampal | rs6448453 | A | G | 0.01470 | 0.00245 | 1.98E-09 | 0.6395 | 0.6536 | 3.28E-01 |
| AD | parahippocampal | rs679515 | T | C | 0.02542 | 0.00286 | 6.83E-19 | 0.3001 | 0.7567 | 6.92E-01 |
| AD | parahippocampal | rs755951 | C | A | 0.01500 | 0.00221 | 1.13E-11 | 0.1405 | 0.5927 | 8.13E-01 |
| AD | parahippocampal | rs7810606 | T | C | -0.01452 | 0.00218 | 2.89E-11 | 0.3327 | 0.5966 | 5.77E-01 |
| AD | parahippocampal | rs846881 | C | A | -0.01737 | 0.00269 | 9.89E-11 | 1.3877 | 0.8053 | 8.49E-02 |
| AD | parahippocampal | rs867611 | G | A | -0.02043 | 0.00232 | 1.48E-18 | 0.3542 | 0.6233 | 5.70E-01 |
| AD | parahippocampal | rs9381563 | C | T | 0.01445 | 0.00227 | 1.99E-10 | -1.1695 | 0.6108 | 5.55E-02 |
| AD | Superior temporal | rs10933431 | G | C | -0.01544 | 0.00251 | 7.62E-10 | 3.6298 | 2.3663 | 1.25E-01 |
| AD | Superior temporal | rs111278892 | G | C | 0.01991 | 0.00305 | 6.67E-11 | 2.5313 | 2.7594 | 3.59E-01 |
| AD | Superior temporal | rs11218343 | C | T | -0.03593 | 0.00526 | 8.12E-12 | -2.0003 | 4.5874 | 6.63E-01 |
| AD | Superior temporal | rs11257238 | C | T | 0.01294 | 0.00226 | 1.04E-08 | -3.3112 | 1.9398 | 8.78E-02 |
| AD | Superior temporal | rs113260531 | A | G | 0.01999 | 0.00325 | 7.91E-10 | -8.4864 | 2.7227 | 1.83E-03 |
| AD | Superior temporal | rs118170342 | C | T | 0.14754 | 0.00570 | 7.93E-148 | -1.5716 | 5.5325 | 7.76E-01 |
| AD | Superior temporal | rs12590654 | A | G | -0.01483 | 0.00231 | 1.32E-10 | -0.3042 | 1.9442 | 8.76E-01 |
| AD | Superior temporal | rs1859788 | A | G | -0.01840 | 0.00231 | 1.80E-15 | 1.3629 | 2.012 | 4.98E-01 |
| AD | Superior temporal | rs204473 | A | G | -0.04167 | 0.00700 | 2.58E-09 | 3.6981 | 5.8877 | 5.30E-01 |
| AD | Superior temporal | rs2081545 | A | C | -0.01787 | 0.00223 | 1.11E-15 | 1.7373 | 1.8299 | 3.42E-01 |
| AD | Superior temporal | rs28394864 | A | G | 0.01230 | 0.00218 | 1.68E-08 | -3.0142 | 1.8008 | 9.42E-02 |
| AD | Superior temporal | rs28399657 | G | A | -0.05464 | 0.00658 | 9.82E-17 | -5.5906 | 5.4337 | 3.04E-01 |
| AD | Superior temporal | rs41289512 | G | C | 0.20630 | 0.00578 | 1.00E-200 | -2.9299 | 5.4249 | 5.89E-01 |
| AD | Superior temporal | rs41290120 | A | G | -0.09905 | 0.00578 | 7.14E-66 | -8.0815 | 4.6157 | 8.00E-02 |
| AD | Superior temporal | rs4236673 | A | G | -0.02016 | 0.00223 | 1.48E-19 | 3.288 | 1.8453 | 7.48E-02 |
| AD | Superior temporal | rs442495 | C | T | -0.01372 | 0.00226 | 1.22E-09 | -2.1848 | 1.9246 | 2.56E-01 |
| AD | Superior temporal | rs4575098 | A | G | 0.01641 | 0.00258 | 1.90E-10 | -1.4902 | 2.1162 | 4.81E-01 |
| AD | Superior temporal | rs4663105 | C | A | 0.03110 | 0.00222 | 1.45E-44 | 1.6154 | 1.8851 | 3.92E-01 |
| AD | Superior temporal | rs59735493 | A | G | -0.01299 | 0.00236 | 3.73E-08 | 0.8668 | 1.9696 | 6.60E-01 |
| AD | Superior temporal | rs6014724 | G | A | -0.02289 | 0.00369 | 5.38E-10 | 4.0758 | 3.2369 | 2.08E-01 |
| AD | Superior temporal | rs6448453 | A | G | 0.01470 | 0.00245 | 1.98E-09 | -1.7681 | 2.0225 | 3.82E-01 |
| AD | Superior temporal | rs679515 | T | C | 0.02542 | 0.00286 | 6.83E-19 | 0.6075 | 2.3298 | 7.94E-01 |
| AD | Superior temporal | rs755951 | C | A | 0.01500 | 0.00221 | 1.13E-11 | -0.64 | 1.8289 | 7.26E-01 |
| AD | Superior temporal | rs7810606 | T | C | -0.01452 | 0.00218 | 2.89E-11 | 1.3565 | 1.8349 | 4.60E-01 |
| AD | Superior temporal | rs846881 | C | A | -0.01737 | 0.00269 | 9.89E-11 | 2.3145 | 2.4594 | 3.47E-01 |
| AD | Superior temporal | rs867611 | G | A | -0.02043 | 0.00232 | 1.48E-18 | -2.1704 | 1.9204 | 2.58E-01 |
| AD | Superior temporal | rs9381563 | C | T | 0.01445 | 0.00227 | 1.99E-10 | 1.3765 | 1.8816 | 4.65E-01 |
| AD | Temporal pole | rs10933431 | G | C | -0.01544 | 0.00251 | 7.62E-10 | -0.2782 | 0.4767 | 5.60E-01 |
| AD | Temporal pole | rs111278892 | G | C | 0.01991 | 0.00305 | 6.67E-11 | -0.0316 | 0.5521 | 9.54E-01 |
| AD | Temporal pole | rs11218343 | C | T | -0.03593 | 0.00526 | 8.12E-12 | -1.5072 | 0.9246 | 1.03E-01 |
| AD | Temporal pole | rs11257238 | C | T | 0.01294 | 0.00226 | 1.04E-08 | 0.0418 | 0.3892 | 9.15E-01 |
| AD | Temporal pole | rs113260531 | A | G | 0.01999 | 0.00325 | 7.91E-10 | -0.3736 | 0.5496 | 4.97E-01 |
| AD | Temporal pole | rs118170342 | C | T | 0.14754 | 0.00570 | 7.93E-148 | 0.5036 | 1.1189 | 6.53E-01 |
| AD | Temporal pole | rs12590654 | A | G | -0.01483 | 0.00231 | 1.32E-10 | -0.2075 | 0.392 | 5.97E-01 |
| AD | Temporal pole | rs1859788 | A | G | -0.01840 | 0.00231 | 1.80E-15 | -0.2272 | 0.4041 | 5.74E-01 |
| AD | Temporal pole | rs204473 | A | G | -0.04167 | 0.00700 | 2.58E-09 | -1.6282 | 1.1889 | 1.71E-01 |
| AD | Temporal pole | rs2081545 | A | C | -0.01787 | 0.00223 | 1.11E-15 | 0.3589 | 0.3687 | 3.30E-01 |
| AD | Temporal pole | rs28394864 | A | G | 0.01230 | 0.00218 | 1.68E-08 | -0.3993 | 0.3634 | 2.72E-01 |
| AD | Temporal pole | rs28399657 | G | A | -0.05464 | 0.00658 | 9.82E-17 | -0.0946 | 1.0884 | 9.31E-01 |
| AD | Temporal pole | rs41289512 | G | C | 0.20630 | 0.00578 | 1.00E-200 | 0.674 | 1.0915 | 5.37E-01 |
| AD | Temporal pole | rs41290120 | A | G | -0.09905 | 0.00578 | 7.14E-66 | 0.4299 | 0.923 | 6.41E-01 |
| AD | Temporal pole | rs4236673 | A | G | -0.02016 | 0.00223 | 1.48E-19 | -0.2813 | 0.3721 | 4.50E-01 |
| AD | Temporal pole | rs442495 | C | T | -0.01372 | 0.00226 | 1.22E-09 | -0.2017 | 0.3881 | 6.03E-01 |
| AD | Temporal pole | rs4575098 | A | G | 0.01641 | 0.00258 | 1.90E-10 | -0.2667 | 0.4261 | 5.31E-01 |
| AD | Temporal pole | rs4663105 | C | A | 0.03110 | 0.00222 | 1.45E-44 | -0.3642 | 0.3801 | 3.38E-01 |
| AD | Temporal pole | rs59735493 | A | G | -0.01299 | 0.00236 | 3.73E-08 | 0.4916 | 0.3965 | 2.15E-01 |
| AD | Temporal pole | rs6014724 | G | A | -0.02289 | 0.00369 | 5.38E-10 | -0.756 | 0.6505 | 2.45E-01 |
| AD | Temporal pole | rs6448453 | A | G | 0.01470 | 0.00245 | 1.98E-09 | -0.1497 | 0.4084 | 7.14E-01 |
| AD | Temporal pole | rs679515 | T | C | 0.02542 | 0.00286 | 6.83E-19 | 0.3221 | 0.4701 | 4.93E-01 |
| AD | Temporal pole | rs755951 | C | A | 0.01500 | 0.00221 | 1.13E-11 | 0.4378 | 0.3686 | 2.35E-01 |
| AD | Temporal pole | rs7810606 | T | C | -0.01452 | 0.00218 | 2.89E-11 | -0.1559 | 0.3701 | 6.74E-01 |
| AD | Temporal pole | rs846881 | C | A | -0.01737 | 0.00269 | 9.89E-11 | -0.4247 | 0.4952 | 3.91E-01 |
| AD | Temporal pole | rs867611 | G | A | -0.02043 | 0.00232 | 1.48E-18 | -0.3157 | 0.3875 | 4.15E-01 |
| AD | Temporal pole | rs9381563 | C | T | 0.01445 | 0.00227 | 1.99E-10 | 0.1601 | 0.3793 | 6.73E-01 |
| AD | Transverse temporal | rs10933431 | G | C | -0.01544 | 0.00251 | 7.62E-10 | 0.9508 | 0.5101 | 6.24E-02 |
| AD | Transverse temporal | rs111278892 | G | C | 0.01991 | 0.00305 | 6.67E-11 | 0.1289 | 0.5934 | 8.28E-01 |
| AD | Transverse temporal | rs11218343 | C | T | -0.03593 | 0.00526 | 8.12E-12 | -0.0146 | 0.9889 | 9.88E-01 |
| AD | Transverse temporal | rs11257238 | C | T | 0.01294 | 0.00226 | 1.04E-08 | 0.1327 | 0.4156 | 7.50E-01 |
| AD | Transverse temporal | rs113260531 | A | G | 0.01999 | 0.00325 | 7.91E-10 | -0.8524 | 0.5854 | 1.45E-01 |
| AD | Transverse temporal | rs118170342 | C | T | 0.14754 | 0.00570 | 7.93E-148 | 0.4543 | 1.2025 | 7.06E-01 |
| AD | Transverse temporal | rs12590654 | A | G | -0.01483 | 0.00231 | 1.32E-10 | 0.08 | 0.4181 | 8.48E-01 |
| AD | Transverse temporal | rs1859788 | A | G | -0.01840 | 0.00231 | 1.80E-15 | 0.0849 | 0.4328 | 8.45E-01 |
| AD | Transverse temporal | rs204473 | A | G | -0.04167 | 0.00700 | 2.58E-09 | -1.9038 | 1.2673 | 1.33E-01 |
| AD | Transverse temporal | rs2081545 | A | C | -0.01787 | 0.00223 | 1.11E-15 | -0.3044 | 0.393 | 4.39E-01 |
| AD | Transverse temporal | rs28394864 | A | G | 0.01230 | 0.00218 | 1.68E-08 | -0.2199 | 0.3873 | 5.70E-01 |
| AD | Transverse temporal | rs28399657 | G | A | -0.05464 | 0.00658 | 9.82E-17 | -2.2025 | 1.1722 | 6.02E-02 |
| AD | Transverse temporal | rs41289512 | G | C | 0.20630 | 0.00578 | 1.00E-200 | 0.7151 | 1.1763 | 5.43E-01 |
| AD | Transverse temporal | rs41290120 | A | G | -0.09905 | 0.00578 | 7.14E-66 | -2.2447 | 0.9875 | 2.30E-02 |
| AD | Transverse temporal | rs4236673 | A | G | -0.02016 | 0.00223 | 1.48E-19 | 0.5973 | 0.3974 | 1.33E-01 |
| AD | Transverse temporal | rs442495 | C | T | -0.01372 | 0.00226 | 1.22E-09 | 0.1018 | 0.4144 | 8.06E-01 |
| AD | Transverse temporal | rs4575098 | A | G | 0.01641 | 0.00258 | 1.90E-10 | -0.1866 | 0.4537 | 6.81E-01 |
| AD | Transverse temporal | rs4663105 | C | A | 0.03110 | 0.00222 | 1.45E-44 | 0.6297 | 0.4056 | 1.21E-01 |
| AD | Transverse temporal | rs59735493 | A | G | -0.01299 | 0.00236 | 3.73E-08 | -0.3021 | 0.4232 | 4.75E-01 |
| AD | Transverse temporal | rs6014724 | G | A | -0.02289 | 0.00369 | 5.38E-10 | 0.9398 | 0.6969 | 1.78E-01 |
| AD | Transverse temporal | rs6448453 | A | G | 0.01470 | 0.00245 | 1.98E-09 | 0.2257 | 0.4349 | 6.04E-01 |
| AD | Transverse temporal | rs679515 | T | C | 0.02542 | 0.00286 | 6.83E-19 | 0.301 | 0.4994 | 5.47E-01 |
| AD | Transverse temporal | rs755951 | C | A | 0.01500 | 0.00221 | 1.13E-11 | 0.0843 | 0.3929 | 8.30E-01 |
| AD | Transverse temporal | rs7810606 | T | C | -0.01452 | 0.00218 | 2.89E-11 | -0.1449 | 0.3955 | 7.14E-01 |
| AD | Transverse temporal | rs846881 | C | A | -0.01737 | 0.00269 | 9.89E-11 | -0.1756 | 0.5328 | 7.42E-01 |
| AD | Transverse temporal | rs867611 | G | A | -0.02043 | 0.00232 | 1.48E-18 | -0.1374 | 0.4125 | 7.39E-01 |
| AD | Transverse temporal | rs9381563 | C | T | 0.01445 | 0.00227 | 1.99E-10 | 0.5044 | 0.4049 | 2.13E-01 |
| AD | Inferior parietal | rs10933431 | G | C | -0.01544 | 0.00251 | 7.62E-10 | -0.3169 | 4.265 | 9.41E-01 |
| AD | Inferior parietal | rs111278892 | G | C | 0.01991 | 0.00305 | 6.67E-11 | -0.7694 | 4.9809 | 8.77E-01 |
| AD | Inferior parietal | rs11218343 | C | T | -0.03593 | 0.00526 | 8.12E-12 | 6.106 | 8.2822 | 4.61E-01 |
| AD | Inferior parietal | rs11257238 | C | T | 0.01294 | 0.00226 | 1.04E-08 | 0.5126 | 3.4966 | 8.83E-01 |
| AD | Inferior parietal | rs113260531 | A | G | 0.01999 | 0.00325 | 7.91E-10 | 4.8474 | 4.9046 | 3.23E-01 |
| AD | Inferior parietal | rs118170342 | C | T | 0.14754 | 0.00570 | 7.93E-148 | -13.6151 | 10.0074 | 1.74E-01 |
| AD | Inferior parietal | rs12590654 | A | G | -0.01483 | 0.00231 | 1.32E-10 | 0.9897 | 3.5096 | 7.78E-01 |
| AD | Inferior parietal | rs1859788 | A | G | -0.01840 | 0.00231 | 1.80E-15 | -6.1583 | 3.6283 | 8.96E-02 |
| AD | Inferior parietal | rs204473 | A | G | -0.04167 | 0.00700 | 2.58E-09 | -17.8804 | 10.5999 | 9.16E-02 |
| AD | Inferior parietal | rs2081545 | A | C | -0.01787 | 0.00223 | 1.11E-15 | -4.2905 | 3.2983 | 1.93E-01 |
| AD | Inferior parietal | rs28394864 | A | G | 0.01230 | 0.00218 | 1.68E-08 | 6.3299 | 3.2499 | 5.15E-02 |
| AD | Inferior parietal | rs28399657 | G | A | -0.05464 | 0.00658 | 9.82E-17 | 2.7839 | 9.8813 | 7.78E-01 |
| AD | Inferior parietal | rs41289512 | G | C | 0.20630 | 0.00578 | 1.00E-200 | 6.5507 | 9.8481 | 5.06E-01 |
| AD | Inferior parietal | rs41290120 | A | G | -0.09905 | 0.00578 | 7.14E-66 | -6.1666 | 8.3393 | 4.60E-01 |
| AD | Inferior parietal | rs4236673 | A | G | -0.02016 | 0.00223 | 1.48E-19 | 3.1313 | 3.3273 | 3.47E-01 |
| AD | Inferior parietal | rs442495 | C | T | -0.01372 | 0.00226 | 1.22E-09 | 3.91 | 3.4664 | 2.59E-01 |
| AD | Inferior parietal | rs4575098 | A | G | 0.01641 | 0.00258 | 1.90E-10 | -2.6463 | 3.8106 | 4.87E-01 |
| AD | Inferior parietal | rs4663105 | C | A | 0.03110 | 0.00222 | 1.45E-44 | 3.2148 | 3.4044 | 3.45E-01 |
| AD | Inferior parietal | rs59735493 | A | G | -0.01299 | 0.00236 | 3.73E-08 | -3.8325 | 3.5473 | 2.80E-01 |
| AD | Inferior parietal | rs6014724 | G | A | -0.02289 | 0.00369 | 5.38E-10 | -2.0975 | 5.8564 | 7.20E-01 |
| AD | Inferior parietal | rs6448453 | A | G | 0.01470 | 0.00245 | 1.98E-09 | -1.0511 | 3.6459 | 7.73E-01 |
| AD | Inferior parietal | rs679515 | T | C | 0.02542 | 0.00286 | 6.83E-19 | 4.2271 | 4.2064 | 3.15E-01 |
| AD | Inferior parietal | rs755951 | C | A | 0.01500 | 0.00221 | 1.13E-11 | -0.2995 | 3.295 | 9.28E-01 |
| AD | Inferior parietal | rs7810606 | T | C | -0.01452 | 0.00218 | 2.89E-11 | 0.5874 | 3.3178 | 8.60E-01 |
| AD | Inferior parietal | rs846881 | C | A | -0.01737 | 0.00269 | 9.89E-11 | -6.6429 | 4.4652 | 1.37E-01 |
| AD | Inferior parietal | rs867611 | G | A | -0.02043 | 0.00232 | 1.48E-18 | 1.567 | 3.4586 | 6.51E-01 |
| AD | Inferior parietal | rs9381563 | C | T | 0.01445 | 0.00227 | 1.99E-10 | 0.4829 | 3.3938 | 8.87E-01 |
| AD | Isthmus cingulate | rs10933431 | G | C | -0.01544 | 0.00251 | 7.62E-10 | -1.4213 | 1.1451 | 2.15E-01 |
| AD | Isthmus cingulate | rs111278892 | G | C | 0.01991 | 0.00305 | 6.67E-11 | -1.436 | 1.3415 | 2.84E-01 |
| AD | Isthmus cingulate | rs11218343 | C | T | -0.03593 | 0.00526 | 8.12E-12 | -1.5771 | 2.2205 | 4.78E-01 |
| AD | Isthmus cingulate | rs11257238 | C | T | 0.01294 | 0.00226 | 1.04E-08 | 0.0908 | 0.9374 | 9.23E-01 |
| AD | Isthmus cingulate | rs113260531 | A | G | 0.01999 | 0.00325 | 7.91E-10 | 1.0164 | 1.3169 | 4.40E-01 |
| AD | Isthmus cingulate | rs118170342 | C | T | 0.14754 | 0.00570 | 7.93E-148 | -5.0084 | 2.7128 | 6.49E-02 |
| AD | Isthmus cingulate | rs12590654 | A | G | -0.01483 | 0.00231 | 1.32E-10 | 0.1164 | 0.9404 | 9.02E-01 |
| AD | Isthmus cingulate | rs1859788 | A | G | -0.01840 | 0.00231 | 1.80E-15 | -0.2168 | 0.9725 | 8.24E-01 |
| AD | Isthmus cingulate | rs204473 | A | G | -0.04167 | 0.00700 | 2.58E-09 | 4.4492 | 2.8593 | 1.20E-01 |
| AD | Isthmus cingulate | rs2081545 | A | C | -0.01787 | 0.00223 | 1.11E-15 | -0.5429 | 0.8847 | 5.39E-01 |
| AD | Isthmus cingulate | rs28394864 | A | G | 0.01230 | 0.00218 | 1.68E-08 | -0.1642 | 0.8711 | 8.51E-01 |
| AD | Isthmus cingulate | rs28399657 | G | A | -0.05464 | 0.00658 | 9.82E-17 | 0.4111 | 2.6257 | 8.76E-01 |
| AD | Isthmus cingulate | rs41289512 | G | C | 0.20630 | 0.00578 | 1.00E-200 | -3.0023 | 2.6432 | 2.56E-01 |
| AD | Isthmus cingulate | rs41290120 | A | G | -0.09905 | 0.00578 | 7.14E-66 | 3.9184 | 2.2273 | 7.85E-02 |
| AD | Isthmus cingulate | rs4236673 | A | G | -0.02016 | 0.00223 | 1.48E-19 | 0.2076 | 0.8939 | 8.16E-01 |
| AD | Isthmus cingulate | rs442495 | C | T | -0.01372 | 0.00226 | 1.22E-09 | -0.4172 | 0.9325 | 6.55E-01 |
| AD | Isthmus cingulate | rs4575098 | A | G | 0.01641 | 0.00258 | 1.90E-10 | -0.6933 | 1.0213 | 4.97E-01 |
| AD | Isthmus cingulate | rs4663105 | C | A | 0.03110 | 0.00222 | 1.45E-44 | -0.5919 | 0.9133 | 5.17E-01 |
| AD | Isthmus cingulate | rs59735493 | A | G | -0.01299 | 0.00236 | 3.73E-08 | -0.2721 | 0.9517 | 7.75E-01 |
| AD | Isthmus cingulate | rs6014724 | G | A | -0.02289 | 0.00369 | 5.38E-10 | 2.3092 | 1.5653 | 1.40E-01 |
| AD | Isthmus cingulate | rs6448453 | A | G | 0.01470 | 0.00245 | 1.98E-09 | 0.9797 | 0.9759 | 3.15E-01 |
| AD | Isthmus cingulate | rs679515 | T | C | 0.02542 | 0.00286 | 6.83E-19 | -1.4888 | 1.1262 | 1.86E-01 |
| AD | Isthmus cingulate | rs755951 | C | A | 0.01500 | 0.00221 | 1.13E-11 | -0.3998 | 0.8835 | 6.51E-01 |
| AD | Isthmus cingulate | rs7810606 | T | C | -0.01452 | 0.00218 | 2.89E-11 | 1.5493 | 0.8893 | 8.15E-02 |
| AD | Isthmus cingulate | rs846881 | C | A | -0.01737 | 0.00269 | 9.89E-11 | -0.3232 | 1.1974 | 7.87E-01 |
| AD | Isthmus cingulate | rs867611 | G | A | -0.02043 | 0.00232 | 1.48E-18 | -1.1948 | 0.9285 | 1.98E-01 |
| AD | Isthmus cingulate | rs9381563 | C | T | 0.01445 | 0.00227 | 1.99E-10 | -1.3143 | 0.9117 | 1.49E-01 |
| AD | postcentral | rs10933431 | G | C | -0.01544 | 0.00251 | 7.62E-10 | -0.9942 | 2.8516 | 7.27E-01 |
| AD | postcentral | rs111278892 | G | C | 0.01991 | 0.00305 | 6.67E-11 | -2.484 | 3.3217 | 4.55E-01 |
| AD | postcentral | rs11218343 | C | T | -0.03593 | 0.00526 | 8.12E-12 | -7.7511 | 5.5105 | 1.60E-01 |
| AD | postcentral | rs11257238 | C | T | 0.01294 | 0.00226 | 1.04E-08 | 0.1322 | 2.3244 | 9.55E-01 |
| AD | postcentral | rs113260531 | A | G | 0.01999 | 0.00325 | 7.91E-10 | -0.8426 | 3.2851 | 7.98E-01 |
| AD | postcentral | rs118170342 | C | T | 0.14754 | 0.00570 | 7.93E-148 | -3.0083 | 6.7339 | 6.55E-01 |
| AD | postcentral | rs12590654 | A | G | -0.01483 | 0.00231 | 1.32E-10 | -3.283 | 2.3381 | 1.60E-01 |
| AD | postcentral | rs1859788 | A | G | -0.01840 | 0.00231 | 1.80E-15 | 6.9892 | 2.4169 | 3.83E-03 |
| AD | postcentral | rs204473 | A | G | -0.04167 | 0.00700 | 2.58E-09 | 0.5341 | 7.1114 | 9.40E-01 |
| AD | postcentral | rs2081545 | A | C | -0.01787 | 0.00223 | 1.11E-15 | -1.2543 | 2.1936 | 5.68E-01 |
| AD | postcentral | rs28394864 | A | G | 0.01230 | 0.00218 | 1.68E-08 | 0.9034 | 2.1621 | 6.76E-01 |
| AD | postcentral | rs28399657 | G | A | -0.05464 | 0.00658 | 9.82E-17 | 7.7602 | 6.5553 | 2.37E-01 |
| AD | postcentral | rs41289512 | G | C | 0.20630 | 0.00578 | 1.00E-200 | -0.2222 | 6.6686 | 9.73E-01 |
| AD | postcentral | rs41290120 | A | G | -0.09905 | 0.00578 | 7.14E-66 | 2.1691 | 5.5552 | 6.96E-01 |
| AD | postcentral | rs4236673 | A | G | -0.02016 | 0.00223 | 1.48E-19 | 1.4495 | 2.2159 | 5.13E-01 |
| AD | postcentral | rs442495 | C | T | -0.01372 | 0.00226 | 1.22E-09 | -2.3794 | 2.3074 | 3.02E-01 |
| AD | postcentral | rs4575098 | A | G | 0.01641 | 0.00258 | 1.90E-10 | -3.2557 | 2.5324 | 1.99E-01 |
| AD | postcentral | rs4663105 | C | A | 0.03110 | 0.00222 | 1.45E-44 | -1.2682 | 2.2705 | 5.77E-01 |
| AD | postcentral | rs59735493 | A | G | -0.01299 | 0.00236 | 3.73E-08 | 0.8368 | 2.36 | 7.23E-01 |
| AD | postcentral | rs6014724 | G | A | -0.02289 | 0.00369 | 5.38E-10 | 1.6745 | 3.8994 | 6.68E-01 |
| AD | postcentral | rs6448453 | A | G | 0.01470 | 0.00245 | 1.98E-09 | -1.0034 | 2.4256 | 6.79E-01 |
| AD | postcentral | rs679515 | T | C | 0.02542 | 0.00286 | 6.83E-19 | 4.9146 | 2.7935 | 7.85E-02 |
| AD | postcentral | rs755951 | C | A | 0.01500 | 0.00221 | 1.13E-11 | -1.9897 | 2.1925 | 3.64E-01 |
| AD | postcentral | rs7810606 | T | C | -0.01452 | 0.00218 | 2.89E-11 | -1.5554 | 2.2112 | 4.82E-01 |
| AD | postcentral | rs846881 | C | A | -0.01737 | 0.00269 | 9.89E-11 | -1.7236 | 2.9893 | 5.64E-01 |
| AD | postcentral | rs867611 | G | A | -0.02043 | 0.00232 | 1.48E-18 | 1.0538 | 2.3062 | 6.48E-01 |
| AD | postcentral | rs9381563 | C | T | 0.01445 | 0.00227 | 1.99E-10 | -0.9031 | 2.2614 | 6.90E-01 |
| AD | Posterior cingulate | rs10933431 | G | C | -0.01544 | 0.00251 | 7.62E-10 | 0.5201 | 1.1489 | 6.51E-01 |
| AD | Posterior cingulate | rs111278892 | G | C | 0.01991 | 0.00305 | 6.67E-11 | -1.7618 | 1.3438 | 1.90E-01 |
| AD | Posterior cingulate | rs11218343 | C | T | -0.03593 | 0.00526 | 8.12E-12 | 4.1127 | 2.2189 | 6.38E-02 |
| AD | Posterior cingulate | rs11257238 | C | T | 0.01294 | 0.00226 | 1.04E-08 | -0.8535 | 0.9371 | 3.62E-01 |
| AD | Posterior cingulate | rs113260531 | A | G | 0.01999 | 0.00325 | 7.91E-10 | 0.6331 | 1.3177 | 6.31E-01 |
| AD | Posterior cingulate | rs118170342 | C | T | 0.14754 | 0.00570 | 7.93E-148 | -2.7366 | 2.6998 | 3.11E-01 |
| AD | Posterior cingulate | rs12590654 | A | G | -0.01483 | 0.00231 | 1.32E-10 | -0.1303 | 0.9406 | 8.90E-01 |
| AD | Posterior cingulate | rs1859788 | A | G | -0.01840 | 0.00231 | 1.80E-15 | -0.3207 | 0.9738 | 7.42E-01 |
| AD | Posterior cingulate | rs204473 | A | G | -0.04167 | 0.00700 | 2.58E-09 | -2.0135 | 2.8551 | 4.81E-01 |
| AD | Posterior cingulate | rs2081545 | A | C | -0.01787 | 0.00223 | 1.11E-15 | -0.532 | 0.8847 | 5.48E-01 |
| AD | Posterior cingulate | rs28394864 | A | G | 0.01230 | 0.00218 | 1.68E-08 | -1.0595 | 0.871 | 2.24E-01 |
| AD | Posterior cingulate | rs28399657 | G | A | -0.05464 | 0.00658 | 9.82E-17 | -3.3845 | 2.6587 | 2.03E-01 |
| AD | Posterior cingulate | rs41289512 | G | C | 0.20630 | 0.00578 | 1.00E-200 | 1.7983 | 2.658 | 4.99E-01 |
| AD | Posterior cingulate | rs41290120 | A | G | -0.09905 | 0.00578 | 7.14E-66 | -2.5152 | 2.2408 | 2.62E-01 |
| AD | Posterior cingulate | rs4236673 | A | G | -0.02016 | 0.00223 | 1.48E-19 | 0.5181 | 0.8929 | 5.62E-01 |
| AD | Posterior cingulate | rs442495 | C | T | -0.01372 | 0.00226 | 1.22E-09 | -2.5833 | 0.9309 | 5.52E-03 |
| AD | Posterior cingulate | rs4575098 | A | G | 0.01641 | 0.00258 | 1.90E-10 | -1.2297 | 1.0216 | 2.29E-01 |
| AD | Posterior cingulate | rs4663105 | C | A | 0.03110 | 0.00222 | 1.45E-44 | -0.3544 | 0.9135 | 6.98E-01 |
| AD | Posterior cingulate | rs59735493 | A | G | -0.01299 | 0.00236 | 3.73E-08 | 0.7041 | 0.9519 | 4.60E-01 |
| AD | Posterior cingulate | rs6014724 | G | A | -0.02289 | 0.00369 | 5.38E-10 | 1.2399 | 1.5696 | 4.30E-01 |
| AD | Posterior cingulate | rs6448453 | A | G | 0.01470 | 0.00245 | 1.98E-09 | -1.1328 | 0.976 | 2.46E-01 |
| AD | Posterior cingulate | rs679515 | T | C | 0.02542 | 0.00286 | 6.83E-19 | 0.5086 | 1.1248 | 6.51E-01 |
| AD | Posterior cingulate | rs755951 | C | A | 0.01500 | 0.00221 | 1.13E-11 | 0.4263 | 0.8826 | 6.29E-01 |
| AD | Posterior cingulate | rs7810606 | T | C | -0.01452 | 0.00218 | 2.89E-11 | 1.6056 | 0.8898 | 7.12E-02 |
| AD | Posterior cingulate | rs846881 | C | A | -0.01737 | 0.00269 | 9.89E-11 | -0.7534 | 1.2033 | 5.31E-01 |
| AD | Posterior cingulate | rs867611 | G | A | -0.02043 | 0.00232 | 1.48E-18 | -1.1762 | 0.9277 | 2.05E-01 |
| AD | Posterior cingulate | rs9381563 | C | T | 0.01445 | 0.00227 | 1.99E-10 | -0.5411 | 0.9124 | 5.53E-01 |
| AD | precuneus | rs10933431 | G | C | -0.01544 | 0.00251 | 7.62E-10 | -1.3426 | 2.8575 | 6.39E-01 |
| AD | precuneus | rs111278892 | G | C | 0.01991 | 0.00305 | 6.67E-11 | -1.2903 | 3.3392 | 6.99E-01 |
| AD | precuneus | rs11218343 | C | T | -0.03593 | 0.00526 | 8.12E-12 | 1.718 | 5.5305 | 7.56E-01 |
| AD | precuneus | rs11257238 | C | T | 0.01294 | 0.00226 | 1.04E-08 | -0.9811 | 2.3336 | 6.74E-01 |
| AD | precuneus | rs113260531 | A | G | 0.01999 | 0.00325 | 7.91E-10 | 1.0078 | 3.2781 | 7.59E-01 |
| AD | precuneus | rs118170342 | C | T | 0.14754 | 0.00570 | 7.93E-148 | 3.1117 | 6.7248 | 6.44E-01 |
| AD | precuneus | rs12590654 | A | G | -0.01483 | 0.00231 | 1.32E-10 | -1.0743 | 2.3492 | 6.48E-01 |
| AD | precuneus | rs1859788 | A | G | -0.01840 | 0.00231 | 1.80E-15 | 2.4352 | 2.4272 | 3.16E-01 |
| AD | precuneus | rs204473 | A | G | -0.04167 | 0.00700 | 2.58E-09 | -5.3458 | 7.1014 | 4.52E-01 |
| AD | precuneus | rs2081545 | A | C | -0.01787 | 0.00223 | 1.11E-15 | -3.1493 | 2.2059 | 1.53E-01 |
| AD | precuneus | rs28394864 | A | G | 0.01230 | 0.00218 | 1.68E-08 | -3.0635 | 2.1725 | 1.59E-01 |
| AD | precuneus | rs28399657 | G | A | -0.05464 | 0.00658 | 9.82E-17 | -6.0666 | 6.567 | 3.56E-01 |
| AD | precuneus | rs41289512 | G | C | 0.20630 | 0.00578 | 1.00E-200 | -1.2104 | 6.5989 | 8.55E-01 |
| AD | precuneus | rs41290120 | A | G | -0.09905 | 0.00578 | 7.14E-66 | 1.1049 | 5.5801 | 8.43E-01 |
| AD | precuneus | rs4236673 | A | G | -0.02016 | 0.00223 | 1.48E-19 | 1.204 | 2.2264 | 5.89E-01 |
| AD | precuneus | rs442495 | C | T | -0.01372 | 0.00226 | 1.22E-09 | -1.0559 | 2.3161 | 6.48E-01 |
| AD | precuneus | rs4575098 | A | G | 0.01641 | 0.00258 | 1.90E-10 | 5.5102 | 2.5477 | 3.06E-02 |
| AD | precuneus | rs4663105 | C | A | 0.03110 | 0.00222 | 1.45E-44 | -4.887 | 2.2762 | 3.18E-02 |
| AD | precuneus | rs59735493 | A | G | -0.01299 | 0.00236 | 3.73E-08 | 2.1922 | 2.372 | 3.55E-01 |
| AD | precuneus | rs6014724 | G | A | -0.02289 | 0.00369 | 5.38E-10 | 0.1424 | 3.9059 | 9.71E-01 |
| AD | precuneus | rs6448453 | A | G | 0.01470 | 0.00245 | 1.98E-09 | -2.7157 | 2.4358 | 2.65E-01 |
| AD | precuneus | rs679515 | T | C | 0.02542 | 0.00286 | 6.83E-19 | -0.228 | 2.803 | 9.35E-01 |
| AD | precuneus | rs755951 | C | A | 0.01500 | 0.00221 | 1.13E-11 | 2.4439 | 2.2013 | 2.67E-01 |
| AD | precuneus | rs7810606 | T | C | -0.01452 | 0.00218 | 2.89E-11 | 2.6672 | 2.2164 | 2.29E-01 |
| AD | precuneus | rs846881 | C | A | -0.01737 | 0.00269 | 9.89E-11 | -0.5182 | 2.993 | 8.63E-01 |
| AD | precuneus | rs867611 | G | A | -0.02043 | 0.00232 | 1.48E-18 | 0.3889 | 2.3143 | 8.67E-01 |
| AD | precuneus | rs9381563 | C | T | 0.01445 | 0.00227 | 1.99E-10 | -2.9881 | 2.2696 | 1.88E-01 |
| AD | Superior parietal | rs10933431 | G | C | -0.01544 | 0.00251 | 7.62E-10 | 1.2063 | 4.0884 | 7.68E-01 |
| AD | Superior parietal | rs111278892 | G | C | 0.01991 | 0.00305 | 6.67E-11 | 1.8004 | 4.7756 | 7.06E-01 |
| AD | Superior parietal | rs11218343 | C | T | -0.03593 | 0.00526 | 8.12E-12 | -9.8404 | 7.8996 | 2.13E-01 |
| AD | Superior parietal | rs11257238 | C | T | 0.01294 | 0.00226 | 1.04E-08 | 0.8033 | 3.3398 | 8.10E-01 |
| AD | Superior parietal | rs113260531 | A | G | 0.01999 | 0.00325 | 7.91E-10 | -3.4614 | 4.6916 | 4.61E-01 |
| AD | Superior parietal | rs118170342 | C | T | 0.14754 | 0.00570 | 7.93E-148 | -9.2795 | 9.6481 | 3.36E-01 |
| AD | Superior parietal | rs12590654 | A | G | -0.01483 | 0.00231 | 1.32E-10 | 0.1001 | 3.3556 | 9.76E-01 |
| AD | Superior parietal | rs1859788 | A | G | -0.01840 | 0.00231 | 1.80E-15 | 5.6285 | 3.4669 | 1.05E-01 |
| AD | Superior parietal | rs204473 | A | G | -0.04167 | 0.00700 | 2.58E-09 | -6.1781 | 10.1714 | 5.44E-01 |
| AD | Superior parietal | rs2081545 | A | C | -0.01787 | 0.00223 | 1.11E-15 | -2.7501 | 3.1485 | 3.82E-01 |
| AD | Superior parietal | rs28394864 | A | G | 0.01230 | 0.00218 | 1.68E-08 | -0.1024 | 3.1048 | 9.74E-01 |
| AD | Superior parietal | rs28399657 | G | A | -0.05464 | 0.00658 | 9.82E-17 | 6.2889 | 9.4127 | 5.04E-01 |
| AD | Superior parietal | rs41289512 | G | C | 0.20630 | 0.00578 | 1.00E-200 | 16.5725 | 9.4997 | 8.11E-02 |
| AD | Superior parietal | rs41290120 | A | G | -0.09905 | 0.00578 | 7.14E-66 | -9.3868 | 7.983 | 2.40E-01 |
| AD | Superior parietal | rs4236673 | A | G | -0.02016 | 0.00223 | 1.48E-19 | -0.5038 | 3.1823 | 8.74E-01 |
| AD | Superior parietal | rs442495 | C | T | -0.01372 | 0.00226 | 1.22E-09 | -2.7418 | 3.3102 | 4.08E-01 |
| AD | Superior parietal | rs4575098 | A | G | 0.01641 | 0.00258 | 1.90E-10 | 4.8709 | 3.6375 | 1.81E-01 |
| AD | Superior parietal | rs4663105 | C | A | 0.03110 | 0.00222 | 1.45E-44 | -5.6683 | 3.2548 | 8.16E-02 |
| AD | Superior parietal | rs59735493 | A | G | -0.01299 | 0.00236 | 3.73E-08 | 0.5636 | 3.3905 | 8.68E-01 |
| AD | Superior parietal | rs6014724 | G | A | -0.02289 | 0.00369 | 5.38E-10 | 1.0589 | 5.5913 | 8.50E-01 |
| AD | Superior parietal | rs6448453 | A | G | 0.01470 | 0.00245 | 1.98E-09 | -3.6907 | 3.4804 | 2.89E-01 |
| AD | Superior parietal | rs679515 | T | C | 0.02542 | 0.00286 | 6.83E-19 | 2.9607 | 4.0174 | 4.61E-01 |
| AD | Superior parietal | rs755951 | C | A | 0.01500 | 0.00221 | 1.13E-11 | 3.7314 | 3.1487 | 2.36E-01 |
| AD | Superior parietal | rs7810606 | T | C | -0.01452 | 0.00218 | 2.89E-11 | -1.899 | 3.1742 | 5.50E-01 |
| AD | Superior parietal | rs846881 | C | A | -0.01737 | 0.00269 | 9.89E-11 | -5.2885 | 4.2871 | 2.17E-01 |
| AD | Superior parietal | rs867611 | G | A | -0.02043 | 0.00232 | 1.48E-18 | 1.3003 | 3.3065 | 6.94E-01 |
| AD | Superior parietal | rs9381563 | C | T | 0.01445 | 0.00227 | 1.99E-10 | -1.5084 | 3.2429 | 6.42E-01 |
| AD | supramarginal | rs10933431 | G | C | -0.01544 | 0.00251 | 7.62E-10 | -3.697 | 3.2702 | 2.58E-01 |
| AD | supramarginal | rs111278892 | G | C | 0.01991 | 0.00305 | 6.67E-11 | 2.5486 | 3.8203 | 5.05E-01 |
| AD | supramarginal | rs11218343 | C | T | -0.03593 | 0.00526 | 8.12E-12 | -2.484 | 6.3643 | 6.96E-01 |
| AD | supramarginal | rs11257238 | C | T | 0.01294 | 0.00226 | 1.04E-08 | 0.2889 | 2.6822 | 9.14E-01 |
| AD | supramarginal | rs113260531 | A | G | 0.01999 | 0.00325 | 7.91E-10 | 7.0323 | 3.7712 | 6.22E-02 |
| AD | supramarginal | rs118170342 | C | T | 0.14754 | 0.00570 | 7.93E-148 | 0.8742 | 7.6547 | 9.09E-01 |
| AD | supramarginal | rs12590654 | A | G | -0.01483 | 0.00231 | 1.32E-10 | -2.9985 | 2.6907 | 2.65E-01 |
| AD | supramarginal | rs1859788 | A | G | -0.01840 | 0.00231 | 1.80E-15 | -1.8759 | 2.7765 | 4.99E-01 |
| AD | supramarginal | rs204473 | A | G | -0.04167 | 0.00700 | 2.58E-09 | 5.8937 | 8.1941 | 4.72E-01 |
| AD | supramarginal | rs2081545 | A | C | -0.01787 | 0.00223 | 1.11E-15 | -0.0791 | 2.5298 | 9.75E-01 |
| AD | supramarginal | rs28394864 | A | G | 0.01230 | 0.00218 | 1.68E-08 | -6.3484 | 2.4904 | 1.08E-02 |
| AD | supramarginal | rs28399657 | G | A | -0.05464 | 0.00658 | 9.82E-17 | -7.9801 | 7.5335 | 2.90E-01 |
| AD | supramarginal | rs41289512 | G | C | 0.20630 | 0.00578 | 1.00E-200 | -0.2779 | 7.5543 | 9.71E-01 |
| AD | supramarginal | rs41290120 | A | G | -0.09905 | 0.00578 | 7.14E-66 | -6.5235 | 6.4144 | 3.09E-01 |
| AD | supramarginal | rs4236673 | A | G | -0.02016 | 0.00223 | 1.48E-19 | 0.1166 | 2.5492 | 9.64E-01 |
| AD | supramarginal | rs442495 | C | T | -0.01372 | 0.00226 | 1.22E-09 | -0.4926 | 2.6565 | 8.53E-01 |
| AD | supramarginal | rs4575098 | A | G | 0.01641 | 0.00258 | 1.90E-10 | -3.9523 | 2.9232 | 1.76E-01 |
| AD | supramarginal | rs4663105 | C | A | 0.03110 | 0.00222 | 1.45E-44 | 2.8288 | 2.6132 | 2.79E-01 |
| AD | supramarginal | rs59735493 | A | G | -0.01299 | 0.00236 | 3.73E-08 | -3.616 | 2.7201 | 1.84E-01 |
| AD | supramarginal | rs6014724 | G | A | -0.02289 | 0.00369 | 5.38E-10 | -0.5587 | 4.4831 | 9.01E-01 |
| AD | supramarginal | rs6448453 | A | G | 0.01470 | 0.00245 | 1.98E-09 | 2.1488 | 2.7902 | 4.41E-01 |
| AD | supramarginal | rs679515 | T | C | 0.02542 | 0.00286 | 6.83E-19 | 1.1015 | 3.2206 | 7.32E-01 |
| AD | supramarginal | rs755951 | C | A | 0.01500 | 0.00221 | 1.13E-11 | 0.7525 | 2.5241 | 7.66E-01 |
| AD | supramarginal | rs7810606 | T | C | -0.01452 | 0.00218 | 2.89E-11 | 0.14 | 2.5382 | 9.56E-01 |
| AD | supramarginal | rs846881 | C | A | -0.01737 | 0.00269 | 9.89E-11 | 5.9975 | 3.4226 | 7.97E-02 |
| AD | supramarginal | rs867611 | G | A | -0.02043 | 0.00232 | 1.48E-18 | -0.1495 | 2.6527 | 9.55E-01 |
| AD | supramarginal | rs9381563 | C | T | 0.01445 | 0.00227 | 1.99E-10 | 1.0257 | 2.6034 | 6.94E-01 |
| AD | cuneus | rs10933431 | G | C | -0.01544 | 0.00251 | 7.62E-10 | 1.9867 | 1.5192 | 1.91E-01 |
| AD | cuneus | rs111278892 | G | C | 0.01991 | 0.00305 | 6.67E-11 | 1.5745 | 1.7816 | 3.77E-01 |
| AD | cuneus | rs11218343 | C | T | -0.03593 | 0.00526 | 8.12E-12 | 0.9125 | 2.922 | 7.55E-01 |
| AD | cuneus | rs11257238 | C | T | 0.01294 | 0.00226 | 1.04E-08 | 4.2018 | 1.2415 | 7.13E-04 |
| AD | cuneus | rs113260531 | A | G | 0.01999 | 0.00325 | 7.91E-10 | -3.1023 | 1.7389 | 7.44E-02 |
| AD | cuneus | rs118170342 | C | T | 0.14754 | 0.00570 | 7.93E-148 | 4.5778 | 3.5978 | 2.03E-01 |
| AD | cuneus | rs12590654 | A | G | -0.01483 | 0.00231 | 1.32E-10 | -1.3718 | 1.2413 | 2.69E-01 |
| AD | cuneus | rs1859788 | A | G | -0.01840 | 0.00231 | 1.80E-15 | -0.8608 | 1.2851 | 5.03E-01 |
| AD | cuneus | rs204473 | A | G | -0.04167 | 0.00700 | 2.58E-09 | -0.6033 | 3.7639 | 8.73E-01 |
| AD | cuneus | rs2081545 | A | C | -0.01787 | 0.00223 | 1.11E-15 | 2.0952 | 1.1689 | 7.31E-02 |
| AD | cuneus | rs28394864 | A | G | 0.01230 | 0.00218 | 1.68E-08 | 1.8984 | 1.1494 | 9.86E-02 |
| AD | cuneus | rs28399657 | G | A | -0.05464 | 0.00658 | 9.82E-17 | 4.1831 | 3.4674 | 2.28E-01 |
| AD | cuneus | rs41289512 | G | C | 0.20630 | 0.00578 | 1.00E-200 | 6.0991 | 3.5114 | 8.24E-02 |
| AD | cuneus | rs41290120 | A | G | -0.09905 | 0.00578 | 7.14E-66 | -3.6008 | 2.9806 | 2.27E-01 |
| AD | cuneus | rs4236673 | A | G | -0.02016 | 0.00223 | 1.48E-19 | -0.2605 | 1.1798 | 8.25E-01 |
| AD | cuneus | rs442495 | C | T | -0.01372 | 0.00226 | 1.22E-09 | -1.1947 | 1.2254 | 3.30E-01 |
| AD | cuneus | rs4575098 | A | G | 0.01641 | 0.00258 | 1.90E-10 | 1.9258 | 1.3468 | 1.53E-01 |
| AD | cuneus | rs4663105 | C | A | 0.03110 | 0.00222 | 1.45E-44 | -0.7971 | 1.207 | 5.09E-01 |
| AD | cuneus | rs59735493 | A | G | -0.01299 | 0.00236 | 3.73E-08 | 1.1209 | 1.2567 | 3.72E-01 |
| AD | cuneus | rs6014724 | G | A | -0.02289 | 0.00369 | 5.38E-10 | -1.3703 | 2.0762 | 5.09E-01 |
| AD | cuneus | rs6448453 | A | G | 0.01470 | 0.00245 | 1.98E-09 | -0.3271 | 1.289 | 8.00E-01 |
| AD | cuneus | rs679515 | T | C | 0.02542 | 0.00286 | 6.83E-19 | 0.5366 | 1.4821 | 7.17E-01 |
| AD | cuneus | rs755951 | C | A | 0.01500 | 0.00221 | 1.13E-11 | 1.6235 | 1.1654 | 1.64E-01 |
| AD | cuneus | rs7810606 | T | C | -0.01452 | 0.00218 | 2.89E-11 | -1.209 | 1.1727 | 3.03E-01 |
| AD | cuneus | rs846881 | C | A | -0.01737 | 0.00269 | 9.89E-11 | 2.6047 | 1.5865 | 1.01E-01 |
| AD | cuneus | rs867611 | G | A | -0.02043 | 0.00232 | 1.48E-18 | -0.8409 | 1.2233 | 4.92E-01 |
| AD | cuneus | rs9381563 | C | T | 0.01445 | 0.00227 | 1.99E-10 | 1.1478 | 1.2028 | 3.40E-01 |
| AD | Lateral occipital | rs10933431 | G | C | -0.01544 | 0.00251 | 7.62E-10 | 1.4087 | 3.7729 | 7.09E-01 |
| AD | Lateral occipital | rs111278892 | G | C | 0.01991 | 0.00305 | 6.67E-11 | -0.6652 | 4.3902 | 8.80E-01 |
| AD | Lateral occipital | rs11218343 | C | T | -0.03593 | 0.00526 | 8.12E-12 | 6.5284 | 7.2951 | 3.71E-01 |
| AD | Lateral occipital | rs11257238 | C | T | 0.01294 | 0.00226 | 1.04E-08 | 3.6979 | 3.0842 | 2.31E-01 |
| AD | Lateral occipital | rs113260531 | A | G | 0.01999 | 0.00325 | 7.91E-10 | 2.1668 | 4.3261 | 6.17E-01 |
| AD | Lateral occipital | rs118170342 | C | T | 0.14754 | 0.00570 | 7.93E-148 | 2.7916 | 8.8094 | 7.51E-01 |
| AD | Lateral occipital | rs12590654 | A | G | -0.01483 | 0.00231 | 1.32E-10 | -2.5787 | 3.0991 | 4.05E-01 |
| AD | Lateral occipital | rs1859788 | A | G | -0.01840 | 0.00231 | 1.80E-15 | 3.8102 | 3.1981 | 2.34E-01 |
| AD | Lateral occipital | rs204473 | A | G | -0.04167 | 0.00700 | 2.58E-09 | -9.04 | 9.3416 | 3.33E-01 |
| AD | Lateral occipital | rs2081545 | A | C | -0.01787 | 0.00223 | 1.11E-15 | 1.1163 | 2.9117 | 7.01E-01 |
| AD | Lateral occipital | rs28394864 | A | G | 0.01230 | 0.00218 | 1.68E-08 | 1.8199 | 2.87 | 5.26E-01 |
| AD | Lateral occipital | rs28399657 | G | A | -0.05464 | 0.00658 | 9.82E-17 | -2.8794 | 8.6602 | 7.40E-01 |
| AD | Lateral occipital | rs41289512 | G | C | 0.20630 | 0.00578 | 1.00E-200 | -4.4871 | 8.6607 | 6.04E-01 |
| AD | Lateral occipital | rs41290120 | A | G | -0.09905 | 0.00578 | 7.14E-66 | 5.3406 | 7.3496 | 4.67E-01 |
| AD | Lateral occipital | rs4236673 | A | G | -0.02016 | 0.00223 | 1.48E-19 | -1.3095 | 2.9354 | 6.56E-01 |
| AD | Lateral occipital | rs442495 | C | T | -0.01372 | 0.00226 | 1.22E-09 | -2.1683 | 3.0603 | 4.79E-01 |
| AD | Lateral occipital | rs4575098 | A | G | 0.01641 | 0.00258 | 1.90E-10 | 5.2413 | 3.3633 | 1.19E-01 |
| AD | Lateral occipital | rs4663105 | C | A | 0.03110 | 0.00222 | 1.45E-44 | -3.0913 | 3.0051 | 3.04E-01 |
| AD | Lateral occipital | rs59735493 | A | G | -0.01299 | 0.00236 | 3.73E-08 | -5.71 | 3.1331 | 6.84E-02 |
| AD | Lateral occipital | rs6014724 | G | A | -0.02289 | 0.00369 | 5.38E-10 | -0.1245 | 5.1665 | 9.81E-01 |
| AD | Lateral occipital | rs6448453 | A | G | 0.01470 | 0.00245 | 1.98E-09 | 0.651 | 3.2197 | 8.40E-01 |
| AD | Lateral occipital | rs679515 | T | C | 0.02542 | 0.00286 | 6.83E-19 | -1.2776 | 3.7079 | 7.30E-01 |
| AD | Lateral occipital | rs755951 | C | A | 0.01500 | 0.00221 | 1.13E-11 | -3.7814 | 2.9094 | 1.94E-01 |
| AD | Lateral occipital | rs7810606 | T | C | -0.01452 | 0.00218 | 2.89E-11 | -4.46 | 2.9229 | 1.27E-01 |
| AD | Lateral occipital | rs846881 | C | A | -0.01737 | 0.00269 | 9.89E-11 | 0.2899 | 3.9309 | 9.41E-01 |
| AD | Lateral occipital | rs867611 | G | A | -0.02043 | 0.00232 | 1.48E-18 | 0.7692 | 3.0547 | 8.01E-01 |
| AD | Lateral occipital | rs9381563 | C | T | 0.01445 | 0.00227 | 1.99E-10 | -2.3497 | 2.9953 | 4.33E-01 |
| AD | lingual | rs10933431 | G | C | -0.01544 | 0.00251 | 7.62E-10 | -4.2261 | 3.0428 | 1.65E-01 |
| AD | lingual | rs111278892 | G | C | 0.01991 | 0.00305 | 6.67E-11 | -3.9124 | 3.5426 | 2.69E-01 |
| AD | lingual | rs11218343 | C | T | -0.03593 | 0.00526 | 8.12E-12 | 1.8578 | 5.8878 | 7.52E-01 |
| AD | lingual | rs11257238 | C | T | 0.01294 | 0.00226 | 1.04E-08 | 0.7806 | 2.4805 | 7.53E-01 |
| AD | lingual | rs113260531 | A | G | 0.01999 | 0.00325 | 7.91E-10 | 0.3373 | 3.4867 | 9.23E-01 |
| AD | lingual | rs118170342 | C | T | 0.14754 | 0.00570 | 7.93E-148 | 5.1991 | 7.1842 | 4.69E-01 |
| AD | lingual | rs12590654 | A | G | -0.01483 | 0.00231 | 1.32E-10 | -0.1102 | 2.4873 | 9.65E-01 |
| AD | lingual | rs1859788 | A | G | -0.01840 | 0.00231 | 1.80E-15 | -3.3929 | 2.5753 | 1.88E-01 |
| AD | lingual | rs204473 | A | G | -0.04167 | 0.00700 | 2.58E-09 | -9.3982 | 7.5749 | 2.15E-01 |
| AD | lingual | rs2081545 | A | C | -0.01787 | 0.00223 | 1.11E-15 | -2.0112 | 2.3427 | 3.91E-01 |
| AD | lingual | rs28394864 | A | G | 0.01230 | 0.00218 | 1.68E-08 | -0.2815 | 2.305 | 9.03E-01 |
| AD | lingual | rs28399657 | G | A | -0.05464 | 0.00658 | 9.82E-17 | 10.7053 | 7.0127 | 1.27E-01 |
| AD | lingual | rs41289512 | G | C | 0.20630 | 0.00578 | 1.00E-200 | -4.0656 | 6.9688 | 5.60E-01 |
| AD | lingual | rs41290120 | A | G | -0.09905 | 0.00578 | 7.14E-66 | 6.3289 | 5.9122 | 2.84E-01 |
| AD | lingual | rs4236673 | A | G | -0.02016 | 0.00223 | 1.48E-19 | -3.5113 | 2.3637 | 1.37E-01 |
| AD | lingual | rs442495 | C | T | -0.01372 | 0.00226 | 1.22E-09 | -1.0425 | 2.4685 | 6.73E-01 |
| AD | lingual | rs4575098 | A | G | 0.01641 | 0.00258 | 1.90E-10 | 4.1944 | 2.7031 | 1.21E-01 |
| AD | lingual | rs4663105 | C | A | 0.03110 | 0.00222 | 1.45E-44 | 0.002 | 2.4177 | 9.99E-01 |
| AD | lingual | rs59735493 | A | G | -0.01299 | 0.00236 | 3.73E-08 | -4.744 | 2.522 | 6.00E-02 |
| AD | lingual | rs6014724 | G | A | -0.02289 | 0.00369 | 5.38E-10 | -1.8561 | 4.1514 | 6.55E-01 |
| AD | lingual | rs6448453 | A | G | 0.01470 | 0.00245 | 1.98E-09 | 0.9198 | 2.5886 | 7.22E-01 |
| AD | lingual | rs679515 | T | C | 0.02542 | 0.00286 | 6.83E-19 | 3.5758 | 2.9723 | 2.29E-01 |
| AD | lingual | rs755951 | C | A | 0.01500 | 0.00221 | 1.13E-11 | -1.9957 | 2.3398 | 3.94E-01 |
| AD | lingual | rs7810606 | T | C | -0.01452 | 0.00218 | 2.89E-11 | -0.2125 | 2.3522 | 9.28E-01 |
| AD | lingual | rs846881 | C | A | -0.01737 | 0.00269 | 9.89E-11 | 6.0533 | 3.1682 | 5.61E-02 |
| AD | lingual | rs867611 | G | A | -0.02043 | 0.00232 | 1.48E-18 | -0.1565 | 2.454 | 9.49E-01 |
| AD | lingual | rs9381563 | C | T | 0.01445 | 0.00227 | 1.99E-10 | 4.1418 | 2.4111 | 8.58E-02 |
| AD | pericalcarine | rs10933431 | G | C | -0.01544 | 0.00251 | 7.62E-10 | -1.7622 | 2.0157 | 3.82E-01 |
| AD | pericalcarine | rs111278892 | G | C | 0.01991 | 0.00305 | 6.67E-11 | 0.5087 | 2.3456 | 8.28E-01 |
| AD | pericalcarine | rs11218343 | C | T | -0.03593 | 0.00526 | 8.12E-12 | 0.8356 | 3.8978 | 8.30E-01 |
| AD | pericalcarine | rs11257238 | C | T | 0.01294 | 0.00226 | 1.04E-08 | 3.0404 | 1.6421 | 6.41E-02 |
| AD | pericalcarine | rs113260531 | A | G | 0.01999 | 0.00325 | 7.91E-10 | -2.7409 | 2.3057 | 2.35E-01 |
| AD | pericalcarine | rs118170342 | C | T | 0.14754 | 0.00570 | 7.93E-148 | 5.0374 | 4.7567 | 2.90E-01 |
| AD | pericalcarine | rs12590654 | A | G | -0.01483 | 0.00231 | 1.32E-10 | -0.8735 | 1.6458 | 5.96E-01 |
| AD | pericalcarine | rs1859788 | A | G | -0.01840 | 0.00231 | 1.80E-15 | -2.6669 | 1.706 | 1.18E-01 |
| AD | pericalcarine | rs204473 | A | G | -0.04167 | 0.00700 | 2.58E-09 | 0.2293 | 5.0098 | 9.64E-01 |
| AD | pericalcarine | rs2081545 | A | C | -0.01787 | 0.00223 | 1.11E-15 | 0.3161 | 1.5519 | 8.39E-01 |
| AD | pericalcarine | rs28394864 | A | G | 0.01230 | 0.00218 | 1.68E-08 | -0.134 | 1.5259 | 9.30E-01 |
| AD | pericalcarine | rs28399657 | G | A | -0.05464 | 0.00658 | 9.82E-17 | 7.1795 | 4.6206 | 1.20E-01 |
| AD | pericalcarine | rs41289512 | G | C | 0.20630 | 0.00578 | 1.00E-200 | 5.7562 | 4.6373 | 2.15E-01 |
| AD | pericalcarine | rs41290120 | A | G | -0.09905 | 0.00578 | 7.14E-66 | -3.5402 | 3.915 | 3.66E-01 |
| AD | pericalcarine | rs4236673 | A | G | -0.02016 | 0.00223 | 1.48E-19 | -0.9367 | 1.5662 | 5.50E-01 |
| AD | pericalcarine | rs442495 | C | T | -0.01372 | 0.00226 | 1.22E-09 | -1.5777 | 1.6336 | 3.34E-01 |
| AD | pericalcarine | rs4575098 | A | G | 0.01641 | 0.00258 | 1.90E-10 | 4.4453 | 1.788 | 1.29E-02 |
| AD | pericalcarine | rs4663105 | C | A | 0.03110 | 0.00222 | 1.45E-44 | -0.2301 | 1.601 | 8.86E-01 |
| AD | pericalcarine | rs59735493 | A | G | -0.01299 | 0.00236 | 3.73E-08 | 1.4317 | 1.6707 | 3.92E-01 |
| AD | pericalcarine | rs6014724 | G | A | -0.02289 | 0.00369 | 5.38E-10 | 0.2062 | 2.755 | 9.40E-01 |
| AD | pericalcarine | rs6448453 | A | G | 0.01470 | 0.00245 | 1.98E-09 | -0.6445 | 1.7131 | 7.07E-01 |
| AD | pericalcarine | rs679515 | T | C | 0.02542 | 0.00286 | 6.83E-19 | 1.2513 | 1.9679 | 5.25E-01 |
| AD | pericalcarine | rs755951 | C | A | 0.01500 | 0.00221 | 1.13E-11 | 0.7373 | 1.5491 | 6.34E-01 |
| AD | pericalcarine | rs7810606 | T | C | -0.01452 | 0.00218 | 2.89E-11 | -1.8076 | 1.5573 | 2.46E-01 |
| AD | pericalcarine | rs846881 | C | A | -0.01737 | 0.00269 | 9.89E-11 | 3.1631 | 2.0977 | 1.32E-01 |
| AD | pericalcarine | rs867611 | G | A | -0.02043 | 0.00232 | 1.48E-18 | -0.8971 | 1.6261 | 5.81E-01 |
| AD | pericalcarine | rs9381563 | C | T | 0.01445 | 0.00227 | 1.99E-10 | 0.1853 | 1.5949 | 9.08E-01 |
| AD | whole cortex | rs10933431 | G | C | -0.01544 | 0.00251 | 7.62E-10 | 120.0361 | 143.7415 | 4.04E-01 |
| AD | whole cortex | rs111278892 | G | C | 0.01991 | 0.00305 | 6.67E-11 | -223.7459 | 166.962 | 1.80E-01 |
| AD | whole cortex | rs11218343 | C | T | -0.03593 | 0.00526 | 8.12E-12 | -87.2116 | 279.0985 | 7.55E-01 |
| AD | whole cortex | rs11257238 | C | T | 0.01294 | 0.00226 | 1.04E-08 | 7.2341 | 118.2524 | 9.51E-01 |
| AD | whole cortex | rs113260531 | A | G | 0.01999 | 0.00325 | 7.91E-10 | 48.1362 | 165.9118 | 7.72E-01 |
| AD | whole cortex | rs118170342 | C | T | 0.14754 | 0.00570 | 7.93E-148 | -341.9125 | 337.5136 | 3.11E-01 |
| AD | whole cortex | rs12590654 | A | G | -0.01483 | 0.00231 | 1.32E-10 | -71.2019 | 118.6423 | 5.48E-01 |
| AD | whole cortex | rs1859788 | A | G | -0.01840 | 0.00231 | 1.80E-15 | 184.8328 | 122.6544 | 1.32E-01 |
| AD | whole cortex | rs204473 | A | G | -0.04167 | 0.00700 | 2.58E-09 | 158.4311 | 359.7203 | 6.60E-01 |
| AD | whole cortex | rs2081545 | A | C | -0.01787 | 0.00223 | 1.11E-15 | 2.6203 | 111.4784 | 9.81E-01 |
| AD | whole cortex | rs28394864 | A | G | 0.01230 | 0.00218 | 1.68E-08 | -49.163 | 109.9861 | 6.55E-01 |
| AD | whole cortex | rs28399657 | G | A | -0.05464 | 0.00658 | 9.82E-17 | -31.205 | 321.9391 | 9.23E-01 |
| AD | whole cortex | rs41289512 | G | C | 0.20630 | 0.00578 | 1.00E-200 | 305.0248 | 330.5691 | 3.56E-01 |
| AD | whole cortex | rs41290120 | A | G | -0.09905 | 0.00578 | 7.14E-66 | -232.4817 | 277.8486 | 4.03E-01 |
| AD | whole cortex | rs4236673 | A | G | -0.02016 | 0.00223 | 1.48E-19 | -4.9168 | 112.7008 | 9.65E-01 |
| AD | whole cortex | rs442495 | C | T | -0.01372 | 0.00226 | 1.22E-09 | 287.7246 | 117.4735 | 1.43E-02 |
| AD | whole cortex | rs4575098 | A | G | 0.01641 | 0.00258 | 1.90E-10 | -109.1834 | 128.7205 | 3.96E-01 |
| AD | whole cortex | rs4663105 | C | A | 0.03110 | 0.00222 | 1.45E-44 | -119.0745 | 114.8653 | 3.00E-01 |
| AD | whole cortex | rs59735493 | A | G | -0.01299 | 0.00236 | 3.73E-08 | -342.8858 | 120.0449 | 4.29E-03 |
| AD | whole cortex | rs6014724 | G | A | -0.02289 | 0.00369 | 5.38E-10 | 137.2758 | 196.0606 | 4.84E-01 |
| AD | whole cortex | rs6448453 | A | G | 0.01470 | 0.00245 | 1.98E-09 | -42.0156 | 123.575 | 7.34E-01 |
| AD | whole cortex | rs679515 | T | C | 0.02542 | 0.00286 | 6.83E-19 | -50.7618 | 142.4855 | 7.22E-01 |
| AD | whole cortex | rs755951 | C | A | 0.01500 | 0.00221 | 1.13E-11 | 52.7308 | 111.655 | 6.37E-01 |
| AD | whole cortex | rs7810606 | T | C | -0.01452 | 0.00218 | 2.89E-11 | 183.5674 | 111.8653 | 1.01E-01 |
| AD | whole cortex | rs846881 | C | A | -0.01737 | 0.00269 | 9.89E-11 | 33.8897 | 150.2947 | 8.22E-01 |
| AD | whole cortex | rs867611 | G | A | -0.02043 | 0.00232 | 1.48E-18 | 196.6688 | 117.3202 | 9.37E-02 |
| AD | whole cortex | rs9381563 | C | T | 0.01445 | 0.00227 | 1.99E-10 | 39.5637 | 114.6054 | 7.30E-01 |

**Table S5.** Summary of genetic variants used to estimate the effect of Alzheimer's disease (AD) on cortical thickness

| **Exposure** | **outcome** | **SNP** | **effect allele** | **other allele** | **beta exposure** | **se exposure** | **pval exposure** | **beta outcome** | **se outcome** | **pval outcome** |
| --- | --- | --- | --- | --- | --- | --- | --- | --- | --- | --- |
| AD | Caudal anterior cingulate | rs10933431 | G | C | -0.01544 | 0.00251 | 7.62E-10 | 0.0017 | 0.0021 | 4.01E-01 |
| AD | Caudal anterior cingulate | rs111278892 | G | C | 0.01991 | 0.00305 | 6.67E-11 | 0.0006 | 0.0024 | 8.13E-01 |
| AD | Caudal anterior cingulate | rs11218343 | C | T | -0.03593 | 0.00526 | 8.12E-12 | -0.0029 | 0.0039 | 4.52E-01 |
| AD | Caudal anterior cingulate | rs11257238 | C | T | 0.01294 | 0.00226 | 1.04E-08 | 0.0021 | 0.0017 | 2.21E-01 |
| AD | Caudal anterior cingulate | rs113260531 | A | G | 0.01999 | 0.00325 | 7.91E-10 | -0.0012 | 0.0023 | 6.11E-01 |
| AD | Caudal anterior cingulate | rs118170342 | C | T | 0.14754 | 0.00570 | 7.93E-148 | 0.0054 | 0.0050 | 2.74E-01 |
| AD | Caudal anterior cingulate | rs12590654 | A | G | -0.01483 | 0.00231 | 1.32E-10 | -0.0001 | 0.0016 | 9.67E-01 |
| AD | Caudal anterior cingulate | rs1859788 | A | G | -0.01840 | 0.00231 | 1.80E-15 | 0.0026 | 0.0017 | 1.22E-01 |
| AD | Caudal anterior cingulate | rs204473 | A | G | -0.04167 | 0.00700 | 2.58E-09 | -0.0029 | 0.0051 | 5.69E-01 |
| AD | Caudal anterior cingulate | rs2081545 | A | C | -0.01787 | 0.00223 | 1.11E-15 | -0.0008 | 0.0016 | 5.98E-01 |
| AD | Caudal anterior cingulate | rs28394864 | A | G | 0.01230 | 0.00218 | 1.68E-08 | -0.0003 | 0.0016 | 8.53E-01 |
| AD | Caudal anterior cingulate | rs28399657 | G | A | -0.05464 | 0.00658 | 9.82E-17 | 0.0054 | 0.0046 | 2.39E-01 |
| AD | Caudal anterior cingulate | rs41289512 | G | C | 0.20630 | 0.00578 | 1.00E-200 | -0.0057 | 0.0048 | 2.32E-01 |
| AD | Caudal anterior cingulate | rs41290120 | A | G | -0.09905 | 0.00578 | 7.14E-66 | 0.0052 | 0.0039 | 1.84E-01 |
| AD | Caudal anterior cingulate | rs4236673 | A | G | -0.02016 | 0.00223 | 1.48E-19 | 0.0007 | 0.0016 | 6.37E-01 |
| AD | Caudal anterior cingulate | rs442495 | C | T | -0.01372 | 0.00226 | 1.22E-09 | 0.0005 | 0.0017 | 7.42E-01 |
| AD | Caudal anterior cingulate | rs4575098 | A | G | 0.01641 | 0.00258 | 1.90E-10 | 0.0017 | 0.0018 | 3.39E-01 |
| AD | Caudal anterior cingulate | rs4663105 | C | A | 0.03110 | 0.00222 | 1.45E-44 | -0.0011 | 0.0016 | 5.16E-01 |
| AD | Caudal anterior cingulate | rs59735493 | A | G | -0.01299 | 0.00236 | 3.73E-08 | -0.0021 | 0.0017 | 2.07E-01 |
| AD | Caudal anterior cingulate | rs6014724 | G | A | -0.02289 | 0.00369 | 5.38E-10 | -0.0013 | 0.0028 | 6.50E-01 |
| AD | Caudal anterior cingulate | rs6448453 | A | G | 0.01470 | 0.00245 | 1.98E-09 | -0.0039 | 0.0017 | 2.58E-02 |
| AD | Caudal anterior cingulate | rs679515 | T | C | 0.02542 | 0.00286 | 6.83E-19 | -0.0033 | 0.0020 | 1.02E-01 |
| AD | Caudal anterior cingulate | rs755951 | C | A | 0.01500 | 0.00221 | 1.13E-11 | -0.0002 | 0.0016 | 8.94E-01 |
| AD | Caudal anterior cingulate | rs7810606 | T | C | -0.01452 | 0.00218 | 2.89E-11 | -0.0011 | 0.0016 | 4.86E-01 |
| AD | Caudal anterior cingulate | rs846881 | C | A | -0.01737 | 0.00269 | 9.89E-11 | -0.0006 | 0.0021 | 7.89E-01 |
| AD | Caudal anterior cingulate | rs867611 | G | A | -0.02043 | 0.00232 | 1.48E-18 | 0.0015 | 0.0016 | 3.49E-01 |
| AD | Caudal anterior cingulate | rs9381563 | C | T | 0.01445 | 0.00227 | 1.99E-10 | 0.0013 | 0.0016 | 4.25E-01 |
| AD | Caudal middle frontal | rs10933431 | G | C | -0.01544 | 0.00251 | 7.62E-10 | 0.0000 | 0.0010 | 9.89E-01 |
| AD | Caudal middle frontal | rs111278892 | G | C | 0.01991 | 0.00305 | 6.67E-11 | 0.0004 | 0.0011 | 7.31E-01 |
| AD | Caudal middle frontal | rs11218343 | C | T | -0.03593 | 0.00526 | 8.12E-12 | 0.0015 | 0.0019 | 4.29E-01 |
| AD | Caudal middle frontal | rs11257238 | C | T | 0.01294 | 0.00226 | 1.04E-08 | 0.0003 | 0.0008 | 7.43E-01 |
| AD | Caudal middle frontal | rs113260531 | A | G | 0.01999 | 0.00325 | 7.91E-10 | -0.0006 | 0.0011 | 5.93E-01 |
| AD | Caudal middle frontal | rs118170342 | C | T | 0.14754 | 0.00570 | 7.93E-148 | -0.0011 | 0.0023 | 6.36E-01 |
| AD | Caudal middle frontal | rs12590654 | A | G | -0.01483 | 0.00231 | 1.32E-10 | 0.0001 | 0.0008 | 8.48E-01 |
| AD | Caudal middle frontal | rs1859788 | A | G | -0.01840 | 0.00231 | 1.80E-15 | 0.0013 | 0.0008 | 1.27E-01 |
| AD | Caudal middle frontal | rs204473 | A | G | -0.04167 | 0.00700 | 2.58E-09 | 0.0022 | 0.0024 | 3.58E-01 |
| AD | Caudal middle frontal | rs2081545 | A | C | -0.01787 | 0.00223 | 1.11E-15 | -0.0001 | 0.0008 | 8.72E-01 |
| AD | Caudal middle frontal | rs28394864 | A | G | 0.01230 | 0.00218 | 1.68E-08 | 0.0001 | 0.0007 | 8.85E-01 |
| AD | Caudal middle frontal | rs28399657 | G | A | -0.05464 | 0.00658 | 9.82E-17 | 0.0016 | 0.0022 | 4.59E-01 |
| AD | Caudal middle frontal | rs41289512 | G | C | 0.20630 | 0.00578 | 1.00E-200 | -0.0027 | 0.0023 | 2.52E-01 |
| AD | Caudal middle frontal | rs41290120 | A | G | -0.09905 | 0.00578 | 7.14E-66 | 0.0028 | 0.0018 | 1.32E-01 |
| AD | Caudal middle frontal | rs4236673 | A | G | -0.02016 | 0.00223 | 1.48E-19 | -0.0006 | 0.0008 | 4.45E-01 |
| AD | Caudal middle frontal | rs442495 | C | T | -0.01372 | 0.00226 | 1.22E-09 | 0.0007 | 0.0008 | 3.74E-01 |
| AD | Caudal middle frontal | rs4575098 | A | G | 0.01641 | 0.00258 | 1.90E-10 | 0.0006 | 0.0009 | 4.87E-01 |
| AD | Caudal middle frontal | rs4663105 | C | A | 0.03110 | 0.00222 | 1.45E-44 | 0.0007 | 0.0008 | 3.47E-01 |
| AD | Caudal middle frontal | rs59735493 | A | G | -0.01299 | 0.00236 | 3.73E-08 | -0.0009 | 0.0008 | 2.32E-01 |
| AD | Caudal middle frontal | rs6014724 | G | A | -0.02289 | 0.00369 | 5.38E-10 | 0.0005 | 0.0013 | 6.87E-01 |
| AD | Caudal middle frontal | rs6448453 | A | G | 0.01470 | 0.00245 | 1.98E-09 | 0.0004 | 0.0008 | 6.18E-01 |
| AD | Caudal middle frontal | rs679515 | T | C | 0.02542 | 0.00286 | 6.83E-19 | -0.0012 | 0.0010 | 1.98E-01 |
| AD | Caudal middle frontal | rs755951 | C | A | 0.01500 | 0.00221 | 1.13E-11 | -0.0015 | 0.0008 | 4.89E-02 |
| AD | Caudal middle frontal | rs7810606 | T | C | -0.01452 | 0.00218 | 2.89E-11 | 0.0006 | 0.0008 | 4.08E-01 |
| AD | Caudal middle frontal | rs846881 | C | A | -0.01737 | 0.00269 | 9.89E-11 | -0.0021 | 0.0010 | 4.62E-02 |
| AD | Caudal middle frontal | rs867611 | G | A | -0.02043 | 0.00232 | 1.48E-18 | -0.0016 | 0.0008 | 4.01E-02 |
| AD | Caudal middle frontal | rs9381563 | C | T | 0.01445 | 0.00227 | 1.99E-10 | -0.0012 | 0.0008 | 1.16E-01 |
| AD | Frontal pole | rs10933431 | G | C | -0.01544 | 0.00251 | 7.62E-10 | -0.0005 | 0.0022 | 8.20E-01 |
| AD | Frontal pole | rs111278892 | G | C | 0.01991 | 0.00305 | 6.67E-11 | -0.0027 | 0.0025 | 2.78E-01 |
| AD | Frontal pole | rs11218343 | C | T | -0.03593 | 0.00526 | 8.12E-12 | 0.0013 | 0.0042 | 7.57E-01 |
| AD | Frontal pole | rs11257238 | C | T | 0.01294 | 0.00226 | 1.04E-08 | -0.0009 | 0.0018 | 6.27E-01 |
| AD | Frontal pole | rs113260531 | A | G | 0.01999 | 0.00325 | 7.91E-10 | -0.0006 | 0.0025 | 8.22E-01 |
| AD | Frontal pole | rs118170342 | C | T | 0.14754 | 0.00570 | 7.93E-148 | -0.0052 | 0.0052 | 3.12E-01 |
| AD | Frontal pole | rs12590654 | A | G | -0.01483 | 0.00231 | 1.32E-10 | 0.0008 | 0.0018 | 6.70E-01 |
| AD | Frontal pole | rs1859788 | A | G | -0.01840 | 0.00231 | 1.80E-15 | 0.0017 | 0.0019 | 3.65E-01 |
| AD | Frontal pole | rs204473 | A | G | -0.04167 | 0.00700 | 2.58E-09 | -0.0005 | 0.0054 | 9.24E-01 |
| AD | Frontal pole | rs2081545 | A | C | -0.01787 | 0.00223 | 1.11E-15 | -0.0014 | 0.0017 | 4.06E-01 |
| AD | Frontal pole | rs28394864 | A | G | 0.01230 | 0.00218 | 1.68E-08 | 0.0017 | 0.0017 | 3.18E-01 |
| AD | Frontal pole | rs28399657 | G | A | -0.05464 | 0.00658 | 9.82E-17 | -0.0028 | 0.0049 | 5.72E-01 |
| AD | Frontal pole | rs41289512 | G | C | 0.20630 | 0.00578 | 1.00E-200 | 0.0051 | 0.0050 | 3.08E-01 |
| AD | Frontal pole | rs41290120 | A | G | -0.09905 | 0.00578 | 7.14E-66 | 0.0001 | 0.0042 | 9.90E-01 |
| AD | Frontal pole | rs4236673 | A | G | -0.02016 | 0.00223 | 1.48E-19 | -0.0001 | 0.0017 | 9.54E-01 |
| AD | Frontal pole | rs442495 | C | T | -0.01372 | 0.00226 | 1.22E-09 | -0.0015 | 0.0018 | 4.01E-01 |
| AD | Frontal pole | rs4575098 | A | G | 0.01641 | 0.00258 | 1.90E-10 | 0.0008 | 0.0019 | 6.60E-01 |
| AD | Frontal pole | rs4663105 | C | A | 0.03110 | 0.00222 | 1.45E-44 | -0.0002 | 0.0017 | 9.15E-01 |
| AD | Frontal pole | rs59735493 | A | G | -0.01299 | 0.00236 | 3.73E-08 | -0.0010 | 0.0018 | 5.95E-01 |
| AD | Frontal pole | rs6014724 | G | A | -0.02289 | 0.00369 | 5.38E-10 | 0.0025 | 0.0030 | 4.10E-01 |
| AD | Frontal pole | rs6448453 | A | G | 0.01470 | 0.00245 | 1.98E-09 | 0.0018 | 0.0018 | 3.40E-01 |
| AD | Frontal pole | rs679515 | T | C | 0.02542 | 0.00286 | 6.83E-19 | 0.0001 | 0.0022 | 9.61E-01 |
| AD | Frontal pole | rs755951 | C | A | 0.01500 | 0.00221 | 1.13E-11 | -0.0008 | 0.0017 | 6.21E-01 |
| AD | Frontal pole | rs7810606 | T | C | -0.01452 | 0.00218 | 2.89E-11 | -0.0022 | 0.0017 | 2.07E-01 |
| AD | Frontal pole | rs846881 | C | A | -0.01737 | 0.00269 | 9.89E-11 | 0.0014 | 0.0023 | 5.22E-01 |
| AD | Frontal pole | rs867611 | G | A | -0.02043 | 0.00232 | 1.48E-18 | 0.0019 | 0.0017 | 2.66E-01 |
| AD | Frontal pole | rs9381563 | C | T | 0.01445 | 0.00227 | 1.99E-10 | -0.0008 | 0.0017 | 6.40E-01 |
| AD | Lateral orbitofrontal | rs10933431 | G | C | -0.01544 | 0.00251 | 7.62E-10 | 0.0000 | 0.0012 | 9.95E-01 |
| AD | Lateral orbitofrontal | rs111278892 | G | C | 0.01991 | 0.00305 | 6.67E-11 | 0.0018 | 0.0013 | 1.69E-01 |
| AD | Lateral orbitofrontal | rs11218343 | C | T | -0.03593 | 0.00526 | 8.12E-12 | -0.0002 | 0.0022 | 9.14E-01 |
| AD | Lateral orbitofrontal | rs11257238 | C | T | 0.01294 | 0.00226 | 1.04E-08 | -0.0012 | 0.0010 | 1.94E-01 |
| AD | Lateral orbitofrontal | rs113260531 | A | G | 0.01999 | 0.00325 | 7.91E-10 | 0.0009 | 0.0013 | 4.86E-01 |
| AD | Lateral orbitofrontal | rs118170342 | C | T | 0.14754 | 0.00570 | 7.93E-148 | -0.0004 | 0.0027 | 8.71E-01 |
| AD | Lateral orbitofrontal | rs12590654 | A | G | -0.01483 | 0.00231 | 1.32E-10 | 0.0009 | 0.0010 | 3.30E-01 |
| AD | Lateral orbitofrontal | rs1859788 | A | G | -0.01840 | 0.00231 | 1.80E-15 | -0.0024 | 0.0010 | 1.33E-02 |
| AD | Lateral orbitofrontal | rs204473 | A | G | -0.04167 | 0.00700 | 2.58E-09 | -0.0007 | 0.0029 | 8.04E-01 |
| AD | Lateral orbitofrontal | rs2081545 | A | C | -0.01787 | 0.00223 | 1.11E-15 | 0.0007 | 0.0009 | 4.66E-01 |
| AD | Lateral orbitofrontal | rs28394864 | A | G | 0.01230 | 0.00218 | 1.68E-08 | -0.0004 | 0.0009 | 6.71E-01 |
| AD | Lateral orbitofrontal | rs28399657 | G | A | -0.05464 | 0.00658 | 9.82E-17 | 0.0006 | 0.0026 | 8.10E-01 |
| AD | Lateral orbitofrontal | rs41289512 | G | C | 0.20630 | 0.00578 | 1.00E-200 | -0.0014 | 0.0027 | 6.08E-01 |
| AD | Lateral orbitofrontal | rs41290120 | A | G | -0.09905 | 0.00578 | 7.14E-66 | 0.0008 | 0.0022 | 7.13E-01 |
| AD | Lateral orbitofrontal | rs4236673 | A | G | -0.02016 | 0.00223 | 1.48E-19 | -0.0012 | 0.0009 | 1.80E-01 |
| AD | Lateral orbitofrontal | rs442495 | C | T | -0.01372 | 0.00226 | 1.22E-09 | -0.0012 | 0.0009 | 1.79E-01 |
| AD | Lateral orbitofrontal | rs4575098 | A | G | 0.01641 | 0.00258 | 1.90E-10 | -0.0010 | 0.0010 | 2.99E-01 |
| AD | Lateral orbitofrontal | rs4663105 | C | A | 0.03110 | 0.00222 | 1.45E-44 | -0.0006 | 0.0009 | 4.82E-01 |
| AD | Lateral orbitofrontal | rs59735493 | A | G | -0.01299 | 0.00236 | 3.73E-08 | -0.0030 | 0.0010 | 2.05E-03 |
| AD | Lateral orbitofrontal | rs6014724 | G | A | -0.02289 | 0.00369 | 5.38E-10 | 0.0016 | 0.0015 | 2.88E-01 |
| AD | Lateral orbitofrontal | rs6448453 | A | G | 0.01470 | 0.00245 | 1.98E-09 | 0.0001 | 0.0010 | 9.32E-01 |
| AD | Lateral orbitofrontal | rs679515 | T | C | 0.02542 | 0.00286 | 6.83E-19 | 0.0010 | 0.0011 | 4.03E-01 |
| AD | Lateral orbitofrontal | rs755951 | C | A | 0.01500 | 0.00221 | 1.13E-11 | -0.0006 | 0.0009 | 4.95E-01 |
| AD | Lateral orbitofrontal | rs7810606 | T | C | -0.01452 | 0.00218 | 2.89E-11 | -0.0002 | 0.0009 | 7.81E-01 |
| AD | Lateral orbitofrontal | rs846881 | C | A | -0.01737 | 0.00269 | 9.89E-11 | -0.0002 | 0.0012 | 8.95E-01 |
| AD | Lateral orbitofrontal | rs867611 | G | A | -0.02043 | 0.00232 | 1.48E-18 | 0.0001 | 0.0009 | 9.16E-01 |
| AD | Lateral orbitofrontal | rs9381563 | C | T | 0.01445 | 0.00227 | 1.99E-10 | 0.0011 | 0.0009 | 2.33E-01 |
| AD | Medial orbitofrontal | rs10933431 | G | C | -0.01544 | 0.00251 | 7.62E-10 | -0.0009 | 0.0013 | 4.99E-01 |
| AD | Medial orbitofrontal | rs111278892 | G | C | 0.01991 | 0.00305 | 6.67E-11 | 0.0031 | 0.0016 | 4.43E-02 |
| AD | Medial orbitofrontal | rs11218343 | C | T | -0.03593 | 0.00526 | 8.12E-12 | 0.0052 | 0.0026 | 4.52E-02 |
| AD | Medial orbitofrontal | rs11257238 | C | T | 0.01294 | 0.00226 | 1.04E-08 | 0.0008 | 0.0011 | 4.78E-01 |
| AD | Medial orbitofrontal | rs113260531 | A | G | 0.01999 | 0.00325 | 7.91E-10 | -0.0006 | 0.0016 | 6.86E-01 |
| AD | Medial orbitofrontal | rs118170342 | C | T | 0.14754 | 0.00570 | 7.93E-148 | -0.0039 | 0.0032 | 2.21E-01 |
| AD | Medial orbitofrontal | rs12590654 | A | G | -0.01483 | 0.00231 | 1.32E-10 | 0.0013 | 0.0011 | 2.15E-01 |
| AD | Medial orbitofrontal | rs1859788 | A | G | -0.01840 | 0.00231 | 1.80E-15 | -0.0009 | 0.0011 | 4.44E-01 |
| AD | Medial orbitofrontal | rs204473 | A | G | -0.04167 | 0.00700 | 2.58E-09 | 0.0008 | 0.0033 | 8.09E-01 |
| AD | Medial orbitofrontal | rs2081545 | A | C | -0.01787 | 0.00223 | 1.11E-15 | 0.0012 | 0.0010 | 2.51E-01 |
| AD | Medial orbitofrontal | rs28394864 | A | G | 0.01230 | 0.00218 | 1.68E-08 | -0.0012 | 0.0010 | 2.36E-01 |
| AD | Medial orbitofrontal | rs28399657 | G | A | -0.05464 | 0.00658 | 9.82E-17 | -0.0005 | 0.0030 | 8.66E-01 |
| AD | Medial orbitofrontal | rs41289512 | G | C | 0.20630 | 0.00578 | 1.00E-200 | 0.0005 | 0.0031 | 8.75E-01 |
| AD | Medial orbitofrontal | rs41290120 | A | G | -0.09905 | 0.00578 | 7.14E-66 | 0.0012 | 0.0026 | 6.28E-01 |
| AD | Medial orbitofrontal | rs4236673 | A | G | -0.02016 | 0.00223 | 1.48E-19 | -0.0014 | 0.0011 | 1.97E-01 |
| AD | Medial orbitofrontal | rs442495 | C | T | -0.01372 | 0.00226 | 1.22E-09 | -0.0014 | 0.0011 | 1.91E-01 |
| AD | Medial orbitofrontal | rs4575098 | A | G | 0.01641 | 0.00258 | 1.90E-10 | -0.0013 | 0.0012 | 2.84E-01 |
| AD | Medial orbitofrontal | rs4663105 | C | A | 0.03110 | 0.00222 | 1.45E-44 | 0.0004 | 0.0011 | 7.33E-01 |
| AD | Medial orbitofrontal | rs59735493 | A | G | -0.01299 | 0.00236 | 3.73E-08 | -0.0014 | 0.0011 | 1.85E-01 |
| AD | Medial orbitofrontal | rs6014724 | G | A | -0.02289 | 0.00369 | 5.38E-10 | 0.0014 | 0.0019 | 4.47E-01 |
| AD | Medial orbitofrontal | rs6448453 | A | G | 0.01470 | 0.00245 | 1.98E-09 | 0.0009 | 0.0012 | 4.27E-01 |
| AD | Medial orbitofrontal | rs679515 | T | C | 0.02542 | 0.00286 | 6.83E-19 | 0.0002 | 0.0013 | 8.99E-01 |
| AD | Medial orbitofrontal | rs755951 | C | A | 0.01500 | 0.00221 | 1.13E-11 | -0.0013 | 0.0010 | 2.10E-01 |
| AD | Medial orbitofrontal | rs7810606 | T | C | -0.01452 | 0.00218 | 2.89E-11 | 0.0001 | 0.0011 | 9.10E-01 |
| AD | Medial orbitofrontal | rs846881 | C | A | -0.01737 | 0.00269 | 9.89E-11 | 0.0018 | 0.0014 | 1.98E-01 |
| AD | Medial orbitofrontal | rs867611 | G | A | -0.02043 | 0.00232 | 1.48E-18 | 0.0006 | 0.0011 | 5.58E-01 |
| AD | Medial orbitofrontal | rs9381563 | C | T | 0.01445 | 0.00227 | 1.99E-10 | 0.0009 | 0.0011 | 4.18E-01 |
| AD | paracentral | rs10933431 | G | C | -0.01544 | 0.00251 | 7.62E-10 | -0.0011 | 0.0011 | 2.96E-01 |
| AD | paracentral | rs111278892 | G | C | 0.01991 | 0.00305 | 6.67E-11 | -0.0002 | 0.0013 | 8.70E-01 |
| AD | paracentral | rs11218343 | C | T | -0.03593 | 0.00526 | 8.12E-12 | -0.0020 | 0.0021 | 3.34E-01 |
| AD | paracentral | rs11257238 | C | T | 0.01294 | 0.00226 | 1.04E-08 | 0.0009 | 0.0009 | 3.15E-01 |
| AD | paracentral | rs113260531 | A | G | 0.01999 | 0.00325 | 7.91E-10 | -0.0011 | 0.0013 | 4.01E-01 |
| AD | paracentral | rs118170342 | C | T | 0.14754 | 0.00570 | 7.93E-148 | 0.0013 | 0.0026 | 6.37E-01 |
| AD | paracentral | rs12590654 | A | G | -0.01483 | 0.00231 | 1.32E-10 | -0.0005 | 0.0009 | 6.24E-01 |
| AD | paracentral | rs1859788 | A | G | -0.01840 | 0.00231 | 1.80E-15 | 0.0027 | 0.0009 | 4.46E-03 |
| AD | paracentral | rs204473 | A | G | -0.04167 | 0.00700 | 2.58E-09 | -0.0002 | 0.0028 | 9.47E-01 |
| AD | paracentral | rs2081545 | A | C | -0.01787 | 0.00223 | 1.11E-15 | -0.0002 | 0.0009 | 7.81E-01 |
| AD | paracentral | rs28394864 | A | G | 0.01230 | 0.00218 | 1.68E-08 | -0.0006 | 0.0009 | 4.99E-01 |
| AD | paracentral | rs28399657 | G | A | -0.05464 | 0.00658 | 9.82E-17 | -0.0026 | 0.0025 | 2.96E-01 |
| AD | paracentral | rs41289512 | G | C | 0.20630 | 0.00578 | 1.00E-200 | 0.0006 | 0.0026 | 8.24E-01 |
| AD | paracentral | rs41290120 | A | G | -0.09905 | 0.00578 | 7.14E-66 | -0.0002 | 0.0021 | 9.27E-01 |
| AD | paracentral | rs4236673 | A | G | -0.02016 | 0.00223 | 1.48E-19 | 0.0006 | 0.0009 | 4.98E-01 |
| AD | paracentral | rs442495 | C | T | -0.01372 | 0.00226 | 1.22E-09 | 0.0010 | 0.0009 | 2.65E-01 |
| AD | paracentral | rs4575098 | A | G | 0.01641 | 0.00258 | 1.90E-10 | -0.0008 | 0.0010 | 4.21E-01 |
| AD | paracentral | rs4663105 | C | A | 0.03110 | 0.00222 | 1.45E-44 | 0.0016 | 0.0009 | 5.64E-02 |
| AD | paracentral | rs59735493 | A | G | -0.01299 | 0.00236 | 3.73E-08 | 0.0004 | 0.0009 | 6.98E-01 |
| AD | paracentral | rs6014724 | G | A | -0.02289 | 0.00369 | 5.38E-10 | 0.0002 | 0.0015 | 8.96E-01 |
| AD | paracentral | rs6448453 | A | G | 0.01470 | 0.00245 | 1.98E-09 | 0.0000 | 0.0010 | 9.67E-01 |
| AD | paracentral | rs679515 | T | C | 0.02542 | 0.00286 | 6.83E-19 | -0.0009 | 0.0011 | 4.07E-01 |
| AD | paracentral | rs755951 | C | A | 0.01500 | 0.00221 | 1.13E-11 | 0.0022 | 0.0009 | 9.61E-03 |
| AD | paracentral | rs7810606 | T | C | -0.01452 | 0.00218 | 2.89E-11 | 0.0005 | 0.0009 | 5.96E-01 |
| AD | paracentral | rs846881 | C | A | -0.01737 | 0.00269 | 9.89E-11 | -0.0002 | 0.0012 | 8.73E-01 |
| AD | paracentral | rs867611 | G | A | -0.02043 | 0.00232 | 1.48E-18 | -0.0015 | 0.0009 | 7.76E-02 |
| AD | paracentral | rs9381563 | C | T | 0.01445 | 0.00227 | 1.99E-10 | 0.0003 | 0.0009 | 7.06E-01 |
| AD | Pars opercularis | rs10933431 | G | C | -0.01544 | 0.00251 | 7.62E-10 | 0.0012 | 0.0009 | 1.93E-01 |
| AD | Pars opercularis | rs111278892 | G | C | 0.01991 | 0.00305 | 6.67E-11 | 0.0004 | 0.0011 | 7.26E-01 |
| AD | Pars opercularis | rs11218343 | C | T | -0.03593 | 0.00526 | 8.12E-12 | 0.0010 | 0.0018 | 5.86E-01 |
| AD | Pars opercularis | rs11257238 | C | T | 0.01294 | 0.00226 | 1.04E-08 | 0.0012 | 0.0008 | 1.22E-01 |
| AD | Pars opercularis | rs113260531 | A | G | 0.01999 | 0.00325 | 7.91E-10 | -0.0001 | 0.0011 | 9.61E-01 |
| AD | Pars opercularis | rs118170342 | C | T | 0.14754 | 0.00570 | 7.93E-148 | 0.0011 | 0.0023 | 6.12E-01 |
| AD | Pars opercularis | rs12590654 | A | G | -0.01483 | 0.00231 | 1.32E-10 | 0.0010 | 0.0008 | 1.89E-01 |
| AD | Pars opercularis | rs1859788 | A | G | -0.01840 | 0.00231 | 1.80E-15 | -0.0012 | 0.0008 | 1.47E-01 |
| AD | Pars opercularis | rs204473 | A | G | -0.04167 | 0.00700 | 2.58E-09 | 0.0008 | 0.0024 | 7.42E-01 |
| AD | Pars opercularis | rs2081545 | A | C | -0.01787 | 0.00223 | 1.11E-15 | 0.0000 | 0.0007 | 9.76E-01 |
| AD | Pars opercularis | rs28394864 | A | G | 0.01230 | 0.00218 | 1.68E-08 | 0.0019 | 0.0007 | 1.18E-02 |
| AD | Pars opercularis | rs28399657 | G | A | -0.05464 | 0.00658 | 9.82E-17 | -0.0022 | 0.0022 | 3.08E-01 |
| AD | Pars opercularis | rs41289512 | G | C | 0.20630 | 0.00578 | 1.00E-200 | -0.0030 | 0.0022 | 1.77E-01 |
| AD | Pars opercularis | rs41290120 | A | G | -0.09905 | 0.00578 | 7.14E-66 | -0.0009 | 0.0018 | 6.30E-01 |
| AD | Pars opercularis | rs4236673 | A | G | -0.02016 | 0.00223 | 1.48E-19 | -0.0006 | 0.0007 | 3.87E-01 |
| AD | Pars opercularis | rs442495 | C | T | -0.01372 | 0.00226 | 1.22E-09 | -0.0007 | 0.0008 | 3.80E-01 |
| AD | Pars opercularis | rs4575098 | A | G | 0.01641 | 0.00258 | 1.90E-10 | -0.0016 | 0.0008 | 5.17E-02 |
| AD | Pars opercularis | rs4663105 | C | A | 0.03110 | 0.00222 | 1.45E-44 | -0.0006 | 0.0007 | 3.92E-01 |
| AD | Pars opercularis | rs59735493 | A | G | -0.01299 | 0.00236 | 3.73E-08 | -0.0005 | 0.0008 | 4.80E-01 |
| AD | Pars opercularis | rs6014724 | G | A | -0.02289 | 0.00369 | 5.38E-10 | 0.0005 | 0.0013 | 6.77E-01 |
| AD | Pars opercularis | rs6448453 | A | G | 0.01470 | 0.00245 | 1.98E-09 | -0.0010 | 0.0008 | 2.24E-01 |
| AD | Pars opercularis | rs679515 | T | C | 0.02542 | 0.00286 | 6.83E-19 | -0.0006 | 0.0009 | 5.48E-01 |
| AD | Pars opercularis | rs755951 | C | A | 0.01500 | 0.00221 | 1.13E-11 | -0.0012 | 0.0007 | 1.22E-01 |
| AD | Pars opercularis | rs7810606 | T | C | -0.01452 | 0.00218 | 2.89E-11 | -0.0005 | 0.0007 | 5.18E-01 |
| AD | Pars opercularis | rs846881 | C | A | -0.01737 | 0.00269 | 9.89E-11 | -0.0011 | 0.0010 | 2.64E-01 |
| AD | Pars opercularis | rs867611 | G | A | -0.02043 | 0.00232 | 1.48E-18 | 0.0007 | 0.0008 | 3.42E-01 |
| AD | Pars opercularis | rs9381563 | C | T | 0.01445 | 0.00227 | 1.99E-10 | -0.0001 | 0.0007 | 8.75E-01 |
| AD | Pars orbitalis | rs10933431 | G | C | -0.01544 | 0.00251 | 7.62E-10 | -0.0008 | 0.0015 | 5.75E-01 |
| AD | Pars orbitalis | rs111278892 | G | C | 0.01991 | 0.00305 | 6.67E-11 | -0.0005 | 0.0017 | 7.54E-01 |
| AD | Pars orbitalis | rs11218343 | C | T | -0.03593 | 0.00526 | 8.12E-12 | 0.0041 | 0.0029 | 1.53E-01 |
| AD | Pars orbitalis | rs11257238 | C | T | 0.01294 | 0.00226 | 1.04E-08 | -0.0005 | 0.0012 | 6.59E-01 |
| AD | Pars orbitalis | rs113260531 | A | G | 0.01999 | 0.00325 | 7.91E-10 | -0.0006 | 0.0017 | 7.37E-01 |
| AD | Pars orbitalis | rs118170342 | C | T | 0.14754 | 0.00570 | 7.93E-148 | 0.0010 | 0.0035 | 7.80E-01 |
| AD | Pars orbitalis | rs12590654 | A | G | -0.01483 | 0.00231 | 1.32E-10 | -0.0002 | 0.0012 | 8.45E-01 |
| AD | Pars orbitalis | rs1859788 | A | G | -0.01840 | 0.00231 | 1.80E-15 | 0.0011 | 0.0013 | 4.04E-01 |
| AD | Pars orbitalis | rs204473 | A | G | -0.04167 | 0.00700 | 2.58E-09 | 0.0028 | 0.0037 | 4.53E-01 |
| AD | Pars orbitalis | rs2081545 | A | C | -0.01787 | 0.00223 | 1.11E-15 | -0.0005 | 0.0012 | 6.70E-01 |
| AD | Pars orbitalis | rs28394864 | A | G | 0.01230 | 0.00218 | 1.68E-08 | 0.0003 | 0.0011 | 7.83E-01 |
| AD | Pars orbitalis | rs28399657 | G | A | -0.05464 | 0.00658 | 9.82E-17 | 0.0047 | 0.0033 | 1.60E-01 |
| AD | Pars orbitalis | rs41289512 | G | C | 0.20630 | 0.00578 | 1.00E-200 | -0.0019 | 0.0035 | 5.89E-01 |
| AD | Pars orbitalis | rs41290120 | A | G | -0.09905 | 0.00578 | 7.14E-66 | -0.0001 | 0.0029 | 9.83E-01 |
| AD | Pars orbitalis | rs4236673 | A | G | -0.02016 | 0.00223 | 1.48E-19 | 0.0009 | 0.0012 | 4.33E-01 |
| AD | Pars orbitalis | rs442495 | C | T | -0.01372 | 0.00226 | 1.22E-09 | -0.0006 | 0.0012 | 6.42E-01 |
| AD | Pars orbitalis | rs4575098 | A | G | 0.01641 | 0.00258 | 1.90E-10 | -0.0004 | 0.0013 | 7.71E-01 |
| AD | Pars orbitalis | rs4663105 | C | A | 0.03110 | 0.00222 | 1.45E-44 | -0.0006 | 0.0012 | 6.18E-01 |
| AD | Pars orbitalis | rs59735493 | A | G | -0.01299 | 0.00236 | 3.73E-08 | 0.0002 | 0.0012 | 8.47E-01 |
| AD | Pars orbitalis | rs6014724 | G | A | -0.02289 | 0.00369 | 5.38E-10 | -0.0051 | 0.0020 | 1.27E-02 |
| AD | Pars orbitalis | rs6448453 | A | G | 0.01470 | 0.00245 | 1.98E-09 | 0.0006 | 0.0012 | 6.48E-01 |
| AD | Pars orbitalis | rs679515 | T | C | 0.02542 | 0.00286 | 6.83E-19 | -0.0007 | 0.0015 | 6.51E-01 |
| AD | Pars orbitalis | rs755951 | C | A | 0.01500 | 0.00221 | 1.13E-11 | 0.0000 | 0.0012 | 9.94E-01 |
| AD | Pars orbitalis | rs7810606 | T | C | -0.01452 | 0.00218 | 2.89E-11 | 0.0016 | 0.0011 | 1.67E-01 |
| AD | Pars orbitalis | rs846881 | C | A | -0.01737 | 0.00269 | 9.89E-11 | -0.0013 | 0.0015 | 3.95E-01 |
| AD | Pars orbitalis | rs867611 | G | A | -0.02043 | 0.00232 | 1.48E-18 | 0.0006 | 0.0012 | 6.13E-01 |
| AD | Pars orbitalis | rs9381563 | C | T | 0.01445 | 0.00227 | 1.99E-10 | 0.0010 | 0.0012 | 3.97E-01 |
| AD | Pars triangularis | rs10933431 | G | C | -0.01544 | 0.00251 | 7.62E-10 | -0.0002 | 0.0010 | 8.42E-01 |
| AD | Pars triangularis | rs111278892 | G | C | 0.01991 | 0.00305 | 6.67E-11 | -0.0003 | 0.0012 | 8.22E-01 |
| AD | Pars triangularis | rs11218343 | C | T | -0.03593 | 0.00526 | 8.12E-12 | -0.0002 | 0.0020 | 9.09E-01 |
| AD | Pars triangularis | rs11257238 | C | T | 0.01294 | 0.00226 | 1.04E-08 | -0.0003 | 0.0008 | 7.05E-01 |
| AD | Pars triangularis | rs113260531 | A | G | 0.01999 | 0.00325 | 7.91E-10 | -0.0025 | 0.0012 | 3.47E-02 |
| AD | Pars triangularis | rs118170342 | C | T | 0.14754 | 0.00570 | 7.93E-148 | -0.0007 | 0.0024 | 7.89E-01 |
| AD | Pars triangularis | rs12590654 | A | G | -0.01483 | 0.00231 | 1.32E-10 | -0.0013 | 0.0008 | 1.21E-01 |
| AD | Pars triangularis | rs1859788 | A | G | -0.01840 | 0.00231 | 1.80E-15 | -0.0003 | 0.0009 | 7.30E-01 |
| AD | Pars triangularis | rs204473 | A | G | -0.04167 | 0.00700 | 2.58E-09 | 0.0019 | 0.0025 | 4.66E-01 |
| AD | Pars triangularis | rs2081545 | A | C | -0.01787 | 0.00223 | 1.11E-15 | 0.0011 | 0.0008 | 1.39E-01 |
| AD | Pars triangularis | rs28394864 | A | G | 0.01230 | 0.00218 | 1.68E-08 | 0.0013 | 0.0008 | 1.02E-01 |
| AD | Pars triangularis | rs28399657 | G | A | -0.05464 | 0.00658 | 9.82E-17 | 0.0023 | 0.0023 | 3.19E-01 |
| AD | Pars triangularis | rs41289512 | G | C | 0.20630 | 0.00578 | 1.00E-200 | 0.0004 | 0.0024 | 8.81E-01 |
| AD | Pars triangularis | rs41290120 | A | G | -0.09905 | 0.00578 | 7.14E-66 | -0.0023 | 0.0020 | 2.47E-01 |
| AD | Pars triangularis | rs4236673 | A | G | -0.02016 | 0.00223 | 1.48E-19 | 0.0006 | 0.0008 | 4.85E-01 |
| AD | Pars triangularis | rs442495 | C | T | -0.01372 | 0.00226 | 1.22E-09 | 0.0001 | 0.0008 | 8.69E-01 |
| AD | Pars triangularis | rs4575098 | A | G | 0.01641 | 0.00258 | 1.90E-10 | -0.0005 | 0.0009 | 6.04E-01 |
| AD | Pars triangularis | rs4663105 | C | A | 0.03110 | 0.00222 | 1.45E-44 | 0.0000 | 0.0008 | 9.62E-01 |
| AD | Pars triangularis | rs59735493 | A | G | -0.01299 | 0.00236 | 3.73E-08 | -0.0011 | 0.0008 | 2.14E-01 |
| AD | Pars triangularis | rs6014724 | G | A | -0.02289 | 0.00369 | 5.38E-10 | 0.0006 | 0.0014 | 6.49E-01 |
| AD | Pars triangularis | rs6448453 | A | G | 0.01470 | 0.00245 | 1.98E-09 | -0.0001 | 0.0009 | 8.90E-01 |
| AD | Pars triangularis | rs679515 | T | C | 0.02542 | 0.00286 | 6.83E-19 | -0.0007 | 0.0010 | 5.14E-01 |
| AD | Pars triangularis | rs755951 | C | A | 0.01500 | 0.00221 | 1.13E-11 | -0.0014 | 0.0008 | 6.78E-02 |
| AD | Pars triangularis | rs7810606 | T | C | -0.01452 | 0.00218 | 2.89E-11 | 0.0001 | 0.0008 | 8.77E-01 |
| AD | Pars triangularis | rs846881 | C | A | -0.01737 | 0.00269 | 9.89E-11 | 0.0010 | 0.0011 | 3.56E-01 |
| AD | Pars triangularis | rs867611 | G | A | -0.02043 | 0.00232 | 1.48E-18 | -0.0006 | 0.0008 | 5.07E-01 |
| AD | Pars triangularis | rs9381563 | C | T | 0.01445 | 0.00227 | 1.99E-10 | 0.0001 | 0.0008 | 9.37E-01 |
| AD | precentral | rs10933431 | G | C | -0.01544 | 0.00251 | 7.62E-10 | 0.0011 | 0.0009 | 2.56E-01 |
| AD | precentral | rs111278892 | G | C | 0.01991 | 0.00305 | 6.67E-11 | -0.0011 | 0.0011 | 3.15E-01 |
| AD | precentral | rs11218343 | C | T | -0.03593 | 0.00526 | 8.12E-12 | -0.0013 | 0.0019 | 4.76E-01 |
| AD | precentral | rs11257238 | C | T | 0.01294 | 0.00226 | 1.04E-08 | 0.0010 | 0.0008 | 1.89E-01 |
| AD | precentral | rs113260531 | A | G | 0.01999 | 0.00325 | 7.91E-10 | -0.0016 | 0.0011 | 1.56E-01 |
| AD | precentral | rs118170342 | C | T | 0.14754 | 0.00570 | 7.93E-148 | -0.0007 | 0.0024 | 7.78E-01 |
| AD | precentral | rs12590654 | A | G | -0.01483 | 0.00231 | 1.32E-10 | -0.0002 | 0.0008 | 7.63E-01 |
| AD | precentral | rs1859788 | A | G | -0.01840 | 0.00231 | 1.80E-15 | 0.0011 | 0.0008 | 1.62E-01 |
| AD | precentral | rs204473 | A | G | -0.04167 | 0.00700 | 2.58E-09 | -0.0010 | 0.0024 | 6.94E-01 |
| AD | precentral | rs2081545 | A | C | -0.01787 | 0.00223 | 1.11E-15 | -0.0013 | 0.0007 | 6.62E-02 |
| AD | precentral | rs28394864 | A | G | 0.01230 | 0.00218 | 1.68E-08 | -0.0002 | 0.0007 | 7.77E-01 |
| AD | precentral | rs28399657 | G | A | -0.05464 | 0.00658 | 9.82E-17 | 0.0019 | 0.0022 | 3.98E-01 |
| AD | precentral | rs41289512 | G | C | 0.20630 | 0.00578 | 1.00E-200 | -0.0018 | 0.0024 | 4.46E-01 |
| AD | precentral | rs41290120 | A | G | -0.09905 | 0.00578 | 7.14E-66 | -0.0019 | 0.0019 | 3.19E-01 |
| AD | precentral | rs4236673 | A | G | -0.02016 | 0.00223 | 1.48E-19 | 0.0004 | 0.0008 | 6.27E-01 |
| AD | precentral | rs442495 | C | T | -0.01372 | 0.00226 | 1.22E-09 | -0.0001 | 0.0008 | 9.38E-01 |
| AD | precentral | rs4575098 | A | G | 0.01641 | 0.00258 | 1.90E-10 | 0.0002 | 0.0009 | 8.25E-01 |
| AD | precentral | rs4663105 | C | A | 0.03110 | 0.00222 | 1.45E-44 | 0.0010 | 0.0008 | 2.04E-01 |
| AD | precentral | rs59735493 | A | G | -0.01299 | 0.00236 | 3.73E-08 | -0.0005 | 0.0008 | 4.94E-01 |
| AD | precentral | rs6014724 | G | A | -0.02289 | 0.00369 | 5.38E-10 | -0.0011 | 0.0013 | 3.88E-01 |
| AD | precentral | rs6448453 | A | G | 0.01470 | 0.00245 | 1.98E-09 | -0.0005 | 0.0008 | 5.45E-01 |
| AD | precentral | rs679515 | T | C | 0.02542 | 0.00286 | 6.83E-19 | -0.0014 | 0.0010 | 1.38E-01 |
| AD | precentral | rs755951 | C | A | 0.01500 | 0.00221 | 1.13E-11 | 0.0004 | 0.0007 | 6.20E-01 |
| AD | precentral | rs7810606 | T | C | -0.01452 | 0.00218 | 2.89E-11 | -0.0002 | 0.0008 | 8.06E-01 |
| AD | precentral | rs846881 | C | A | -0.01737 | 0.00269 | 9.89E-11 | -0.0004 | 0.0010 | 6.95E-01 |
| AD | precentral | rs867611 | G | A | -0.02043 | 0.00232 | 1.48E-18 | -0.0019 | 0.0008 | 1.95E-02 |
| AD | precentral | rs9381563 | C | T | 0.01445 | 0.00227 | 1.99E-10 | -0.0001 | 0.0008 | 8.57E-01 |
| AD | Rostral anterior cingulate | rs10933431 | G | C | -0.01544 | 0.00251 | 7.62E-10 | -0.0004 | 0.0018 | 8.36E-01 |
| AD | Rostral anterior cingulate | rs111278892 | G | C | 0.01991 | 0.00305 | 6.67E-11 | 0.0043 | 0.0021 | 4.24E-02 |
| AD | Rostral anterior cingulate | rs11218343 | C | T | -0.03593 | 0.00526 | 8.12E-12 | 0.0010 | 0.0035 | 7.72E-01 |
| AD | Rostral anterior cingulate | rs11257238 | C | T | 0.01294 | 0.00226 | 1.04E-08 | -0.0006 | 0.0015 | 6.96E-01 |
| AD | Rostral anterior cingulate | rs113260531 | A | G | 0.01999 | 0.00325 | 7.91E-10 | -0.0005 | 0.0021 | 8.21E-01 |
| AD | Rostral anterior cingulate | rs118170342 | C | T | 0.14754 | 0.00570 | 7.93E-148 | 0.0013 | 0.0043 | 7.60E-01 |
| AD | Rostral anterior cingulate | rs12590654 | A | G | -0.01483 | 0.00231 | 1.32E-10 | -0.0014 | 0.0015 | 3.35E-01 |
| AD | Rostral anterior cingulate | rs1859788 | A | G | -0.01840 | 0.00231 | 1.80E-15 | -0.0010 | 0.0015 | 5.12E-01 |
| AD | Rostral anterior cingulate | rs204473 | A | G | -0.04167 | 0.00700 | 2.58E-09 | -0.0012 | 0.0046 | 7.86E-01 |
| AD | Rostral anterior cingulate | rs2081545 | A | C | -0.01787 | 0.00223 | 1.11E-15 | 0.0002 | 0.0014 | 8.72E-01 |
| AD | Rostral anterior cingulate | rs28394864 | A | G | 0.01230 | 0.00218 | 1.68E-08 | -0.0009 | 0.0014 | 5.41E-01 |
| AD | Rostral anterior cingulate | rs28399657 | G | A | -0.05464 | 0.00658 | 9.82E-17 | -0.0049 | 0.0040 | 2.27E-01 |
| AD | Rostral anterior cingulate | rs41289512 | G | C | 0.20630 | 0.00578 | 1.00E-200 | 0.0067 | 0.0042 | 1.10E-01 |
| AD | Rostral anterior cingulate | rs41290120 | A | G | -0.09905 | 0.00578 | 7.14E-66 | 0.0064 | 0.0035 | 6.78E-02 |
| AD | Rostral anterior cingulate | rs4236673 | A | G | -0.02016 | 0.00223 | 1.48E-19 | -0.0013 | 0.0014 | 3.43E-01 |
| AD | Rostral anterior cingulate | rs442495 | C | T | -0.01372 | 0.00226 | 1.22E-09 | -0.0008 | 0.0015 | 5.83E-01 |
| AD | Rostral anterior cingulate | rs4575098 | A | G | 0.01641 | 0.00258 | 1.90E-10 | -0.0034 | 0.0016 | 3.20E-02 |
| AD | Rostral anterior cingulate | rs4663105 | C | A | 0.03110 | 0.00222 | 1.45E-44 | 0.0017 | 0.0015 | 2.52E-01 |
| AD | Rostral anterior cingulate | rs59735493 | A | G | -0.01299 | 0.00236 | 3.73E-08 | -0.0020 | 0.0015 | 1.81E-01 |
| AD | Rostral anterior cingulate | rs6014724 | G | A | -0.02289 | 0.00369 | 5.38E-10 | -0.0001 | 0.0025 | 9.77E-01 |
| AD | Rostral anterior cingulate | rs6448453 | A | G | 0.01470 | 0.00245 | 1.98E-09 | -0.0012 | 0.0016 | 4.49E-01 |
| AD | Rostral anterior cingulate | rs679515 | T | C | 0.02542 | 0.00286 | 6.83E-19 | -0.0015 | 0.0018 | 3.96E-01 |
| AD | Rostral anterior cingulate | rs755951 | C | A | 0.01500 | 0.00221 | 1.13E-11 | -0.0025 | 0.0014 | 7.92E-02 |
| AD | Rostral anterior cingulate | rs7810606 | T | C | -0.01452 | 0.00218 | 2.89E-11 | -0.0020 | 0.0014 | 1.62E-01 |
| AD | Rostral anterior cingulate | rs846881 | C | A | -0.01737 | 0.00269 | 9.89E-11 | 0.0022 | 0.0019 | 2.37E-01 |
| AD | Rostral anterior cingulate | rs867611 | G | A | -0.02043 | 0.00232 | 1.48E-18 | 0.0016 | 0.0015 | 2.95E-01 |
| AD | Rostral anterior cingulate | rs9381563 | C | T | 0.01445 | 0.00227 | 1.99E-10 | 0.0001 | 0.0015 | 9.64E-01 |
| AD | Rostral middle frontal | rs10933431 | G | C | -0.01544 | 0.00251 | 7.62E-10 | -0.0010 | 0.0008 | 2.40E-01 |
| AD | Rostral middle frontal | rs111278892 | G | C | 0.01991 | 0.00305 | 6.67E-11 | -0.0003 | 0.0010 | 7.74E-01 |
| AD | Rostral middle frontal | rs11218343 | C | T | -0.03593 | 0.00526 | 8.12E-12 | -0.0003 | 0.0016 | 8.48E-01 |
| AD | Rostral middle frontal | rs11257238 | C | T | 0.01294 | 0.00226 | 1.04E-08 | -0.0004 | 0.0007 | 5.78E-01 |
| AD | Rostral middle frontal | rs113260531 | A | G | 0.01999 | 0.00325 | 7.91E-10 | -0.0006 | 0.0009 | 5.33E-01 |
| AD | Rostral middle frontal | rs118170342 | C | T | 0.14754 | 0.00570 | 7.93E-148 | -0.0048 | 0.0020 | 1.62E-02 |
| AD | Rostral middle frontal | rs12590654 | A | G | -0.01483 | 0.00231 | 1.32E-10 | 0.0000 | 0.0007 | 9.50E-01 |
| AD | Rostral middle frontal | rs1859788 | A | G | -0.01840 | 0.00231 | 1.80E-15 | 0.0003 | 0.0007 | 7.04E-01 |
| AD | Rostral middle frontal | rs204473 | A | G | -0.04167 | 0.00700 | 2.58E-09 | 0.0013 | 0.0021 | 5.22E-01 |
| AD | Rostral middle frontal | rs2081545 | A | C | -0.01787 | 0.00223 | 1.11E-15 | -0.0002 | 0.0007 | 7.67E-01 |
| AD | Rostral middle frontal | rs28394864 | A | G | 0.01230 | 0.00218 | 1.68E-08 | 0.0008 | 0.0007 | 2.11E-01 |
| AD | Rostral middle frontal | rs28399657 | G | A | -0.05464 | 0.00658 | 9.82E-17 | 0.0017 | 0.0019 | 3.80E-01 |
| AD | Rostral middle frontal | rs41289512 | G | C | 0.20630 | 0.00578 | 1.00E-200 | 0.0013 | 0.0020 | 5.22E-01 |
| AD | Rostral middle frontal | rs41290120 | A | G | -0.09905 | 0.00578 | 7.14E-66 | 0.0011 | 0.0016 | 4.96E-01 |
| AD | Rostral middle frontal | rs4236673 | A | G | -0.02016 | 0.00223 | 1.48E-19 | -0.0003 | 0.0007 | 6.74E-01 |
| AD | Rostral middle frontal | rs442495 | C | T | -0.01372 | 0.00226 | 1.22E-09 | -0.0014 | 0.0007 | 4.13E-02 |
| AD | Rostral middle frontal | rs4575098 | A | G | 0.01641 | 0.00258 | 1.90E-10 | -0.0006 | 0.0008 | 4.50E-01 |
| AD | Rostral middle frontal | rs4663105 | C | A | 0.03110 | 0.00222 | 1.45E-44 | 0.0000 | 0.0007 | 9.89E-01 |
| AD | Rostral middle frontal | rs59735493 | A | G | -0.01299 | 0.00236 | 3.73E-08 | -0.0009 | 0.0007 | 2.11E-01 |
| AD | Rostral middle frontal | rs6014724 | G | A | -0.02289 | 0.00369 | 5.38E-10 | 0.0006 | 0.0012 | 5.83E-01 |
| AD | Rostral middle frontal | rs6448453 | A | G | 0.01470 | 0.00245 | 1.98E-09 | 0.0002 | 0.0007 | 7.21E-01 |
| AD | Rostral middle frontal | rs679515 | T | C | 0.02542 | 0.00286 | 6.83E-19 | -0.0009 | 0.0009 | 2.78E-01 |
| AD | Rostral middle frontal | rs755951 | C | A | 0.01500 | 0.00221 | 1.13E-11 | -0.0012 | 0.0007 | 7.31E-02 |
| AD | Rostral middle frontal | rs7810606 | T | C | -0.01452 | 0.00218 | 2.89E-11 | -0.0012 | 0.0007 | 8.28E-02 |
| AD | Rostral middle frontal | rs846881 | C | A | -0.01737 | 0.00269 | 9.89E-11 | 0.0001 | 0.0009 | 9.55E-01 |
| AD | Rostral middle frontal | rs867611 | G | A | -0.02043 | 0.00232 | 1.48E-18 | 0.0001 | 0.0007 | 8.37E-01 |
| AD | Rostral middle frontal | rs9381563 | C | T | 0.01445 | 0.00227 | 1.99E-10 | 0.0003 | 0.0007 | 6.42E-01 |
| AD | Superior frontal | rs10933431 | G | C | -0.01544 | 0.00251 | 7.62E-10 | 0.0003 | 0.0009 | 6.99E-01 |
| AD | Superior frontal | rs111278892 | G | C | 0.01991 | 0.00305 | 6.67E-11 | -0.0006 | 0.0010 | 5.92E-01 |
| AD | Superior frontal | rs11218343 | C | T | -0.03593 | 0.00526 | 8.12E-12 | -0.0006 | 0.0017 | 7.11E-01 |
| AD | Superior frontal | rs11257238 | C | T | 0.01294 | 0.00226 | 1.04E-08 | -0.0008 | 0.0007 | 2.59E-01 |
| AD | Superior frontal | rs113260531 | A | G | 0.01999 | 0.00325 | 7.91E-10 | -0.0003 | 0.0010 | 7.50E-01 |
| AD | Superior frontal | rs118170342 | C | T | 0.14754 | 0.00570 | 7.93E-148 | -0.0007 | 0.0021 | 7.62E-01 |
| AD | Superior frontal | rs12590654 | A | G | -0.01483 | 0.00231 | 1.32E-10 | -0.0007 | 0.0007 | 3.14E-01 |
| AD | Superior frontal | rs1859788 | A | G | -0.01840 | 0.00231 | 1.80E-15 | 0.0003 | 0.0008 | 6.62E-01 |
| AD | Superior frontal | rs204473 | A | G | -0.04167 | 0.00700 | 2.58E-09 | 0.0034 | 0.0022 | 1.28E-01 |
| AD | Superior frontal | rs2081545 | A | C | -0.01787 | 0.00223 | 1.11E-15 | 0.0000 | 0.0007 | 9.80E-01 |
| AD | Superior frontal | rs28394864 | A | G | 0.01230 | 0.00218 | 1.68E-08 | 0.0002 | 0.0006 | 7.71E-01 |
| AD | Superior frontal | rs28399657 | G | A | -0.05464 | 0.00658 | 9.82E-17 | 0.0034 | 0.0020 | 8.96E-02 |
| AD | Superior frontal | rs41289512 | G | C | 0.20630 | 0.00578 | 1.00E-200 | -0.0026 | 0.0021 | 2.26E-01 |
| AD | Superior frontal | rs41290120 | A | G | -0.09905 | 0.00578 | 7.14E-66 | -0.0028 | 0.0017 | 1.08E-01 |
| AD | Superior frontal | rs4236673 | A | G | -0.02016 | 0.00223 | 1.48E-19 | -0.0005 | 0.0007 | 4.95E-01 |
| AD | Superior frontal | rs442495 | C | T | -0.01372 | 0.00226 | 1.22E-09 | -0.0008 | 0.0007 | 2.84E-01 |
| AD | Superior frontal | rs4575098 | A | G | 0.01641 | 0.00258 | 1.90E-10 | 0.0003 | 0.0008 | 7.04E-01 |
| AD | Superior frontal | rs4663105 | C | A | 0.03110 | 0.00222 | 1.45E-44 | 0.0004 | 0.0007 | 5.98E-01 |
| AD | Superior frontal | rs59735493 | A | G | -0.01299 | 0.00236 | 3.73E-08 | -0.0005 | 0.0007 | 5.24E-01 |
| AD | Superior frontal | rs6014724 | G | A | -0.02289 | 0.00369 | 5.38E-10 | 0.0002 | 0.0012 | 9.02E-01 |
| AD | Superior frontal | rs6448453 | A | G | 0.01470 | 0.00245 | 1.98E-09 | -0.0003 | 0.0007 | 6.63E-01 |
| AD | Superior frontal | rs679515 | T | C | 0.02542 | 0.00286 | 6.83E-19 | -0.0007 | 0.0009 | 4.09E-01 |
| AD | Superior frontal | rs755951 | C | A | 0.01500 | 0.00221 | 1.13E-11 | -0.0004 | 0.0007 | 5.28E-01 |
| AD | Superior frontal | rs7810606 | T | C | -0.01452 | 0.00218 | 2.89E-11 | 0.0005 | 0.0007 | 5.02E-01 |
| AD | Superior frontal | rs846881 | C | A | -0.01737 | 0.00269 | 9.89E-11 | 0.0005 | 0.0009 | 5.90E-01 |
| AD | Superior frontal | rs867611 | G | A | -0.02043 | 0.00232 | 1.48E-18 | -0.0001 | 0.0007 | 8.95E-01 |
| AD | Superior frontal | rs9381563 | C | T | 0.01445 | 0.00227 | 1.99E-10 | -0.0006 | 0.0007 | 3.66E-01 |
| AD | banks of the superior temporal sulcus | rs10933431 | G | C | -0.01544 | 0.00251 | 7.62E-10 | 0.0010 | 0.0012 | 4.03E-01 |
| AD | banks of the superior temporal sulcus | rs111278892 | G | C | 0.01991 | 0.00305 | 6.67E-11 | -0.0008 | 0.0015 | 5.80E-01 |
| AD | banks of the superior temporal sulcus | rs11218343 | C | T | -0.03593 | 0.00526 | 8.12E-12 | -0.0011 | 0.0024 | 6.36E-01 |
| AD | banks of the superior temporal sulcus | rs11257238 | C | T | 0.01294 | 0.00226 | 1.04E-08 | 0.0014 | 0.0010 | 1.78E-01 |
| AD | banks of the superior temporal sulcus | rs113260531 | A | G | 0.01999 | 0.00325 | 7.91E-10 | 0.0023 | 0.0014 | 1.06E-01 |
| AD | banks of the superior temporal sulcus | rs118170342 | C | T | 0.14754 | 0.00570 | 7.93E-148 | 0.0016 | 0.0031 | 6.07E-01 |
| AD | banks of the superior temporal sulcus | rs12590654 | A | G | -0.01483 | 0.00231 | 1.32E-10 | 0.0012 | 0.0010 | 2.48E-01 |
| AD | banks of the superior temporal sulcus | rs1859788 | A | G | -0.01840 | 0.00231 | 1.80E-15 | -0.0016 | 0.0011 | 1.39E-01 |
| AD | banks of the superior temporal sulcus | rs204473 | A | G | -0.04167 | 0.00700 | 2.58E-09 | 0.0001 | 0.0032 | 9.77E-01 |
| AD | banks of the superior temporal sulcus | rs2081545 | A | C | -0.01787 | 0.00223 | 1.11E-15 | 0.0005 | 0.0009 | 5.93E-01 |
| AD | banks of the superior temporal sulcus | rs28394864 | A | G | 0.01230 | 0.00218 | 1.68E-08 | -0.0016 | 0.0009 | 9.95E-02 |
| AD | banks of the superior temporal sulcus | rs28399657 | G | A | -0.05464 | 0.00658 | 9.82E-17 | 0.0036 | 0.0028 | 1.94E-01 |
| AD | banks of the superior temporal sulcus | rs41289512 | G | C | 0.20630 | 0.00578 | 1.00E-200 | 0.0009 | 0.0029 | 7.72E-01 |
| AD | banks of the superior temporal sulcus | rs41290120 | A | G | -0.09905 | 0.00578 | 7.14E-66 | 0.0019 | 0.0024 | 4.27E-01 |
| AD | banks of the superior temporal sulcus | rs4236673 | A | G | -0.02016 | 0.00223 | 1.48E-19 | 0.0007 | 0.0009 | 4.35E-01 |
| AD | banks of the superior temporal sulcus | rs442495 | C | T | -0.01372 | 0.00226 | 1.22E-09 | 0.0014 | 0.0010 | 1.64E-01 |
| AD | banks of the superior temporal sulcus | rs4575098 | A | G | 0.01641 | 0.00258 | 1.90E-10 | 0.0002 | 0.0011 | 8.39E-01 |
| AD | banks of the superior temporal sulcus | rs4663105 | C | A | 0.03110 | 0.00222 | 1.45E-44 | 0.0002 | 0.0010 | 8.66E-01 |
| AD | banks of the superior temporal sulcus | rs59735493 | A | G | -0.01299 | 0.00236 | 3.73E-08 | 0.0008 | 0.0010 | 4.37E-01 |
| AD | banks of the superior temporal sulcus | rs6014724 | G | A | -0.02289 | 0.00369 | 5.38E-10 | -0.0019 | 0.0017 | 2.61E-01 |
| AD | banks of the superior temporal sulcus | rs6448453 | A | G | 0.01470 | 0.00245 | 1.98E-09 | 0.0010 | 0.0010 | 3.45E-01 |
| AD | banks of the superior temporal sulcus | rs679515 | T | C | 0.02542 | 0.00286 | 6.83E-19 | 0.0010 | 0.0012 | 4.31E-01 |
| AD | banks of the superior temporal sulcus | rs755951 | C | A | 0.01500 | 0.00221 | 1.13E-11 | 0.0007 | 0.0010 | 4.80E-01 |
| AD | banks of the superior temporal sulcus | rs7810606 | T | C | -0.01452 | 0.00218 | 2.89E-11 | -0.0002 | 0.0009 | 8.02E-01 |
| AD | banks of the superior temporal sulcus | rs846881 | C | A | -0.01737 | 0.00269 | 9.89E-11 | 0.0011 | 0.0013 | 4.12E-01 |
| AD | banks of the superior temporal sulcus | rs867611 | G | A | -0.02043 | 0.00232 | 1.48E-18 | 0.0006 | 0.0010 | 5.43E-01 |
| AD | banks of the superior temporal sulcus | rs9381563 | C | T | 0.01445 | 0.00227 | 1.99E-10 | 0.0000 | 0.0010 | 9.80E-01 |
| AD | entorhinal | rs10933431 | G | C | -0.01544 | 0.00251 | 7.62E-10 | 0.0037 | 0.0031 | 2.34E-01 |
| AD | entorhinal | rs111278892 | G | C | 0.01991 | 0.00305 | 6.67E-11 | -0.0008 | 0.0037 | 8.32E-01 |
| AD | entorhinal | rs11218343 | C | T | -0.03593 | 0.00526 | 8.12E-12 | -0.0052 | 0.0061 | 3.99E-01 |
| AD | entorhinal | rs11257238 | C | T | 0.01294 | 0.00226 | 1.04E-08 | -0.0053 | 0.0026 | 3.97E-02 |
| AD | entorhinal | rs113260531 | A | G | 0.01999 | 0.00325 | 7.91E-10 | 0.0021 | 0.0037 | 5.60E-01 |
| AD | entorhinal | rs118170342 | C | T | 0.14754 | 0.00570 | 7.93E-148 | 0.0105 | 0.0076 | 1.65E-01 |
| AD | entorhinal | rs12590654 | A | G | -0.01483 | 0.00231 | 1.32E-10 | 0.0017 | 0.0026 | 5.13E-01 |
| AD | entorhinal | rs1859788 | A | G | -0.01840 | 0.00231 | 1.80E-15 | -0.0004 | 0.0027 | 8.85E-01 |
| AD | entorhinal | rs204473 | A | G | -0.04167 | 0.00700 | 2.58E-09 | -0.0106 | 0.0080 | 1.88E-01 |
| AD | entorhinal | rs2081545 | A | C | -0.01787 | 0.00223 | 1.11E-15 | 0.0040 | 0.0024 | 1.01E-01 |
| AD | entorhinal | rs28394864 | A | G | 0.01230 | 0.00218 | 1.68E-08 | -0.0035 | 0.0024 | 1.47E-01 |
| AD | entorhinal | rs28399657 | G | A | -0.05464 | 0.00658 | 9.82E-17 | -0.0004 | 0.0070 | 9.51E-01 |
| AD | entorhinal | rs41289512 | G | C | 0.20630 | 0.00578 | 1.00E-200 | -0.0024 | 0.0073 | 7.42E-01 |
| AD | entorhinal | rs41290120 | A | G | -0.09905 | 0.00578 | 7.14E-66 | -0.0018 | 0.0061 | 7.62E-01 |
| AD | entorhinal | rs4236673 | A | G | -0.02016 | 0.00223 | 1.48E-19 | -0.0017 | 0.0024 | 4.92E-01 |
| AD | entorhinal | rs442495 | C | T | -0.01372 | 0.00226 | 1.22E-09 | 0.0064 | 0.0026 | 1.26E-02 |
| AD | entorhinal | rs4575098 | A | G | 0.01641 | 0.00258 | 1.90E-10 | 0.0042 | 0.0028 | 1.37E-01 |
| AD | entorhinal | rs4663105 | C | A | 0.03110 | 0.00222 | 1.45E-44 | -0.0029 | 0.0025 | 2.47E-01 |
| AD | entorhinal | rs59735493 | A | G | -0.01299 | 0.00236 | 3.73E-08 | -0.0019 | 0.0026 | 4.73E-01 |
| AD | entorhinal | rs6014724 | G | A | -0.02289 | 0.00369 | 5.38E-10 | 0.0004 | 0.0043 | 9.34E-01 |
| AD | entorhinal | rs6448453 | A | G | 0.01470 | 0.00245 | 1.98E-09 | 0.0002 | 0.0027 | 9.50E-01 |
| AD | entorhinal | rs679515 | T | C | 0.02542 | 0.00286 | 6.83E-19 | 0.0047 | 0.0031 | 1.32E-01 |
| AD | entorhinal | rs755951 | C | A | 0.01500 | 0.00221 | 1.13E-11 | -0.0009 | 0.0024 | 7.27E-01 |
| AD | entorhinal | rs7810606 | T | C | -0.01452 | 0.00218 | 2.89E-11 | 0.0001 | 0.0024 | 9.76E-01 |
| AD | entorhinal | rs846881 | C | A | -0.01737 | 0.00269 | 9.89E-11 | 0.0029 | 0.0033 | 3.71E-01 |
| AD | entorhinal | rs867611 | G | A | -0.02043 | 0.00232 | 1.48E-18 | -0.0059 | 0.0025 | 2.09E-02 |
| AD | entorhinal | rs9381563 | C | T | 0.01445 | 0.00227 | 1.99E-10 | 0.0053 | 0.0025 | 3.42E-02 |
| AD | fusiform | rs10933431 | G | C | -0.01544 | 0.00251 | 7.62E-10 | -0.0009 | 0.0011 | 3.92E-01 |
| AD | fusiform | rs111278892 | G | C | 0.01991 | 0.00305 | 6.67E-11 | 0.0017 | 0.0012 | 1.62E-01 |
| AD | fusiform | rs11218343 | C | T | -0.03593 | 0.00526 | 8.12E-12 | 0.0025 | 0.0020 | 2.11E-01 |
| AD | fusiform | rs11257238 | C | T | 0.01294 | 0.00226 | 1.04E-08 | -0.0019 | 0.0009 | 2.95E-02 |
| AD | fusiform | rs113260531 | A | G | 0.01999 | 0.00325 | 7.91E-10 | 0.0034 | 0.0012 | 5.23E-03 |
| AD | fusiform | rs118170342 | C | T | 0.14754 | 0.00570 | 7.93E-148 | 0.0018 | 0.0025 | 4.71E-01 |
| AD | fusiform | rs12590654 | A | G | -0.01483 | 0.00231 | 1.32E-10 | -0.0002 | 0.0009 | 8.56E-01 |
| AD | fusiform | rs1859788 | A | G | -0.01840 | 0.00231 | 1.80E-15 | -0.0011 | 0.0009 | 2.14E-01 |
| AD | fusiform | rs204473 | A | G | -0.04167 | 0.00700 | 2.58E-09 | 0.0011 | 0.0026 | 6.78E-01 |
| AD | fusiform | rs2081545 | A | C | -0.01787 | 0.00223 | 1.11E-15 | 0.0002 | 0.0008 | 7.59E-01 |
| AD | fusiform | rs28394864 | A | G | 0.01230 | 0.00218 | 1.68E-08 | -0.0005 | 0.0008 | 5.44E-01 |
| AD | fusiform | rs28399657 | G | A | -0.05464 | 0.00658 | 9.82E-17 | 0.0006 | 0.0023 | 7.80E-01 |
| AD | fusiform | rs41289512 | G | C | 0.20630 | 0.00578 | 1.00E-200 | -0.0027 | 0.0025 | 2.76E-01 |
| AD | fusiform | rs41290120 | A | G | -0.09905 | 0.00578 | 7.14E-66 | 0.0009 | 0.0020 | 6.57E-01 |
| AD | fusiform | rs4236673 | A | G | -0.02016 | 0.00223 | 1.48E-19 | 0.0001 | 0.0008 | 8.74E-01 |
| AD | fusiform | rs442495 | C | T | -0.01372 | 0.00226 | 1.22E-09 | -0.0009 | 0.0009 | 2.95E-01 |
| AD | fusiform | rs4575098 | A | G | 0.01641 | 0.00258 | 1.90E-10 | 0.0003 | 0.0009 | 7.52E-01 |
| AD | fusiform | rs4663105 | C | A | 0.03110 | 0.00222 | 1.45E-44 | -0.0003 | 0.0009 | 7.18E-01 |
| AD | fusiform | rs59735493 | A | G | -0.01299 | 0.00236 | 3.73E-08 | -0.0001 | 0.0009 | 9.31E-01 |
| AD | fusiform | rs6014724 | G | A | -0.02289 | 0.00369 | 5.38E-10 | -0.0011 | 0.0014 | 4.13E-01 |
| AD | fusiform | rs6448453 | A | G | 0.01470 | 0.00245 | 1.98E-09 | -0.0006 | 0.0009 | 4.97E-01 |
| AD | fusiform | rs679515 | T | C | 0.02542 | 0.00286 | 6.83E-19 | 0.0023 | 0.0010 | 3.16E-02 |
| AD | fusiform | rs755951 | C | A | 0.01500 | 0.00221 | 1.13E-11 | -0.0011 | 0.0008 | 1.76E-01 |
| AD | fusiform | rs7810606 | T | C | -0.01452 | 0.00218 | 2.89E-11 | 0.0002 | 0.0008 | 7.81E-01 |
| AD | fusiform | rs846881 | C | A | -0.01737 | 0.00269 | 9.89E-11 | -0.0006 | 0.0011 | 6.06E-01 |
| AD | fusiform | rs867611 | G | A | -0.02043 | 0.00232 | 1.48E-18 | 0.0008 | 0.0009 | 3.68E-01 |
| AD | fusiform | rs9381563 | C | T | 0.01445 | 0.00227 | 1.99E-10 | 0.0005 | 0.0008 | 5.54E-01 |
| AD | Inferior temporal | rs10933431 | G | C | -0.01544 | 0.00251 | 7.62E-10 | 0.0007 | 0.0012 | 5.57E-01 |
| AD | Inferior temporal | rs111278892 | G | C | 0.01991 | 0.00305 | 6.67E-11 | 0.0009 | 0.0013 | 4.87E-01 |
| AD | Inferior temporal | rs11218343 | C | T | -0.03593 | 0.00526 | 8.12E-12 | 0.0045 | 0.0023 | 5.08E-02 |
| AD | Inferior temporal | rs11257238 | C | T | 0.01294 | 0.00226 | 1.04E-08 | -0.0026 | 0.0010 | 6.53E-03 |
| AD | Inferior temporal | rs113260531 | A | G | 0.01999 | 0.00325 | 7.91E-10 | 0.0005 | 0.0013 | 7.07E-01 |
| AD | Inferior temporal | rs118170342 | C | T | 0.14754 | 0.00570 | 7.93E-148 | 0.0025 | 0.0028 | 3.80E-01 |
| AD | Inferior temporal | rs12590654 | A | G | -0.01483 | 0.00231 | 1.32E-10 | 0.0002 | 0.0010 | 8.70E-01 |
| AD | Inferior temporal | rs1859788 | A | G | -0.01840 | 0.00231 | 1.80E-15 | -0.0031 | 0.0010 | 1.35E-03 |
| AD | Inferior temporal | rs204473 | A | G | -0.04167 | 0.00700 | 2.58E-09 | 0.0017 | 0.0029 | 5.53E-01 |
| AD | Inferior temporal | rs2081545 | A | C | -0.01787 | 0.00223 | 1.11E-15 | -0.0002 | 0.0009 | 8.26E-01 |
| AD | Inferior temporal | rs28394864 | A | G | 0.01230 | 0.00218 | 1.68E-08 | 0.0000 | 0.0009 | 9.61E-01 |
| AD | Inferior temporal | rs28399657 | G | A | -0.05464 | 0.00658 | 9.82E-17 | -0.0039 | 0.0026 | 1.34E-01 |
| AD | Inferior temporal | rs41289512 | G | C | 0.20630 | 0.00578 | 1.00E-200 | 0.0004 | 0.0027 | 8.83E-01 |
| AD | Inferior temporal | rs41290120 | A | G | -0.09905 | 0.00578 | 7.14E-66 | 0.0027 | 0.0022 | 2.26E-01 |
| AD | Inferior temporal | rs4236673 | A | G | -0.02016 | 0.00223 | 1.48E-19 | -0.0009 | 0.0009 | 3.15E-01 |
| AD | Inferior temporal | rs442495 | C | T | -0.01372 | 0.00226 | 1.22E-09 | -0.0021 | 0.0010 | 2.74E-02 |
| AD | Inferior temporal | rs4575098 | A | G | 0.01641 | 0.00258 | 1.90E-10 | 0.0023 | 0.0011 | 3.03E-02 |
| AD | Inferior temporal | rs4663105 | C | A | 0.03110 | 0.00222 | 1.45E-44 | -0.0015 | 0.0009 | 1.14E-01 |
| AD | Inferior temporal | rs59735493 | A | G | -0.01299 | 0.00236 | 3.73E-08 | -0.0003 | 0.0010 | 7.94E-01 |
| AD | Inferior temporal | rs6014724 | G | A | -0.02289 | 0.00369 | 5.38E-10 | 0.0013 | 0.0016 | 4.20E-01 |
| AD | Inferior temporal | rs6448453 | A | G | 0.01470 | 0.00245 | 1.98E-09 | 0.0023 | 0.0010 | 1.95E-02 |
| AD | Inferior temporal | rs679515 | T | C | 0.02542 | 0.00286 | 6.83E-19 | 0.0000 | 0.0011 | 9.98E-01 |
| AD | Inferior temporal | rs755951 | C | A | 0.01500 | 0.00221 | 1.13E-11 | -0.0001 | 0.0009 | 8.71E-01 |
| AD | Inferior temporal | rs7810606 | T | C | -0.01452 | 0.00218 | 2.89E-11 | -0.0006 | 0.0009 | 4.96E-01 |
| AD | Inferior temporal | rs846881 | C | A | -0.01737 | 0.00269 | 9.89E-11 | 0.0014 | 0.0012 | 2.72E-01 |
| AD | Inferior temporal | rs867611 | G | A | -0.02043 | 0.00232 | 1.48E-18 | 0.0022 | 0.0010 | 2.00E-02 |
| AD | Inferior temporal | rs9381563 | C | T | 0.01445 | 0.00227 | 1.99E-10 | 0.0002 | 0.0010 | 8.22E-01 |
| AD | insula | rs10933431 | G | C | -0.01544 | 0.00251 | 7.62E-10 | -0.0003 | 0.0012 | 7.81E-01 |
| AD | insula | rs111278892 | G | C | 0.01991 | 0.00305 | 6.67E-11 | 0.0004 | 0.0014 | 7.59E-01 |
| AD | insula | rs11218343 | C | T | -0.03593 | 0.00526 | 8.12E-12 | 0.0006 | 0.0024 | 7.88E-01 |
| AD | insula | rs11257238 | C | T | 0.01294 | 0.00226 | 1.04E-08 | 0.0000 | 0.0010 | 9.99E-01 |
| AD | insula | rs113260531 | A | G | 0.01999 | 0.00325 | 7.91E-10 | 0.0015 | 0.0014 | 2.91E-01 |
| AD | insula | rs118170342 | C | T | 0.14754 | 0.00570 | 7.93E-148 | 0.0026 | 0.0030 | 3.77E-01 |
| AD | insula | rs12590654 | A | G | -0.01483 | 0.00231 | 1.32E-10 | 0.0002 | 0.0010 | 8.04E-01 |
| AD | insula | rs1859788 | A | G | -0.01840 | 0.00231 | 1.80E-15 | -0.0020 | 0.0011 | 6.42E-02 |
| AD | insula | rs204473 | A | G | -0.04167 | 0.00700 | 2.58E-09 | -0.0002 | 0.0031 | 9.45E-01 |
| AD | insula | rs2081545 | A | C | -0.01787 | 0.00223 | 1.11E-15 | 0.0015 | 0.0009 | 1.11E-01 |
| AD | insula | rs28394864 | A | G | 0.01230 | 0.00218 | 1.68E-08 | 0.0002 | 0.0009 | 7.92E-01 |
| AD | insula | rs28399657 | G | A | -0.05464 | 0.00658 | 9.82E-17 | -0.0017 | 0.0027 | 5.38E-01 |
| AD | insula | rs41289512 | G | C | 0.20630 | 0.00578 | 1.00E-200 | -0.0032 | 0.0029 | 2.65E-01 |
| AD | insula | rs41290120 | A | G | -0.09905 | 0.00578 | 7.14E-66 | 0.0015 | 0.0024 | 5.36E-01 |
| AD | insula | rs4236673 | A | G | -0.02016 | 0.00223 | 1.48E-19 | -0.0014 | 0.0009 | 1.41E-01 |
| AD | insula | rs442495 | C | T | -0.01372 | 0.00226 | 1.22E-09 | 0.0008 | 0.0010 | 4.28E-01 |
| AD | insula | rs4575098 | A | G | 0.01641 | 0.00258 | 1.90E-10 | -0.0010 | 0.0011 | 3.56E-01 |
| AD | insula | rs4663105 | C | A | 0.03110 | 0.00222 | 1.45E-44 | -0.0002 | 0.0010 | 8.24E-01 |
| AD | insula | rs59735493 | A | G | -0.01299 | 0.00236 | 3.73E-08 | -0.0017 | 0.0010 | 9.45E-02 |
| AD | insula | rs6014724 | G | A | -0.02289 | 0.00369 | 5.38E-10 | -0.0003 | 0.0017 | 8.65E-01 |
| AD | insula | rs6448453 | A | G | 0.01470 | 0.00245 | 1.98E-09 | -0.0009 | 0.0010 | 3.83E-01 |
| AD | insula | rs679515 | T | C | 0.02542 | 0.00286 | 6.83E-19 | 0.0000 | 0.0012 | 9.83E-01 |
| AD | insula | rs755951 | C | A | 0.01500 | 0.00221 | 1.13E-11 | -0.0005 | 0.0009 | 5.83E-01 |
| AD | insula | rs7810606 | T | C | -0.01452 | 0.00218 | 2.89E-11 | 0.0001 | 0.0009 | 9.52E-01 |
| AD | insula | rs846881 | C | A | -0.01737 | 0.00269 | 9.89E-11 | 0.0025 | 0.0013 | 4.72E-02 |
| AD | insula | rs867611 | G | A | -0.02043 | 0.00232 | 1.48E-18 | 0.0019 | 0.0010 | 5.81E-02 |
| AD | insula | rs9381563 | C | T | 0.01445 | 0.00227 | 1.99E-10 | 0.0001 | 0.0010 | 9.44E-01 |
| AD | Middle temporal | rs10933431 | G | C | -0.01544 | 0.00251 | 7.62E-10 | 0.0004 | 0.0011 | 7.00E-01 |
| AD | Middle temporal | rs111278892 | G | C | 0.01991 | 0.00305 | 6.67E-11 | 0.0004 | 0.0013 | 7.66E-01 |
| AD | Middle temporal | rs11218343 | C | T | -0.03593 | 0.00526 | 8.12E-12 | 0.0034 | 0.0021 | 1.01E-01 |
| AD | Middle temporal | rs11257238 | C | T | 0.01294 | 0.00226 | 1.04E-08 | -0.0017 | 0.0009 | 5.53E-02 |
| AD | Middle temporal | rs113260531 | A | G | 0.01999 | 0.00325 | 7.91E-10 | 0.0024 | 0.0012 | 5.28E-02 |
| AD | Middle temporal | rs118170342 | C | T | 0.14754 | 0.00570 | 7.93E-148 | 0.0038 | 0.0026 | 1.43E-01 |
| AD | Middle temporal | rs12590654 | A | G | -0.01483 | 0.00231 | 1.32E-10 | 0.0007 | 0.0009 | 4.48E-01 |
| AD | Middle temporal | rs1859788 | A | G | -0.01840 | 0.00231 | 1.80E-15 | -0.0006 | 0.0009 | 5.42E-01 |
| AD | Middle temporal | rs204473 | A | G | -0.04167 | 0.00700 | 2.58E-09 | 0.0003 | 0.0027 | 9.09E-01 |
| AD | Middle temporal | rs2081545 | A | C | -0.01787 | 0.00223 | 1.11E-15 | -0.0002 | 0.0009 | 8.05E-01 |
| AD | Middle temporal | rs28394864 | A | G | 0.01230 | 0.00218 | 1.68E-08 | -0.0002 | 0.0008 | 8.01E-01 |
| AD | Middle temporal | rs28399657 | G | A | -0.05464 | 0.00658 | 9.82E-17 | -0.0005 | 0.0024 | 8.29E-01 |
| AD | Middle temporal | rs41289512 | G | C | 0.20630 | 0.00578 | 1.00E-200 | 0.0012 | 0.0025 | 6.27E-01 |
| AD | Middle temporal | rs41290120 | A | G | -0.09905 | 0.00578 | 7.14E-66 | 0.0011 | 0.0021 | 6.15E-01 |
| AD | Middle temporal | rs4236673 | A | G | -0.02016 | 0.00223 | 1.48E-19 | 0.0007 | 0.0009 | 3.88E-01 |
| AD | Middle temporal | rs442495 | C | T | -0.01372 | 0.00226 | 1.22E-09 | 0.0007 | 0.0009 | 4.49E-01 |
| AD | Middle temporal | rs4575098 | A | G | 0.01641 | 0.00258 | 1.90E-10 | 0.0008 | 0.0010 | 4.05E-01 |
| AD | Middle temporal | rs4663105 | C | A | 0.03110 | 0.00222 | 1.45E-44 | -0.0004 | 0.0009 | 6.34E-01 |
| AD | Middle temporal | rs59735493 | A | G | -0.01299 | 0.00236 | 3.73E-08 | -0.0001 | 0.0009 | 9.11E-01 |
| AD | Middle temporal | rs6014724 | G | A | -0.02289 | 0.00369 | 5.38E-10 | 0.0011 | 0.0015 | 4.70E-01 |
| AD | Middle temporal | rs6448453 | A | G | 0.01470 | 0.00245 | 1.98E-09 | 0.0006 | 0.0010 | 5.34E-01 |
| AD | Middle temporal | rs679515 | T | C | 0.02542 | 0.00286 | 6.83E-19 | 0.0016 | 0.0011 | 1.39E-01 |
| AD | Middle temporal | rs755951 | C | A | 0.01500 | 0.00221 | 1.13E-11 | -0.0005 | 0.0009 | 5.75E-01 |
| AD | Middle temporal | rs7810606 | T | C | -0.01452 | 0.00218 | 2.89E-11 | 0.0003 | 0.0009 | 7.69E-01 |
| AD | Middle temporal | rs846881 | C | A | -0.01737 | 0.00269 | 9.89E-11 | 0.0002 | 0.0011 | 8.50E-01 |
| AD | Middle temporal | rs867611 | G | A | -0.02043 | 0.00232 | 1.48E-18 | 0.0009 | 0.0009 | 3.19E-01 |
| AD | Middle temporal | rs9381563 | C | T | 0.01445 | 0.00227 | 1.99E-10 | 0.0015 | 0.0009 | 7.89E-02 |
| AD | parahippocampal | rs10933431 | G | C | -0.01544 | 0.00251 | 7.62E-10 | -0.0015 | 0.0028 | 5.90E-01 |
| AD | parahippocampal | rs111278892 | G | C | 0.01991 | 0.00305 | 6.67E-11 | -0.0006 | 0.0032 | 8.56E-01 |
| AD | parahippocampal | rs11218343 | C | T | -0.03593 | 0.00526 | 8.12E-12 | 0.0046 | 0.0053 | 3.88E-01 |
| AD | parahippocampal | rs11257238 | C | T | 0.01294 | 0.00226 | 1.04E-08 | -0.0038 | 0.0022 | 8.98E-02 |
| AD | parahippocampal | rs113260531 | A | G | 0.01999 | 0.00325 | 7.91E-10 | 0.0005 | 0.0031 | 8.85E-01 |
| AD | parahippocampal | rs118170342 | C | T | 0.14754 | 0.00570 | 7.93E-148 | -0.0122 | 0.0068 | 7.17E-02 |
| AD | parahippocampal | rs12590654 | A | G | -0.01483 | 0.00231 | 1.32E-10 | 0.0003 | 0.0023 | 9.06E-01 |
| AD | parahippocampal | rs1859788 | A | G | -0.01840 | 0.00231 | 1.80E-15 | 0.0028 | 0.0023 | 2.24E-01 |
| AD | parahippocampal | rs204473 | A | G | -0.04167 | 0.00700 | 2.58E-09 | -0.0126 | 0.0070 | 6.93E-02 |
| AD | parahippocampal | rs2081545 | A | C | -0.01787 | 0.00223 | 1.11E-15 | 0.0004 | 0.0021 | 8.51E-01 |
| AD | parahippocampal | rs28394864 | A | G | 0.01230 | 0.00218 | 1.68E-08 | -0.0056 | 0.0021 | 7.36E-03 |
| AD | parahippocampal | rs28399657 | G | A | -0.05464 | 0.00658 | 9.82E-17 | 0.0013 | 0.0062 | 8.29E-01 |
| AD | parahippocampal | rs41289512 | G | C | 0.20630 | 0.00578 | 1.00E-200 | -0.0033 | 0.0065 | 6.15E-01 |
| AD | parahippocampal | rs41290120 | A | G | -0.09905 | 0.00578 | 7.14E-66 | 0.0055 | 0.0052 | 2.94E-01 |
| AD | parahippocampal | rs4236673 | A | G | -0.02016 | 0.00223 | 1.48E-19 | -0.0003 | 0.0021 | 8.92E-01 |
| AD | parahippocampal | rs442495 | C | T | -0.01372 | 0.00226 | 1.22E-09 | 0.0022 | 0.0022 | 3.14E-01 |
| AD | parahippocampal | rs4575098 | A | G | 0.01641 | 0.00258 | 1.90E-10 | 0.0046 | 0.0025 | 6.04E-02 |
| AD | parahippocampal | rs4663105 | C | A | 0.03110 | 0.00222 | 1.45E-44 | -0.0044 | 0.0022 | 4.18E-02 |
| AD | parahippocampal | rs59735493 | A | G | -0.01299 | 0.00236 | 3.73E-08 | 0.0047 | 0.0023 | 4.00E-02 |
| AD | parahippocampal | rs6014724 | G | A | -0.02289 | 0.00369 | 5.38E-10 | 0.0023 | 0.0037 | 5.30E-01 |
| AD | parahippocampal | rs6448453 | A | G | 0.01470 | 0.00245 | 1.98E-09 | 0.0013 | 0.0023 | 5.74E-01 |
| AD | parahippocampal | rs679515 | T | C | 0.02542 | 0.00286 | 6.83E-19 | -0.0002 | 0.0027 | 9.44E-01 |
| AD | parahippocampal | rs755951 | C | A | 0.01500 | 0.00221 | 1.13E-11 | 0.0030 | 0.0021 | 1.49E-01 |
| AD | parahippocampal | rs7810606 | T | C | -0.01452 | 0.00218 | 2.89E-11 | -0.0043 | 0.0021 | 3.89E-02 |
| AD | parahippocampal | rs846881 | C | A | -0.01737 | 0.00269 | 9.89E-11 | -0.0005 | 0.0029 | 8.56E-01 |
| AD | parahippocampal | rs867611 | G | A | -0.02043 | 0.00232 | 1.48E-18 | -0.0005 | 0.0022 | 8.18E-01 |
| AD | parahippocampal | rs9381563 | C | T | 0.01445 | 0.00227 | 1.99E-10 | 0.0036 | 0.0022 | 9.79E-02 |
| AD | Superior temporal | rs10933431 | G | C | -0.01544 | 0.00251 | 7.62E-10 | -0.0008 | 0.0011 | 4.84E-01 |
| AD | Superior temporal | rs111278892 | G | C | 0.01991 | 0.00305 | 6.67E-11 | 0.0011 | 0.0013 | 3.88E-01 |
| AD | Superior temporal | rs11218343 | C | T | -0.03593 | 0.00526 | 8.12E-12 | -0.0006 | 0.0021 | 7.66E-01 |
| AD | Superior temporal | rs11257238 | C | T | 0.01294 | 0.00226 | 1.04E-08 | 0.0008 | 0.0009 | 3.58E-01 |
| AD | Superior temporal | rs113260531 | A | G | 0.01999 | 0.00325 | 7.91E-10 | -0.0004 | 0.0012 | 7.51E-01 |
| AD | Superior temporal | rs118170342 | C | T | 0.14754 | 0.00570 | 7.93E-148 | -0.0031 | 0.0026 | 2.33E-01 |
| AD | Superior temporal | rs12590654 | A | G | -0.01483 | 0.00231 | 1.32E-10 | 0.0001 | 0.0009 | 9.45E-01 |
| AD | Superior temporal | rs1859788 | A | G | -0.01840 | 0.00231 | 1.80E-15 | 0.0011 | 0.0009 | 2.32E-01 |
| AD | Superior temporal | rs204473 | A | G | -0.04167 | 0.00700 | 2.58E-09 | -0.0024 | 0.0027 | 3.87E-01 |
| AD | Superior temporal | rs2081545 | A | C | -0.01787 | 0.00223 | 1.11E-15 | 0.0001 | 0.0009 | 9.47E-01 |
| AD | Superior temporal | rs28394864 | A | G | 0.01230 | 0.00218 | 1.68E-08 | -0.0013 | 0.0008 | 9.15E-02 |
| AD | Superior temporal | rs28399657 | G | A | -0.05464 | 0.00658 | 9.82E-17 | -0.0031 | 0.0024 | 1.97E-01 |
| AD | Superior temporal | rs41289512 | G | C | 0.20630 | 0.00578 | 1.00E-200 | -0.0015 | 0.0025 | 5.51E-01 |
| AD | Superior temporal | rs41290120 | A | G | -0.09905 | 0.00578 | 7.14E-66 | 0.0020 | 0.0021 | 3.39E-01 |
| AD | Superior temporal | rs4236673 | A | G | -0.02016 | 0.00223 | 1.48E-19 | 0.0005 | 0.0009 | 5.70E-01 |
| AD | Superior temporal | rs442495 | C | T | -0.01372 | 0.00226 | 1.22E-09 | 0.0020 | 0.0009 | 2.50E-02 |
| AD | Superior temporal | rs4575098 | A | G | 0.01641 | 0.00258 | 1.90E-10 | 0.0024 | 0.0010 | 1.21E-02 |
| AD | Superior temporal | rs4663105 | C | A | 0.03110 | 0.00222 | 1.45E-44 | -0.0003 | 0.0009 | 7.01E-01 |
| AD | Superior temporal | rs59735493 | A | G | -0.01299 | 0.00236 | 3.73E-08 | 0.0003 | 0.0009 | 7.64E-01 |
| AD | Superior temporal | rs6014724 | G | A | -0.02289 | 0.00369 | 5.38E-10 | -0.0028 | 0.0015 | 5.35E-02 |
| AD | Superior temporal | rs6448453 | A | G | 0.01470 | 0.00245 | 1.98E-09 | 0.0003 | 0.0009 | 7.53E-01 |
| AD | Superior temporal | rs679515 | T | C | 0.02542 | 0.00286 | 6.83E-19 | 0.0014 | 0.0011 | 1.91E-01 |
| AD | Superior temporal | rs755951 | C | A | 0.01500 | 0.00221 | 1.13E-11 | 0.0003 | 0.0009 | 7.56E-01 |
| AD | Superior temporal | rs7810606 | T | C | -0.01452 | 0.00218 | 2.89E-11 | -0.0009 | 0.0009 | 2.73E-01 |
| AD | Superior temporal | rs846881 | C | A | -0.01737 | 0.00269 | 9.89E-11 | -0.0003 | 0.0012 | 7.94E-01 |
| AD | Superior temporal | rs867611 | G | A | -0.02043 | 0.00232 | 1.48E-18 | 0.0000 | 0.0009 | 9.96E-01 |
| AD | Superior temporal | rs9381563 | C | T | 0.01445 | 0.00227 | 1.99E-10 | -0.0005 | 0.0009 | 5.73E-01 |
| AD | Temporal pole | rs10933431 | G | C | -0.01544 | 0.00251 | 7.62E-10 | 0.0013 | 0.0027 | 6.25E-01 |
| AD | Temporal pole | rs111278892 | G | C | 0.01991 | 0.00305 | 6.67E-11 | 0.0082 | 0.0032 | 1.04E-02 |
| AD | Temporal pole | rs11218343 | C | T | -0.03593 | 0.00526 | 8.12E-12 | 0.0027 | 0.0053 | 6.08E-01 |
| AD | Temporal pole | rs11257238 | C | T | 0.01294 | 0.00226 | 1.04E-08 | -0.0010 | 0.0022 | 6.62E-01 |
| AD | Temporal pole | rs113260531 | A | G | 0.01999 | 0.00325 | 7.91E-10 | 0.0025 | 0.0032 | 4.37E-01 |
| AD | Temporal pole | rs118170342 | C | T | 0.14754 | 0.00570 | 7.93E-148 | 0.0094 | 0.0065 | 1.49E-01 |
| AD | Temporal pole | rs12590654 | A | G | -0.01483 | 0.00231 | 1.32E-10 | 0.0012 | 0.0023 | 6.03E-01 |
| AD | Temporal pole | rs1859788 | A | G | -0.01840 | 0.00231 | 1.80E-15 | -0.0007 | 0.0023 | 7.68E-01 |
| AD | Temporal pole | rs204473 | A | G | -0.04167 | 0.00700 | 2.58E-09 | -0.0046 | 0.0069 | 5.07E-01 |
| AD | Temporal pole | rs2081545 | A | C | -0.01787 | 0.00223 | 1.11E-15 | 0.0039 | 0.0021 | 6.31E-02 |
| AD | Temporal pole | rs28394864 | A | G | 0.01230 | 0.00218 | 1.68E-08 | -0.0020 | 0.0021 | 3.47E-01 |
| AD | Temporal pole | rs28399657 | G | A | -0.05464 | 0.00658 | 9.82E-17 | 0.0043 | 0.0061 | 4.82E-01 |
| AD | Temporal pole | rs41289512 | G | C | 0.20630 | 0.00578 | 1.00E-200 | -0.0041 | 0.0063 | 5.19E-01 |
| AD | Temporal pole | rs41290120 | A | G | -0.09905 | 0.00578 | 7.14E-66 | -0.0029 | 0.0053 | 5.79E-01 |
| AD | Temporal pole | rs4236673 | A | G | -0.02016 | 0.00223 | 1.48E-19 | -0.0016 | 0.0021 | 4.41E-01 |
| AD | Temporal pole | rs442495 | C | T | -0.01372 | 0.00226 | 1.22E-09 | 0.0024 | 0.0022 | 2.89E-01 |
| AD | Temporal pole | rs4575098 | A | G | 0.01641 | 0.00258 | 1.90E-10 | 0.0030 | 0.0025 | 2.23E-01 |
| AD | Temporal pole | rs4663105 | C | A | 0.03110 | 0.00222 | 1.45E-44 | -0.0009 | 0.0022 | 6.72E-01 |
| AD | Temporal pole | rs59735493 | A | G | -0.01299 | 0.00236 | 3.73E-08 | -0.0017 | 0.0023 | 4.61E-01 |
| AD | Temporal pole | rs6014724 | G | A | -0.02289 | 0.00369 | 5.38E-10 | -0.0051 | 0.0037 | 1.75E-01 |
| AD | Temporal pole | rs6448453 | A | G | 0.01470 | 0.00245 | 1.98E-09 | 0.0033 | 0.0024 | 1.64E-01 |
| AD | Temporal pole | rs679515 | T | C | 0.02542 | 0.00286 | 6.83E-19 | 0.0006 | 0.0027 | 8.37E-01 |
| AD | Temporal pole | rs755951 | C | A | 0.01500 | 0.00221 | 1.13E-11 | -0.0008 | 0.0021 | 6.97E-01 |
| AD | Temporal pole | rs7810606 | T | C | -0.01452 | 0.00218 | 2.89E-11 | -0.0035 | 0.0021 | 9.75E-02 |
| AD | Temporal pole | rs846881 | C | A | -0.01737 | 0.00269 | 9.89E-11 | 0.0029 | 0.0029 | 3.13E-01 |
| AD | Temporal pole | rs867611 | G | A | -0.02043 | 0.00232 | 1.48E-18 | -0.0004 | 0.0022 | 8.50E-01 |
| AD | Temporal pole | rs9381563 | C | T | 0.01445 | 0.00227 | 1.99E-10 | -0.0002 | 0.0022 | 9.14E-01 |
| AD | Transverse temporal | rs10933431 | G | C | -0.01544 | 0.00251 | 7.62E-10 | 0.0001 | 0.0017 | 9.64E-01 |
| AD | Transverse temporal | rs111278892 | G | C | 0.01991 | 0.00305 | 6.67E-11 | 0.0023 | 0.0021 | 2.58E-01 |
| AD | Transverse temporal | rs11218343 | C | T | -0.03593 | 0.00526 | 8.12E-12 | -0.0020 | 0.0034 | 5.65E-01 |
| AD | Transverse temporal | rs11257238 | C | T | 0.01294 | 0.00226 | 1.04E-08 | 0.0042 | 0.0014 | 3.56E-03 |
| AD | Transverse temporal | rs113260531 | A | G | 0.01999 | 0.00325 | 7.91E-10 | 0.0054 | 0.0021 | 8.26E-03 |
| AD | Transverse temporal | rs118170342 | C | T | 0.14754 | 0.00570 | 7.93E-148 | 0.0028 | 0.0042 | 5.16E-01 |
| AD | Transverse temporal | rs12590654 | A | G | -0.01483 | 0.00231 | 1.32E-10 | 0.0012 | 0.0015 | 3.94E-01 |
| AD | Transverse temporal | rs1859788 | A | G | -0.01840 | 0.00231 | 1.80E-15 | -0.0003 | 0.0015 | 8.60E-01 |
| AD | Transverse temporal | rs204473 | A | G | -0.04167 | 0.00700 | 2.58E-09 | 0.0029 | 0.0044 | 5.03E-01 |
| AD | Transverse temporal | rs2081545 | A | C | -0.01787 | 0.00223 | 1.11E-15 | -0.0006 | 0.0014 | 6.48E-01 |
| AD | Transverse temporal | rs28394864 | A | G | 0.01230 | 0.00218 | 1.68E-08 | -0.0006 | 0.0013 | 6.50E-01 |
| AD | Transverse temporal | rs28399657 | G | A | -0.05464 | 0.00658 | 9.82E-17 | 0.0023 | 0.0040 | 5.53E-01 |
| AD | Transverse temporal | rs41289512 | G | C | 0.20630 | 0.00578 | 1.00E-200 | 0.0018 | 0.0041 | 6.59E-01 |
| AD | Transverse temporal | rs41290120 | A | G | -0.09905 | 0.00578 | 7.14E-66 | 0.0035 | 0.0034 | 3.01E-01 |
| AD | Transverse temporal | rs4236673 | A | G | -0.02016 | 0.00223 | 1.48E-19 | -0.0008 | 0.0014 | 5.67E-01 |
| AD | Transverse temporal | rs442495 | C | T | -0.01372 | 0.00226 | 1.22E-09 | -0.0010 | 0.0014 | 4.94E-01 |
| AD | Transverse temporal | rs4575098 | A | G | 0.01641 | 0.00258 | 1.90E-10 | 0.0015 | 0.0016 | 3.53E-01 |
| AD | Transverse temporal | rs4663105 | C | A | 0.03110 | 0.00222 | 1.45E-44 | -0.0003 | 0.0014 | 8.36E-01 |
| AD | Transverse temporal | rs59735493 | A | G | -0.01299 | 0.00236 | 3.73E-08 | 0.0021 | 0.0015 | 1.54E-01 |
| AD | Transverse temporal | rs6014724 | G | A | -0.02289 | 0.00369 | 5.38E-10 | -0.0028 | 0.0024 | 2.46E-01 |
| AD | Transverse temporal | rs6448453 | A | G | 0.01470 | 0.00245 | 1.98E-09 | -0.0004 | 0.0015 | 8.05E-01 |
| AD | Transverse temporal | rs679515 | T | C | 0.02542 | 0.00286 | 6.83E-19 | -0.0009 | 0.0017 | 6.14E-01 |
| AD | Transverse temporal | rs755951 | C | A | 0.01500 | 0.00221 | 1.13E-11 | -0.0008 | 0.0014 | 5.61E-01 |
| AD | Transverse temporal | rs7810606 | T | C | -0.01452 | 0.00218 | 2.89E-11 | 0.0007 | 0.0014 | 6.11E-01 |
| AD | Transverse temporal | rs846881 | C | A | -0.01737 | 0.00269 | 9.89E-11 | -0.0010 | 0.0019 | 5.93E-01 |
| AD | Transverse temporal | rs867611 | G | A | -0.02043 | 0.00232 | 1.48E-18 | 0.0009 | 0.0014 | 5.11E-01 |
| AD | Transverse temporal | rs9381563 | C | T | 0.01445 | 0.00227 | 1.99E-10 | -0.0004 | 0.0014 | 7.55E-01 |
| AD | Inferior parietal | rs10933431 | G | C | -0.01544 | 0.00251 | 7.62E-10 | 0.0000 | 0.0007 | 9.56E-01 |
| AD | Inferior parietal | rs111278892 | G | C | 0.01991 | 0.00305 | 6.67E-11 | -0.0016 | 0.0009 | 7.17E-02 |
| AD | Inferior parietal | rs11218343 | C | T | -0.03593 | 0.00526 | 8.12E-12 | 0.0009 | 0.0015 | 5.22E-01 |
| AD | Inferior parietal | rs11257238 | C | T | 0.01294 | 0.00226 | 1.04E-08 | -0.0008 | 0.0006 | 2.29E-01 |
| AD | Inferior parietal | rs113260531 | A | G | 0.01999 | 0.00325 | 7.91E-10 | -0.0013 | 0.0009 | 1.54E-01 |
| AD | Inferior parietal | rs118170342 | C | T | 0.14754 | 0.00570 | 7.93E-148 | 0.0022 | 0.0018 | 2.27E-01 |
| AD | Inferior parietal | rs12590654 | A | G | -0.01483 | 0.00231 | 1.32E-10 | 0.0007 | 0.0006 | 2.14E-01 |
| AD | Inferior parietal | rs1859788 | A | G | -0.01840 | 0.00231 | 1.80E-15 | 0.0012 | 0.0007 | 7.26E-02 |
| AD | Inferior parietal | rs204473 | A | G | -0.04167 | 0.00700 | 2.58E-09 | 0.0022 | 0.0019 | 2.51E-01 |
| AD | Inferior parietal | rs2081545 | A | C | -0.01787 | 0.00223 | 1.11E-15 | -0.0008 | 0.0006 | 2.01E-01 |
| AD | Inferior parietal | rs28394864 | A | G | 0.01230 | 0.00218 | 1.68E-08 | 0.0001 | 0.0006 | 8.41E-01 |
| AD | Inferior parietal | rs28399657 | G | A | -0.05464 | 0.00658 | 9.82E-17 | -0.0003 | 0.0017 | 8.73E-01 |
| AD | Inferior parietal | rs41289512 | G | C | 0.20630 | 0.00578 | 1.00E-200 | -0.0001 | 0.0018 | 9.48E-01 |
| AD | Inferior parietal | rs41290120 | A | G | -0.09905 | 0.00578 | 7.14E-66 | 0.0001 | 0.0015 | 9.33E-01 |
| AD | Inferior parietal | rs4236673 | A | G | -0.02016 | 0.00223 | 1.48E-19 | 0.0001 | 0.0006 | 8.14E-01 |
| AD | Inferior parietal | rs442495 | C | T | -0.01372 | 0.00226 | 1.22E-09 | 0.0008 | 0.0006 | 1.77E-01 |
| AD | Inferior parietal | rs4575098 | A | G | 0.01641 | 0.00258 | 1.90E-10 | -0.0005 | 0.0007 | 4.69E-01 |
| AD | Inferior parietal | rs4663105 | C | A | 0.03110 | 0.00222 | 1.45E-44 | -0.0010 | 0.0006 | 8.24E-02 |
| AD | Inferior parietal | rs59735493 | A | G | -0.01299 | 0.00236 | 3.73E-08 | 0.0018 | 0.0006 | 2.79E-03 |
| AD | Inferior parietal | rs6014724 | G | A | -0.02289 | 0.00369 | 5.38E-10 | 0.0016 | 0.0011 | 1.34E-01 |
| AD | Inferior parietal | rs6448453 | A | G | 0.01470 | 0.00245 | 1.98E-09 | 0.0018 | 0.0007 | 8.35E-03 |
| AD | Inferior parietal | rs679515 | T | C | 0.02542 | 0.00286 | 6.83E-19 | -0.0002 | 0.0008 | 7.75E-01 |
| AD | Inferior parietal | rs755951 | C | A | 0.01500 | 0.00221 | 1.13E-11 | 0.0010 | 0.0006 | 8.10E-02 |
| AD | Inferior parietal | rs7810606 | T | C | -0.01452 | 0.00218 | 2.89E-11 | 0.0001 | 0.0006 | 8.71E-01 |
| AD | Inferior parietal | rs846881 | C | A | -0.01737 | 0.00269 | 9.89E-11 | -0.0004 | 0.0008 | 6.49E-01 |
| AD | Inferior parietal | rs867611 | G | A | -0.02043 | 0.00232 | 1.48E-18 | -0.0005 | 0.0006 | 3.99E-01 |
| AD | Inferior parietal | rs9381563 | C | T | 0.01445 | 0.00227 | 1.99E-10 | 0.0001 | 0.0006 | 9.03E-01 |
| AD | Isthmus cingulate | rs10933431 | G | C | -0.01544 | 0.00251 | 7.62E-10 | 0.0023 | 0.0017 | 1.81E-01 |
| AD | Isthmus cingulate | rs111278892 | G | C | 0.01991 | 0.00305 | 6.67E-11 | 0.0024 | 0.0020 | 2.39E-01 |
| AD | Isthmus cingulate | rs11218343 | C | T | -0.03593 | 0.00526 | 8.12E-12 | 0.0050 | 0.0034 | 1.40E-01 |
| AD | Isthmus cingulate | rs11257238 | C | T | 0.01294 | 0.00226 | 1.04E-08 | 0.0006 | 0.0014 | 6.92E-01 |
| AD | Isthmus cingulate | rs113260531 | A | G | 0.01999 | 0.00325 | 7.91E-10 | 0.0014 | 0.0020 | 4.93E-01 |
| AD | Isthmus cingulate | rs118170342 | C | T | 0.14754 | 0.00570 | 7.93E-148 | -0.0007 | 0.0041 | 8.59E-01 |
| AD | Isthmus cingulate | rs12590654 | A | G | -0.01483 | 0.00231 | 1.32E-10 | 0.0013 | 0.0014 | 3.56E-01 |
| AD | Isthmus cingulate | rs1859788 | A | G | -0.01840 | 0.00231 | 1.80E-15 | 0.0017 | 0.0015 | 2.37E-01 |
| AD | Isthmus cingulate | rs204473 | A | G | -0.04167 | 0.00700 | 2.58E-09 | -0.0042 | 0.0043 | 3.33E-01 |
| AD | Isthmus cingulate | rs2081545 | A | C | -0.01787 | 0.00223 | 1.11E-15 | -0.0023 | 0.0013 | 7.63E-02 |
| AD | Isthmus cingulate | rs28394864 | A | G | 0.01230 | 0.00218 | 1.68E-08 | -0.0005 | 0.0013 | 7.21E-01 |
| AD | Isthmus cingulate | rs28399657 | G | A | -0.05464 | 0.00658 | 9.82E-17 | 0.0046 | 0.0039 | 2.36E-01 |
| AD | Isthmus cingulate | rs41289512 | G | C | 0.20630 | 0.00578 | 1.00E-200 | -0.0026 | 0.0040 | 5.26E-01 |
| AD | Isthmus cingulate | rs41290120 | A | G | -0.09905 | 0.00578 | 7.14E-66 | 0.0016 | 0.0033 | 6.34E-01 |
| AD | Isthmus cingulate | rs4236673 | A | G | -0.02016 | 0.00223 | 1.48E-19 | 0.0003 | 0.0014 | 8.44E-01 |
| AD | Isthmus cingulate | rs442495 | C | T | -0.01372 | 0.00226 | 1.22E-09 | 0.0012 | 0.0014 | 4.04E-01 |
| AD | Isthmus cingulate | rs4575098 | A | G | 0.01641 | 0.00258 | 1.90E-10 | 0.0005 | 0.0016 | 7.44E-01 |
| AD | Isthmus cingulate | rs4663105 | C | A | 0.03110 | 0.00222 | 1.45E-44 | -0.0015 | 0.0014 | 2.69E-01 |
| AD | Isthmus cingulate | rs59735493 | A | G | -0.01299 | 0.00236 | 3.73E-08 | 0.0006 | 0.0015 | 6.75E-01 |
| AD | Isthmus cingulate | rs6014724 | G | A | -0.02289 | 0.00369 | 5.38E-10 | 0.0006 | 0.0024 | 8.15E-01 |
| AD | Isthmus cingulate | rs6448453 | A | G | 0.01470 | 0.00245 | 1.98E-09 | -0.0029 | 0.0015 | 4.78E-02 |
| AD | Isthmus cingulate | rs679515 | T | C | 0.02542 | 0.00286 | 6.83E-19 | 0.0029 | 0.0017 | 9.81E-02 |
| AD | Isthmus cingulate | rs755951 | C | A | 0.01500 | 0.00221 | 1.13E-11 | 0.0012 | 0.0013 | 3.60E-01 |
| AD | Isthmus cingulate | rs7810606 | T | C | -0.01452 | 0.00218 | 2.89E-11 | 0.0000 | 0.0014 | 9.84E-01 |
| AD | Isthmus cingulate | rs846881 | C | A | -0.01737 | 0.00269 | 9.89E-11 | -0.0011 | 0.0018 | 5.43E-01 |
| AD | Isthmus cingulate | rs867611 | G | A | -0.02043 | 0.00232 | 1.48E-18 | 0.0010 | 0.0014 | 4.80E-01 |
| AD | Isthmus cingulate | rs9381563 | C | T | 0.01445 | 0.00227 | 1.99E-10 | -0.0006 | 0.0014 | 6.43E-01 |
| AD | postcentral | rs10933431 | G | C | -0.01544 | 0.00251 | 7.62E-10 | -0.0009 | 0.0009 | 3.15E-01 |
| AD | postcentral | rs111278892 | G | C | 0.01991 | 0.00305 | 6.67E-11 | 0.0002 | 0.0011 | 8.13E-01 |
| AD | postcentral | rs11218343 | C | T | -0.03593 | 0.00526 | 8.12E-12 | -0.0022 | 0.0017 | 2.10E-01 |
| AD | postcentral | rs11257238 | C | T | 0.01294 | 0.00226 | 1.04E-08 | 0.0002 | 0.0008 | 7.99E-01 |
| AD | postcentral | rs113260531 | A | G | 0.01999 | 0.00325 | 7.91E-10 | 0.0019 | 0.0010 | 6.38E-02 |
| AD | postcentral | rs118170342 | C | T | 0.14754 | 0.00570 | 7.93E-148 | 0.0013 | 0.0021 | 5.41E-01 |
| AD | postcentral | rs12590654 | A | G | -0.01483 | 0.00231 | 1.32E-10 | 0.0007 | 0.0008 | 3.81E-01 |
| AD | postcentral | rs1859788 | A | G | -0.01840 | 0.00231 | 1.80E-15 | 0.0023 | 0.0008 | 2.59E-03 |
| AD | postcentral | rs204473 | A | G | -0.04167 | 0.00700 | 2.58E-09 | -0.0011 | 0.0023 | 6.34E-01 |
| AD | postcentral | rs2081545 | A | C | -0.01787 | 0.00223 | 1.11E-15 | -0.0012 | 0.0007 | 7.68E-02 |
| AD | postcentral | rs28394864 | A | G | 0.01230 | 0.00218 | 1.68E-08 | 0.0012 | 0.0007 | 9.08E-02 |
| AD | postcentral | rs28399657 | G | A | -0.05464 | 0.00658 | 9.82E-17 | 0.0017 | 0.0020 | 3.94E-01 |
| AD | postcentral | rs41289512 | G | C | 0.20630 | 0.00578 | 1.00E-200 | 0.0012 | 0.0021 | 5.82E-01 |
| AD | postcentral | rs41290120 | A | G | -0.09905 | 0.00578 | 7.14E-66 | 0.0005 | 0.0017 | 7.64E-01 |
| AD | postcentral | rs4236673 | A | G | -0.02016 | 0.00223 | 1.48E-19 | -0.0006 | 0.0007 | 4.14E-01 |
| AD | postcentral | rs442495 | C | T | -0.01372 | 0.00226 | 1.22E-09 | 0.0010 | 0.0007 | 1.50E-01 |
| AD | postcentral | rs4575098 | A | G | 0.01641 | 0.00258 | 1.90E-10 | -0.0012 | 0.0008 | 1.31E-01 |
| AD | postcentral | rs4663105 | C | A | 0.03110 | 0.00222 | 1.45E-44 | 0.0001 | 0.0007 | 9.14E-01 |
| AD | postcentral | rs59735493 | A | G | -0.01299 | 0.00236 | 3.73E-08 | 0.0003 | 0.0008 | 6.57E-01 |
| AD | postcentral | rs6014724 | G | A | -0.02289 | 0.00369 | 5.38E-10 | -0.0012 | 0.0012 | 3.12E-01 |
| AD | postcentral | rs6448453 | A | G | 0.01470 | 0.00245 | 1.98E-09 | 0.0005 | 0.0008 | 5.53E-01 |
| AD | postcentral | rs679515 | T | C | 0.02542 | 0.00286 | 6.83E-19 | -0.0004 | 0.0009 | 6.80E-01 |
| AD | postcentral | rs755951 | C | A | 0.01500 | 0.00221 | 1.13E-11 | 0.0014 | 0.0007 | 4.15E-02 |
| AD | postcentral | rs7810606 | T | C | -0.01452 | 0.00218 | 2.89E-11 | 0.0004 | 0.0007 | 6.02E-01 |
| AD | postcentral | rs846881 | C | A | -0.01737 | 0.00269 | 9.89E-11 | 0.0007 | 0.0010 | 4.91E-01 |
| AD | postcentral | rs867611 | G | A | -0.02043 | 0.00232 | 1.48E-18 | -0.0005 | 0.0007 | 4.69E-01 |
| AD | postcentral | rs9381563 | C | T | 0.01445 | 0.00227 | 1.99E-10 | 0.0003 | 0.0007 | 6.79E-01 |
| AD | Posterior cingulate | rs10933431 | G | C | -0.01544 | 0.00251 | 7.62E-10 | 0.0007 | 0.0012 | 5.72E-01 |
| AD | Posterior cingulate | rs111278892 | G | C | 0.01991 | 0.00305 | 6.67E-11 | 0.0035 | 0.0014 | 1.31E-02 |
| AD | Posterior cingulate | rs11218343 | C | T | -0.03593 | 0.00526 | 8.12E-12 | -0.0008 | 0.0024 | 7.38E-01 |
| AD | Posterior cingulate | rs11257238 | C | T | 0.01294 | 0.00226 | 1.04E-08 | 0.0010 | 0.0010 | 3.11E-01 |
| AD | Posterior cingulate | rs113260531 | A | G | 0.01999 | 0.00325 | 7.91E-10 | -0.0008 | 0.0014 | 5.52E-01 |
| AD | Posterior cingulate | rs118170342 | C | T | 0.14754 | 0.00570 | 7.93E-148 | 0.0013 | 0.0029 | 6.51E-01 |
| AD | Posterior cingulate | rs12590654 | A | G | -0.01483 | 0.00231 | 1.32E-10 | -0.0004 | 0.0010 | 7.23E-01 |
| AD | Posterior cingulate | rs1859788 | A | G | -0.01840 | 0.00231 | 1.80E-15 | 0.0009 | 0.0011 | 3.75E-01 |
| AD | Posterior cingulate | rs204473 | A | G | -0.04167 | 0.00700 | 2.58E-09 | -0.0041 | 0.0030 | 1.72E-01 |
| AD | Posterior cingulate | rs2081545 | A | C | -0.01787 | 0.00223 | 1.11E-15 | -0.0023 | 0.0009 | 1.49E-02 |
| AD | Posterior cingulate | rs28394864 | A | G | 0.01230 | 0.00218 | 1.68E-08 | -0.0002 | 0.0009 | 8.53E-01 |
| AD | Posterior cingulate | rs28399657 | G | A | -0.05464 | 0.00658 | 9.82E-17 | -0.0017 | 0.0027 | 5.19E-01 |
| AD | Posterior cingulate | rs41289512 | G | C | 0.20630 | 0.00578 | 1.00E-200 | -0.0025 | 0.0028 | 3.81E-01 |
| AD | Posterior cingulate | rs41290120 | A | G | -0.09905 | 0.00578 | 7.14E-66 | 0.0020 | 0.0023 | 3.87E-01 |
| AD | Posterior cingulate | rs4236673 | A | G | -0.02016 | 0.00223 | 1.48E-19 | 0.0001 | 0.0009 | 8.76E-01 |
| AD | Posterior cingulate | rs442495 | C | T | -0.01372 | 0.00226 | 1.22E-09 | 0.0002 | 0.0010 | 8.35E-01 |
| AD | Posterior cingulate | rs4575098 | A | G | 0.01641 | 0.00258 | 1.90E-10 | -0.0009 | 0.0011 | 3.89E-01 |
| AD | Posterior cingulate | rs4663105 | C | A | 0.03110 | 0.00222 | 1.45E-44 | 0.0007 | 0.0010 | 4.69E-01 |
| AD | Posterior cingulate | rs59735493 | A | G | -0.01299 | 0.00236 | 3.73E-08 | -0.0005 | 0.0010 | 6.10E-01 |
| AD | Posterior cingulate | rs6014724 | G | A | -0.02289 | 0.00369 | 5.38E-10 | 0.0006 | 0.0016 | 7.31E-01 |
| AD | Posterior cingulate | rs6448453 | A | G | 0.01470 | 0.00245 | 1.98E-09 | -0.0016 | 0.0010 | 1.13E-01 |
| AD | Posterior cingulate | rs679515 | T | C | 0.02542 | 0.00286 | 6.83E-19 | -0.0005 | 0.0012 | 6.77E-01 |
| AD | Posterior cingulate | rs755951 | C | A | 0.01500 | 0.00221 | 1.13E-11 | 0.0008 | 0.0009 | 4.04E-01 |
| AD | Posterior cingulate | rs7810606 | T | C | -0.01452 | 0.00218 | 2.89E-11 | 0.0012 | 0.0009 | 2.00E-01 |
| AD | Posterior cingulate | rs846881 | C | A | -0.01737 | 0.00269 | 9.89E-11 | -0.0017 | 0.0013 | 1.73E-01 |
| AD | Posterior cingulate | rs867611 | G | A | -0.02043 | 0.00232 | 1.48E-18 | 0.0028 | 0.0010 | 4.46E-03 |
| AD | Posterior cingulate | rs9381563 | C | T | 0.01445 | 0.00227 | 1.99E-10 | -0.0003 | 0.0010 | 7.69E-01 |
| AD | precuneus | rs10933431 | G | C | -0.01544 | 0.00251 | 7.62E-10 | 0.0006 | 0.0008 | 4.52E-01 |
| AD | precuneus | rs111278892 | G | C | 0.01991 | 0.00305 | 6.67E-11 | -0.0007 | 0.0009 | 4.67E-01 |
| AD | precuneus | rs11218343 | C | T | -0.03593 | 0.00526 | 8.12E-12 | -0.0014 | 0.0016 | 3.78E-01 |
| AD | precuneus | rs11257238 | C | T | 0.01294 | 0.00226 | 1.04E-08 | 0.0007 | 0.0007 | 3.23E-01 |
| AD | precuneus | rs113260531 | A | G | 0.01999 | 0.00325 | 7.91E-10 | -0.0005 | 0.0009 | 6.03E-01 |
| AD | precuneus | rs118170342 | C | T | 0.14754 | 0.00570 | 7.93E-148 | 0.0021 | 0.0019 | 2.71E-01 |
| AD | precuneus | rs12590654 | A | G | -0.01483 | 0.00231 | 1.32E-10 | -0.0013 | 0.0007 | 4.92E-02 |
| AD | precuneus | rs1859788 | A | G | -0.01840 | 0.00231 | 1.80E-15 | 0.0012 | 0.0007 | 7.17E-02 |
| AD | precuneus | rs204473 | A | G | -0.04167 | 0.00700 | 2.58E-09 | -0.0022 | 0.0020 | 2.83E-01 |
| AD | precuneus | rs2081545 | A | C | -0.01787 | 0.00223 | 1.11E-15 | -0.0001 | 0.0006 | 8.17E-01 |
| AD | precuneus | rs28394864 | A | G | 0.01230 | 0.00218 | 1.68E-08 | 0.0002 | 0.0006 | 7.60E-01 |
| AD | precuneus | rs28399657 | G | A | -0.05464 | 0.00658 | 9.82E-17 | 0.0010 | 0.0018 | 5.86E-01 |
| AD | precuneus | rs41289512 | G | C | 0.20630 | 0.00578 | 1.00E-200 | 0.0023 | 0.0019 | 2.27E-01 |
| AD | precuneus | rs41290120 | A | G | -0.09905 | 0.00578 | 7.14E-66 | -0.0023 | 0.0016 | 1.44E-01 |
| AD | precuneus | rs4236673 | A | G | -0.02016 | 0.00223 | 1.48E-19 | 0.0009 | 0.0006 | 1.48E-01 |
| AD | precuneus | rs442495 | C | T | -0.01372 | 0.00226 | 1.22E-09 | 0.0004 | 0.0007 | 5.89E-01 |
| AD | precuneus | rs4575098 | A | G | 0.01641 | 0.00258 | 1.90E-10 | -0.0002 | 0.0007 | 7.91E-01 |
| AD | precuneus | rs4663105 | C | A | 0.03110 | 0.00222 | 1.45E-44 | -0.0001 | 0.0007 | 8.28E-01 |
| AD | precuneus | rs59735493 | A | G | -0.01299 | 0.00236 | 3.73E-08 | 0.0001 | 0.0007 | 8.54E-01 |
| AD | precuneus | rs6014724 | G | A | -0.02289 | 0.00369 | 5.38E-10 | 0.0007 | 0.0011 | 5.11E-01 |
| AD | precuneus | rs6448453 | A | G | 0.01470 | 0.00245 | 1.98E-09 | 0.0005 | 0.0007 | 4.84E-01 |
| AD | precuneus | rs679515 | T | C | 0.02542 | 0.00286 | 6.83E-19 | -0.0002 | 0.0008 | 8.34E-01 |
| AD | precuneus | rs755951 | C | A | 0.01500 | 0.00221 | 1.13E-11 | 0.0010 | 0.0006 | 9.54E-02 |
| AD | precuneus | rs7810606 | T | C | -0.01452 | 0.00218 | 2.89E-11 | 0.0011 | 0.0006 | 8.10E-02 |
| AD | precuneus | rs846881 | C | A | -0.01737 | 0.00269 | 9.89E-11 | -0.0001 | 0.0008 | 8.65E-01 |
| AD | precuneus | rs867611 | G | A | -0.02043 | 0.00232 | 1.48E-18 | -0.0003 | 0.0007 | 6.83E-01 |
| AD | precuneus | rs9381563 | C | T | 0.01445 | 0.00227 | 1.99E-10 | -0.0005 | 0.0006 | 4.05E-01 |
| AD | Superior parietal | rs10933431 | G | C | -0.01544 | 0.00251 | 7.62E-10 | 0.0004 | 0.0008 | 6.51E-01 |
| AD | Superior parietal | rs111278892 | G | C | 0.01991 | 0.00305 | 6.67E-11 | -0.0014 | 0.0009 | 1.19E-01 |
| AD | Superior parietal | rs11218343 | C | T | -0.03593 | 0.00526 | 8.12E-12 | -0.0015 | 0.0016 | 3.39E-01 |
| AD | Superior parietal | rs11257238 | C | T | 0.01294 | 0.00226 | 1.04E-08 | 0.0002 | 0.0007 | 8.06E-01 |
| AD | Superior parietal | rs113260531 | A | G | 0.01999 | 0.00325 | 7.91E-10 | -0.0008 | 0.0009 | 4.23E-01 |
| AD | Superior parietal | rs118170342 | C | T | 0.14754 | 0.00570 | 7.93E-148 | 0.0036 | 0.0020 | 6.94E-02 |
| AD | Superior parietal | rs12590654 | A | G | -0.01483 | 0.00231 | 1.32E-10 | -0.0003 | 0.0007 | 6.74E-01 |
| AD | Superior parietal | rs1859788 | A | G | -0.01840 | 0.00231 | 1.80E-15 | 0.0012 | 0.0007 | 8.82E-02 |
| AD | Superior parietal | rs204473 | A | G | -0.04167 | 0.00700 | 2.58E-09 | -0.0007 | 0.0020 | 7.26E-01 |
| AD | Superior parietal | rs2081545 | A | C | -0.01787 | 0.00223 | 1.11E-15 | -0.0008 | 0.0006 | 2.20E-01 |
| AD | Superior parietal | rs28394864 | A | G | 0.01230 | 0.00218 | 1.68E-08 | -0.0002 | 0.0006 | 7.17E-01 |
| AD | Superior parietal | rs28399657 | G | A | -0.05464 | 0.00658 | 9.82E-17 | -0.0018 | 0.0018 | 3.04E-01 |
| AD | Superior parietal | rs41289512 | G | C | 0.20630 | 0.00578 | 1.00E-200 | 0.0013 | 0.0019 | 4.87E-01 |
| AD | Superior parietal | rs41290120 | A | G | -0.09905 | 0.00578 | 7.14E-66 | -0.0028 | 0.0015 | 7.00E-02 |
| AD | Superior parietal | rs4236673 | A | G | -0.02016 | 0.00223 | 1.48E-19 | 0.0007 | 0.0006 | 2.33E-01 |
| AD | Superior parietal | rs442495 | C | T | -0.01372 | 0.00226 | 1.22E-09 | 0.0008 | 0.0006 | 2.01E-01 |
| AD | Superior parietal | rs4575098 | A | G | 0.01641 | 0.00258 | 1.90E-10 | -0.0007 | 0.0007 | 3.20E-01 |
| AD | Superior parietal | rs4663105 | C | A | 0.03110 | 0.00222 | 1.45E-44 | -0.0002 | 0.0006 | 7.42E-01 |
| AD | Superior parietal | rs59735493 | A | G | -0.01299 | 0.00236 | 3.73E-08 | 0.0013 | 0.0007 | 6.42E-02 |
| AD | Superior parietal | rs6014724 | G | A | -0.02289 | 0.00369 | 5.38E-10 | 0.0018 | 0.0011 | 1.05E-01 |
| AD | Superior parietal | rs6448453 | A | G | 0.01470 | 0.00245 | 1.98E-09 | 0.0013 | 0.0007 | 6.56E-02 |
| AD | Superior parietal | rs679515 | T | C | 0.02542 | 0.00286 | 6.83E-19 | -0.0005 | 0.0008 | 5.15E-01 |
| AD | Superior parietal | rs755951 | C | A | 0.01500 | 0.00221 | 1.13E-11 | 0.0009 | 0.0006 | 1.31E-01 |
| AD | Superior parietal | rs7810606 | T | C | -0.01452 | 0.00218 | 2.89E-11 | 0.0009 | 0.0006 | 1.34E-01 |
| AD | Superior parietal | rs846881 | C | A | -0.01737 | 0.00269 | 9.89E-11 | 0.0002 | 0.0008 | 8.22E-01 |
| AD | Superior parietal | rs867611 | G | A | -0.02043 | 0.00232 | 1.48E-18 | -0.0012 | 0.0006 | 5.80E-02 |
| AD | Superior parietal | rs9381563 | C | T | 0.01445 | 0.00227 | 1.99E-10 | -0.0002 | 0.0006 | 7.02E-01 |
| AD | supramarginal | rs10933431 | G | C | -0.01544 | 0.00251 | 7.62E-10 | 0.0001 | 0.0008 | 9.00E-01 |
| AD | supramarginal | rs111278892 | G | C | 0.01991 | 0.00305 | 6.67E-11 | 0.0007 | 0.0009 | 4.08E-01 |
| AD | supramarginal | rs11218343 | C | T | -0.03593 | 0.00526 | 8.12E-12 | 0.0014 | 0.0015 | 3.44E-01 |
| AD | supramarginal | rs11257238 | C | T | 0.01294 | 0.00226 | 1.04E-08 | 0.0010 | 0.0006 | 1.13E-01 |
| AD | supramarginal | rs113260531 | A | G | 0.01999 | 0.00325 | 7.91E-10 | -0.0002 | 0.0009 | 8.30E-01 |
| AD | supramarginal | rs118170342 | C | T | 0.14754 | 0.00570 | 7.93E-148 | -0.0009 | 0.0018 | 6.33E-01 |
| AD | supramarginal | rs12590654 | A | G | -0.01483 | 0.00231 | 1.32E-10 | -0.0003 | 0.0007 | 6.83E-01 |
| AD | supramarginal | rs1859788 | A | G | -0.01840 | 0.00231 | 1.80E-15 | 0.0004 | 0.0007 | 4.96E-01 |
| AD | supramarginal | rs204473 | A | G | -0.04167 | 0.00700 | 2.58E-09 | 0.0017 | 0.0019 | 3.78E-01 |
| AD | supramarginal | rs2081545 | A | C | -0.01787 | 0.00223 | 1.11E-15 | -0.0001 | 0.0006 | 9.29E-01 |
| AD | supramarginal | rs28394864 | A | G | 0.01230 | 0.00218 | 1.68E-08 | 0.0001 | 0.0006 | 9.19E-01 |
| AD | supramarginal | rs28399657 | G | A | -0.05464 | 0.00658 | 9.82E-17 | 0.0010 | 0.0017 | 5.72E-01 |
| AD | supramarginal | rs41289512 | G | C | 0.20630 | 0.00578 | 1.00E-200 | 0.0012 | 0.0018 | 5.15E-01 |
| AD | supramarginal | rs41290120 | A | G | -0.09905 | 0.00578 | 7.14E-66 | -0.0010 | 0.0015 | 5.25E-01 |
| AD | supramarginal | rs4236673 | A | G | -0.02016 | 0.00223 | 1.48E-19 | 0.0003 | 0.0006 | 6.19E-01 |
| AD | supramarginal | rs442495 | C | T | -0.01372 | 0.00226 | 1.22E-09 | 0.0004 | 0.0006 | 4.95E-01 |
| AD | supramarginal | rs4575098 | A | G | 0.01641 | 0.00258 | 1.90E-10 | 0.0014 | 0.0007 | 4.30E-02 |
| AD | supramarginal | rs4663105 | C | A | 0.03110 | 0.00222 | 1.45E-44 | -0.0009 | 0.0006 | 1.77E-01 |
| AD | supramarginal | rs59735493 | A | G | -0.01299 | 0.00236 | 3.73E-08 | 0.0013 | 0.0007 | 4.19E-02 |
| AD | supramarginal | rs6014724 | G | A | -0.02289 | 0.00369 | 5.38E-10 | -0.0010 | 0.0011 | 3.27E-01 |
| AD | supramarginal | rs6448453 | A | G | 0.01470 | 0.00245 | 1.98E-09 | -0.0002 | 0.0007 | 7.94E-01 |
| AD | supramarginal | rs679515 | T | C | 0.02542 | 0.00286 | 6.83E-19 | 0.0008 | 0.0008 | 2.89E-01 |
| AD | supramarginal | rs755951 | C | A | 0.01500 | 0.00221 | 1.13E-11 | 0.0011 | 0.0006 | 6.89E-02 |
| AD | supramarginal | rs7810606 | T | C | -0.01452 | 0.00218 | 2.89E-11 | 0.0007 | 0.0006 | 2.51E-01 |
| AD | supramarginal | rs846881 | C | A | -0.01737 | 0.00269 | 9.89E-11 | -0.0007 | 0.0008 | 3.62E-01 |
| AD | supramarginal | rs867611 | G | A | -0.02043 | 0.00232 | 1.48E-18 | 0.0005 | 0.0006 | 4.42E-01 |
| AD | supramarginal | rs9381563 | C | T | 0.01445 | 0.00227 | 1.99E-10 | -0.0009 | 0.0006 | 1.49E-01 |
| AD | cuneus | rs10933431 | G | C | -0.01544 | 0.00251 | 7.62E-10 | 0.0005 | 0.0011 | 6.31E-01 |
| AD | cuneus | rs111278892 | G | C | 0.01991 | 0.00305 | 6.67E-11 | 0.0002 | 0.0013 | 8.64E-01 |
| AD | cuneus | rs11218343 | C | T | -0.03593 | 0.00526 | 8.12E-12 | -0.0022 | 0.0021 | 2.98E-01 |
| AD | cuneus | rs11257238 | C | T | 0.01294 | 0.00226 | 1.04E-08 | 0.0004 | 0.0009 | 6.19E-01 |
| AD | cuneus | rs113260531 | A | G | 0.01999 | 0.00325 | 7.91E-10 | 0.0018 | 0.0013 | 1.66E-01 |
| AD | cuneus | rs118170342 | C | T | 0.14754 | 0.00570 | 7.93E-148 | 0.0002 | 0.0026 | 9.31E-01 |
| AD | cuneus | rs12590654 | A | G | -0.01483 | 0.00231 | 1.32E-10 | -0.0009 | 0.0009 | 3.16E-01 |
| AD | cuneus | rs1859788 | A | G | -0.01840 | 0.00231 | 1.80E-15 | 0.0005 | 0.0009 | 5.88E-01 |
| AD | cuneus | rs204473 | A | G | -0.04167 | 0.00700 | 2.58E-09 | 0.0011 | 0.0027 | 6.78E-01 |
| AD | cuneus | rs2081545 | A | C | -0.01787 | 0.00223 | 1.11E-15 | -0.0006 | 0.0009 | 4.92E-01 |
| AD | cuneus | rs28394864 | A | G | 0.01230 | 0.00218 | 1.68E-08 | 0.0008 | 0.0009 | 3.69E-01 |
| AD | cuneus | rs28399657 | G | A | -0.05464 | 0.00658 | 9.82E-17 | 0.0004 | 0.0024 | 8.70E-01 |
| AD | cuneus | rs41289512 | G | C | 0.20630 | 0.00578 | 1.00E-200 | 0.0032 | 0.0025 | 2.04E-01 |
| AD | cuneus | rs41290120 | A | G | -0.09905 | 0.00578 | 7.14E-66 | -0.0004 | 0.0021 | 8.45E-01 |
| AD | cuneus | rs4236673 | A | G | -0.02016 | 0.00223 | 1.48E-19 | -0.0003 | 0.0009 | 7.12E-01 |
| AD | cuneus | rs442495 | C | T | -0.01372 | 0.00226 | 1.22E-09 | -0.0018 | 0.0009 | 4.40E-02 |
| AD | cuneus | rs4575098 | A | G | 0.01641 | 0.00258 | 1.90E-10 | 0.0005 | 0.0010 | 6.17E-01 |
| AD | cuneus | rs4663105 | C | A | 0.03110 | 0.00222 | 1.45E-44 | 0.0001 | 0.0009 | 8.69E-01 |
| AD | cuneus | rs59735493 | A | G | -0.01299 | 0.00236 | 3.73E-08 | -0.0009 | 0.0009 | 2.93E-01 |
| AD | cuneus | rs6014724 | G | A | -0.02289 | 0.00369 | 5.38E-10 | 0.0018 | 0.0015 | 2.39E-01 |
| AD | cuneus | rs6448453 | A | G | 0.01470 | 0.00245 | 1.98E-09 | 0.0001 | 0.0010 | 8.75E-01 |
| AD | cuneus | rs679515 | T | C | 0.02542 | 0.00286 | 6.83E-19 | 0.0015 | 0.0011 | 1.54E-01 |
| AD | cuneus | rs755951 | C | A | 0.01500 | 0.00221 | 1.13E-11 | 0.0006 | 0.0009 | 5.04E-01 |
| AD | cuneus | rs7810606 | T | C | -0.01452 | 0.00218 | 2.89E-11 | 0.0006 | 0.0009 | 4.83E-01 |
| AD | cuneus | rs846881 | C | A | -0.01737 | 0.00269 | 9.89E-11 | -0.0004 | 0.0011 | 7.19E-01 |
| AD | cuneus | rs867611 | G | A | -0.02043 | 0.00232 | 1.48E-18 | -0.0004 | 0.0009 | 6.08E-01 |
| AD | cuneus | rs9381563 | C | T | 0.01445 | 0.00227 | 1.99E-10 | 0.0002 | 0.0009 | 7.85E-01 |
| AD | Lateral occipital | rs10933431 | G | C | -0.01544 | 0.00251 | 7.62E-10 | -0.0009 | 0.0009 | 3.45E-01 |
| AD | Lateral occipital | rs111278892 | G | C | 0.01991 | 0.00305 | 6.67E-11 | 0.0005 | 0.0011 | 6.38E-01 |
| AD | Lateral occipital | rs11218343 | C | T | -0.03593 | 0.00526 | 8.12E-12 | 0.0009 | 0.0018 | 6.06E-01 |
| AD | Lateral occipital | rs11257238 | C | T | 0.01294 | 0.00226 | 1.04E-08 | -0.0007 | 0.0008 | 3.32E-01 |
| AD | Lateral occipital | rs113260531 | A | G | 0.01999 | 0.00325 | 7.91E-10 | 0.0003 | 0.0010 | 7.51E-01 |
| AD | Lateral occipital | rs118170342 | C | T | 0.14754 | 0.00570 | 7.93E-148 | 0.0026 | 0.0022 | 2.36E-01 |
| AD | Lateral occipital | rs12590654 | A | G | -0.01483 | 0.00231 | 1.32E-10 | -0.0006 | 0.0008 | 4.07E-01 |
| AD | Lateral occipital | rs1859788 | A | G | -0.01840 | 0.00231 | 1.80E-15 | -0.0008 | 0.0008 | 3.29E-01 |
| AD | Lateral occipital | rs204473 | A | G | -0.04167 | 0.00700 | 2.58E-09 | -0.0007 | 0.0022 | 7.46E-01 |
| AD | Lateral occipital | rs2081545 | A | C | -0.01787 | 0.00223 | 1.11E-15 | 0.0001 | 0.0007 | 8.42E-01 |
| AD | Lateral occipital | rs28394864 | A | G | 0.01230 | 0.00218 | 1.68E-08 | 0.0007 | 0.0007 | 2.85E-01 |
| AD | Lateral occipital | rs28399657 | G | A | -0.05464 | 0.00658 | 9.82E-17 | -0.0042 | 0.0020 | 4.04E-02 |
| AD | Lateral occipital | rs41289512 | G | C | 0.20630 | 0.00578 | 1.00E-200 | 0.0046 | 0.0021 | 3.10E-02 |
| AD | Lateral occipital | rs41290120 | A | G | -0.09905 | 0.00578 | 7.14E-66 | -0.0028 | 0.0017 | 1.03E-01 |
| AD | Lateral occipital | rs4236673 | A | G | -0.02016 | 0.00223 | 1.48E-19 | 0.0002 | 0.0007 | 7.43E-01 |
| AD | Lateral occipital | rs442495 | C | T | -0.01372 | 0.00226 | 1.22E-09 | -0.0008 | 0.0007 | 2.47E-01 |
| AD | Lateral occipital | rs4575098 | A | G | 0.01641 | 0.00258 | 1.90E-10 | -0.0007 | 0.0008 | 3.78E-01 |
| AD | Lateral occipital | rs4663105 | C | A | 0.03110 | 0.00222 | 1.45E-44 | -0.0010 | 0.0007 | 1.46E-01 |
| AD | Lateral occipital | rs59735493 | A | G | -0.01299 | 0.00236 | 3.73E-08 | 0.0007 | 0.0008 | 3.87E-01 |
| AD | Lateral occipital | rs6014724 | G | A | -0.02289 | 0.00369 | 5.38E-10 | 0.0021 | 0.0012 | 8.38E-02 |
| AD | Lateral occipital | rs6448453 | A | G | 0.01470 | 0.00245 | 1.98E-09 | 0.0013 | 0.0008 | 1.04E-01 |
| AD | Lateral occipital | rs679515 | T | C | 0.02542 | 0.00286 | 6.83E-19 | 0.0019 | 0.0009 | 3.11E-02 |
| AD | Lateral occipital | rs755951 | C | A | 0.01500 | 0.00221 | 1.13E-11 | 0.0000 | 0.0007 | 9.46E-01 |
| AD | Lateral occipital | rs7810606 | T | C | -0.01452 | 0.00218 | 2.89E-11 | 0.0001 | 0.0007 | 9.03E-01 |
| AD | Lateral occipital | rs846881 | C | A | -0.01737 | 0.00269 | 9.89E-11 | -0.0005 | 0.0009 | 5.65E-01 |
| AD | Lateral occipital | rs867611 | G | A | -0.02043 | 0.00232 | 1.48E-18 | -0.0011 | 0.0007 | 1.29E-01 |
| AD | Lateral occipital | rs9381563 | C | T | 0.01445 | 0.00227 | 1.99E-10 | 0.0001 | 0.0007 | 9.23E-01 |
| AD | lingual | rs10933431 | G | C | -0.01544 | 0.00251 | 7.62E-10 | -0.0003 | 0.0010 | 7.21E-01 |
| AD | lingual | rs111278892 | G | C | 0.01991 | 0.00305 | 6.67E-11 | 0.0005 | 0.0011 | 6.76E-01 |
| AD | lingual | rs11218343 | C | T | -0.03593 | 0.00526 | 8.12E-12 | -0.0021 | 0.0018 | 2.44E-01 |
| AD | lingual | rs11257238 | C | T | 0.01294 | 0.00226 | 1.04E-08 | 0.0002 | 0.0008 | 8.02E-01 |
| AD | lingual | rs113260531 | A | G | 0.01999 | 0.00325 | 7.91E-10 | 0.0016 | 0.0011 | 1.50E-01 |
| AD | lingual | rs118170342 | C | T | 0.14754 | 0.00570 | 7.93E-148 | -0.0008 | 0.0023 | 7.39E-01 |
| AD | lingual | rs12590654 | A | G | -0.01483 | 0.00231 | 1.32E-10 | 0.0000 | 0.0008 | 9.73E-01 |
| AD | lingual | rs1859788 | A | G | -0.01840 | 0.00231 | 1.80E-15 | -0.0005 | 0.0008 | 5.13E-01 |
| AD | lingual | rs204473 | A | G | -0.04167 | 0.00700 | 2.58E-09 | -0.0009 | 0.0024 | 6.99E-01 |
| AD | lingual | rs2081545 | A | C | -0.01787 | 0.00223 | 1.11E-15 | 0.0005 | 0.0008 | 4.77E-01 |
| AD | lingual | rs28394864 | A | G | 0.01230 | 0.00218 | 1.68E-08 | 0.0000 | 0.0007 | 9.51E-01 |
| AD | lingual | rs28399657 | G | A | -0.05464 | 0.00658 | 9.82E-17 | -0.0005 | 0.0021 | 8.21E-01 |
| AD | lingual | rs41289512 | G | C | 0.20630 | 0.00578 | 1.00E-200 | 0.0019 | 0.0022 | 3.79E-01 |
| AD | lingual | rs41290120 | A | G | -0.09905 | 0.00578 | 7.14E-66 | -0.0010 | 0.0018 | 5.86E-01 |
| AD | lingual | rs4236673 | A | G | -0.02016 | 0.00223 | 1.48E-19 | 0.0003 | 0.0008 | 6.82E-01 |
| AD | lingual | rs442495 | C | T | -0.01372 | 0.00226 | 1.22E-09 | -0.0009 | 0.0008 | 2.25E-01 |
| AD | lingual | rs4575098 | A | G | 0.01641 | 0.00258 | 1.90E-10 | 0.0005 | 0.0009 | 5.90E-01 |
| AD | lingual | rs4663105 | C | A | 0.03110 | 0.00222 | 1.45E-44 | 0.0014 | 0.0008 | 5.84E-02 |
| AD | lingual | rs59735493 | A | G | -0.01299 | 0.00236 | 3.73E-08 | -0.0010 | 0.0008 | 1.90E-01 |
| AD | lingual | rs6014724 | G | A | -0.02289 | 0.00369 | 5.38E-10 | -0.0008 | 0.0013 | 5.40E-01 |
| AD | lingual | rs6448453 | A | G | 0.01470 | 0.00245 | 1.98E-09 | -0.0003 | 0.0008 | 7.12E-01 |
| AD | lingual | rs679515 | T | C | 0.02542 | 0.00286 | 6.83E-19 | 0.0015 | 0.0009 | 1.17E-01 |
| AD | lingual | rs755951 | C | A | 0.01500 | 0.00221 | 1.13E-11 | -0.0007 | 0.0008 | 3.67E-01 |
| AD | lingual | rs7810606 | T | C | -0.01452 | 0.00218 | 2.89E-11 | 0.0006 | 0.0008 | 3.96E-01 |
| AD | lingual | rs846881 | C | A | -0.01737 | 0.00269 | 9.89E-11 | -0.0002 | 0.0010 | 8.34E-01 |
| AD | lingual | rs867611 | G | A | -0.02043 | 0.00232 | 1.48E-18 | -0.0004 | 0.0008 | 6.37E-01 |
| AD | lingual | rs9381563 | C | T | 0.01445 | 0.00227 | 1.99E-10 | 0.0005 | 0.0008 | 5.45E-01 |
| AD | pericalcarine | rs10933431 | G | C | -0.01544 | 0.00251 | 7.62E-10 | -0.0013 | 0.0011 | 2.25E-01 |
| AD | pericalcarine | rs111278892 | G | C | 0.01991 | 0.00305 | 6.67E-11 | -0.0010 | 0.0013 | 4.37E-01 |
| AD | pericalcarine | rs11218343 | C | T | -0.03593 | 0.00526 | 8.12E-12 | 0.0006 | 0.0021 | 7.65E-01 |
| AD | pericalcarine | rs11257238 | C | T | 0.01294 | 0.00226 | 1.04E-08 | 0.0003 | 0.0009 | 7.66E-01 |
| AD | pericalcarine | rs113260531 | A | G | 0.01999 | 0.00325 | 7.91E-10 | -0.0002 | 0.0013 | 8.65E-01 |
| AD | pericalcarine | rs118170342 | C | T | 0.14754 | 0.00570 | 7.93E-148 | -0.0016 | 0.0026 | 5.44E-01 |
| AD | pericalcarine | rs12590654 | A | G | -0.01483 | 0.00231 | 1.32E-10 | -0.0003 | 0.0009 | 7.12E-01 |
| AD | pericalcarine | rs1859788 | A | G | -0.01840 | 0.00231 | 1.80E-15 | -0.0019 | 0.0009 | 3.45E-02 |
| AD | pericalcarine | rs204473 | A | G | -0.04167 | 0.00700 | 2.58E-09 | -0.0038 | 0.0027 | 1.61E-01 |
| AD | pericalcarine | rs2081545 | A | C | -0.01787 | 0.00223 | 1.11E-15 | -0.0003 | 0.0008 | 7.20E-01 |
| AD | pericalcarine | rs28394864 | A | G | 0.01230 | 0.00218 | 1.68E-08 | 0.0012 | 0.0008 | 1.36E-01 |
| AD | pericalcarine | rs28399657 | G | A | -0.05464 | 0.00658 | 9.82E-17 | 0.0020 | 0.0024 | 3.95E-01 |
| AD | pericalcarine | rs41289512 | G | C | 0.20630 | 0.00578 | 1.00E-200 | 0.0007 | 0.0025 | 7.78E-01 |
| AD | pericalcarine | rs41290120 | A | G | -0.09905 | 0.00578 | 7.14E-66 | 0.0006 | 0.0021 | 7.59E-01 |
| AD | pericalcarine | rs4236673 | A | G | -0.02016 | 0.00223 | 1.48E-19 | 0.0018 | 0.0008 | 3.04E-02 |
| AD | pericalcarine | rs442495 | C | T | -0.01372 | 0.00226 | 1.22E-09 | -0.0012 | 0.0009 | 1.79E-01 |
| AD | pericalcarine | rs4575098 | A | G | 0.01641 | 0.00258 | 1.90E-10 | -0.0002 | 0.0009 | 7.92E-01 |
| AD | pericalcarine | rs4663105 | C | A | 0.03110 | 0.00222 | 1.45E-44 | 0.0015 | 0.0008 | 7.60E-02 |
| AD | pericalcarine | rs59735493 | A | G | -0.01299 | 0.00236 | 3.73E-08 | -0.0006 | 0.0009 | 5.21E-01 |
| AD | pericalcarine | rs6014724 | G | A | -0.02289 | 0.00369 | 5.38E-10 | -0.0014 | 0.0015 | 3.55E-01 |
| AD | pericalcarine | rs6448453 | A | G | 0.01470 | 0.00245 | 1.98E-09 | -0.0005 | 0.0009 | 5.59E-01 |
| AD | pericalcarine | rs679515 | T | C | 0.02542 | 0.00286 | 6.83E-19 | 0.0005 | 0.0010 | 6.55E-01 |
| AD | pericalcarine | rs755951 | C | A | 0.01500 | 0.00221 | 1.13E-11 | 0.0010 | 0.0008 | 2.18E-01 |
| AD | pericalcarine | rs7810606 | T | C | -0.01452 | 0.00218 | 2.89E-11 | -0.0004 | 0.0008 | 6.27E-01 |
| AD | pericalcarine | rs846881 | C | A | -0.01737 | 0.00269 | 9.89E-11 | -0.0004 | 0.0011 | 7.42E-01 |
| AD | pericalcarine | rs867611 | G | A | -0.02043 | 0.00232 | 1.48E-18 | -0.0018 | 0.0009 | 3.57E-02 |
| AD | pericalcarine | rs9381563 | C | T | 0.01445 | 0.00227 | 1.99E-10 | -0.0009 | 0.0008 | 3.07E-01 |
| AD | whole cortex | rs10933431 | G | C | -0.01544 | 0.00251 | 7.62E-10 | -0.0007 | 0.0010 | 4.50E-01 |
| AD | whole cortex | rs111278892 | G | C | 0.01991 | 0.00305 | 6.67E-11 | -0.0014 | 0.0011 | 2.16E-01 |
| AD | whole cortex | rs11218343 | C | T | -0.03593 | 0.00526 | 8.12E-12 | -0.0002 | 0.0019 | 9.30E-01 |
| AD | whole cortex | rs11257238 | C | T | 0.01294 | 0.00226 | 1.04E-08 | 0.0003 | 0.0008 | 6.97E-01 |
| AD | whole cortex | rs113260531 | A | G | 0.01999 | 0.00325 | 7.91E-10 | -0.0019 | 0.0011 | 8.37E-02 |
| AD | whole cortex | rs118170342 | C | T | 0.14754 | 0.00570 | 7.93E-148 | -0.0002 | 0.0023 | 9.34E-01 |
| AD | whole cortex | rs12590654 | A | G | -0.01483 | 0.00231 | 1.32E-10 | -0.0004 | 0.0008 | 5.95E-01 |
| AD | whole cortex | rs1859788 | A | G | -0.01840 | 0.00231 | 1.80E-15 | -0.0009 | 0.0008 | 2.61E-01 |
| AD | whole cortex | rs204473 | A | G | -0.04167 | 0.00700 | 2.58E-09 | 0.0037 | 0.0025 | 1.32E-01 |
| AD | whole cortex | rs2081545 | A | C | -0.01787 | 0.00223 | 1.11E-15 | 0.0010 | 0.0008 | 2.09E-01 |
| AD | whole cortex | rs28394864 | A | G | 0.01230 | 0.00218 | 1.68E-08 | -0.0004 | 0.0008 | 5.59E-01 |
| AD | whole cortex | rs28399657 | G | A | -0.05464 | 0.00658 | 9.82E-17 | -0.0026 | 0.0022 | 2.50E-01 |
| AD | whole cortex | rs41289512 | G | C | 0.20630 | 0.00578 | 1.00E-200 | 0.0017 | 0.0023 | 4.51E-01 |
| AD | whole cortex | rs41290120 | A | G | -0.09905 | 0.00578 | 7.14E-66 | -0.0006 | 0.0019 | 7.33E-01 |
| AD | whole cortex | rs4236673 | A | G | -0.02016 | 0.00223 | 1.48E-19 | 0.0008 | 0.0008 | 3.09E-01 |
| AD | whole cortex | rs442495 | C | T | -0.01372 | 0.00226 | 1.22E-09 | -0.0016 | 0.0008 | 4.02E-02 |
| AD | whole cortex | rs4575098 | A | G | 0.01641 | 0.00258 | 1.90E-10 | -0.0002 | 0.0009 | 8.06E-01 |
| AD | whole cortex | rs4663105 | C | A | 0.03110 | 0.00222 | 1.45E-44 | 0.0003 | 0.0008 | 6.55E-01 |
| AD | whole cortex | rs59735493 | A | G | -0.01299 | 0.00236 | 3.73E-08 | -0.0005 | 0.0008 | 5.41E-01 |
| AD | whole cortex | rs6014724 | G | A | -0.02289 | 0.00369 | 5.38E-10 | 0.0002 | 0.0013 | 8.99E-01 |
| AD | whole cortex | rs6448453 | A | G | 0.01470 | 0.00245 | 1.98E-09 | 0.0004 | 0.0009 | 6.50E-01 |
| AD | whole cortex | rs679515 | T | C | 0.02542 | 0.00286 | 6.83E-19 | -0.0011 | 0.0010 | 2.60E-01 |
| AD | whole cortex | rs755951 | C | A | 0.01500 | 0.00221 | 1.13E-11 | 0.0002 | 0.0008 | 7.68E-01 |
| AD | whole cortex | rs7810606 | T | C | -0.01452 | 0.00218 | 2.89E-11 | 0.0002 | 0.0008 | 7.53E-01 |
| AD | whole cortex | rs846881 | C | A | -0.01737 | 0.00269 | 9.89E-11 | -0.0007 | 0.0010 | 4.79E-01 |
| AD | whole cortex | rs867611 | G | A | -0.02043 | 0.00232 | 1.48E-18 | -0.0002 | 0.0008 | 8.29E-01 |
| AD | whole cortex | rs9381563 | C | T | 0.01445 | 0.00227 | 1.99E-10 | 0.0002 | 0.0008 | 7.88E-01 |

**Table S6.** Sensitivity analysis and pleiotropy analysis for causal effect of cortical surface area on Alzheimer’s Disease

| **Exposure** | **Lobe** | **Outcome** | **No. of SNPs** | **MR Egger** | | | **Weighted median** | | **MR PRESSO** | | **Horizontal pleiotropy** |
| --- | --- | --- | --- | --- | --- | --- | --- | --- | --- | --- | --- |
|  |  |  |  | I**^2^_GX_** | **OR (95%CI)** | **P** | **OR (95%CI)** | **P** | **Global test P** | **Correct P*** | **Egger Intercept P** |
| Whole cortex | global | AD | 29 |  | 1.05 (0.97, 1.14) | 0.258 | 1.02 (0.99, 1.05) | 0.205 | <0.001 | 0.253 | 0.333 |
| Caudal Anterior Cingulate | frontal | AD | 7 |  | 1.08 (0.94, 1.26) | 0.328 | 1.05 (0.99, 1.12) | 0.085 | 0.185 | NA | 0.493 |
| Caudal middle frontal | frontal | AD | 11 |  | 1.22 (1.04, 1.42) | 0.037 | 1.01 (0.96, 1.07) | 0.723 | 0.183 | NA | 0.049 |
| Frontal pole | frontal | AD | 6 |  | 1.03 (0.9, 1.17) | 0.672 | 0.99 (0.93, 1.05) | 0.69 | 0.223 | NA | 0.596 |
| Lateral orbitofrontal | frontal | AD | 21 | 0.97 | 0.96 (0.79, 1.17) | 0.695 | 1.04 (0.99, 1.09) | 0.168 | 0.629 | NA | 0.416 |
| Lateral orbitofrontal^ | frontal | AD | 20 | 0.97 | 0.94 (0.78, 1.15) | 0.568 | 1.03 (0.98, 1.09) | 0.218 | 0.875 | NA | 0.371 |
| Medial orbitofrontal | frontal | AD | 5 |  | 0.95 (0.77, 1.17) | 0.644 | 1.01 (0.92, 1.11) | 0.828 | 0.852 | NA | 0.493 |
| paracentral | frontal | AD | 9 |  | 1.07 (0.83, 1.38) | 0.624 | 0.99 (0.93, 1.05) | 0.73 | 0.4 | NA | 0.489 |
| Pars opercularis | frontal | AD | 7 |  | 1.1 (0.91, 1.33) | 0.371 | 1.07 (1.01, 1.13) | 0.031 | 0.023 | 0.075 | 0.59 |
| Pars orbitalis | frontal | AD | 11 |  | 0.94 (0.8, 1.12) | 0.508 | 0.97 (0.92, 1.02) | 0.268 | 0.726 | NA | 0.741 |
| Pars triangularis | frontal | AD | 17 |  | 0.96 (0.88, 1.05) | 0.388 | 0.97 (0.94, 1.01) | 0.143 | 0.033 | NA | 0.588 |
| Precentral | frontal | AD | 12 |  | 0.95 (0.9, 1) | 0.079 | 0.96 (0.93, 0.99) | 0.022 | 0.028 | NA | 0.119 |
| Rostral anterior cingulate | frontal | AD | 6 |  | 0.73 (0.47, 1.14) | 0.24 | 1.05 (0.97, 1.12) | 0.224 | 0.725 | NA | 0.177 |
| Rostral middle frontal | frontal | AD | 21 |  | 1.17 (0.97, 1.41) | 0.12 | 0.99 (0.92, 1.05) | 0.662 | 0.016 | NA | 0.106 |
| Superior frontal | frontal | AD | 14 |  | 1.02 (0.77, 1.35) | 0.895 | 0.93 (0.85, 1.02) | 0.13 | 0.682 | NA | 0.603 |
| banks of the superior temporal sulcus | temporal | AD | 8 |  | 1.01 (0.88, 1.15) | 0.917 | 0.96 (0.91, 1.01) | 0.154 | 0.357 | NA | 0.479 |
| entorhinal | temporal | AD | 12 |  | 0.96 (0.81, 1.14) | 0.64 | 0.98 (0.93, 1.02) | 0.323 | 0.238 | NA | 0.844 |
| fusiform | temporal | AD | 8 |  | 0.95 (0.69, 1.3) | 0.746 | 1 (0.92, 1.09) | 0.944 | 0.026 | NA | 0.831 |
| Inferior temporal | temporal | AD | 11 |  | 1.17 (0.87, 1.57) | 0.337 | 0.99 (0.92, 1.06) | 0.727 | 0.213 | NA | 0.379 |
| insula | temporal | AD | 15 |  | 0.93 (0.78, 1.12) | 0.463 | 0.98 (0.93, 1.04) | 0.579 | 0.196 | NA | 0.507 |
| Middle temporal | temporal | AD | 12 |  | 1.02 (0.84, 1.23) | 0.866 | 1.03 (0.96, 1.1) | 0.38 | 0.081 | NA | 0.886 |
| parahippocampal | temporal | AD | 8 |  | 1.03 (0.76, 1.4) | 0.861 | 1.01 (0.95, 1.08) | 0.778 | 0.127 | NA | 0.969 |
| Superior temporal | temporal | AD | 12 |  | 0.88 (0.71, 1.1) | 0.295 | 0.96 (0.9, 1.03) | 0.248 | 0.319 | NA | 0.486 |
| Temporal pole | temporal | AD | 4 | 0.97 | 0.89 (0.77, 1.02) | 0.235 | 0.95 (0.89, 1.02) | 0.161 | 0.711 | NA | 0.434 |
| Transverse temporal | temporal | AD | 14 |  | 1.07 (0.88, 1.31) | 0.495 | 0.99 (0.95, 1.03) | 0.644 | 0.024 | NA | 0.565 |
| Inferior parietal | parietal | AD | 19 |  | 1.04 (0.93, 1.17) | 0.486 | 1 (0.96, 1.05) | 0.94 | 0.175 | NA | 0.684 |
| Isthmus cingulate | parietal | AD | 7 |  | 1.26 (0.86, 1.85) | 0.292 | 1.05 (0.98, 1.13) | 0.171 | 0.692 | NA | 0.348 |
| postcentral | parietal | AD | 13 |  | 0.97 (0.9, 1.04) | 0.368 | 0.97 (0.93, 1.02) | 0.304 | 0.092 | NA | 0.533 |
| Posterior cingulate | parietal | AD | 9 |  | 1 (0.76, 1.32) | 0.997 | 1.01 (0.94, 1.08) | 0.878 | 0.059 | NA | 0.977 |
| precuneus | parietal | AD | 20 |  | 1.07 (0.92, 1.24) | 0.414 | 1 (0.94, 1.05) | 0.902 | 0.167 | NA | 0.25 |
| Superior parietal | parietal | AD | 18 |  | 1.03 (0.96, 1.11) | 0.406 | 0.99 (0.95, 1.04) | 0.771 | 0.666 | NA | 0.404 |
| supramarginal | parietal | AD | 11 | 0.98 | 1.08 (1, 1.18) | 0.081 | 1.07 (1.02, 1.12) | 0.009 | 0.443 | NA | 0.407 |
| cuneus | occipital | AD | 13 |  | 0.96 (0.86, 1.09) | 0.569 | 0.98 (0.94, 1.03) | 0.456 | 0.348 | NA | 0.502 |
| Lateral occipital | occipital | AD | 16 |  | 1.03 (0.9, 1.18) | 0.697 | 0.97 (0.92, 1.02) | 0.24 | 0.19 | NA | 0.595 |
| lingual | occipital | AD | 21 | 0.97 | 0.97 (0.85, 1.12) | 0.712 | 1.02 (0.99, 1.06) | 0.199 | 0.498 | NA | 0.426 |
| pericalcarine | occipital | AD | 38 |  | 1 (0.94, 1.06) | 0.929 | 1 (0.98, 1.03) | 0.735 | 0.034 | NA | 0.694 |

AD, Alzheimer’s Disease; SNP, single nucleotide polymorphism; OR: odds ratio genetically predicted 1-SD unit increase in the cortical surface area; CI: confidence interval; MR PRESSO, MR Pleiotropy Residual Sum and Outlier. I2GX is calculated to measure the violation of NO Measurement Error (NOME) assumption in MR Egger. I2GX < 0.9 means there is potential violation of NOME assumption in MR Egger. *If MR PRESSO Global test detect the horizontal pleiotropy and there is significant difference before and after removing outlier, correct P is calculated by removing instruments variants which have horizontal pleiotropy. ^ the corrected results after removing outlier SNP.

**Table S7.** Leave-one-out(loo) analysis of association between genetically predicted cortical surface area and Alzheimer's disease (AD) risk

| **exposure** | **outcome** | **SNP** | **b** | **se** | **p** |
| --- | --- | --- | --- | --- | --- |
| caudalanteriorcingulate | AD | rs10845985 | 0.04082 | 0.03019 | 1.76E-01 |
| caudalanteriorcingulate | AD | rs13021985 | 0.02362 | 0.03272 | 4.70E-01 |
| caudalanteriorcingulate | AD | rs2509765 | 0.03077 | 0.03336 | 3.56E-01 |
| caudalanteriorcingulate | AD | rs2647416 | 0.04887 | 0.02375 | 3.96E-02 |
| caudalanteriorcingulate | AD | rs4747503 | 0.01580 | 0.02854 | 5.80E-01 |
| caudalanteriorcingulate | AD | rs7728751 | 0.02545 | 0.03302 | 4.41E-01 |
| caudalanteriorcingulate | AD | rs80241863 | 0.02529 | 0.03442 | 4.62E-01 |
| caudalmiddlefrontal | AD | rs1261073 | 0.02265 | 0.02223 | 3.08E-01 |
| caudalmiddlefrontal | AD | rs2589230 | 0.01587 | 0.02256 | 4.82E-01 |
| caudalmiddlefrontal | AD | rs30641 | 0.01107 | 0.02188 | 6.13E-01 |
| caudalmiddlefrontal | AD | rs4273712 | -0.00084 | 0.02084 | 9.68E-01 |
| caudalmiddlefrontal | AD | rs448939 | 0.02678 | 0.01968 | 1.74E-01 |
| caudalmiddlefrontal | AD | rs655045 | 0.02394 | 0.02214 | 2.80E-01 |
| caudalmiddlefrontal | AD | rs7184835 | 0.02504 | 0.02205 | 2.56E-01 |
| caudalmiddlefrontal | AD | rs7356095 | 0.02158 | 0.02232 | 3.34E-01 |
| caudalmiddlefrontal | AD | rs7562708 | 0.01719 | 0.02285 | 4.52E-01 |
| caudalmiddlefrontal | AD | rs9345125 | 0.02191 | 0.02299 | 3.41E-01 |
| caudalmiddlefrontal | AD | rs9402979 | 0.01207 | 0.02148 | 5.74E-01 |
| frontalpole | AD | rs1054442 | 0.00534 | 0.02901 | 8.54E-01 |
| frontalpole | AD | rs113050298 | -0.01214 | 0.03101 | 6.95E-01 |
| frontalpole | AD | rs114620601 | -0.00465 | 0.03224 | 8.85E-01 |
| frontalpole | AD | rs17464221 | -0.02766 | 0.02515 | 2.71E-01 |
| frontalpole | AD | rs403351 | 0.00502 | 0.03189 | 8.75E-01 |
| frontalpole | AD | rs872505 | 0.00704 | 0.02735 | 7.97E-01 |
| lateralorbitofrontal | AD | rs10179285 | 0.04272 | 0.01858 | 2.15E-02 |
| lateralorbitofrontal | AD | rs10916258 | 0.04230 | 0.01856 | 2.27E-02 |
| lateralorbitofrontal | AD | rs11012730 | 0.04300 | 0.01856 | 2.05E-02 |
| lateralorbitofrontal | AD | rs12626790 | 0.04217 | 0.01864 | 2.37E-02 |
| lateralorbitofrontal | AD | rs12652639 | 0.04112 | 0.01822 | 2.40E-02 |
| lateralorbitofrontal | AD | rs13208234 | 0.03358 | 0.01863 | 7.15E-02 |
| lateralorbitofrontal | AD | rs1822951 | 0.04472 | 0.01863 | 1.64E-02 |
| lateralorbitofrontal | AD | rs2237133 | 0.04116 | 0.01817 | 2.35E-02 |
| lateralorbitofrontal | AD | rs2358483 | 0.05142 | 0.01899 | 6.77E-03 |
| lateralorbitofrontal | AD | rs4721802 | 0.04125 | 0.01920 | 3.17E-02 |
| lateralorbitofrontal | AD | rs4897178 | 0.04624 | 0.01836 | 1.18E-02 |
| lateralorbitofrontal | AD | rs56329255 | 0.04324 | 0.01853 | 1.96E-02 |
| lateralorbitofrontal | AD | rs6729276 | 0.03863 | 0.01853 | 3.71E-02 |
| lateralorbitofrontal | AD | rs6737150 | 0.04340 | 0.01864 | 1.99E-02 |
| lateralorbitofrontal | AD | rs67616210 | 0.03885 | 0.01856 | 3.64E-02 |
| lateralorbitofrontal | AD | rs6949868 | 0.04077 | 0.01852 | 2.77E-02 |
| lateralorbitofrontal | AD | rs7252428 | 0.03186 | 0.01869 | 8.83E-02 |
| lateralorbitofrontal | AD | rs7529542 | 0.04199 | 0.01882 | 2.57E-02 |
| lateralorbitofrontal | AD | rs7621856 | 0.04516 | 0.01853 | 1.48E-02 |
| lateralorbitofrontal | AD | rs77692431 | 0.03987 | 0.01864 | 3.24E-02 |
| lateralorbitofrontal | AD | rs79487293 | 0.04057 | 0.01870 | 3.00E-02 |
| medialorbitofrontal | AD | rs117833963 | 0.03767 | 0.04252 | 3.76E-01 |
| medialorbitofrontal | AD | rs2446113 | 0.01032 | 0.04270 | 8.09E-01 |
| medialorbitofrontal | AD | rs2609186 | 0.03088 | 0.04214 | 4.64E-01 |
| medialorbitofrontal | AD | rs7097933 | 0.02743 | 0.04317 | 5.25E-01 |
| medialorbitofrontal | AD | rs9375435 | 0.00853 | 0.04273 | 8.42E-01 |
| paracentral | AD | rs10064431 | -0.02502 | 0.02797 | 3.71E-01 |
| paracentral | AD | rs10850057 | -0.03709 | 0.02590 | 1.52E-01 |
| paracentral | AD | rs12146713 | -0.02670 | 0.02754 | 3.32E-01 |
| paracentral | AD | rs13147448 | -0.03095 | 0.02760 | 2.62E-01 |
| paracentral | AD | rs2269084 | -0.02286 | 0.02781 | 4.11E-01 |
| paracentral | AD | rs2760751 | -0.03076 | 0.02728 | 2.59E-01 |
| paracentral | AD | rs62515458 | -0.00562 | 0.02519 | 8.23E-01 |
| paracentral | AD | rs6582653 | -0.02538 | 0.02547 | 3.19E-01 |
| paracentral | AD | rs9565516 | -0.03492 | 0.02583 | 1.76E-01 |
| parsopercularis | AD | rs11107546 | 0.02206 | 0.01638 | 1.78E-01 |
| parsopercularis | AD | rs1159974 | 0.01453 | 0.01676 | 3.86E-01 |
| parsopercularis | AD | rs12938190 | 0.02303 | 0.01622 | 1.56E-01 |
| parsopercularis | AD | rs2033939 | 0.01075 | 0.01892 | 5.70E-01 |
| parsopercularis | AD | rs2279829 | 0.02695 | 0.01205 | 2.53E-02 |
| parsopercularis | AD | rs441890 | 0.01833 | 0.01724 | 2.88E-01 |
| parsopercularis | AD | rs7550758 | 0.01209 | 0.01471 | 4.11E-01 |
| parsorbitalis | AD | rs10901380 | -0.03145 | 0.02138 | 1.41E-01 |
| parsorbitalis | AD | rs139214174 | -0.03188 | 0.02156 | 1.39E-01 |
| parsorbitalis | AD | rs1503738 | -0.02032 | 0.02170 | 3.49E-01 |
| parsorbitalis | AD | rs2287283 | -0.03088 | 0.02186 | 1.58E-01 |
| parsorbitalis | AD | rs2396373 | -0.03981 | 0.02155 | 6.47E-02 |
| parsorbitalis | AD | rs61901866 | -0.03053 | 0.02218 | 1.69E-01 |
| parsorbitalis | AD | rs7147119 | -0.02880 | 0.02207 | 1.92E-01 |
| parsorbitalis | AD | rs72673107 | -0.02748 | 0.02148 | 2.01E-01 |
| parsorbitalis | AD | rs72691108 | -0.03424 | 0.02207 | 1.21E-01 |
| parsorbitalis | AD | rs9329203 | -0.02565 | 0.02151 | 2.33E-01 |
| parsorbitalis | AD | rs9875836 | -0.03831 | 0.02155 | 7.54E-02 |
| parstriangularis | AD | rs10058365 | -0.00456 | 0.00653 | 4.85E-01 |
| parstriangularis | AD | rs10278627 | -0.00665 | 0.00799 | 4.05E-01 |
| parstriangularis | AD | rs1125867 | -0.00695 | 0.00746 | 3.51E-01 |
| parstriangularis | AD | rs2144366 | -0.00786 | 0.00736 | 2.85E-01 |
| parstriangularis | AD | rs2279829 | -0.00246 | 0.00792 | 7.57E-01 |
| parstriangularis | AD | rs2287283 | -0.00695 | 0.00752 | 3.55E-01 |
| parstriangularis | AD | rs2999980 | -0.00681 | 0.00743 | 3.59E-01 |
| parstriangularis | AD | rs4920605 | -0.00961 | 0.00662 | 1.46E-01 |
| parstriangularis | AD | rs4924345 | -0.01035 | 0.00666 | 1.20E-01 |
| parstriangularis | AD | rs55818129 | -0.00778 | 0.00742 | 2.94E-01 |
| parstriangularis | AD | rs5750482 | -0.00562 | 0.00716 | 4.32E-01 |
| parstriangularis | AD | rs59614433 | -0.00595 | 0.00737 | 4.20E-01 |
| parstriangularis | AD | rs6443469 | -0.00697 | 0.00744 | 3.49E-01 |
| parstriangularis | AD | rs6867851 | -0.00798 | 0.00734 | 2.77E-01 |
| parstriangularis | AD | rs75187227 | -0.00837 | 0.00729 | 2.50E-01 |
| parstriangularis | AD | rs7967462 | -0.00694 | 0.00747 | 3.53E-01 |
| parstriangularis | AD | rs7996803 | -0.00901 | 0.00715 | 2.08E-01 |
| precentral | AD | rs10064431 | -0.01909 | 0.02243 | 3.95E-01 |
| precentral | AD | rs1080066 | 0.01924 | 0.03404 | 5.72E-01 |
| precentral | AD | rs17756000 | -0.01807 | 0.02194 | 4.10E-01 |
| precentral | AD | rs189874581 | -0.01913 | 0.02091 | 3.60E-01 |
| precentral | AD | rs2929680 | -0.01883 | 0.02142 | 3.79E-01 |
| precentral | AD | rs4499967 | -0.01905 | 0.02219 | 3.91E-01 |
| precentral | AD | rs4706392 | -0.02097 | 0.02283 | 3.58E-01 |
| precentral | AD | rs4751614 | -0.02663 | 0.01988 | 1.80E-01 |
| precentral | AD | rs4794859 | -0.02590 | 0.01996 | 1.95E-01 |
| precentral | AD | rs7134627 | -0.02050 | 0.02190 | 3.49E-01 |
| precentral | AD | rs7868648 | -0.01872 | 0.02216 | 3.98E-01 |
| precentral | AD | rs853974 | -0.02814 | 0.01787 | 1.15E-01 |
| rostralanteriorcingulate | AD | rs1178101 | 0.07025 | 0.03213 | 2.88E-02 |
| rostralanteriorcingulate | AD | rs1986012 | 0.05355 | 0.03185 | 9.27E-02 |
| rostralanteriorcingulate | AD | rs2202895 | 0.06366 | 0.03267 | 5.14E-02 |
| rostralanteriorcingulate | AD | rs7874287 | 0.05855 | 0.03205 | 6.77E-02 |
| rostralanteriorcingulate | AD | rs797832 | 0.04148 | 0.03178 | 1.92E-01 |
| rostralanteriorcingulate | AD | rs9905914 | 0.04584 | 0.03185 | 1.50E-01 |
| rostralmiddlefrontal | AD | rs10139160 | -0.00169 | 0.02905 | 9.54E-01 |
| rostralmiddlefrontal | AD | rs10264709 | 0.00196 | 0.02899 | 9.46E-01 |
| rostralmiddlefrontal | AD | rs10283100 | -0.01816 | 0.02634 | 4.90E-01 |
| rostralmiddlefrontal | AD | rs1080066 | -0.01070 | 0.02722 | 6.94E-01 |
| rostralmiddlefrontal | AD | rs1165645 | 0.00043 | 0.02928 | 9.88E-01 |
| rostralmiddlefrontal | AD | rs1257415 | 0.00764 | 0.02808 | 7.86E-01 |
| rostralmiddlefrontal | AD | rs13019832 | -0.00504 | 0.02857 | 8.60E-01 |
| rostralmiddlefrontal | AD | rs1431272 | 0.00263 | 0.02897 | 9.28E-01 |
| rostralmiddlefrontal | AD | rs194242 | 0.00097 | 0.02906 | 9.73E-01 |
| rostralmiddlefrontal | AD | rs2276133 | -0.00002 | 0.02917 | 1.00E+00 |
| rostralmiddlefrontal | AD | rs2279829 | 0.00935 | 0.02727 | 7.32E-01 |
| rostralmiddlefrontal | AD | rs28645132 | -0.00442 | 0.02865 | 8.77E-01 |
| rostralmiddlefrontal | AD | rs35612915 | -0.00686 | 0.02882 | 8.12E-01 |
| rostralmiddlefrontal | AD | rs36006722 | 0.00357 | 0.02894 | 9.02E-01 |
| rostralmiddlefrontal | AD | rs40115 | 0.00015 | 0.02920 | 9.96E-01 |
| rostralmiddlefrontal | AD | rs4670555 | 0.00674 | 0.02810 | 8.10E-01 |
| rostralmiddlefrontal | AD | rs4722006 | -0.00801 | 0.02745 | 7.70E-01 |
| rostralmiddlefrontal | AD | rs6682671 | 0.01120 | 0.02756 | 6.85E-01 |
| rostralmiddlefrontal | AD | rs7529537 | 0.00401 | 0.02898 | 8.90E-01 |
| rostralmiddlefrontal | AD | rs77640487 | 0.00369 | 0.02905 | 8.99E-01 |
| rostralmiddlefrontal | AD | rs9971479 | 0.00270 | 0.02888 | 9.26E-01 |
| superiorfrontal | AD | rs10835817 | -0.05331 | 0.03445 | 1.22E-01 |
| superiorfrontal | AD | rs12989517 | -0.05610 | 0.03443 | 1.03E-01 |
| superiorfrontal | AD | rs140562220 | -0.05736 | 0.03342 | 8.61E-02 |
| superiorfrontal | AD | rs142301939 | -0.05683 | 0.03313 | 8.62E-02 |
| superiorfrontal | AD | rs1473608 | -0.06220 | 0.03445 | 7.10E-02 |
| superiorfrontal | AD | rs17669337 | -0.06944 | 0.03481 | 4.60E-02 |
| superiorfrontal | AD | rs28704635 | -0.04912 | 0.03459 | 1.56E-01 |
| superiorfrontal | AD | rs354085 | -0.05719 | 0.03309 | 8.39E-02 |
| superiorfrontal | AD | rs4842266 | -0.03988 | 0.03589 | 2.66E-01 |
| superiorfrontal | AD | rs4843560 | -0.05435 | 0.03450 | 1.15E-01 |
| superiorfrontal | AD | rs4915928 | -0.05239 | 0.03528 | 1.38E-01 |
| superiorfrontal | AD | rs628380 | -0.03864 | 0.03444 | 2.62E-01 |
| superiorfrontal | AD | rs6912198 | -0.05192 | 0.03328 | 1.19E-01 |
| superiorfrontal | AD | rs76696867 | -0.06507 | 0.03454 | 5.95E-02 |
| banks of the superior temporal sulcus | AD | rs160458 | -0.04273 | 0.02560 | 9.51E-02 |
| banks of the superior temporal sulcus | AD | rs2043294 | -0.03629 | 0.02377 | 1.27E-01 |
| banks of the superior temporal sulcus | AD | rs2573727 | -0.04300 | 0.02458 | 8.03E-02 |
| banks of the superior temporal sulcus | AD | rs62384380 | -0.04254 | 0.02538 | 9.37E-02 |
| banks of the superior temporal sulcus | AD | rs6565627 | -0.04345 | 0.02465 | 7.79E-02 |
| banks of the superior temporal sulcus | AD | rs73006822 | -0.05943 | 0.02236 | 7.85E-03 |
| banks of the superior temporal sulcus | AD | rs7862092 | -0.04185 | 0.02486 | 9.23E-02 |
| banks of the superior temporal sulcus | AD | rs9436222 | -0.02824 | 0.02181 | 1.95E-01 |
| entorhinal | AD | rs12921392 | -0.02760 | 0.02125 | 1.94E-01 |
| entorhinal | AD | rs141912254 | -0.01983 | 0.02040 | 3.31E-01 |
| entorhinal | AD | rs17698176 | -0.02585 | 0.02147 | 2.29E-01 |
| entorhinal | AD | rs2270027 | -0.01664 | 0.01866 | 3.73E-01 |
| entorhinal | AD | rs247787 | -0.03886 | 0.01791 | 3.00E-02 |
| entorhinal | AD | rs35004829 | -0.02379 | 0.02117 | 2.61E-01 |
| entorhinal | AD | rs3743462 | -0.02393 | 0.02031 | 2.39E-01 |
| entorhinal | AD | rs4147321 | -0.03079 | 0.02068 | 1.36E-01 |
| entorhinal | AD | rs4888640 | -0.01890 | 0.01999 | 3.44E-01 |
| entorhinal | AD | rs6693283 | -0.02211 | 0.02098 | 2.92E-01 |
| entorhinal | AD | rs7141150 | -0.03043 | 0.02128 | 1.53E-01 |
| entorhinal | AD | rs73205654 | -0.02507 | 0.02119 | 2.37E-01 |
| fusiform | AD | rs10784446 | -0.00721 | 0.04673 | 8.77E-01 |
| fusiform | AD | rs10940512 | -0.03228 | 0.05112 | 5.28E-01 |
| fusiform | AD | rs17834032 | -0.02997 | 0.05300 | 5.72E-01 |
| fusiform | AD | rs190064082 | -0.01718 | 0.04957 | 7.29E-01 |
| fusiform | AD | rs2074404 | -0.02946 | 0.04374 | 5.01E-01 |
| fusiform | AD | rs61784835 | -0.00259 | 0.04683 | 9.56E-01 |
| fusiform | AD | rs6801733 | -0.00103 | 0.04246 | 9.81E-01 |
| fusiform | AD | rs949279 | -0.04726 | 0.04187 | 2.59E-01 |
| inferiortemporal | AD | rs12351219 | 0.02396 | 0.03138 | 4.45E-01 |
| inferiortemporal | AD | rs2252655 | 0.00088 | 0.02774 | 9.75E-01 |
| inferiortemporal | AD | rs2664129 | 0.01006 | 0.03173 | 7.51E-01 |
| inferiortemporal | AD | rs4646623 | 0.03081 | 0.02812 | 2.73E-01 |
| inferiortemporal | AD | rs4895534 | 0.02424 | 0.03136 | 4.40E-01 |
| inferiortemporal | AD | rs62054449 | 0.02475 | 0.03149 | 4.32E-01 |
| inferiortemporal | AD | rs7155669 | 0.02043 | 0.03236 | 5.28E-01 |
| inferiortemporal | AD | rs71575448 | 0.01838 | 0.03246 | 5.71E-01 |
| inferiortemporal | AD | rs72775393 | 0.00236 | 0.02911 | 9.35E-01 |
| inferiortemporal | AD | rs73006822 | 0.00747 | 0.03180 | 8.14E-01 |
| inferiortemporal | AD | rs9309013 | 0.01472 | 0.03029 | 6.27E-01 |
| insula | AD | rs10123515 | -0.01064 | 0.02454 | 6.65E-01 |
| insula | AD | rs1122688 | 0.00020 | 0.02356 | 9.93E-01 |
| insula | AD | rs11231598 | -0.01368 | 0.02420 | 5.72E-01 |
| insula | AD | rs11901774 | -0.01098 | 0.02467 | 6.56E-01 |
| insula | AD | rs139034643 | -0.01416 | 0.02369 | 5.50E-01 |
| insula | AD | rs4291964 | -0.00325 | 0.02146 | 8.80E-01 |
| insula | AD | rs4706392 | -0.00870 | 0.02473 | 7.25E-01 |
| insula | AD | rs58066679 | -0.00101 | 0.02417 | 9.67E-01 |
| insula | AD | rs6739199 | -0.01797 | 0.02242 | 4.23E-01 |
| insula | AD | rs73166833 | -0.00845 | 0.02461 | 7.31E-01 |
| insula | AD | rs7728751 | -0.00324 | 0.02448 | 8.95E-01 |
| insula | AD | rs78986234 | -0.01498 | 0.02337 | 5.22E-01 |
| insula | AD | rs80043648 | -0.00408 | 0.02390 | 8.64E-01 |
| insula | AD | rs9375452 | -0.00547 | 0.02441 | 8.23E-01 |
| insula | AD | rs976423 | -0.01839 | 0.02242 | 4.12E-01 |
| middletemporal | AD | rs10045552 | 0.03587 | 0.03155 | 2.55E-01 |
| middletemporal | AD | rs10048146 | 0.04089 | 0.02910 | 1.60E-01 |
| middletemporal | AD | rs12794347 | 0.02401 | 0.03178 | 4.50E-01 |
| middletemporal | AD | rs1344762 | 0.02760 | 0.03206 | 3.89E-01 |
| middletemporal | AD | rs141834426 | 0.04690 | 0.02969 | 1.14E-01 |
| middletemporal | AD | rs16971055 | 0.03381 | 0.03212 | 2.93E-01 |
| middletemporal | AD | rs28711421 | 0.00953 | 0.02503 | 7.04E-01 |
| middletemporal | AD | rs621952 | 0.03014 | 0.03215 | 3.48E-01 |
| middletemporal | AD | rs62256903 | 0.03053 | 0.03214 | 3.42E-01 |
| middletemporal | AD | rs72867280 | 0.02871 | 0.03209 | 3.71E-01 |
| middletemporal | AD | rs73006822 | 0.02411 | 0.03235 | 4.56E-01 |
| middletemporal | AD | rs7314572 | 0.03229 | 0.03205 | 3.14E-01 |
| parahippocampal | AD | rs10117940 | 0.03334 | 0.03171 | 2.93E-01 |
| parahippocampal | AD | rs10474080 | 0.03723 | 0.02925 | 2.03E-01 |
| parahippocampal | AD | rs11919722 | 0.01667 | 0.03357 | 6.19E-01 |
| parahippocampal | AD | rs1792354 | 0.02893 | 0.03417 | 3.97E-01 |
| parahippocampal | AD | rs4870489 | 0.02402 | 0.03372 | 4.76E-01 |
| parahippocampal | AD | rs5003492 | -0.00360 | 0.02513 | 8.86E-01 |
| parahippocampal | AD | rs58131984 | 0.02044 | 0.03395 | 5.47E-01 |
| parahippocampal | AD | rs58321169 | 0.02155 | 0.03216 | 5.03E-01 |
| superiortemporal | AD | rs115241741 | -0.04602 | 0.02953 | 1.19E-01 |
| superiortemporal | AD | rs143927182 | -0.03765 | 0.02679 | 1.60E-01 |
| superiortemporal | AD | rs17317075 | -0.04078 | 0.02529 | 1.07E-01 |
| superiortemporal | AD | rs1785181 | -0.05306 | 0.02738 | 5.27E-02 |
| superiortemporal | AD | rs2180127 | -0.04750 | 0.02888 | 1.00E-01 |
| superiortemporal | AD | rs389020 | -0.06402 | 0.02649 | 1.57E-02 |
| superiortemporal | AD | rs4515470 | -0.04521 | 0.02895 | 1.18E-01 |
| superiortemporal | AD | rs4690466 | -0.04646 | 0.02884 | 1.07E-01 |
| superiortemporal | AD | rs4841029 | -0.04206 | 0.03026 | 1.64E-01 |
| superiortemporal | AD | rs7107246 | -0.04665 | 0.02881 | 1.05E-01 |
| superiortemporal | AD | rs7601767 | -0.02859 | 0.02633 | 2.78E-01 |
| superiortemporal | AD | rs7874052 | -0.05000 | 0.02854 | 7.97E-02 |
| temporalpole | AD | rs160472 | -0.06540 | 0.03074 | 3.34E-02 |
| temporalpole | AD | rs2139446 | -0.06311 | 0.03039 | 3.78E-02 |
| temporalpole | AD | rs6855246 | -0.03491 | 0.03377 | 3.01E-01 |
| temporalpole | AD | rs73084102 | -0.05677 | 0.03128 | 6.95E-02 |
| transversetemporal | AD | rs112315969 | 0.01717 | 0.02304 | 4.56E-01 |
| transversetemporal | AD | rs11684511 | 0.01214 | 0.02426 | 6.17E-01 |
| transversetemporal | AD | rs11785060 | 0.00790 | 0.02275 | 7.28E-01 |
| transversetemporal | AD | rs2033939 | 0.00427 | 0.02132 | 8.41E-01 |
| transversetemporal | AD | rs2409691 | 0.00679 | 0.02246 | 7.63E-01 |
| transversetemporal | AD | rs2548226 | 0.01613 | 0.02290 | 4.81E-01 |
| transversetemporal | AD | rs4706391 | 0.01487 | 0.02359 | 5.28E-01 |
| transversetemporal | AD | rs72834200 | 0.02075 | 0.02132 | 3.30E-01 |
| transversetemporal | AD | rs7315185 | 0.00554 | 0.02136 | 7.95E-01 |
| transversetemporal | AD | rs75061235 | 0.00525 | 0.02154 | 8.07E-01 |
| transversetemporal | AD | rs7714191 | 0.01495 | 0.02351 | 5.25E-01 |
| transversetemporal | AD | rs949279 | 0.02073 | 0.02092 | 3.22E-01 |
| transversetemporal | AD | rs9615351 | 0.01577 | 0.02327 | 4.98E-01 |
| transversetemporal | AD | rs9844184 | 0.01462 | 0.02311 | 5.27E-01 |
| inferiorparietal | AD | rs118054914 | 0.01517 | 0.01821 | 4.05E-01 |
| inferiorparietal | AD | rs1413536 | 0.01825 | 0.01934 | 3.45E-01 |
| inferiorparietal | AD | rs148683230 | 0.02080 | 0.01885 | 2.70E-01 |
| inferiorparietal | AD | rs149940542 | 0.01707 | 0.01900 | 3.69E-01 |
| inferiorparietal | AD | rs17019370 | 0.02088 | 0.01892 | 2.70E-01 |
| inferiorparietal | AD | rs2218439 | 0.01021 | 0.01621 | 5.29E-01 |
| inferiorparietal | AD | rs2336714 | 0.03109 | 0.01753 | 7.61E-02 |
| inferiorparietal | AD | rs27540 | 0.02213 | 0.01909 | 2.46E-01 |
| inferiorparietal | AD | rs2779710 | 0.01928 | 0.01900 | 3.10E-01 |
| inferiorparietal | AD | rs34146683 | 0.01350 | 0.01755 | 4.42E-01 |
| inferiorparietal | AD | rs4437022 | 0.02144 | 0.01945 | 2.70E-01 |
| inferiorparietal | AD | rs4889898 | 0.02216 | 0.01859 | 2.33E-01 |
| inferiorparietal | AD | rs62399042 | 0.01581 | 0.01913 | 4.09E-01 |
| inferiorparietal | AD | rs639016 | 0.01661 | 0.01922 | 3.88E-01 |
| inferiorparietal | AD | rs68175985 | 0.01432 | 0.01942 | 4.61E-01 |
| inferiorparietal | AD | rs75911833 | 0.01997 | 0.01898 | 2.93E-01 |
| inferiorparietal | AD | rs7862092 | 0.02191 | 0.01864 | 2.40E-01 |
| inferiorparietal | AD | rs79272390 | 0.02234 | 0.01901 | 2.40E-01 |
| inferiorparietal | AD | rs9856782 | 0.01769 | 0.01847 | 3.38E-01 |
| isthmuscingulate | AD | rs1123680 | 0.04767 | 0.03171 | 1.33E-01 |
| isthmuscingulate | AD | rs12616022 | 0.03298 | 0.02934 | 2.61E-01 |
| isthmuscingulate | AD | rs258035 | 0.02553 | 0.03171 | 4.21E-01 |
| isthmuscingulate | AD | rs3770776 | 0.02213 | 0.03470 | 5.24E-01 |
| isthmuscingulate | AD | rs625695 | 0.02561 | 0.02933 | 3.83E-01 |
| isthmuscingulate | AD | rs78156452 | 0.03748 | 0.03117 | 2.29E-01 |
| isthmuscingulate | AD | rs9392155 | 0.01957 | 0.03209 | 5.42E-01 |
| postcentral | AD | rs11033898 | -0.01405 | 0.01981 | 4.78E-01 |
| postcentral | AD | rs117623407 | -0.01924 | 0.01805 | 2.86E-01 |
| postcentral | AD | rs11789773 | -0.01382 | 0.01980 | 4.85E-01 |
| postcentral | AD | rs1884010 | -0.01291 | 0.01866 | 4.89E-01 |
| postcentral | AD | rs2279829 | -0.03568 | 0.01869 | 5.62E-02 |
| postcentral | AD | rs313135 | -0.01805 | 0.01910 | 3.45E-01 |
| postcentral | AD | rs34322452 | -0.01406 | 0.02016 | 4.86E-01 |
| postcentral | AD | rs344142 | -0.01348 | 0.01959 | 4.92E-01 |
| postcentral | AD | rs4924346 | 0.01425 | 0.02093 | 4.96E-01 |
| postcentral | AD | rs555720 | -0.01245 | 0.01945 | 5.22E-01 |
| postcentral | AD | rs61842496 | -0.01362 | 0.01981 | 4.92E-01 |
| postcentral | AD | rs7856501 | -0.01458 | 0.01964 | 4.58E-01 |
| postcentral | AD | rs881743 | -0.01200 | 0.01938 | 5.36E-01 |
| posteriorcingulate | AD | rs11161942 | 0.00940 | 0.04067 | 8.17E-01 |
| posteriorcingulate | AD | rs11695609 | -0.00764 | 0.03966 | 8.47E-01 |
| posteriorcingulate | AD | rs12764880 | 0.00204 | 0.04037 | 9.60E-01 |
| posteriorcingulate | AD | rs2412771 | 0.02566 | 0.03142 | 4.14E-01 |
| posteriorcingulate | AD | rs66651545 | 0.00126 | 0.03292 | 9.69E-01 |
| posteriorcingulate | AD | rs72761270 | -0.01599 | 0.03608 | 6.58E-01 |
| posteriorcingulate | AD | rs7315284 | 0.00502 | 0.04011 | 9.00E-01 |
| posteriorcingulate | AD | rs77935092 | 0.00846 | 0.04004 | 8.33E-01 |
| posteriorcingulate | AD | rs841860 | 0.01085 | 0.03503 | 7.57E-01 |
| precuneus | AD | rs10008615 | -0.01942 | 0.02152 | 3.67E-01 |
| precuneus | AD | rs10749233 | -0.01938 | 0.02202 | 3.79E-01 |
| precuneus | AD | rs12639074 | -0.02160 | 0.02194 | 3.25E-01 |
| precuneus | AD | rs13060816 | -0.02047 | 0.02179 | 3.48E-01 |
| precuneus | AD | rs13166639 | -0.02454 | 0.02144 | 2.52E-01 |
| precuneus | AD | rs1451294 | -0.02687 | 0.02146 | 2.11E-01 |
| precuneus | AD | rs2022932 | -0.02185 | 0.02200 | 3.20E-01 |
| precuneus | AD | rs2432803 | -0.02797 | 0.02136 | 1.90E-01 |
| precuneus | AD | rs4774220 | -0.02639 | 0.02169 | 2.24E-01 |
| precuneus | AD | rs4811601 | -0.02606 | 0.02188 | 2.34E-01 |
| precuneus | AD | rs56252769 | -0.01553 | 0.02003 | 4.38E-01 |
| precuneus | AD | rs59373415 | -0.01178 | 0.01958 | 5.47E-01 |
| precuneus | AD | rs7111565 | -0.02400 | 0.02205 | 2.76E-01 |
| precuneus | AD | rs73313052 | -0.03559 | 0.02245 | 1.13E-01 |
| precuneus | AD | rs7559976 | -0.02559 | 0.02225 | 2.50E-01 |
| precuneus | AD | rs775718 | -0.02065 | 0.02177 | 3.43E-01 |
| precuneus | AD | rs7782319 | -0.02287 | 0.02165 | 2.91E-01 |
| precuneus | AD | rs888814 | -0.01883 | 0.02159 | 3.83E-01 |
| precuneus | AD | rs905124 | -0.03464 | 0.02003 | 8.38E-02 |
| precuneus | AD | rs9399245 | -0.02104 | 0.02228 | 3.45E-01 |
| superiorparietal | AD | rs10109434 | 0.00361 | 0.01543 | 8.15E-01 |
| superiorparietal | AD | rs114489117 | 0.00301 | 0.01552 | 8.46E-01 |
| superiorparietal | AD | rs115877304 | -0.00131 | 0.01570 | 9.33E-01 |
| superiorparietal | AD | rs142050688 | 0.00216 | 0.01539 | 8.89E-01 |
| superiorparietal | AD | rs17718831 | 0.00249 | 0.01541 | 8.72E-01 |
| superiorparietal | AD | rs1884368 | 0.00293 | 0.01515 | 8.47E-01 |
| superiorparietal | AD | rs2144366 | 0.00419 | 0.01539 | 7.85E-01 |
| superiorparietal | AD | rs40084 | -0.00100 | 0.01535 | 9.48E-01 |
| superiorparietal | AD | rs4924345 | -0.01093 | 0.01634 | 5.03E-01 |
| superiorparietal | AD | rs6022786 | 0.00066 | 0.01546 | 9.66E-01 |
| superiorparietal | AD | rs6059516 | 0.00488 | 0.01538 | 7.51E-01 |
| superiorparietal | AD | rs61872090 | 0.00632 | 0.01540 | 6.81E-01 |
| superiorparietal | AD | rs639016 | 0.00700 | 0.01581 | 6.58E-01 |
| superiorparietal | AD | rs6554054 | 0.00596 | 0.01548 | 7.00E-01 |
| superiorparietal | AD | rs68175985 | 0.01101 | 0.01631 | 4.99E-01 |
| superiorparietal | AD | rs6840242 | 0.00158 | 0.01546 | 9.19E-01 |
| superiorparietal | AD | rs79272390 | 0.00108 | 0.01546 | 9.44E-01 |
| superiorparietal | AD | rs7980991 | 0.00353 | 0.01547 | 8.19E-01 |
| supramarginal | AD | rs1398859 | 0.05377 | 0.01877 | 4.17E-03 |
| supramarginal | AD | rs17011924 | 0.04880 | 0.01997 | 1.45E-02 |
| supramarginal | AD | rs2164950 | 0.04703 | 0.02064 | 2.27E-02 |
| supramarginal | AD | rs2200225 | 0.05764 | 0.01887 | 2.25E-03 |
| supramarginal | AD | rs2279829 | 0.04059 | 0.01958 | 3.82E-02 |
| supramarginal | AD | rs28395235 | 0.04236 | 0.01880 | 2.42E-02 |
| supramarginal | AD | rs28421555 | 0.05327 | 0.01864 | 4.26E-03 |
| supramarginal | AD | rs35378400 | 0.04680 | 0.01981 | 1.82E-02 |
| supramarginal | AD | rs4924345 | 0.04029 | 0.02300 | 7.99E-02 |
| supramarginal | AD | rs61407096 | 0.05339 | 0.01956 | 6.34E-03 |
| supramarginal | AD | rs724972 | 0.05100 | 0.01968 | 9.57E-03 |
| cuneus | AD | rs10765918 | 0.00178 | 0.01915 | 9.26E-01 |
| cuneus | AD | rs112484789 | 0.00838 | 0.01871 | 6.54E-01 |
| cuneus | AD | rs115136616 | -0.00382 | 0.01685 | 8.21E-01 |
| cuneus | AD | rs11652557 | 0.00710 | 0.01909 | 7.10E-01 |
| cuneus | AD | rs12536836 | -0.00004 | 0.01845 | 9.98E-01 |
| cuneus | AD | rs16829649 | 0.01226 | 0.01681 | 4.66E-01 |
| cuneus | AD | rs2155645 | 0.00220 | 0.01857 | 9.06E-01 |
| cuneus | AD | rs4895120 | 0.00985 | 0.01879 | 6.00E-01 |
| cuneus | AD | rs71427711 | -0.00042 | 0.01796 | 9.81E-01 |
| cuneus | AD | rs73092904 | 0.00647 | 0.01881 | 7.31E-01 |
| cuneus | AD | rs73313052 | 0.01174 | 0.01900 | 5.36E-01 |
| cuneus | AD | rs76470478 | 0.00593 | 0.01911 | 7.56E-01 |
| cuneus | AD | rs9834227 | 0.00128 | 0.01855 | 9.45E-01 |
| lateraloccipital | AD | rs10438427 | -0.00677 | 0.02247 | 7.63E-01 |
| lateraloccipital | AD | rs12485668 | -0.02807 | 0.02053 | 1.72E-01 |
| lateraloccipital | AD | rs12609819 | -0.00468 | 0.02300 | 8.39E-01 |
| lateraloccipital | AD | rs16915479 | -0.00492 | 0.02305 | 8.31E-01 |
| lateraloccipital | AD | rs1781030 | -0.00862 | 0.02330 | 7.11E-01 |
| lateraloccipital | AD | rs2074404 | -0.01153 | 0.02118 | 5.86E-01 |
| lateraloccipital | AD | rs2282710 | -0.00446 | 0.02299 | 8.46E-01 |
| lateraloccipital | AD | rs28496034 | 0.00120 | 0.02327 | 9.59E-01 |
| lateraloccipital | AD | rs28514429 | -0.00906 | 0.02275 | 6.90E-01 |
| lateraloccipital | AD | rs4953152 | -0.00999 | 0.02338 | 6.69E-01 |
| lateraloccipital | AD | rs552305 | -0.00887 | 0.02285 | 6.98E-01 |
| lateraloccipital | AD | rs56007616 | -0.00012 | 0.02205 | 9.95E-01 |
| lateraloccipital | AD | rs76341705 | -0.00024 | 0.02398 | 9.92E-01 |
| lateraloccipital | AD | rs8076087 | -0.00972 | 0.02329 | 6.76E-01 |
| lateraloccipital | AD | rs9401907 | -0.01469 | 0.02295 | 5.22E-01 |
| lateraloccipital | AD | rs9863836 | -0.01101 | 0.02344 | 6.38E-01 |
| lingual | AD | rs1014444 | 0.02997 | 0.01344 | 2.58E-02 |
| lingual | AD | rs10237280 | 0.02932 | 0.01360 | 3.10E-02 |
| lingual | AD | rs1078081 | 0.03110 | 0.01331 | 1.94E-02 |
| lingual | AD | rs11929686 | 0.03057 | 0.01350 | 2.36E-02 |
| lingual | AD | rs17419290 | 0.02525 | 0.01331 | 5.79E-02 |
| lingual | AD | rs17690987 | 0.02489 | 0.01339 | 6.32E-02 |
| lingual | AD | rs1905346 | 0.02982 | 0.01339 | 2.60E-02 |
| lingual | AD | rs1934057 | 0.03461 | 0.01337 | 9.65E-03 |
| lingual | AD | rs1952750 | 0.02986 | 0.01317 | 2.34E-02 |
| lingual | AD | rs2022130 | 0.03095 | 0.01352 | 2.21E-02 |
| lingual | AD | rs2043294 | 0.02686 | 0.01327 | 4.29E-02 |
| lingual | AD | rs28410513 | 0.02743 | 0.01331 | 3.94E-02 |
| lingual | AD | rs2999158 | 0.02479 | 0.01343 | 6.49E-02 |
| lingual | AD | rs61508189 | 0.02972 | 0.01321 | 2.45E-02 |
| lingual | AD | rs6603991 | 0.02595 | 0.01331 | 5.13E-02 |
| lingual | AD | rs6812278 | 0.02884 | 0.01353 | 3.30E-02 |
| lingual | AD | rs76341705 | 0.03714 | 0.01366 | 6.54E-03 |
| lingual | AD | rs7809950 | 0.03313 | 0.01335 | 1.31E-02 |
| lingual | AD | rs7914158 | 0.02724 | 0.01330 | 4.05E-02 |
| lingual | AD | rs9401907 | 0.02821 | 0.01353 | 3.71E-02 |
| lingual | AD | rs9545145 | 0.03327 | 0.01330 | 1.24E-02 |
| pericalcarine | AD | rs1046953 | 0.00549 | 0.00918 | 5.49E-01 |
| pericalcarine | AD | rs10765918 | 0.00861 | 0.01019 | 3.98E-01 |
| pericalcarine | AD | rs11103221 | 0.00930 | 0.01004 | 3.55E-01 |
| pericalcarine | AD | rs11252615 | 0.00977 | 0.01009 | 3.33E-01 |
| pericalcarine | AD | rs117892760 | 0.00949 | 0.01022 | 3.53E-01 |
| pericalcarine | AD | rs1223090 | 0.00857 | 0.01018 | 4.00E-01 |
| pericalcarine | AD | rs13115025 | 0.00894 | 0.01019 | 3.80E-01 |
| pericalcarine | AD | rs13726 | 0.00812 | 0.01001 | 4.17E-01 |
| pericalcarine | AD | rs1420791 | 0.00983 | 0.01013 | 3.32E-01 |
| pericalcarine | AD | rs147753572 | 0.00961 | 0.01012 | 3.42E-01 |
| pericalcarine | AD | rs163499 | 0.01000 | 0.01008 | 3.21E-01 |
| pericalcarine | AD | rs16822665 | 0.00931 | 0.01013 | 3.58E-01 |
| pericalcarine | AD | rs16829649 | 0.01171 | 0.00976 | 2.31E-01 |
| pericalcarine | AD | rs17179798 | 0.00900 | 0.01013 | 3.74E-01 |
| pericalcarine | AD | rs28633576 | 0.00796 | 0.01002 | 4.27E-01 |
| pericalcarine | AD | rs2999158 | 0.00654 | 0.00991 | 5.09E-01 |
| pericalcarine | AD | rs35342371 | 0.01121 | 0.00994 | 2.59E-01 |
| pericalcarine | AD | rs4811476 | 0.00948 | 0.01018 | 3.52E-01 |
| pericalcarine | AD | rs4895532 | 0.00984 | 0.01013 | 3.31E-01 |
| pericalcarine | AD | rs56111638 | 0.00856 | 0.01008 | 3.96E-01 |
| pericalcarine | AD | rs57063427 | 0.00616 | 0.00946 | 5.15E-01 |
| pericalcarine | AD | rs57334908 | 0.01161 | 0.00987 | 2.39E-01 |
| pericalcarine | AD | rs62367903 | 0.00569 | 0.00994 | 5.67E-01 |
| pericalcarine | AD | rs6461386 | 0.00799 | 0.01013 | 4.30E-01 |
| pericalcarine | AD | rs6650695 | 0.01005 | 0.01009 | 3.19E-01 |
| pericalcarine | AD | rs667801 | 0.01129 | 0.00980 | 2.49E-01 |
| pericalcarine | AD | rs6741950 | 0.00980 | 0.01009 | 3.32E-01 |
| pericalcarine | AD | rs6812278 | 0.00843 | 0.01020 | 4.08E-01 |
| pericalcarine | AD | rs7188071 | 0.01093 | 0.00995 | 2.72E-01 |
| pericalcarine | AD | rs73313052 | 0.01215 | 0.01027 | 2.37E-01 |
| pericalcarine | AD | rs7364475 | 0.00867 | 0.01008 | 3.90E-01 |
| pericalcarine | AD | rs7378179 | 0.00868 | 0.01012 | 3.91E-01 |
| pericalcarine | AD | rs8034885 | 0.01025 | 0.01012 | 3.11E-01 |
| pericalcarine | AD | rs8103241 | 0.01053 | 0.00999 | 2.92E-01 |
| pericalcarine | AD | rs816328 | 0.00712 | 0.00971 | 4.64E-01 |
| pericalcarine | AD | rs9545158 | 0.01170 | 0.01000 | 2.42E-01 |
| pericalcarine | AD | rs961848 | 0.01080 | 0.00991 | 2.76E-01 |
| pericalcarine | AD | rs971550 | 0.00896 | 0.01015 | 3.77E-01 |
| whole cortex | AD | rs10496091 | 0.00946 | 0.01458 | 5.16E-01 |
| whole cortex | AD | rs10876864 | 0.01044 | 0.01458 | 4.74E-01 |
| whole cortex | AD | rs10878349 | 0.01781 | 0.01394 | 2.01E-01 |
| whole cortex | AD | rs10927043 | 0.01012 | 0.01457 | 4.87E-01 |
| whole cortex | AD | rs11759026 | 0.00411 | 0.01471 | 7.80E-01 |
| whole cortex | AD | rs12357321 | 0.00990 | 0.01462 | 4.98E-01 |
| whole cortex | AD | rs12452834 | 0.00992 | 0.01441 | 4.91E-01 |
| whole cortex | AD | rs12630663 | 0.01074 | 0.01456 | 4.61E-01 |
| whole cortex | AD | rs139849708 | 0.00990 | 0.01466 | 5.00E-01 |
| whole cortex | AD | rs149352678 | 0.01505 | 0.01290 | 2.43E-01 |
| whole cortex | AD | rs1628768 | 0.00746 | 0.01468 | 6.12E-01 |
| whole cortex | AD | rs17543864 | 0.01055 | 0.01453 | 4.68E-01 |
| whole cortex | AD | rs2066827 | 0.01136 | 0.01461 | 4.37E-01 |
| whole cortex | AD | rs2195243 | 0.00930 | 0.01461 | 5.24E-01 |
| whole cortex | AD | rs2301718 | 0.00740 | 0.01440 | 6.07E-01 |
| whole cortex | AD | rs2802295 | 0.00450 | 0.01374 | 7.44E-01 |
| whole cortex | AD | rs3217901 | 0.01077 | 0.01403 | 4.43E-01 |
| whole cortex | AD | rs34464850 | 0.01168 | 0.01475 | 4.28E-01 |
| whole cortex | AD | rs35227403 | 0.00664 | 0.01414 | 6.39E-01 |
| whole cortex | AD | rs386424 | 0.00734 | 0.01435 | 6.09E-01 |
| whole cortex | AD | rs41563 | 0.00919 | 0.01456 | 5.28E-01 |
| whole cortex | AD | rs4846200 | 0.00935 | 0.01442 | 5.17E-01 |
| whole cortex | AD | rs57415181 | 0.00696 | 0.01425 | 6.25E-01 |
| whole cortex | AD | rs6463758 | 0.01401 | 0.01346 | 2.98E-01 |
| whole cortex | AD | rs6572878 | 0.00993 | 0.01440 | 4.90E-01 |
| whole cortex | AD | rs6673449 | 0.01157 | 0.01434 | 4.20E-01 |
| whole cortex | AD | rs7715167 | 0.00961 | 0.01463 | 5.11E-01 |
| whole cortex | AD | rs79600142 | 0.00455 | 0.01532 | 7.66E-01 |
| whole cortex | AD | rs7975351 | 0.00872 | 0.01457 | 5.50E-01 |

**Table S8.** Single SNP analysis of association between genetically predicted cortical surface area and Alzheimer's disease (AD) risk

| **exposure** | **outcome** | **SNP** | **b** | **se** | **p** |
| --- | --- | --- | --- | --- | --- |
| caudalanteriorcingulate | AD | rs10845985 | -0.04606 | 0.06445 | 4.75E-01 |
| caudalanteriorcingulate | AD | rs13021985 | 0.06833 | 0.05813 | 2.40E-01 |
| caudalanteriorcingulate | AD | rs2509765 | 0.02721 | 0.06087 | 6.55E-01 |
| caudalanteriorcingulate | AD | rs2647416 | -0.12446 | 0.06854 | 6.94E-02 |
| caudalanteriorcingulate | AD | rs4747503 | 0.12016 | 0.06023 | 4.60E-02 |
| caudalanteriorcingulate | AD | rs7728751 | 0.05916 | 0.05925 | 3.18E-01 |
| caudalanteriorcingulate | AD | rs80241863 | 0.04972 | 0.04963 | 3.16E-01 |
| caudalmiddlefrontal | AD | rs1261073 | -0.03751 | 0.06824 | 5.83E-01 |
| caudalmiddlefrontal | AD | rs2589230 | 0.05149 | 0.07382 | 4.86E-01 |
| caudalmiddlefrontal | AD | rs30641 | 0.08469 | 0.06017 | 1.59E-01 |
| caudalmiddlefrontal | AD | rs4273712 | 0.09490 | 0.04190 | 2.35E-02 |
| caudalmiddlefrontal | AD | rs448939 | -0.10921 | 0.07404 | 1.40E-01 |
| caudalmiddlefrontal | AD | rs655045 | -0.04131 | 0.06263 | 5.10E-01 |
| caudalmiddlefrontal | AD | rs7184835 | -0.04380 | 0.05897 | 4.58E-01 |
| caudalmiddlefrontal | AD | rs7356095 | -0.03163 | 0.07346 | 6.67E-01 |
| caudalmiddlefrontal | AD | rs7562708 | 0.03013 | 0.06858 | 6.60E-01 |
| caudalmiddlefrontal | AD | rs9345125 | -0.01322 | 0.05704 | 8.17E-01 |
| caudalmiddlefrontal | AD | rs9402979 | 0.10014 | 0.07103 | 1.59E-01 |
| frontalpole | AD | rs1054442 | -0.06639 | 0.06132 | 2.79E-01 |
| frontalpole | AD | rs113050298 | 0.03496 | 0.05489 | 5.24E-01 |
| frontalpole | AD | rs114620601 | -0.00220 | 0.05689 | 9.69E-01 |
| frontalpole | AD | rs17464221 | 0.08729 | 0.04975 | 7.93E-02 |
| frontalpole | AD | rs403351 | -0.03828 | 0.04845 | 4.29E-01 |
| frontalpole | AD | rs872505 | -0.08333 | 0.06345 | 1.89E-01 |
| lateralorbitofrontal | AD | rs10179285 | 0.01829 | 0.08523 | 8.30E-01 |
| lateralorbitofrontal | AD | rs10916258 | 0.02651 | 0.08677 | 7.60E-01 |
| lateralorbitofrontal | AD | rs11012730 | 0.01108 | 0.08690 | 8.99E-01 |
| lateralorbitofrontal | AD | rs12626790 | 0.03150 | 0.07967 | 6.93E-01 |
| lateralorbitofrontal | AD | rs12652639 | 0.11192 | 0.21864 | 6.09E-01 |
| lateralorbitofrontal | AD | rs13208234 | 0.19205 | 0.08062 | 1.72E-02 |
| lateralorbitofrontal | AD | rs1822951 | -0.01698 | 0.08088 | 8.34E-01 |
| lateralorbitofrontal | AD | rs2237133 | 0.28583 | 0.42318 | 4.99E-01 |
| lateralorbitofrontal | AD | rs2358483 | -0.06237 | 0.06182 | 3.13E-01 |
| lateralorbitofrontal | AD | rs4721802 | 0.04469 | 0.05578 | 4.23E-01 |
| lateralorbitofrontal | AD | rs4897178 | -0.16266 | 0.12190 | 1.82E-01 |
| lateralorbitofrontal | AD | rs56329255 | 0.00311 | 0.09011 | 9.72E-01 |
| lateralorbitofrontal | AD | rs6729276 | 0.11198 | 0.09006 | 2.14E-01 |
| lateralorbitofrontal | AD | rs6737150 | 0.00868 | 0.07995 | 9.14E-01 |
| lateralorbitofrontal | AD | rs67616210 | 0.10202 | 0.08675 | 2.40E-01 |
| lateralorbitofrontal | AD | rs6949868 | 0.06230 | 0.09154 | 4.96E-01 |
| lateralorbitofrontal | AD | rs7252428 | 0.20356 | 0.07616 | 7.52E-03 |
| lateralorbitofrontal | AD | rs7529542 | 0.03659 | 0.06900 | 5.96E-01 |
| lateralorbitofrontal | AD | rs7621856 | -0.04231 | 0.09014 | 6.39E-01 |
| lateralorbitofrontal | AD | rs77692431 | 0.07365 | 0.08002 | 3.57E-01 |
| lateralorbitofrontal | AD | rs79487293 | 0.05870 | 0.07582 | 4.39E-01 |
| medialorbitofrontal | AD | rs117833963 | -0.03744 | 0.08638 | 6.65E-01 |
| medialorbitofrontal | AD | rs2446113 | 0.07316 | 0.08486 | 3.89E-01 |
| medialorbitofrontal | AD | rs2609186 | -0.01263 | 0.08975 | 8.88E-01 |
| medialorbitofrontal | AD | rs7097933 | 0.00730 | 0.08147 | 9.29E-01 |
| medialorbitofrontal | AD | rs9375435 | 0.07987 | 0.08465 | 3.45E-01 |
| paracentral | AD | rs10064431 | -0.03614 | 0.06406 | 5.73E-01 |
| paracentral | AD | rs10850057 | 0.03914 | 0.06384 | 5.40E-01 |
| paracentral | AD | rs12146713 | -0.02526 | 0.07250 | 7.28E-01 |
| paracentral | AD | rs13147448 | 0.00181 | 0.06472 | 9.78E-01 |
| paracentral | AD | rs2269084 | -0.04917 | 0.06345 | 4.38E-01 |
| paracentral | AD | rs2760751 | 0.00550 | 0.06963 | 9.37E-01 |
| paracentral | AD | rs62515458 | -0.19306 | 0.07107 | 6.60E-03 |
| paracentral | AD | rs6582653 | -0.21873 | 0.30605 | 4.75E-01 |
| paracentral | AD | rs9565516 | 0.04219 | 0.07207 | 5.58E-01 |
| parsopercularis | AD | rs11107546 | -0.01289 | 0.02864 | 6.53E-01 |
| parsopercularis | AD | rs1159974 | 0.04418 | 0.02457 | 7.22E-02 |
| parsopercularis | AD | rs12938190 | -0.01583 | 0.02672 | 5.54E-01 |
| parsopercularis | AD | rs2033939 | 0.03426 | 0.01572 | 2.93E-02 |
| parsopercularis | AD | rs2279829 | -0.06029 | 0.02921 | 3.90E-02 |
| parsopercularis | AD | rs441890 | 0.02039 | 0.02682 | 4.47E-01 |
| parsopercularis | AD | rs7550758 | 0.07274 | 0.02774 | 8.74E-03 |
| parsorbitalis | AD | rs10901380 | -0.02184 | 0.08325 | 7.93E-01 |
| parsorbitalis | AD | rs139214174 | -0.01872 | 0.07434 | 8.01E-01 |
| parsorbitalis | AD | rs1503738 | -0.13729 | 0.06899 | 4.66E-02 |
| parsorbitalis | AD | rs2287283 | -0.03068 | 0.06441 | 6.34E-01 |
| parsorbitalis | AD | rs2396373 | 0.07637 | 0.07458 | 3.06E-01 |
| parsorbitalis | AD | rs61901866 | -0.03309 | 0.05769 | 5.66E-01 |
| parsorbitalis | AD | rs7147119 | -0.04599 | 0.05983 | 4.42E-01 |
| parsorbitalis | AD | rs72673107 | -0.07496 | 0.07759 | 3.34E-01 |
| parsorbitalis | AD | rs72691108 | -0.00603 | 0.05977 | 9.20E-01 |
| parsorbitalis | AD | rs9329203 | -0.09626 | 0.07627 | 2.07E-01 |
| parsorbitalis | AD | rs9875836 | 0.05887 | 0.07474 | 4.31E-01 |
| parstriangularis | AD | rs10058365 | -0.07831 | 0.02844 | 5.89E-03 |
| parstriangularis | AD | rs10278627 | -0.00977 | 0.01322 | 4.60E-01 |
| parstriangularis | AD | rs1125867 | -0.01206 | 0.02642 | 6.48E-01 |
| parstriangularis | AD | rs2144366 | 0.01447 | 0.03024 | 6.32E-01 |
| parstriangularis | AD | rs2279829 | -0.02312 | 0.01120 | 3.90E-02 |
| parstriangularis | AD | rs2287283 | -0.01088 | 0.02284 | 6.34E-01 |
| parstriangularis | AD | rs2999980 | -0.01685 | 0.02856 | 5.55E-01 |
| parstriangularis | AD | rs4920605 | 0.06146 | 0.02882 | 3.29E-02 |
| parstriangularis | AD | rs4924345 | 0.04731 | 0.02273 | 3.74E-02 |
| parstriangularis | AD | rs55818129 | 0.00791 | 0.02701 | 7.70E-01 |
| parstriangularis | AD | rs5750482 | -0.04746 | 0.02786 | 8.84E-02 |
| parstriangularis | AD | rs59614433 | -0.03004 | 0.02380 | 2.07E-01 |
| parstriangularis | AD | rs6443469 | -0.01252 | 0.02872 | 6.63E-01 |
| parstriangularis | AD | rs6867851 | 0.01700 | 0.02955 | 5.65E-01 |
| parstriangularis | AD | rs75187227 | 0.02306 | 0.02725 | 3.97E-01 |
| parstriangularis | AD | rs7967462 | -0.01222 | 0.02587 | 6.37E-01 |
| parstriangularis | AD | rs7996803 | 0.03074 | 0.02479 | 2.15E-01 |
| precentral | AD | rs10064431 | -0.03946 | 0.06996 | 5.73E-01 |
| precentral | AD | rs1080066 | -0.04076 | 0.01898 | 3.17E-02 |
| precentral | AD | rs17756000 | -0.08641 | 0.08957 | 3.35E-01 |
| precentral | AD | rs189874581 | -0.48779 | 0.34243 | 1.54E-01 |
| precentral | AD | rs2929680 | -0.21853 | 0.19426 | 2.61E-01 |
| precentral | AD | rs4499967 | -0.05268 | 0.08790 | 5.49E-01 |
| precentral | AD | rs4706392 | -0.00970 | 0.05443 | 8.59E-01 |
| precentral | AD | rs4751614 | 0.13504 | 0.07632 | 7.68E-02 |
| precentral | AD | rs4794859 | 0.14441 | 0.08310 | 8.23E-02 |
| precentral | AD | rs7134627 | 0.06105 | 0.21271 | 7.74E-01 |
| precentral | AD | rs7868648 | -0.06048 | 0.08528 | 4.78E-01 |
| precentral | AD | rs853974 | 0.19410 | 0.08063 | 1.61E-02 |
| rostralanteriorcingulate | AD | rs1178101 | -0.01650 | 0.07078 | 8.16E-01 |
| rostralanteriorcingulate | AD | rs1986012 | 0.06557 | 0.07405 | 3.76E-01 |
| rostralanteriorcingulate | AD | rs2202895 | 0.02210 | 0.06574 | 7.37E-01 |
| rostralanteriorcingulate | AD | rs7874287 | 0.03980 | 0.07171 | 5.79E-01 |
| rostralanteriorcingulate | AD | rs797832 | 0.13295 | 0.07494 | 7.61E-02 |
| rostralanteriorcingulate | AD | rs9905914 | 0.10731 | 0.07408 | 1.47E-01 |
| rostralmiddlefrontal | AD | rs10139160 | 0.04087 | 0.10318 | 6.92E-01 |
| rostralmiddlefrontal | AD | rs10264709 | -0.04863 | 0.10694 | 6.49E-01 |
| rostralmiddlefrontal | AD | rs10283100 | 0.18854 | 0.07016 | 7.20E-03 |
| rostralmiddlefrontal | AD | rs1080066 | 0.19626 | 0.09137 | 3.17E-02 |
| rostralmiddlefrontal | AD | rs1165645 | -0.00710 | 0.09187 | 9.38E-01 |
| rostralmiddlefrontal | AD | rs1257415 | -0.15437 | 0.09616 | 1.08E-01 |
| rostralmiddlefrontal | AD | rs13019832 | 0.11675 | 0.10191 | 2.52E-01 |
| rostralmiddlefrontal | AD | rs1431272 | -0.06034 | 0.10269 | 5.57E-01 |
| rostralmiddlefrontal | AD | rs194242 | -0.02311 | 0.10605 | 8.28E-01 |
| rostralmiddlefrontal | AD | rs2276133 | 0.00132 | 0.09861 | 9.89E-01 |
| rostralmiddlefrontal | AD | rs2279829 | -0.20735 | 0.10046 | 3.90E-02 |
| rostralmiddlefrontal | AD | rs28645132 | 0.10695 | 0.10404 | 3.04E-01 |
| rostralmiddlefrontal | AD | rs35612915 | 0.10002 | 0.08194 | 2.22E-01 |
| rostralmiddlefrontal | AD | rs36006722 | -0.07446 | 0.09794 | 4.47E-01 |
| rostralmiddlefrontal | AD | rs40115 | -0.00213 | 0.09670 | 9.82E-01 |
| rostralmiddlefrontal | AD | rs4670555 | -0.15805 | 0.10328 | 1.26E-01 |
| rostralmiddlefrontal | AD | rs4722006 | 0.20705 | 0.10757 | 5.43E-02 |
| rostralmiddlefrontal | AD | rs6682671 | -0.17066 | 0.08408 | 4.24E-02 |
| rostralmiddlefrontal | AD | rs7529537 | -0.07465 | 0.09274 | 4.21E-01 |
| rostralmiddlefrontal | AD | rs77640487 | -0.06706 | 0.09175 | 4.65E-01 |
| rostralmiddlefrontal | AD | rs9971479 | -0.07006 | 0.10907 | 5.21E-01 |
| superiorfrontal | AD | rs10835817 | -0.06990 | 0.11624 | 5.48E-01 |
| superiorfrontal | AD | rs12989517 | -0.03797 | 0.11699 | 7.46E-01 |
| superiorfrontal | AD | rs140562220 | 0.06075 | 0.21826 | 7.81E-01 |
| superiorfrontal | AD | rs142301939 | 0.32874 | 0.43963 | 4.55E-01 |
| superiorfrontal | AD | rs1473608 | 0.03162 | 0.11647 | 7.86E-01 |
| superiorfrontal | AD | rs17669337 | 0.07953 | 0.10485 | 4.48E-01 |
| superiorfrontal | AD | rs28704635 | -0.11190 | 0.11129 | 3.15E-01 |
| superiorfrontal | AD | rs354085 | 0.69478 | 0.56818 | 2.21E-01 |
| superiorfrontal | AD | rs4842266 | -0.13657 | 0.08450 | 1.06E-01 |
| superiorfrontal | AD | rs4843560 | -0.05796 | 0.11461 | 6.13E-01 |
| superiorfrontal | AD | rs4915928 | -0.07072 | 0.09404 | 4.52E-01 |
| superiorfrontal | AD | rs628380 | -0.23804 | 0.11657 | 4.12E-02 |
| superiorfrontal | AD | rs6912198 | -0.23267 | 0.26881 | 3.87E-01 |
| superiorfrontal | AD | rs76696867 | 0.05732 | 0.11321 | 6.13E-01 |
| banks of the superior temporal sulcus | AD | rs160458 | -0.03887 | 0.05049 | 4.41E-01 |
| banks of the superior temporal sulcus | AD | rs2043294 | -0.08801 | 0.06174 | 1.54E-01 |
| banks of the superior temporal sulcus | AD | rs2573727 | -0.03372 | 0.06589 | 6.09E-01 |
| banks of the superior temporal sulcus | AD | rs62384380 | -0.03958 | 0.05295 | 4.55E-01 |
| banks of the superior temporal sulcus | AD | rs6565627 | -0.03052 | 0.06370 | 6.32E-01 |
| banks of the superior temporal sulcus | AD | rs73006822 | 0.05959 | 0.05413 | 2.71E-01 |
| banks of the superior temporal sulcus | AD | rs7862092 | -0.04385 | 0.06050 | 4.69E-01 |
| banks of the superior temporal sulcus | AD | rs9436222 | -0.16357 | 0.06460 | 1.13E-02 |
| entorhinal | AD | rs12921392 | -0.00281 | 0.05601 | 9.60E-01 |
| entorhinal | AD | rs141912254 | -0.08540 | 0.05957 | 1.52E-01 |
| entorhinal | AD | rs17698176 | -0.02018 | 0.05365 | 7.07E-01 |
| entorhinal | AD | rs2270027 | -0.13204 | 0.06272 | 3.53E-02 |
| entorhinal | AD | rs247787 | 0.12545 | 0.05964 | 3.54E-02 |
| entorhinal | AD | rs35004829 | -0.04213 | 0.06031 | 4.85E-01 |
| entorhinal | AD | rs3743462 | -0.09531 | 0.12504 | 4.46E-01 |
| entorhinal | AD | rs4147321 | 0.02602 | 0.05502 | 6.36E-01 |
| entorhinal | AD | rs4888640 | -0.09849 | 0.06064 | 1.04E-01 |
| entorhinal | AD | rs6693283 | -0.05980 | 0.05924 | 3.13E-01 |
| entorhinal | AD | rs7141150 | 0.01066 | 0.04840 | 8.26E-01 |
| entorhinal | AD | rs73205654 | -0.02769 | 0.06167 | 6.53E-01 |
| fusiform | AD | rs10784446 | -0.14265 | 0.08880 | 1.08E-01 |
| fusiform | AD | rs10940512 | 0.03193 | 0.06541 | 6.25E-01 |
| fusiform | AD | rs17834032 | 0.01245 | 0.05936 | 8.34E-01 |
| fusiform | AD | rs190064082 | -0.05280 | 0.09023 | 5.58E-01 |
| fusiform | AD | rs2074404 | 0.24273 | 0.15461 | 1.16E-01 |
| fusiform | AD | rs61784835 | -0.13533 | 0.07580 | 7.42E-02 |
| fusiform | AD | rs6801733 | -0.20722 | 0.09062 | 2.22E-02 |
| fusiform | AD | rs949279 | 0.14401 | 0.07474 | 5.40E-02 |
| inferiortemporal | AD | rs12351219 | -0.05779 | 0.08233 | 4.83E-01 |
| inferiortemporal | AD | rs2252655 | 0.16696 | 0.08364 | 4.59E-02 |
| inferiortemporal | AD | rs2664129 | 0.07638 | 0.08356 | 3.61E-01 |
| inferiortemporal | AD | rs4646623 | -0.12940 | 0.08404 | 1.24E-01 |
| inferiortemporal | AD | rs4895534 | -0.05872 | 0.08149 | 4.71E-01 |
| inferiortemporal | AD | rs62054449 | -0.05585 | 0.07788 | 4.73E-01 |
| inferiortemporal | AD | rs7155669 | -0.02033 | 0.07865 | 7.96E-01 |
| inferiortemporal | AD | rs71575448 | -0.00396 | 0.08092 | 9.61E-01 |
| inferiortemporal | AD | rs72775393 | 0.14110 | 0.08044 | 7.94E-02 |
| inferiortemporal | AD | rs73006822 | 0.08141 | 0.07394 | 2.71E-01 |
| inferiortemporal | AD | rs9309013 | 0.27764 | 0.34068 | 4.15E-01 |
| insula | AD | rs10123515 | 0.01547 | 0.08033 | 8.47E-01 |
| insula | AD | rs1122688 | -0.09978 | 0.06570 | 1.29E-01 |
| insula | AD | rs11231598 | 0.05445 | 0.07642 | 4.76E-01 |
| insula | AD | rs11901774 | 0.01595 | 0.07416 | 8.30E-01 |
| insula | AD | rs139034643 | 0.08370 | 0.08718 | 3.37E-01 |
| insula | AD | rs4291964 | -0.27345 | 0.13644 | 4.51E-02 |
| insula | AD | rs4706392 | -0.01337 | 0.07501 | 8.59E-01 |
| insula | AD | rs58066679 | -0.07976 | 0.06255 | 2.02E-01 |
| insula | AD | rs6739199 | 0.13017 | 0.08126 | 1.09E-01 |
| insula | AD | rs73166833 | -0.01775 | 0.07995 | 8.24E-01 |
| insula | AD | rs7728751 | -0.06524 | 0.06535 | 3.18E-01 |
| insula | AD | rs78986234 | 0.10043 | 0.08793 | 2.53E-01 |
| insula | AD | rs80043648 | -0.08882 | 0.08261 | 2.82E-01 |
| insula | AD | rs9375452 | -0.05754 | 0.07636 | 4.51E-01 |
| insula | AD | rs976423 | 0.12702 | 0.07869 | 1.06E-01 |
| middletemporal | AD | rs10045552 | -0.03741 | 0.08761 | 6.69E-01 |
| middletemporal | AD | rs10048146 | -0.12542 | 0.09566 | 1.90E-01 |
| middletemporal | AD | rs12794347 | 0.09298 | 0.07970 | 2.43E-01 |
| middletemporal | AD | rs1344762 | 0.06277 | 0.08668 | 4.69E-01 |
| middletemporal | AD | rs141834426 | -0.08311 | 0.06753 | 2.18E-01 |
| middletemporal | AD | rs16971055 | -0.00731 | 0.08283 | 9.30E-01 |
| middletemporal | AD | rs28711421 | 0.29680 | 0.08962 | 9.26E-04 |
| middletemporal | AD | rs621952 | 0.03256 | 0.08875 | 7.14E-01 |
| middletemporal | AD | rs62256903 | 0.02763 | 0.08917 | 7.57E-01 |
| middletemporal | AD | rs72867280 | 0.05062 | 0.08880 | 5.69E-01 |
| middletemporal | AD | rs73006822 | 0.07872 | 0.07150 | 2.71E-01 |
| middletemporal | AD | rs7314572 | 0.00523 | 0.08932 | 9.53E-01 |
| parahippocampal | AD | rs10117940 | -0.04912 | 0.06379 | 4.41E-01 |
| parahippocampal | AD | rs10474080 | -0.08030 | 0.06548 | 2.20E-01 |
| parahippocampal | AD | rs11919722 | 0.05701 | 0.06237 | 3.61E-01 |
| parahippocampal | AD | rs1792354 | -0.00962 | 0.05631 | 8.64E-01 |
| parahippocampal | AD | rs4870489 | 0.01005 | 0.06682 | 8.80E-01 |
| parahippocampal | AD | rs5003492 | 0.18344 | 0.06266 | 3.42E-03 |
| parahippocampal | AD | rs58131984 | 0.03444 | 0.06375 | 5.89E-01 |
| parahippocampal | AD | rs58321169 | 0.04428 | 0.12703 | 7.27E-01 |
| superiortemporal | AD | rs115241741 | -0.04254 | 0.07493 | 5.70E-01 |
| superiortemporal | AD | rs143927182 | -0.17204 | 0.10195 | 9.15E-02 |
| superiortemporal | AD | rs17317075 | -0.34708 | 0.19732 | 7.86E-02 |
| superiortemporal | AD | rs1785181 | 0.05750 | 0.09597 | 5.49E-01 |
| superiortemporal | AD | rs2180127 | -0.02264 | 0.09093 | 8.03E-01 |
| superiortemporal | AD | rs389020 | 0.08973 | 0.07190 | 2.12E-01 |
| superiortemporal | AD | rs4515470 | -0.05096 | 0.09130 | 5.77E-01 |
| superiortemporal | AD | rs4690466 | -0.03446 | 0.09512 | 7.17E-01 |
| superiortemporal | AD | rs4841029 | -0.06447 | 0.06223 | 3.00E-01 |
| superiortemporal | AD | rs7107246 | -0.03159 | 0.09609 | 7.42E-01 |
| superiortemporal | AD | rs7601767 | -0.18559 | 0.07544 | 1.39E-02 |
| superiortemporal | AD | rs7874052 | 0.00772 | 0.09042 | 9.32E-01 |
| temporalpole | AD | rs160472 | -0.02146 | 0.05886 | 7.15E-01 |
| temporalpole | AD | rs2139446 | -0.02671 | 0.06159 | 6.65E-01 |
| temporalpole | AD | rs6855246 | -0.09532 | 0.04614 | 3.88E-02 |
| temporalpole | AD | rs73084102 | -0.05353 | 0.05550 | 3.35E-01 |
| transversetemporal | AD | rs112315969 | -0.04152 | 0.05616 | 4.60E-01 |
| transversetemporal | AD | rs11684511 | 0.01578 | 0.04277 | 7.12E-01 |
| transversetemporal | AD | rs11785060 | 0.07976 | 0.06100 | 1.91E-01 |
| transversetemporal | AD | rs2033939 | 0.13463 | 0.06177 | 2.93E-02 |
| transversetemporal | AD | rs2409691 | 0.09375 | 0.06036 | 1.20E-01 |
| transversetemporal | AD | rs2548226 | -0.04395 | 0.06474 | 4.97E-01 |
| transversetemporal | AD | rs4706391 | -0.00985 | 0.05185 | 8.49E-01 |
| transversetemporal | AD | rs72834200 | -0.11069 | 0.06287 | 7.83E-02 |
| transversetemporal | AD | rs7315185 | 0.14380 | 0.06908 | 3.74E-02 |
| transversetemporal | AD | rs75061235 | 0.13170 | 0.06475 | 4.19E-02 |
| transversetemporal | AD | rs7714191 | -0.01223 | 0.05347 | 8.19E-01 |
| transversetemporal | AD | rs949279 | -0.12981 | 0.06737 | 5.40E-02 |
| transversetemporal | AD | rs9615351 | -0.02538 | 0.05652 | 6.53E-01 |
| transversetemporal | AD | rs9844184 | -0.02141 | 0.06640 | 7.47E-01 |
| inferiorparietal | AD | rs118054914 | 0.14132 | 0.09197 | 1.24E-01 |
| inferiorparietal | AD | rs1413536 | 0.02838 | 0.06084 | 6.41E-01 |
| inferiorparietal | AD | rs148683230 | -0.03318 | 0.08568 | 6.99E-01 |
| inferiorparietal | AD | rs149940542 | 0.05836 | 0.07464 | 4.34E-01 |
| inferiorparietal | AD | rs17019370 | -0.02789 | 0.07978 | 7.27E-01 |
| inferiorparietal | AD | rs2218439 | 0.23769 | 0.08113 | 3.39E-03 |
| inferiorparietal | AD | rs2336714 | -0.08459 | 0.04906 | 8.47E-02 |
| inferiorparietal | AD | rs27540 | -0.02650 | 0.06214 | 6.70E-01 |
| inferiorparietal | AD | rs2779710 | 0.00933 | 0.08596 | 9.14E-01 |
| inferiorparietal | AD | rs34146683 | 0.17885 | 0.08759 | 4.12E-02 |
| inferiorparietal | AD | rs4437022 | -0.00717 | 0.05381 | 8.94E-01 |
| inferiorparietal | AD | rs4889898 | -0.06573 | 0.08315 | 4.29E-01 |
| inferiorparietal | AD | rs62399042 | 0.06169 | 0.06081 | 3.10E-01 |
| inferiorparietal | AD | rs639016 | 0.05108 | 0.06114 | 4.03E-01 |
| inferiorparietal | AD | rs68175985 | 0.05910 | 0.04950 | 2.32E-01 |
| inferiorparietal | AD | rs75911833 | -0.00830 | 0.08342 | 9.21E-01 |
| inferiorparietal | AD | rs7862092 | -0.06080 | 0.08389 | 4.69E-01 |
| inferiorparietal | AD | rs79272390 | -0.03192 | 0.06356 | 6.15E-01 |
| inferiorparietal | AD | rs9856782 | 0.16622 | 0.17295 | 3.37E-01 |
| isthmuscingulate | AD | rs1123680 | -0.05735 | 0.07137 | 4.22E-01 |
| isthmuscingulate | AD | rs12616022 | -0.07422 | 0.18505 | 6.88E-01 |
| isthmuscingulate | AD | rs258035 | 0.05482 | 0.07143 | 4.43E-01 |
| isthmuscingulate | AD | rs3770776 | 0.04932 | 0.05271 | 3.49E-01 |
| isthmuscingulate | AD | rs625695 | 0.22388 | 0.18734 | 2.32E-01 |
| isthmuscingulate | AD | rs78156452 | -0.01518 | 0.07876 | 8.47E-01 |
| isthmuscingulate | AD | rs9392155 | 0.07807 | 0.06750 | 2.47E-01 |
| postcentral | AD | rs11033898 | -0.01846 | 0.07509 | 8.06E-01 |
| postcentral | AD | rs117623407 | 0.11597 | 0.08819 | 1.89E-01 |
| postcentral | AD | rs11789773 | -0.02311 | 0.07573 | 7.60E-01 |
| postcentral | AD | rs1884010 | -0.23124 | 0.21381 | 2.79E-01 |
| postcentral | AD | rs2279829 | 0.08167 | 0.03957 | 3.90E-02 |
| postcentral | AD | rs313135 | 0.05994 | 0.07686 | 4.36E-01 |
| postcentral | AD | rs34322452 | -0.01673 | 0.05855 | 7.75E-01 |
| postcentral | AD | rs344142 | -0.03755 | 0.09264 | 6.85E-01 |
| postcentral | AD | rs4924346 | -0.06772 | 0.02865 | 1.81E-02 |
| postcentral | AD | rs555720 | -0.06343 | 0.08914 | 4.77E-01 |
| postcentral | AD | rs61842496 | -0.02627 | 0.07393 | 7.22E-01 |
| postcentral | AD | rs7856501 | -0.00564 | 0.09139 | 9.51E-01 |
| postcentral | AD | rs881743 | -0.07072 | 0.08577 | 4.10E-01 |
| posteriorcingulate | AD | rs11161942 | -0.02179 | 0.06331 | 7.31E-01 |
| posteriorcingulate | AD | rs11695609 | 0.06518 | 0.06097 | 2.85E-01 |
| posteriorcingulate | AD | rs12764880 | 0.02064 | 0.06783 | 7.61E-01 |
| posteriorcingulate | AD | rs2412771 | -0.17227 | 0.07642 | 2.42E-02 |
| posteriorcingulate | AD | rs66651545 | 0.71869 | 0.36823 | 5.10E-02 |
| posteriorcingulate | AD | rs72761270 | 0.11353 | 0.06264 | 6.99E-02 |
| posteriorcingulate | AD | rs7315284 | 0.00118 | 0.07192 | 9.87E-01 |
| posteriorcingulate | AD | rs77935092 | -0.02242 | 0.07015 | 7.49E-01 |
| posteriorcingulate | AD | rs841860 | -0.23917 | 0.15727 | 1.28E-01 |
| precuneus | AD | rs10008615 | -0.11998 | 0.09646 | 2.14E-01 |
| precuneus | AD | rs10749233 | -0.07947 | 0.07417 | 2.84E-01 |
| precuneus | AD | rs12639074 | -0.06207 | 0.09461 | 5.12E-01 |
| precuneus | AD | rs13060816 | -0.08865 | 0.09359 | 3.44E-01 |
| precuneus | AD | rs13166639 | 0.11657 | 0.18957 | 5.39E-01 |
| precuneus | AD | rs1451294 | 0.08063 | 0.10058 | 4.23E-01 |
| precuneus | AD | rs2022932 | -0.05358 | 0.09135 | 5.58E-01 |
| precuneus | AD | rs2432803 | 0.08576 | 0.09077 | 3.45E-01 |
| precuneus | AD | rs4774220 | 0.05500 | 0.09382 | 5.58E-01 |
| precuneus | AD | rs4811601 | 0.03586 | 0.08627 | 6.78E-01 |
| precuneus | AD | rs56252769 | -0.21203 | 0.09449 | 2.48E-02 |
| precuneus | AD | rs59373415 | -0.19178 | 0.07410 | 9.65E-03 |
| precuneus | AD | rs7111565 | -0.00488 | 0.08947 | 9.56E-01 |
| precuneus | AD | rs73313052 | 0.04065 | 0.04620 | 3.79E-01 |
| precuneus | AD | rs7559976 | 0.01008 | 0.07167 | 8.88E-01 |
| precuneus | AD | rs775718 | -0.08993 | 0.09775 | 3.58E-01 |
| precuneus | AD | rs7782319 | -0.06613 | 0.22177 | 7.66E-01 |
| precuneus | AD | rs888814 | -0.11213 | 0.08637 | 1.94E-01 |
| precuneus | AD | rs905124 | 0.12399 | 0.06934 | 7.38E-02 |
| precuneus | AD | rs9399245 | -0.05207 | 0.07115 | 4.64E-01 |
| superiorparietal | AD | rs10109434 | -0.01963 | 0.07420 | 7.91E-01 |
| superiorparietal | AD | rs114489117 | -0.00380 | 0.06532 | 9.54E-01 |
| superiorparietal | AD | rs115877304 | 0.05219 | 0.05551 | 3.47E-01 |
| superiorparietal | AD | rs142050688 | 0.01531 | 0.07810 | 8.45E-01 |
| superiorparietal | AD | rs17718831 | 0.00653 | 0.07596 | 9.31E-01 |
| superiorparietal | AD | rs1884368 | -0.04015 | 0.18811 | 8.31E-01 |
| superiorparietal | AD | rs2144366 | -0.03761 | 0.07858 | 6.32E-01 |
| superiorparietal | AD | rs40084 | 0.11486 | 0.08510 | 1.77E-01 |
| superiorparietal | AD | rs4924345 | 0.08242 | 0.03960 | 3.74E-02 |
| superiorparietal | AD | rs6022786 | 0.04471 | 0.07110 | 5.29E-01 |
| superiorparietal | AD | rs6059516 | -0.05862 | 0.08060 | 4.67E-01 |
| superiorparietal | AD | rs61872090 | -0.09146 | 0.07791 | 2.40E-01 |
| superiorparietal | AD | rs639016 | -0.04263 | 0.05103 | 4.03E-01 |
| superiorparietal | AD | rs6554054 | -0.06269 | 0.06876 | 3.62E-01 |
| superiorparietal | AD | rs68175985 | -0.04784 | 0.04007 | 2.32E-01 |
| superiorparietal | AD | rs6840242 | 0.02503 | 0.07067 | 7.23E-01 |
| superiorparietal | AD | rs79272390 | 0.03561 | 0.07090 | 6.15E-01 |
| superiorparietal | AD | rs7980991 | -0.01530 | 0.06981 | 8.27E-01 |
| supramarginal | AD | rs1398859 | -0.03756 | 0.07909 | 6.35E-01 |
| supramarginal | AD | rs17011924 | 0.05129 | 0.08479 | 5.45E-01 |
| supramarginal | AD | rs2164950 | 0.06351 | 0.05398 | 2.39E-01 |
| supramarginal | AD | rs2200225 | -0.07716 | 0.07170 | 2.82E-01 |
| supramarginal | AD | rs2279829 | 0.10380 | 0.05029 | 3.90E-02 |
| supramarginal | AD | rs28395235 | 0.15517 | 0.07573 | 4.05E-02 |
| supramarginal | AD | rs28421555 | -0.05176 | 0.08953 | 5.63E-01 |
| supramarginal | AD | rs35378400 | 0.08621 | 0.07883 | 2.74E-01 |
| supramarginal | AD | rs4924345 | 0.06765 | 0.03250 | 3.74E-02 |
| supramarginal | AD | rs61407096 | -0.00520 | 0.06601 | 9.37E-01 |
| supramarginal | AD | rs724972 | 0.00460 | 0.08598 | 9.57E-01 |
| cuneus | AD | rs10765918 | 0.03238 | 0.05225 | 5.36E-01 |
| cuneus | AD | rs112484789 | -0.03992 | 0.05976 | 5.04E-01 |
| cuneus | AD | rs115136616 | 0.14107 | 0.06709 | 3.55E-02 |
| cuneus | AD | rs11652557 | -0.02016 | 0.05597 | 7.19E-01 |
| cuneus | AD | rs12536836 | 0.06154 | 0.05845 | 2.92E-01 |
| cuneus | AD | rs16829649 | -0.12304 | 0.06943 | 7.64E-02 |
| cuneus | AD | rs2155645 | 0.05263 | 0.07235 | 4.67E-01 |
| cuneus | AD | rs4895120 | -0.04055 | 0.05147 | 4.31E-01 |
| cuneus | AD | rs71427711 | 0.08184 | 0.06504 | 2.08E-01 |
| cuneus | AD | rs73092904 | -0.02384 | 0.06903 | 7.30E-01 |
| cuneus | AD | rs73313052 | -0.03866 | 0.04393 | 3.79E-01 |
| cuneus | AD | rs76470478 | -0.00920 | 0.05898 | 8.76E-01 |
| cuneus | AD | rs9834227 | 0.05627 | 0.06482 | 3.85E-01 |
| lateraloccipital | AD | rs10438427 | -0.14978 | 0.19056 | 4.32E-01 |
| lateraloccipital | AD | rs12485668 | 0.16621 | 0.06093 | 6.37E-03 |
| lateraloccipital | AD | rs12609819 | -0.06856 | 0.08220 | 4.04E-01 |
| lateraloccipital | AD | rs16915479 | -0.06447 | 0.08221 | 4.33E-01 |
| lateraloccipital | AD | rs1781030 | -0.00168 | 0.08554 | 9.84E-01 |
| lateraloccipital | AD | rs2074404 | 0.29611 | 0.18861 | 1.16E-01 |
| lateraloccipital | AD | rs2282710 | -0.07064 | 0.08118 | 3.84E-01 |
| lateraloccipital | AD | rs28496034 | -0.07228 | 0.05423 | 1.83E-01 |
| lateraloccipital | AD | rs28514429 | 0.05730 | 0.17659 | 7.46E-01 |
| lateraloccipital | AD | rs4953152 | 0.01707 | 0.07691 | 8.24E-01 |
| lateraloccipital | AD | rs552305 | 0.02717 | 0.14895 | 8.55E-01 |
| lateraloccipital | AD | rs56007616 | -0.11635 | 0.07354 | 1.14E-01 |
| lateraloccipital | AD | rs76341705 | -0.05376 | 0.05026 | 2.85E-01 |
| lateraloccipital | AD | rs8076087 | 0.01667 | 0.08265 | 8.40E-01 |
| lateraloccipital | AD | rs9401907 | 0.06193 | 0.06717 | 3.57E-01 |
| lateraloccipital | AD | rs9863836 | 0.02582 | 0.07113 | 7.17E-01 |
| lingual | AD | rs1014444 | 0.01781 | 0.06368 | 7.80E-01 |
| lingual | AD | rs10237280 | 0.03149 | 0.05231 | 5.47E-01 |
| lingual | AD | rs1078081 | -0.00983 | 0.06493 | 8.80E-01 |
| lingual | AD | rs11929686 | 0.01040 | 0.05552 | 8.51E-01 |
| lingual | AD | rs17419290 | 0.11931 | 0.06150 | 5.24E-02 |
| lingual | AD | rs17690987 | 0.10560 | 0.05468 | 5.34E-02 |
| lingual | AD | rs1905346 | 0.01936 | 0.07019 | 7.83E-01 |
| lingual | AD | rs1934057 | -0.06225 | 0.05644 | 2.70E-01 |
| lingual | AD | rs1952750 | -0.02453 | 0.15129 | 8.71E-01 |
| lingual | AD | rs2022130 | 0.00653 | 0.05258 | 9.01E-01 |
| lingual | AD | rs2043294 | 0.09491 | 0.06658 | 1.54E-01 |
| lingual | AD | rs28410513 | 0.07228 | 0.06114 | 2.37E-01 |
| lingual | AD | rs2999158 | 0.10018 | 0.05229 | 5.54E-02 |
| lingual | AD | rs61508189 | 0.00095 | 0.13553 | 9.94E-01 |
| lingual | AD | rs6603991 | 0.10345 | 0.06114 | 9.06E-02 |
| lingual | AD | rs6812278 | 0.04023 | 0.05596 | 4.72E-01 |
| lingual | AD | rs76341705 | -0.04570 | 0.04272 | 2.85E-01 |
| lingual | AD | rs7809950 | -0.03910 | 0.05767 | 4.98E-01 |
| lingual | AD | rs7914158 | 0.07928 | 0.06307 | 2.09E-01 |
| lingual | AD | rs9401907 | 0.04891 | 0.05305 | 3.57E-01 |
| lingual | AD | rs9545145 | -0.05401 | 0.06229 | 3.86E-01 |
| pericalcarine | AD | rs1046953 | 0.18092 | 0.05592 | 1.21E-03 |
| pericalcarine | AD | rs10765918 | 0.02676 | 0.04320 | 5.36E-01 |
| pericalcarine | AD | rs11103221 | 0.00187 | 0.13110 | 9.89E-01 |
| pericalcarine | AD | rs11252615 | -0.01943 | 0.06270 | 7.57E-01 |
| pericalcarine | AD | rs117892760 | 0.00379 | 0.04157 | 9.27E-01 |
| pericalcarine | AD | rs1223090 | 0.02851 | 0.04400 | 5.17E-01 |
| pericalcarine | AD | rs13115025 | 0.01884 | 0.04491 | 6.75E-01 |
| pericalcarine | AD | rs13726 | 0.07073 | 0.06066 | 2.44E-01 |
| pericalcarine | AD | rs1420791 | -0.01272 | 0.05191 | 8.06E-01 |
| pericalcarine | AD | rs147753572 | -0.00735 | 0.05747 | 8.98E-01 |
| pericalcarine | AD | rs163499 | -0.02688 | 0.05812 | 6.44E-01 |
| pericalcarine | AD | rs16822665 | 0.00727 | 0.05652 | 8.98E-01 |
| pericalcarine | AD | rs16829649 | -0.09696 | 0.05471 | 7.64E-02 |
| pericalcarine | AD | rs17179798 | 0.02117 | 0.05531 | 7.02E-01 |
| pericalcarine | AD | rs28633576 | 0.06960 | 0.05643 | 2.17E-01 |
| pericalcarine | AD | rs2999158 | 0.08350 | 0.04358 | 5.54E-02 |
| pericalcarine | AD | rs35342371 | -0.06614 | 0.05169 | 2.01E-01 |
| pericalcarine | AD | rs4811476 | 0.00285 | 0.04623 | 9.51E-01 |
| pericalcarine | AD | rs4895532 | -0.01265 | 0.05122 | 8.05E-01 |
| pericalcarine | AD | rs56111638 | 0.04589 | 0.05946 | 4.40E-01 |
| pericalcarine | AD | rs57063427 | 0.15695 | 0.05711 | 5.99E-03 |
| pericalcarine | AD | rs57334908 | -0.07746 | 0.05057 | 1.26E-01 |
| pericalcarine | AD | rs62367903 | 0.07591 | 0.03635 | 3.68E-02 |
| pericalcarine | AD | rs6461386 | 0.04565 | 0.04460 | 3.06E-01 |
| pericalcarine | AD | rs6650695 | -0.02629 | 0.05601 | 6.39E-01 |
| pericalcarine | AD | rs667801 | -0.09894 | 0.06049 | 1.02E-01 |
| pericalcarine | AD | rs6741950 | -0.01978 | 0.06132 | 7.47E-01 |
| pericalcarine | AD | rs6812278 | 0.02999 | 0.04171 | 4.72E-01 |
| pericalcarine | AD | rs7188071 | -0.06638 | 0.05584 | 2.35E-01 |
| pericalcarine | AD | rs73313052 | -0.02639 | 0.02998 | 3.79E-01 |
| pericalcarine | AD | rs7364475 | 0.04298 | 0.06224 | 4.90E-01 |
| pericalcarine | AD | rs7378179 | 0.03452 | 0.05426 | 5.25E-01 |
| pericalcarine | AD | rs8034885 | -0.02513 | 0.04912 | 6.09E-01 |
| pericalcarine | AD | rs8103241 | -0.05931 | 0.06087 | 3.30E-01 |
| pericalcarine | AD | rs816328 | 0.13671 | 0.06374 | 3.20E-02 |
| pericalcarine | AD | rs9545158 | -0.05217 | 0.04203 | 2.15E-01 |
| pericalcarine | AD | rs961848 | -0.08064 | 0.06334 | 2.03E-01 |
| pericalcarine | AD | rs971550 | 0.02080 | 0.05122 | 6.85E-01 |
| whole cortex | AD | rs10496091 | 0.01583 | 0.06183 | 7.98E-01 |
| whole cortex | AD | rs10876864 | -0.01814 | 0.05738 | 7.52E-01 |
| whole cortex | AD | rs10878349 | -0.08461 | 0.03440 | 1.39E-02 |
| whole cortex | AD | rs10927043 | -0.01075 | 0.06273 | 8.64E-01 |
| whole cortex | AD | rs11759026 | 0.06365 | 0.03200 | 4.67E-02 |
| whole cortex | AD | rs12357321 | 0.00047 | 0.05592 | 9.93E-01 |
| whole cortex | AD | rs12452834 | -0.05724 | 0.14526 | 6.94E-01 |
| whole cortex | AD | rs12630663 | -0.02876 | 0.05770 | 6.18E-01 |
| whole cortex | AD | rs139849708 | 0.00193 | 0.05194 | 9.70E-01 |
| whole cortex | AD | rs149352678 | -0.21408 | 0.06317 | 7.02E-04 |
| whole cortex | AD | rs1628768 | 0.04885 | 0.04256 | 2.51E-01 |
| whole cortex | AD | rs17543864 | -0.02818 | 0.06290 | 6.54E-01 |
| whole cortex | AD | rs2066827 | -0.03125 | 0.04813 | 5.16E-01 |
| whole cortex | AD | rs2195243 | 0.02014 | 0.05700 | 7.24E-01 |
| whole cortex | AD | rs2301718 | 0.08460 | 0.05734 | 1.40E-01 |
| whole cortex | AD | rs2802295 | 0.14454 | 0.05087 | 4.49E-03 |
| whole cortex | AD | rs3217901 | -0.25871 | 0.14920 | 8.29E-02 |
| whole cortex | AD | rs34464850 | -0.02395 | 0.04044 | 5.54E-01 |
| whole cortex | AD | rs35227403 | 0.12367 | 0.06096 | 4.25E-02 |
| whole cortex | AD | rs386424 | 0.09300 | 0.05968 | 1.19E-01 |
| whole cortex | AD | rs41563 | 0.02799 | 0.06422 | 6.63E-01 |
| whole cortex | AD | rs4846200 | 0.06538 | 0.13987 | 6.40E-01 |
| whole cortex | AD | rs57415181 | 0.10740 | 0.05980 | 7.25E-02 |
| whole cortex | AD | rs6463758 | -0.17704 | 0.06416 | 5.79E-03 |
| whole cortex | AD | rs6572878 | -0.06911 | 0.15472 | 6.55E-01 |
| whole cortex | AD | rs6673449 | -0.07799 | 0.06594 | 2.37E-01 |
| whole cortex | AD | rs7715167 | 0.00988 | 0.05497 | 8.57E-01 |
| whole cortex | AD | rs79600142 | 0.04058 | 0.02595 | 1.18E-01 |
| whole cortex | AD | rs7975351 | 0.04184 | 0.05890 | 4.78E-01 |

**Table S9.** Heterogeneity analysis of association between genetically predicted cortical surface area and Alzheimer's disease (AD) risk

| **exposure** | **outcome** | **method** | **Q** | **Q_df** | **Q_pval** |
| --- | --- | --- | --- | --- | --- |
| caudal anterior cingulate | AD | MR Egger | 8.61 | 5 | 1.26E-01 |
|  |  | Inverse variance weighted | 9.55 | 6 | 1.45E-01 |
| caudal middle frontal | AD | MR Egger | 7.34 | 9 | 6.02E-01 |
|  |  | Inverse variance weighted | 12.54 | 10 | 2.51E-01 |
| frontal pole | AD | MR Egger | 6.44 | 4 | 1.69E-01 |
|  |  | Inverse variance weighted | 6.97 | 5 | 2.23E-01 |
| lateral orbitofrontal | AD | MR Egger | 16.74 | 19 | 6.07E-01 |
|  |  | Inverse variance weighted | 17.43 | 20 | 6.25E-01 |
| medial orbitofrontal | AD | MR Egger | 0.88 | 3 | 8.31E-01 |
|  |  | Inverse variance weighted | 1.49 | 4 | 8.29E-01 |
| paracentral | AD | MR Egger | 7.81 | 7 | 3.50E-01 |
|  |  | Inverse variance weighted | 8.41 | 8 | 3.95E-01 |
| parsopercularis | AD | MR Egger | 15.05 | 5 | 1.01E-02 |
|  |  | Inverse variance weighted | 16.05 | 6 | 1.35E-02 |
| parsorbitalis | AD | MR Egger | 7.11 | 9 | 6.26E-01 |
|  |  | Inverse variance weighted | 7.22 | 10 | 7.04E-01 |
| parstriangularis | AD | MR Egger | 27.50 | 15 | 2.49E-02 |
|  |  | Inverse variance weighted | 28.07 | 16 | 3.10E-02 |
| precentral | AD | MR Egger | 15.79 | 10 | 1.06E-01 |
|  |  | Inverse variance weighted | 20.37 | 11 | 4.05E-02 |
| rostral anterior cingulate | AD | MR Egger | 0.24 | 4 | 9.93E-01 |
|  |  | Inverse variance weighted | 2.92 | 5 | 7.13E-01 |
| rostral middle frontal | AD | MR Egger | 30.95 | 19 | 4.09E-02 |
|  |  | Inverse variance weighted | 35.63 | 20 | 1.70E-02 |
| superior frontal | AD | MR Egger | 9.84 | 12 | 6.30E-01 |
|  |  | Inverse variance weighted | 10.13 | 13 | 6.83E-01 |
| banks of the superior temporal sulcus | AD | MR Egger | 7.01 | 6 | 3.20E-01 |
|  |  | Inverse variance weighted | 7.67 | 7 | 3.62E-01 |
| entorhinal | AD | MR Egger | 14.03 | 10 | 1.72E-01 |
|  |  | Inverse variance weighted | 14.08 | 11 | 2.28E-01 |
| fusiform | AD | MR Egger | 17.11 | 6 | 8.88E-03 |
|  |  | Inverse variance weighted | 17.25 | 7 | 1.58E-02 |
| inferior temporal | AD | MR Egger | 12.18 | 9 | 2.04E-01 |
|  |  | Inverse variance weighted | 13.33 | 10 | 2.06E-01 |
| insula | AD | MR Egger | 17.90 | 13 | 1.62E-01 |
|  |  | Inverse variance weighted | 18.54 | 14 | 1.83E-01 |
| middle temporal | AD | MR Egger | 16.43 | 10 | 8.79E-02 |
|  |  | Inverse variance weighted | 16.47 | 11 | 1.25E-01 |
| parahippocampal | AD | MR Egger | 11.05 | 6 | 8.69E-02 |
|  |  | Inverse variance weighted | 11.05 | 7 | 1.36E-01 |
| superior temporal | AD | MR Egger | 11.93 | 10 | 2.90E-01 |
|  |  | Inverse variance weighted | 12.56 | 11 | 3.23E-01 |
| temporal pole | AD | MR Egger | 0.36 | 2 | 8.37E-01 |
|  |  | Inverse variance weighted | 1.30 | 3 | 7.29E-01 |
| transverse temporal | AD | MR Egger | 24.33 | 12 | 1.83E-02 |
|  |  | Inverse variance weighted | 25.04 | 13 | 2.28E-02 |
| inferior parietal | AD | MR Egger | 23.24 | 17 | 1.42E-01 |
|  |  | Inverse variance weighted | 23.47 | 18 | 1.73E-01 |
| isthmus cingulate | AD | MR Egger | 2.91 | 5 | 7.15E-01 |
|  |  | Inverse variance weighted | 3.98 | 6 | 6.80E-01 |
| postcentral | AD | MR Egger | 13.84 | 11 | 2.42E-01 |
|  |  | Inverse variance weighted | 14.36 | 12 | 2.78E-01 |
| posterior cingulate | AD | MR Egger | 15.91 | 7 | 2.60E-02 |
|  |  | Inverse variance weighted | 15.91 | 8 | 4.37E-02 |
| precuneus | AD | MR Egger | 22.38 | 18 | 2.16E-01 |
|  |  | Inverse variance weighted | 24.14 | 19 | 1.91E-01 |
| superior parietal | AD | MR Egger | 12.35 | 16 | 7.20E-01 |
|  |  | Inverse variance weighted | 13.09 | 17 | 7.30E-01 |
| supramarginal | AD | MR Egger | 9.48 | 9 | 3.94E-01 |
|  |  | Inverse variance weighted | 10.28 | 10 | 4.16E-01 |
| cuneus | AD | MR Egger | 13.36 | 11 | 2.70E-01 |
|  |  | Inverse variance weighted | 13.95 | 12 | 3.04E-01 |
| lateral occipital | AD | MR Egger | 18.65 | 14 | 1.79E-01 |
|  |  | Inverse variance weighted | 19.05 | 15 | 2.12E-01 |
| lingual | AD | MR Egger | 18.81 | 19 | 4.69E-01 |
|  |  | Inverse variance weighted | 19.47 | 20 | 4.92E-01 |
| pericalcarine | AD | MR Egger | 53.52 | 36 | 3.03E-02 |
|  |  | Inverse variance weighted | 53.75 | 37 | 3.69E-02 |
| whole cortex | AD | MR Egger | 57.11 | 27 | 6.23E-04 |
|  |  | Inverse variance weighted | 59.16 | 28 | 5.20E-04 |

**Table S10.** Sensitivity analysis and pleiotropy analysis for causal effect of cortical thickness on Alzheimer’s Disease

| **Exposure** | **Lobe** | **Outcome** | **No. of SNPs** | **MR Egger** | | | **Weighted median** | | **MR PRESSO** | | **Horizontal pleiotropy** |
| --- | --- | --- | --- | --- | --- | --- | --- | --- | --- | --- | --- |
|  |  |  |  | **I^2^_GX_** | **OR (95%CI)** | **P** | **OR (95%CI)** | **P** | **Global test P** | **Correct P*** | **Egger Intercept P** |
| Whole cortex | global | AD | 20 |  | 0.85 (0.75, 0.97) | 0.023 | 1 (0.96, 1.03) | 0.932 | 0.112 | NA | 0.031 |
| Caudal anterior cingulate | frontal | AD | 5 |  | 0.96 (0.77, 1.2) | 0.753 | 1.03 (0.96, 1.1) | 0.472 | 0.439 | NA | 0.632 |
| Caudal middle frontal | frontal | AD | 5 |  | 1.09 (0.85, 1.4) | 0.546 | 1.07 (0.97, 1.17) | 0.156 | 0.333 | NA | 0.802 |
| Frontal pole | frontal | AD | 6 |  | 0.82 (0.65, 1.02) | 0.149 | 0.97 (0.91, 1.03) | 0.318 | 0.488 | NA | 0.212 |
| Lateral orbitofrontal | frontal | AD | 4 |  | 1.09 (0.72, 1.65) | 0.714 | 0.99 (0.89, 1.11) | 0.916 | 0.518 | NA | 0.636 |
| Medial orbitofrontal | frontal | AD | 3 |  | 0.89 (0.46, 1.7) | 0.78 | 0.96 (0.87, 1.05) | 0.376 | NA | NA | 0.885 |
| paracentral | frontal | AD | 5 |  | 1.11 (0.48, 2.55) | 0.824 | 1.11 (1.02, 1.21) | 0.012 | 0.048 | 0.008 | 0.929 |
| Pars opercularis | frontal | AD | 9 |  | 1.11 (0.92, 1.35) | 0.3 | 0.99 (0.91, 1.08) | 0.868 | 0.066 | NA | 0.362 |
| Pars orbitalis | frontal | AD | 3 |  | 0.72 (0.25, 2.04) | 0.648 | 0.97 (0.87, 1.09) | 0.628 | NA | NA | 0.672 |
| Pars triangularis | frontal | AD | 4 |  | 1.01 (0.76, 1.33) | 0.96 | 1 (0.9, 1.12) | 0.943 | 0.76 | NA | 0.926 |
| precentral | frontal | AD | 8 |  | 0.97 (0.82, 1.15) | 0.756 | 0.98 (0.91, 1.07) | 0.71 | 0.106 | NA | 0.901 |
| Rostral anterior cingulate | frontal | AD | 7 |  | 1.09 (0.92, 1.29) | 0.381 | 0.99 (0.92, 1.06) | 0.729 | 0.121 | NA | 0.338 |
| Rostral middle frontal | frontal | AD | 8 |  | 0.71 (0.55, 0.91) | 0.034 | 0.98 (0.87, 1.11) | 0.804 | 0.143 | NA | 0.044 |
| Superior frontal | frontal | AD | 8 |  | 0.91 (0.72, 1.15) | 0.447 | 0.98 (0.9, 1.06) | 0.583 | 0.264 | NA | 0.448 |
| banks of the superior temporal sulcus | temporal | AD | 5 |  | 0.76 (0.54, 1.06) | 0.207 | 1.01 (0.94, 1.09) | 0.808 | 0.243 | NA | 0.177 |
| entorhinal | temporal | AD | 4 |  | 0.96 (0.72, 1.29) | 0.824 | 0.97 (0.92, 1.03) | 0.327 | 0.824 | NA | 0.986 |
| fusiform | temporal | AD | 3 |  | 1.12 (0.61, 2.04) | 0.775 | 1.02 (0.93, 1.12) | 0.702 | NA | NA | 0.805 |
| Inferior temporal | temporal | AD | 2 |  | NA | NA | NA | NA | NA | NA | NA |
| insula | temporal | AD | 6 |  | 0.95 (0.78, 1.15) | 0.616 | 0.98 (0.9, 1.05) | 0.531 | 0.365 | NA | 0.813 |
| Middle temporal | temporal | AD | 10 |  | 0.94 (0.67, 1.32) | 0.734 | 1 (0.93, 1.07) | 0.969 | 0.479 | NA | 0.815 |
| parahippocampal | temporal | AD | 12 |  | 0.99 (0.84, 1.16) | 0.865 | 1.02 (0.97, 1.06) | 0.486 | 0.343 | NA | 0.641 |
| Superior temporal | temporal | AD | 9 |  | 1.14 (0.94, 1.39) | 0.224 | 1.06 (0.98, 1.13) | 0.128 | 0.35 | NA | 0.325 |
| Temporal pole | temporal | AD | 5 |  | 1.12 (0.62, 2.02) | 0.729 | 0.95 (0.88, 1.03) | 0.251 | 0.01 | 0.662 | 0.764 |
| Transverse temporal | temporal | AD | 4 |  | 0.99 (0.85, 1.16) | 0.926 | 0.99 (0.92, 1.06) | 0.693 | 0.981 | NA | 0.985 |
| Inferior parietal | parietal | AD | 5 |  | 1.11 (0.93, 1.32) | 0.335 | 1.03 (0.92, 1.15) | 0.631 | 0.725 | NA | 0.564 |
| Isthmus cingulate | parietal | AD | 8 |  | 0.9 (0.65, 1.24) | 0.537 | 1 (0.95, 1.05) | 0.933 | 0.57 | NA | 0.49 |
| postcentral | parietal | AD | 15 |  | 0.95 (0.9, 1.01) | 0.136 | 0.96 (0.93, 1) | 0.03 | 0.002 | 0.601 | 0.136 |
| Posterior cingulate | parietal | AD | 7 |  | 1.07 (0.75, 1.55) | 0.714 | 0.96 (0.89, 1.03) | 0.269 | 0.047 | 0.087 | 0.602 |
| precuneus | parietal | AD | 9 |  | 0.84 (0.73, 0.97) | 0.054 | 1.08 (1, 1.16) | 0.053 | 0.156 | NA | 0.02 |
| Superior parietal | parietal | AD | 7 |  | 1.06 (0.79, 1.43) | 0.704 | 1.05 (0.97, 1.12) | 0.209 | 0.156 | NA | 0.83 |
| supramarginal | parietal | AD | 6 |  | 0.79 (0.52, 1.19) | 0.318 | 1.05 (0.93, 1.18) | 0.424 | 0.154 | NA | 0.252 |
| cuneus | occipital | AD | 5 | 0.96 | 0.92 (0.83, 1.02) | 0.219 | 0.93 (0.88, 0.99) | 0.023 | 0.754 | NA | 0.78 |
| Lateral occipital | occipital | AD | 8 |  | 0.84 (0.62, 1.15) | 0.322 | 0.97 (0.9, 1.04) | 0.331 | 0.005 | 0.282 | 0.297 |
| lingual | occipital | AD | 8 |  | 0.9 (0.68, 1.18) | 0.459 | 1.06 (0.99, 1.14) | 0.091 | 0.196 | NA | 0.343 |
| pericalcarine | occipital | AD | 5 |  | 0.98 (0.62, 1.56) | 0.944 | 0.95 (0.88, 1.02) | 0.124 | 0.364 | NA | 0.895 |

AD, Alzheimer’s Disease; SNP, single nucleotide polymorphism; OR: odds ratio genetically predicted 1-SD unit increase in the cortical thickness; CI: confidence interval; MR PRESSO, MR Pleiotropy Residual Sum and Outlier. I2GX is calculated to measure the violation of NO Measurement Error (NOME) assumption in MR Egger. I2GX < 0.9 means there is potential violation of NOME assumption in MR Egger. *If MR PRESSO Global test detect the horizontal pleiotropy and there is significant difference before and after removing outlier, correct P is calculated by removing instruments variants which have horizontal pleiotropy.

**Table S11.** Leave-one-out(loo) analysis of association between genetically predicted cortical thickness and Alzheimer's disease (AD) risk

| **exposure** | **outcome** | **SNP** | **b** | **se** | **p** |
| --- | --- | --- | --- | --- | --- |
| caudalanteriorcingulate | AD | rs111635253 | 0.02021 | 0.03921 | 6.06E-01 |
| caudalanteriorcingulate | AD | rs11778360 | 0.00666 | 0.03558 | 8.52E-01 |
| caudalanteriorcingulate | AD | rs73528979 | 0.02193 | 0.03798 | 5.64E-01 |
| caudalanteriorcingulate | AD | rs7737603 | 0.00673 | 0.03478 | 8.46E-01 |
| caudalanteriorcingulate | AD | rs9929528 | 0.03480 | 0.02835 | 2.20E-01 |
| caudalmiddlefrontal | AD | rs11748651 | 0.04843 | 0.05230 | 3.54E-01 |
| caudalmiddlefrontal | AD | rs13135092 | 0.03205 | 0.04015 | 4.25E-01 |
| caudalmiddlefrontal | AD | rs73004648 | 0.04596 | 0.04821 | 3.40E-01 |
| caudalmiddlefrontal | AD | rs73006822 | 0.08755 | 0.04028 | 2.97E-02 |
| caudalmiddlefrontal | AD | rs74700892 | 0.05599 | 0.05090 | 2.71E-01 |
| frontalpole | AD | rs56023709 | -0.04452 | 0.03050 | 1.44E-01 |
| frontalpole | AD | rs73596628 | -0.02056 | 0.02606 | 4.30E-01 |
| frontalpole | AD | rs748319 | -0.04970 | 0.02562 | 5.24E-02 |
| frontalpole | AD | rs77128055 | -0.03416 | 0.02682 | 2.03E-01 |
| frontalpole | AD | rs78759106 | -0.03337 | 0.02620 | 2.03E-01 |
| frontalpole | AD | rs9828792 | -0.04408 | 0.02581 | 8.77E-02 |
| lateralorbitofrontal | AD | rs11609649 | -0.03623 | 0.05673 | 5.23E-01 |
| lateralorbitofrontal | AD | rs59698523 | -0.03681 | 0.04313 | 3.93E-01 |
| lateralorbitofrontal | AD | rs62394879 | -0.03378 | 0.05822 | 5.62E-01 |
| lateralorbitofrontal | AD | rs6983605 | 0.01030 | 0.04935 | 8.35E-01 |
| medialorbitofrontal | AD | rs177080 | -0.08602 | 0.04708 | 6.77E-02 |
| medialorbitofrontal | AD | rs35515850 | -0.02599 | 0.04462 | 5.60E-01 |
| medialorbitofrontal | AD | rs78728690 | -0.07074 | 0.07052 | 3.16E-01 |
| paracentral | AD | rs11759026 | 0.04191 | 0.05771 | 4.68E-01 |
| paracentral | AD | rs2003698 | 0.04666 | 0.05220 | 3.71E-01 |
| paracentral | AD | rs2525990 | 0.10965 | 0.03480 | 1.63E-03 |
| paracentral | AD | rs35288226 | 0.05133 | 0.05782 | 3.75E-01 |
| paracentral | AD | rs7854681 | 0.06237 | 0.05779 | 2.80E-01 |
| parsopercularis | AD | rs10987671 | 0.02685 | 0.03994 | 5.01E-01 |
| parsopercularis | AD | rs117421027 | 0.02538 | 0.04574 | 5.79E-01 |
| parsopercularis | AD | rs12517522 | 0.02452 | 0.04390 | 5.76E-01 |
| parsopercularis | AD | rs143482636 | -0.00482 | 0.03170 | 8.79E-01 |
| parsopercularis | AD | rs17694988 | -0.00249 | 0.03975 | 9.50E-01 |
| parsopercularis | AD | rs3740221 | 0.02803 | 0.04434 | 5.27E-01 |
| parsopercularis | AD | rs3904916 | 0.02948 | 0.04298 | 4.93E-01 |
| parsopercularis | AD | rs77303563 | 0.03654 | 0.04124 | 3.76E-01 |
| parsopercularis | AD | rs9459027 | 0.03308 | 0.04306 | 4.42E-01 |
| parsorbitalis | AD | rs10871753 | -0.08418 | 0.06963 | 2.27E-01 |
| parsorbitalis | AD | rs34754191 | -0.00304 | 0.06237 | 9.61E-01 |
| parsorbitalis | AD | rs72766503 | -0.01594 | 0.05205 | 7.59E-01 |
| parstriangularis | AD | rs11577200 | 0.00006 | 0.05810 | 9.99E-01 |
| parstriangularis | AD | rs35320047 | 0.03768 | 0.05608 | 5.02E-01 |
| parstriangularis | AD | rs6965709 | 0.01784 | 0.04692 | 7.04E-01 |
| parstriangularis | AD | rs76782422 | 0.03295 | 0.05681 | 5.62E-01 |
| precentral | AD | rs112216293 | -0.00207 | 0.04139 | 9.60E-01 |
| precentral | AD | rs117753404 | -0.01545 | 0.04529 | 7.33E-01 |
| precentral | AD | rs149866019 | -0.02480 | 0.04383 | 5.72E-01 |
| precentral | AD | rs1880694 | -0.03533 | 0.04186 | 3.99E-01 |
| precentral | AD | rs2277403 | -0.04071 | 0.03385 | 2.29E-01 |
| precentral | AD | rs3752962 | -0.00352 | 0.04070 | 9.31E-01 |
| precentral | AD | rs3852000 | -0.00349 | 0.04074 | 9.32E-01 |
| precentral | AD | rs73150969 | -0.02196 | 0.04611 | 6.34E-01 |
| rostralanteriorcingulate | AD | rs116956554 | 0.00961 | 0.03223 | 7.66E-01 |
| rostralanteriorcingulate | AD | rs12698894 | 0.00405 | 0.04122 | 9.22E-01 |
| rostralanteriorcingulate | AD | rs192834490 | 0.01739 | 0.03638 | 6.33E-01 |
| rostralanteriorcingulate | AD | rs323483 | 0.00071 | 0.04111 | 9.86E-01 |
| rostralanteriorcingulate | AD | rs72768970 | -0.02542 | 0.02977 | 3.93E-01 |
| rostralanteriorcingulate | AD | rs77479170 | -0.00598 | 0.03633 | 8.69E-01 |
| rostralanteriorcingulate | AD | rs79927507 | -0.01077 | 0.04128 | 7.94E-01 |
| rostralmiddlefrontal | AD | rs10103320 | -0.05116 | 0.05488 | 3.51E-01 |
| rostralmiddlefrontal | AD | rs111297085 | -0.02707 | 0.05893 | 6.46E-01 |
| rostralmiddlefrontal | AD | rs13164785 | -0.07240 | 0.05847 | 2.16E-01 |
| rostralmiddlefrontal | AD | rs1741344 | -0.07293 | 0.05677 | 1.99E-01 |
| rostralmiddlefrontal | AD | rs2959992 | -0.02494 | 0.04878 | 6.09E-01 |
| rostralmiddlefrontal | AD | rs62320715 | -0.04333 | 0.05649 | 4.43E-01 |
| rostralmiddlefrontal | AD | rs6671321 | 0.00145 | 0.05602 | 9.79E-01 |
| rostralmiddlefrontal | AD | rs7236701 | -0.05311 | 0.05406 | 3.26E-01 |
| superiorfrontal | AD | rs11748651 | -0.02159 | 0.04223 | 6.09E-01 |
| superiorfrontal | AD | rs12091574 | -0.00762 | 0.04541 | 8.67E-01 |
| superiorfrontal | AD | rs12779836 | -0.01481 | 0.04318 | 7.32E-01 |
| superiorfrontal | AD | rs12826251 | 0.00223 | 0.04370 | 9.59E-01 |
| superiorfrontal | AD | rs12956276 | -0.02879 | 0.03628 | 4.28E-01 |
| superiorfrontal | AD | rs7199462 | 0.00451 | 0.04297 | 9.16E-01 |
| superiorfrontal | AD | rs7305141 | 0.00198 | 0.04435 | 9.64E-01 |
| superiorfrontal | AD | rs75098304 | 0.01928 | 0.03691 | 6.01E-01 |
| banks of the superior temporal sulcus | AD | rs11259736 | 0.02279 | 0.04363 | 6.01E-01 |
| banks of the superior temporal sulcus | AD | rs11710662 | 0.03096 | 0.03388 | 3.61E-01 |
| banks of the superior temporal sulcus | AD | rs13311548 | 0.01793 | 0.04795 | 7.08E-01 |
| banks of the superior temporal sulcus | AD | rs149142 | 0.05365 | 0.05575 | 3.36E-01 |
| banks of the superior temporal sulcus | AD | rs2416560 | -0.00973 | 0.03527 | 7.83E-01 |
| entorhinal | AD | rs111658633 | -0.04635 | 0.02992 | 1.21E-01 |
| entorhinal | AD | rs17790394 | -0.03985 | 0.02916 | 1.72E-01 |
| entorhinal | AD | rs72812170 | -0.02783 | 0.02950 | 3.45E-01 |
| entorhinal | AD | rs8044158 | -0.04832 | 0.03077 | 1.16E-01 |
| fusiform | AD | rs3742960 | -0.00251 | 0.11431 | 9.82E-01 |
| fusiform | AD | rs62308301 | -0.02027 | 0.07666 | 7.91E-01 |
| fusiform | AD | rs6658111 | 0.12603 | 0.05532 | 2.27E-02 |
| insula | AD | rs10980625 | -0.03675 | 0.03920 | 3.48E-01 |
| insula | AD | rs11086605 | -0.01404 | 0.03320 | 6.72E-01 |
| insula | AD | rs1533034 | -0.03624 | 0.03083 | 2.40E-01 |
| insula | AD | rs4396807 | -0.01465 | 0.03118 | 6.39E-01 |
| insula | AD | rs4843226 | -0.03105 | 0.03739 | 4.06E-01 |
| insula | AD | rs72848575 | -0.04999 | 0.03126 | 1.10E-01 |
| middletemporal | AD | rs10762574 | -0.03298 | 0.02663 | 2.16E-01 |
| middletemporal | AD | rs1344762 | -0.01520 | 0.02793 | 5.86E-01 |
| middletemporal | AD | rs1378358 | -0.01091 | 0.02648 | 6.80E-01 |
| middletemporal | AD | rs2793626 | -0.02408 | 0.02743 | 3.80E-01 |
| middletemporal | AD | rs4839254 | -0.01852 | 0.02785 | 5.06E-01 |
| middletemporal | AD | rs6483830 | -0.00899 | 0.02674 | 7.37E-01 |
| middletemporal | AD | rs7155669 | -0.02681 | 0.02862 | 3.49E-01 |
| middletemporal | AD | rs7563883 | -0.00838 | 0.02662 | 7.53E-01 |
| middletemporal | AD | rs7707060 | -0.02771 | 0.02681 | 3.01E-01 |
| middletemporal | AD | rs79887807 | -0.02336 | 0.02769 | 3.99E-01 |
| parahippocampal | AD | rs115914417 | 0.02807 | 0.01656 | 9.01E-02 |
| parahippocampal | AD | rs12257242 | 0.02499 | 0.01757 | 1.55E-01 |
| parahippocampal | AD | rs16909919 | 0.03313 | 0.01621 | 4.10E-02 |
| parahippocampal | AD | rs17400875 | 0.02517 | 0.01750 | 1.50E-01 |
| parahippocampal | AD | rs206517 | 0.01974 | 0.01670 | 2.37E-01 |
| parahippocampal | AD | rs224697 | 0.02056 | 0.01768 | 2.45E-01 |
| parahippocampal | AD | rs352359 | 0.02494 | 0.01769 | 1.59E-01 |
| parahippocampal | AD | rs4795529 | 0.01917 | 0.01658 | 2.48E-01 |
| parahippocampal | AD | rs5174 | 0.02042 | 0.01695 | 2.28E-01 |
| parahippocampal | AD | rs62348867 | 0.01483 | 0.01610 | 3.57E-01 |
| parahippocampal | AD | rs8028503 | 0.02922 | 0.01654 | 7.74E-02 |
| parahippocampal | AD | rs8183037 | 0.02932 | 0.01652 | 7.59E-02 |
| superiortemporal | AD | rs11706205 | 0.02501 | 0.02837 | 3.78E-01 |
| superiortemporal | AD | rs1344762 | 0.04050 | 0.02672 | 1.30E-01 |
| superiortemporal | AD | rs150356945 | 0.03443 | 0.02886 | 2.33E-01 |
| superiortemporal | AD | rs199441 | 0.02035 | 0.02662 | 4.45E-01 |
| superiortemporal | AD | rs4843227 | 0.04094 | 0.02780 | 1.41E-01 |
| superiortemporal | AD | rs4857715 | 0.02833 | 0.02878 | 3.25E-01 |
| superiortemporal | AD | rs61784835 | 0.01589 | 0.02706 | 5.57E-01 |
| superiortemporal | AD | rs9472314 | 0.04477 | 0.02530 | 7.68E-02 |
| superiortemporal | AD | rs9844757 | 0.02895 | 0.02898 | 3.18E-01 |
| temporalpole | AD | rs113981818 | -0.00510 | 0.05676 | 9.28E-01 |
| temporalpole | AD | rs13344413 | -0.02176 | 0.04500 | 6.29E-01 |
| temporalpole | AD | rs1948948 | 0.03960 | 0.05991 | 5.09E-01 |
| temporalpole | AD | rs36004933 | 0.04266 | 0.05790 | 4.61E-01 |
| temporalpole | AD | rs3858678 | 0.03085 | 0.06227 | 6.20E-01 |
| transversetemporal | AD | rs117236536 | -0.00743 | 0.03377 | 8.26E-01 |
| transversetemporal | AD | rs12221335 | -0.01671 | 0.03495 | 6.33E-01 |
| transversetemporal | AD | rs68044532 | -0.00550 | 0.03554 | 8.77E-01 |
| transversetemporal | AD | rs72831258 | -0.01057 | 0.03869 | 7.85E-01 |
| inferiorparietal | AD | rs111915190 | 0.06995 | 0.05027 | 1.64E-01 |
| inferiorparietal | AD | rs4506714 | 0.06044 | 0.04968 | 2.24E-01 |
| inferiorparietal | AD | rs7124343 | 0.06155 | 0.05084 | 2.26E-01 |
| inferiorparietal | AD | rs76659172 | 0.01865 | 0.05277 | 7.24E-01 |
| inferiorparietal | AD | rs77103055 | 0.04971 | 0.05052 | 3.25E-01 |
| isthmuscingulate | AD | rs10067618 | 0.00269 | 0.02106 | 8.99E-01 |
| isthmuscingulate | AD | rs12412476 | 0.01124 | 0.02044 | 5.82E-01 |
| isthmuscingulate | AD | rs34929358 | 0.02090 | 0.02044 | 3.07E-01 |
| isthmuscingulate | AD | rs3820823 | 0.01510 | 0.02092 | 4.71E-01 |
| isthmuscingulate | AD | rs4962691 | 0.01790 | 0.02169 | 4.09E-01 |
| isthmuscingulate | AD | rs6947897 | 0.01345 | 0.02091 | 5.20E-01 |
| isthmuscingulate | AD | rs9783497 | 0.00085 | 0.02085 | 9.68E-01 |
| isthmuscingulate | AD | rs9870832 | 0.01668 | 0.02061 | 4.18E-01 |
| postcentral | AD | rs10498193 | -0.01805 | 0.02040 | 3.76E-01 |
| postcentral | AD | rs11639391 | 0.03114 | 0.02872 | 2.78E-01 |
| postcentral | AD | rs12128305 | -0.01134 | 0.02122 | 5.93E-01 |
| postcentral | AD | rs13056230 | -0.01302 | 0.02146 | 5.44E-01 |
| postcentral | AD | rs13118125 | -0.01557 | 0.02076 | 4.53E-01 |
| postcentral | AD | rs149625242 | -0.01195 | 0.02157 | 5.80E-01 |
| postcentral | AD | rs1668120 | -0.01210 | 0.02171 | 5.77E-01 |
| postcentral | AD | rs1918352 | -0.00954 | 0.01780 | 5.92E-01 |
| postcentral | AD | rs1938573 | -0.01260 | 0.02146 | 5.57E-01 |
| postcentral | AD | rs2489009 | -0.01257 | 0.01944 | 5.18E-01 |
| postcentral | AD | rs4778990 | -0.01745 | 0.01946 | 3.70E-01 |
| postcentral | AD | rs4823878 | -0.01210 | 0.02090 | 5.63E-01 |
| postcentral | AD | rs55686521 | -0.00960 | 0.02150 | 6.55E-01 |
| postcentral | AD | rs5769797 | -0.00975 | 0.02137 | 6.48E-01 |
| postcentral | AD | rs62521928 | -0.01319 | 0.02164 | 5.42E-01 |
| posteriorcingulate | AD | rs10051155 | -0.02661 | 0.04411 | 5.46E-01 |
| posteriorcingulate | AD | rs10117809 | -0.02664 | 0.04457 | 5.50E-01 |
| posteriorcingulate | AD | rs143550882 | -0.02598 | 0.04086 | 5.25E-01 |
| posteriorcingulate | AD | rs2415142 | -0.02586 | 0.04487 | 5.64E-01 |
| posteriorcingulate | AD | rs3111626 | 0.00422 | 0.03410 | 9.01E-01 |
| posteriorcingulate | AD | rs4924345 | -0.05887 | 0.02863 | 3.97E-02 |
| posteriorcingulate | AD | rs7700617 | -0.03964 | 0.04201 | 3.45E-01 |
| precuneus | AD | rs11641011 | 0.02965 | 0.03742 | 4.28E-01 |
| precuneus | AD | rs11738990 | 0.02799 | 0.03486 | 4.22E-01 |
| precuneus | AD | rs12485175 | 0.02361 | 0.03537 | 5.04E-01 |
| precuneus | AD | rs1452628 | 0.02740 | 0.03494 | 4.33E-01 |
| precuneus | AD | rs150000053 | 0.06293 | 0.02962 | 3.36E-02 |
| precuneus | AD | rs4326243 | 0.03645 | 0.03712 | 3.26E-01 |
| precuneus | AD | rs623640 | 0.04957 | 0.03378 | 1.42E-01 |
| precuneus | AD | rs6658111 | 0.02345 | 0.03738 | 5.30E-01 |
| precuneus | AD | rs79583401 | 0.04010 | 0.03665 | 2.74E-01 |
| superiorparietal | AD | rs11641011 | 0.02066 | 0.04151 | 6.19E-01 |
| superiorparietal | AD | rs1452628 | 0.01451 | 0.04101 | 7.23E-01 |
| superiorparietal | AD | rs216527 | 0.04764 | 0.02888 | 9.90E-02 |
| superiorparietal | AD | rs4843226 | 0.03392 | 0.04956 | 4.94E-01 |
| superiorparietal | AD | rs61784835 | 0.01283 | 0.03651 | 7.25E-01 |
| superiorparietal | AD | rs72748146 | 0.02992 | 0.03801 | 4.31E-01 |
| superiorparietal | AD | rs73215353 | 0.03685 | 0.03873 | 3.41E-01 |
| supramarginal | AD | rs11250157 | 0.04564 | 0.07057 | 5.18E-01 |
| supramarginal | AD | rs2282910 | 0.00087 | 0.05924 | 9.88E-01 |
| supramarginal | AD | rs72794732 | 0.07564 | 0.04887 | 1.22E-01 |
| supramarginal | AD | rs7685753 | 0.01181 | 0.06860 | 8.63E-01 |
| supramarginal | AD | rs78138595 | 0.02073 | 0.07183 | 7.73E-01 |
| supramarginal | AD | rs865010 | 0.03725 | 0.07380 | 6.14E-01 |
| cuneus | AD | rs117923045 | -0.06307 | 0.02790 | 2.38E-02 |
| cuneus | AD | rs12810533 | -0.05939 | 0.02681 | 2.67E-02 |
| cuneus | AD | rs13107325 | -0.05973 | 0.02728 | 2.86E-02 |
| cuneus | AD | rs34163390 | -0.07533 | 0.02649 | 4.46E-03 |
| cuneus | AD | rs8005394 | -0.08096 | 0.02953 | 6.12E-03 |
| lateraloccipital | AD | rs12953229 | 0.00625 | 0.05306 | 9.06E-01 |
| lateraloccipital | AD | rs13107325 | 0.02426 | 0.04812 | 6.14E-01 |
| lateraloccipital | AD | rs1411164 | 0.00973 | 0.05238 | 8.53E-01 |
| lateraloccipital | AD | rs245105 | -0.03534 | 0.02989 | 2.37E-01 |
| lateraloccipital | AD | rs256376 | 0.01028 | 0.05076 | 8.40E-01 |
| lateraloccipital | AD | rs5747112 | -0.01217 | 0.04872 | 8.03E-01 |
| lateraloccipital | AD | rs595789 | 0.00115 | 0.05181 | 9.82E-01 |
| lateraloccipital | AD | rs62200349 | 0.01669 | 0.05005 | 7.39E-01 |
| lingual | AD | rs10495963 | 0.00996 | 0.03313 | 7.64E-01 |
| lingual | AD | rs11760588 | 0.01864 | 0.03554 | 6.00E-01 |
| lingual | AD | rs12591705 | 0.03205 | 0.03628 | 3.77E-01 |
| lingual | AD | rs13135092 | 0.05877 | 0.02981 | 4.87E-02 |
| lingual | AD | rs13180395 | 0.03522 | 0.03181 | 2.68E-01 |
| lingual | AD | rs1391550 | 0.03013 | 0.03636 | 4.07E-01 |
| lingual | AD | rs73239710 | 0.02088 | 0.03539 | 5.55E-01 |
| lingual | AD | rs9537915 | 0.02845 | 0.03440 | 4.08E-01 |
| pericalcarine | AD | rs13262416 | -0.06964 | 0.05319 | 1.90E-01 |
| pericalcarine | AD | rs2164950 | -0.04631 | 0.05442 | 3.95E-01 |
| pericalcarine | AD | rs34324736 | -0.04084 | 0.03756 | 2.77E-01 |
| pericalcarine | AD | rs4682946 | -0.05712 | 0.03116 | 6.68E-02 |
| pericalcarine | AD | rs629404 | -0.04843 | 0.04986 | 3.31E-01 |
| whole cortex | AD | rs11656696 | -0.01473 | 0.01616 | 3.62E-01 |
| whole cortex | AD | rs11692435 | -0.01203 | 0.01727 | 4.86E-01 |
| whole cortex | AD | rs117826338 | -0.00301 | 0.01384 | 8.28E-01 |
| whole cortex | AD | rs1180331 | -0.01135 | 0.01689 | 5.02E-01 |
| whole cortex | AD | rs12938775 | -0.01458 | 0.01704 | 3.92E-01 |
| whole cortex | AD | rs13107325 | -0.00851 | 0.01616 | 5.99E-01 |
| whole cortex | AD | rs1742401 | -0.01381 | 0.01695 | 4.15E-01 |
| whole cortex | AD | rs2316766 | -0.00874 | 0.01490 | 5.57E-01 |
| whole cortex | AD | rs3200031 | -0.01272 | 0.01691 | 4.52E-01 |
| whole cortex | AD | rs35021943 | -0.01181 | 0.01706 | 4.89E-01 |
| whole cortex | AD | rs3770776 | -0.01672 | 0.01656 | 3.12E-01 |
| whole cortex | AD | rs3816046 | -0.01937 | 0.01560 | 2.14E-01 |
| whole cortex | AD | rs40565 | -0.01376 | 0.01697 | 4.17E-01 |
| whole cortex | AD | rs533577 | -0.01672 | 0.01703 | 3.26E-01 |
| whole cortex | AD | rs5994871 | -0.01357 | 0.01698 | 4.24E-01 |
| whole cortex | AD | rs6738528 | -0.01389 | 0.01711 | 4.17E-01 |
| whole cortex | AD | rs724265 | -0.01209 | 0.01695 | 4.75E-01 |
| whole cortex | AD | rs7531555 | -0.01630 | 0.01673 | 3.30E-01 |
| whole cortex | AD | rs7657284 | -0.01461 | 0.01688 | 3.87E-01 |
| whole cortex | AD | rs7824177 | -0.01524 | 0.01703 | 3.71E-01 |

**Table S12.** Single SNP analysis of association between genetically predicted cortical thickness and Alzheimer's disease (AD) risk

| **exposure** | **outcome** | **SNP** | **b** | **se** | **p** |
| --- | --- | --- | --- | --- | --- |
| caudalanteriorcingulate | AD | rs111635253 | 0.01401 | 0.05310 | 7.92E-01 |
| caudalanteriorcingulate | AD | rs11778360 | 0.05912 | 0.05685 | 2.98E-01 |
| caudalanteriorcingulate | AD | rs73528979 | 0.00674 | 0.05785 | 9.07E-01 |
| caudalanteriorcingulate | AD | rs7737603 | 0.06404 | 0.05960 | 2.83E-01 |
| caudalanteriorcingulate | AD | rs9929528 | -0.15538 | 0.09287 | 9.43E-02 |
| caudalmiddlefrontal | AD | rs11748651 | 0.06989 | 0.07125 | 3.27E-01 |
| caudalmiddlefrontal | AD | rs13135092 | 0.15393 | 0.08561 | 7.22E-02 |
| caudalmiddlefrontal | AD | rs73004648 | 0.09501 | 0.08971 | 2.90E-01 |
| caudalmiddlefrontal | AD | rs73006822 | -0.09277 | 0.08426 | 2.71E-01 |
| caudalmiddlefrontal | AD | rs74700892 | 0.04656 | 0.07938 | 5.57E-01 |
| frontalpole | AD | rs56023709 | -0.02348 | 0.04141 | 5.71E-01 |
| frontalpole | AD | rs73596628 | -0.12546 | 0.05931 | 3.44E-02 |
| frontalpole | AD | rs748319 | 0.04177 | 0.06543 | 5.23E-01 |
| frontalpole | AD | rs77128055 | -0.06033 | 0.06644 | 3.64E-01 |
| frontalpole | AD | rs78759106 | -0.07120 | 0.07185 | 3.22E-01 |
| frontalpole | AD | rs9828792 | 0.00470 | 0.06513 | 9.42E-01 |
| lateralorbitofrontal | AD | rs11609649 | -0.00033 | 0.07389 | 9.96E-01 |
| lateralorbitofrontal | AD | rs59698523 | 0.16227 | 0.16976 | 3.39E-01 |
| lateralorbitofrontal | AD | rs62394879 | -0.00695 | 0.07202 | 9.23E-01 |
| lateralorbitofrontal | AD | rs6983605 | -0.11377 | 0.07865 | 1.48E-01 |
| medialorbitofrontal | AD | rs177080 | 0.00051 | 0.06868 | 9.94E-01 |
| medialorbitofrontal | AD | rs35515850 | -0.14054 | 0.06797 | 3.87E-02 |
| medialorbitofrontal | AD | rs78728690 | -0.04535 | 0.05871 | 4.40E-01 |
| paracentral | AD | rs11759026 | 0.11766 | 0.05915 | 4.67E-02 |
| paracentral | AD | rs2003698 | 0.15470 | 0.08071 | 5.53E-02 |
| paracentral | AD | rs2525990 | -0.10132 | 0.06464 | 1.17E-01 |
| paracentral | AD | rs35288226 | 0.10455 | 0.06769 | 1.22E-01 |
| paracentral | AD | rs7854681 | 0.06151 | 0.07713 | 4.25E-01 |
| parsopercularis | AD | rs10987671 | -0.24933 | 0.21690 | 2.50E-01 |
| parsopercularis | AD | rs117421027 | 0.00509 | 0.07053 | 9.42E-01 |
| parsopercularis | AD | rs12517522 | -0.00198 | 0.09348 | 9.83E-01 |
| parsopercularis | AD | rs143482636 | 0.24552 | 0.08758 | 5.06E-03 |
| parsopercularis | AD | rs17694988 | 0.14612 | 0.07061 | 3.85E-02 |
| parsopercularis | AD | rs3740221 | -0.01959 | 0.08080 | 8.08E-01 |
| parsopercularis | AD | rs3904916 | -0.05114 | 0.09430 | 5.88E-01 |
| parsopercularis | AD | rs77303563 | -0.09464 | 0.08622 | 2.72E-01 |
| parsopercularis | AD | rs9459027 | -0.05780 | 0.08224 | 4.82E-01 |
| parsorbitalis | AD | rs10871753 | 0.02548 | 0.06901 | 7.12E-01 |
| parsorbitalis | AD | rs34754191 | -0.07057 | 0.07927 | 3.73E-01 |
| parsorbitalis | AD | rs72766503 | -0.13020 | 0.14574 | 3.72E-01 |
| parstriangularis | AD | rs11577200 | 0.06182 | 0.07829 | 4.30E-01 |
| parstriangularis | AD | rs35320047 | -0.01327 | 0.08407 | 8.75E-01 |
| parstriangularis | AD | rs6965709 | 0.38548 | 0.43892 | 3.80E-01 |
| parstriangularis | AD | rs76782422 | -0.00071 | 0.08176 | 9.93E-01 |
| precentral | AD | rs112216293 | -0.12620 | 0.08059 | 1.17E-01 |
| precentral | AD | rs117753404 | -0.03710 | 0.07924 | 6.40E-01 |
| precentral | AD | rs149866019 | 0.03702 | 0.09084 | 6.84E-01 |
| precentral | AD | rs1880694 | 0.08172 | 0.07684 | 2.88E-01 |
| precentral | AD | rs2277403 | 0.18311 | 0.09256 | 4.79E-02 |
| precentral | AD | rs3752962 | -0.14094 | 0.08881 | 1.13E-01 |
| precentral | AD | rs3852000 | -0.14015 | 0.08849 | 1.13E-01 |
| precentral | AD | rs73150969 | -0.00068 | 0.07147 | 9.92E-01 |
| rostralanteriorcingulate | AD | rs116956554 | -0.19107 | 0.10814 | 7.73E-02 |
| rostralanteriorcingulate | AD | rs12698894 | -0.02476 | 0.05814 | 6.70E-01 |
| rostralanteriorcingulate | AD | rs192834490 | -0.08599 | 0.05946 | 1.48E-01 |
| rostralanteriorcingulate | AD | rs323483 | -0.01168 | 0.06086 | 8.48E-01 |
| rostralanteriorcingulate | AD | rs72768970 | 0.13371 | 0.06539 | 4.09E-02 |
| rostralanteriorcingulate | AD | rs77479170 | 0.13467 | 0.14130 | 3.41E-01 |
| rostralanteriorcingulate | AD | rs79927507 | 0.03254 | 0.05470 | 5.52E-01 |
| rostralmiddlefrontal | AD | rs10103320 | 0.15742 | 0.21984 | 4.74E-01 |
| rostralmiddlefrontal | AD | rs111297085 | -0.13020 | 0.10776 | 2.27E-01 |
| rostralmiddlefrontal | AD | rs13164785 | 0.05933 | 0.09087 | 5.14E-01 |
| rostralmiddlefrontal | AD | rs1741344 | 0.07494 | 0.09542 | 4.32E-01 |
| rostralmiddlefrontal | AD | rs2959992 | -0.34386 | 0.17817 | 5.36E-02 |
| rostralmiddlefrontal | AD | rs62320715 | -0.03875 | 0.43799 | 9.30E-01 |
| rostralmiddlefrontal | AD | rs6671321 | -0.16176 | 0.08161 | 4.75E-02 |
| rostralmiddlefrontal | AD | rs7236701 | 0.17844 | 0.20737 | 3.90E-01 |
| superiorfrontal | AD | rs11748651 | 0.08820 | 0.08991 | 3.27E-01 |
| superiorfrontal | AD | rs12091574 | 0.00631 | 0.09110 | 9.45E-01 |
| superiorfrontal | AD | rs12779836 | 0.06893 | 0.10359 | 5.06E-01 |
| superiorfrontal | AD | rs12826251 | -0.06719 | 0.10169 | 5.09E-01 |
| superiorfrontal | AD | rs12956276 | 0.18438 | 0.10398 | 7.62E-02 |
| superiorfrontal | AD | rs7199462 | -0.08567 | 0.10210 | 4.01E-01 |
| superiorfrontal | AD | rs7305141 | -0.05628 | 0.09468 | 5.52E-01 |
| superiorfrontal | AD | rs75098304 | -0.16074 | 0.09206 | 8.08E-02 |
| banks of the superior temporal sulcus | AD | rs11259736 | -0.02150 | 0.16203 | 8.94E-01 |
| banks of the superior temporal sulcus | AD | rs11710662 | -0.20941 | 0.15496 | 1.77E-01 |
| banks of the superior temporal sulcus | AD | rs13311548 | 0.03403 | 0.07022 | 6.28E-01 |
| banks of the superior temporal sulcus | AD | rs149142 | -0.00994 | 0.04380 | 8.20E-01 |
| banks of the superior temporal sulcus | AD | rs2416560 | 0.13623 | 0.06811 | 4.55E-02 |
| entorhinal | AD | rs111658633 | -0.02294 | 0.05114 | 6.54E-01 |
| entorhinal | AD | rs17790394 | -0.04232 | 0.05560 | 4.47E-01 |
| entorhinal | AD | rs72812170 | -0.08158 | 0.05344 | 1.27E-01 |
| entorhinal | AD | rs8044158 | -0.02147 | 0.04750 | 6.51E-01 |
| fusiform | AD | rs3742960 | 0.08625 | 0.07393 | 2.43E-01 |
| fusiform | AD | rs62308301 | 0.17665 | 0.08339 | 3.41E-02 |
| fusiform | AD | rs6658111 | -0.07544 | 0.05320 | 1.56E-01 |
| inferiortemporal | AD | rs111735741 | -0.24571 | 0.15495 | 1.13E-01 |
| inferiortemporal | AD | rs7507079 | 0.06110 | 0.08100 | 4.51E-01 |
| insula | AD | rs10980625 | -0.01372 | 0.05529 | 8.04E-01 |
| insula | AD | rs11086605 | -0.09612 | 0.06372 | 1.31E-01 |
| insula | AD | rs1533034 | 0.13369 | 0.15682 | 3.94E-01 |
| insula | AD | rs4396807 | -0.11525 | 0.07188 | 1.09E-01 |
| insula | AD | rs4843226 | -0.02843 | 0.06806 | 6.76E-01 |
| insula | AD | rs72848575 | 0.06959 | 0.07101 | 3.27E-01 |
| middletemporal | AD | rs10762574 | 0.11398 | 0.08425 | 1.76E-01 |
| middletemporal | AD | rs1344762 | -0.05538 | 0.07648 | 4.69E-01 |
| middletemporal | AD | rs1378358 | -0.11904 | 0.08942 | 1.83E-01 |
| middletemporal | AD | rs2793626 | 0.02758 | 0.08651 | 7.50E-01 |
| middletemporal | AD | rs4839254 | -0.03206 | 0.08878 | 7.18E-01 |
| middletemporal | AD | rs6483830 | -0.11738 | 0.08103 | 1.47E-01 |
| middletemporal | AD | rs7155669 | 0.01604 | 0.06203 | 7.96E-01 |
| middletemporal | AD | rs7563883 | -0.13327 | 0.08458 | 1.15E-01 |
| middletemporal | AD | rs7707060 | 0.05054 | 0.07903 | 5.22E-01 |
| middletemporal | AD | rs79887807 | 0.01747 | 0.08400 | 8.35E-01 |
| parahippocampal | AD | rs115914417 | -0.04299 | 0.06473 | 5.07E-01 |
| parahippocampal | AD | rs12257242 | 0.01409 | 0.05441 | 7.96E-01 |
| parahippocampal | AD | rs16909919 | -0.04297 | 0.04428 | 3.32E-01 |
| parahippocampal | AD | rs17400875 | 0.01087 | 0.05668 | 8.48E-01 |
| parahippocampal | AD | rs206517 | 0.08294 | 0.05773 | 1.51E-01 |
| parahippocampal | AD | rs224697 | 0.05094 | 0.04438 | 2.51E-01 |
| parahippocampal | AD | rs352359 | 0.01601 | 0.05072 | 7.52E-01 |
| parahippocampal | AD | rs4795529 | 0.08652 | 0.05606 | 1.23E-01 |
| parahippocampal | AD | rs5174 | 0.07351 | 0.05754 | 2.01E-01 |
| parahippocampal | AD | rs62348867 | 0.10337 | 0.04696 | 2.77E-02 |
| parahippocampal | AD | rs8028503 | -0.03905 | 0.05578 | 4.84E-01 |
| parahippocampal | AD | rs8183037 | -0.03967 | 0.05551 | 4.75E-01 |
| superiortemporal | AD | rs11706205 | 0.08196 | 0.07361 | 2.66E-01 |
| superiortemporal | AD | rs1344762 | -0.05656 | 0.07811 | 4.69E-01 |
| superiortemporal | AD | rs150356945 | 0.00219 | 0.07619 | 9.77E-01 |
| superiortemporal | AD | rs199441 | 0.11719 | 0.07241 | 1.06E-01 |
| superiortemporal | AD | rs4843227 | -0.03368 | 0.06690 | 6.15E-01 |
| superiortemporal | AD | rs4857715 | 0.05887 | 0.07937 | 4.58E-01 |
| superiortemporal | AD | rs61784835 | 0.10351 | 0.05798 | 7.42E-02 |
| superiortemporal | AD | rs9472314 | -0.11589 | 0.08320 | 1.64E-01 |
| superiortemporal | AD | rs9844757 | 0.05115 | 0.07655 | 5.04E-01 |
| temporalpole | AD | rs113981818 | 0.12241 | 0.06484 | 5.91E-02 |
| temporalpole | AD | rs13344413 | 0.15975 | 0.05849 | 6.31E-03 |
| temporalpole | AD | rs1948948 | -0.06545 | 0.05856 | 2.64E-01 |
| temporalpole | AD | rs36004933 | -0.07710 | 0.05866 | 1.89E-01 |
| temporalpole | AD | rs3858678 | -0.04349 | 0.06306 | 4.90E-01 |
| transversetemporal | AD | rs117236536 | -0.02300 | 0.07563 | 7.61E-01 |
| transversetemporal | AD | rs12221335 | 0.01348 | 0.06550 | 8.37E-01 |
| transversetemporal | AD | rs68044532 | -0.02379 | 0.06202 | 7.01E-01 |
| transversetemporal | AD | rs72831258 | -0.00906 | 0.05105 | 8.59E-01 |
| inferiorparietal | AD | rs111915190 | -0.02402 | 0.10601 | 8.21E-01 |
| inferiorparietal | AD | rs4506714 | 0.01320 | 0.11218 | 9.06E-01 |
| inferiorparietal | AD | rs7124343 | 0.01762 | 0.10115 | 8.62E-01 |
| inferiorparietal | AD | rs76659172 | 0.15004 | 0.08924 | 9.27E-02 |
[truncated: 294,724 more chars]
