# Supplementary material for: Cortical structure and the risk for Alzheimer’s disease: a bidirectional Mendelian randomization study
Source: Transl Psychiatry. 2021 Sep 15;11:476. doi: 10.1038/s41398-021-01599-x (PMC8443658; doi:10.1038/s41398-021-01599-x)
Supplement: Supplementary file 2 — Additional File 2 [file 41398_2021_1599_MOESM2_ESM.pdf]

**Supporting Information 2:** Supplementary figures


| Figures | Contents | Page |
|---|---|---|
| Figure S1 | Causal effect of genetically predicted cortical surface area on Alzheimer's disease using IVW method. | 3 |
| Figure S2 | Leave-one-out analysis for causal estimate of surface area of Temporal pole on AD | 4 |
| Figure S3 | Single SNP analysis for causal estimate of surface area of Temporal pole on AD | 5 |
| Figure S4 | Scatterplot for causal estimate of surface area of Lateral orbitofrontal on AD | 6 |
| Figure S5 | Scatterplot for causal estimate of surface area of Supramarginal on AD | 7 |
| Figure S6 | Scatterplot for causal estimate of surface area of lingual on AD | 8 |
| Figure S7 | Leave-one-out analysis for causal estimate of surface area of Lateral orbitofrontal on AD | 9 |
| Figure S8 | Single SNP analysis for causal estimate of surface area of Lateral orbitofrontal on AD | 10 |
| Figure S9 | Leave-one-out analysis for causal estimate of surface area of Supramarginal on AD | 11 |
| Figure S10 | Single SNP analysis for causal estimate of surface area of Supramarginal on AD | 12 |
| Figure S11 | Leave-one-out analysis for causal estimate of surface area of lingual on AD | 13 |
| Figure S12 | Single SNP analysis for causal estimate of surface area of lingual on AD | 14 |
| Figure S13 | Causal effect of genetically predicted cortical thickness on Alzheimer's disease using IVW method. | 15 |
| Figure S14 | Leave-one-out analysis for causal estimate of thickness of cuneus on AD | 16 |
| Figure S15 | Single SNP analysis for causal estimate of thickness of cuneus on AD | 17 |
| Figure S16 | Causal effect of genetically predicted Alzheimer's disease on cortical surface area using IVW method. | 18 |
| Figure S17 | Leave-one-out analysis for causal estimate of AD on surface area of Precentral | 19 |
| Figure S18 | Single SNP analysis for causal estimate of AD on surface area of Precentral | 20 |
| Figure S19 | Leave-one-out analysis for causal estimate of AD on surface area of Isthmus cingulate | 21 |
| Figure S20 | Single SNP analysis for causal estimate of AD on surface area of Isthmus cingulate | 22 |
| Figure S21 | Scatterplot for causal estimate of AD on surface area of Cuneus | 23 |
| Figure S22 | Scatterplot for causal estimate of AD on surface area of Pericalcarine | 24 |
| Figure S23 | Leave-one-out analysis for causal estimate of AD on surface area of Cuneus | 25 |
| Figure S24 | Single SNP analysis for causal estimate of AD on surface area of Cuneus | 26 |

| | | |
|---|---|---|
| Figure S25 | Leave-one-out analysis for causal estimate of AD on surface area of Pericalcarine | 27 |
| Figure S26 | Single SNP analysis for causal estimate of AD on surface area of Pericalcarine | 28 |
| Figure S27 | Causal effect of genetically predicted Alzheimer's disease on cortical thickness using IVW method. | 29 |
| Figure S28 | Scatterplot for causal estimate of AD on thickness of Cuneus | 30 |
| Figure S29 | Scatterplot for causal estimate of AD on thickness of Lateral occipital | 31 |
| Figure S30 | Scatterplot for causal estimate of AD on thickness of lingual | 32 |
| Figure S31 | Leave-one-out analysis for causal estimate of AD on thickness of Cuneus | 33 |
| Figure S32 | Single SNP analysis for causal estimate of AD on thickness of Cuneus | 34 |
| Figure S33 | Leave-one-out analysis for causal estimate of AD on thickness of Lateral occipital | 35 |
| Figure S34 | Single SNP analysis for causal estimate of AD on thickness of Lateral occipital | 36 |
| Figure S35 | Leave-one-out analysis for causal estimate of AD on thickness of lingual | 37 |
| Figure S36 | Single SNP analysis for causal estimate of AD on thickness of lingual | 38 |

| Exposures | Lobe | No. of SNPs | OR (95%CI) | P |
|---|---|---|---|---|
| **Global** | | | | |
| Whole cortex | global | 29 | 1.01 (0.98, 1.04) | 0.496 |
| **Regional** | | | | |
| Caudal Anterior Cingulate | frontal | 7 | 1.03 (0.98, 1.09) | 0.285 |
| Caudal middle frontal | frontal | 11 | 1.02 (0.98, 1.06) | 0.385 |
| Frontal pole | frontal | 6 | 1 (0.95, 1.05) | 0.872 |
| Lateral orbitofrontal | frontal | 21 | 1.04 (1.01, 1.08) | **0.022** |
| Lateral orbitofrontal* | frontal | 20 | 1.03 (0.99, 1.07) | 0.088 |
| Medial orbitofrontal | frontal | 5 | 1.02 (0.95, 1.1) | 0.546 |
| Paracentral | frontal | 9 | 0.97 (0.93, 1.02) | 0.275 |
| Pars opercularis | frontal | 7 | 1.04 (0.98, 1.12) | 0.21 |
| Pars orbitalis | frontal | 11 | 0.97 (0.93, 1.01) | 0.136 |
| Pars triangularis | frontal | 17 | 0.98 (0.95, 1.02) | 0.312 |
| Precentral | frontal | 12 | 0.98 (0.94, 1.02) | 0.337 |
| Rostral anterior cingulate | frontal | 6 | 1.06 (1, 1.12) | 0.058 |
| Rostral middle frontal | frontal | 21 | 1 (0.95, 1.06) | 0.999 |
| Superior frontal | frontal | 14 | 0.95 (0.89, 1.01) | 0.098 |
| Banks of the superior temporal sulcus | temporal | 8 | 0.96 (0.92, 1) | 0.052 |
| Entorhinal | temporal | 12 | 0.98 (0.94, 1.01) | 0.193 |
| Fusiform | temporal | 8 | 0.98 (0.9, 1.07) | 0.638 |
| Inferior temporal | temporal | 11 | 1.02 (0.96, 1.08) | 0.581 |
| Insula | temporal | 15 | 0.99 (0.95, 1.04) | 0.694 |
| Middle temporal | temporal | 12 | 1.03 (0.97, 1.09) | 0.304 |
| Parahippocampal | temporal | 8 | 1.02 (0.97, 1.08) | 0.446 |
| Superior temporal | temporal | 12 | 0.96 (0.91, 1.01) | 0.086 |
| Temporal pole | temporal | 4 | 0.95 (0.9, 0.997) | **0.04** |
| Transverse temporal | temporal | 14 | 1.01 (0.97, 1.06) | 0.561 |
| Inferior parietal | parietal | 19 | 1.02 (0.98, 1.06) | 0.297 |
| Isthmus cingulate | parietal | 7 | 1.03 (0.97, 1.09) | 0.295 |
| Postcentral | parietal | 13 | 0.99 (0.95, 1.02) | 0.44 |
| Posterior cingulate | parietal | 9 | 1 (0.94, 1.08) | 0.897 |
| Precuneus | parietal | 20 | 0.98 (0.94, 1.02) | 0.27 |
| Superior parietal | parietal | 18 | 1 (0.97, 1.03) | 0.861 |
| Supramarginal | parietal | 11 | 1.05 (1.01, 1.09) | **0.008** |
| Cuneus | occipital | 13 | 1 (0.97, 1.04) | 0.786 |
| Lateral occipital | occipital | 16 | 0.99 (0.95, 1.04) | 0.706 |
| Lingual | occipital | 21 | 1.03 (1.004, 1.06) | **0.024** |
| Pericalcarine | occipital | 38 | 1.01 (0.99, 1.03) | 0.348 |

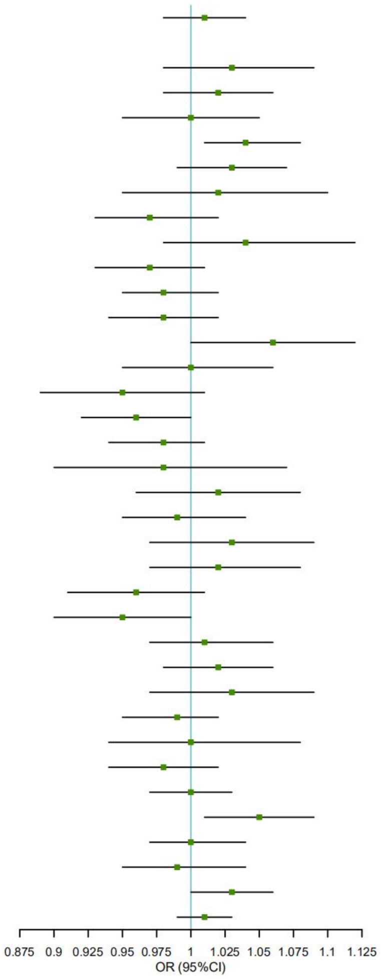


**Figure S1.** Causal effect of genetically predicted cortical surface area on Alzheimer's disease using IVW method.

SNP, single nucleotide polymorphism; OR, odds ratio genetically predicted 1-SD unit increase in the cortical thickness; CI, confidence interval; IVW, inverse variance weighted.

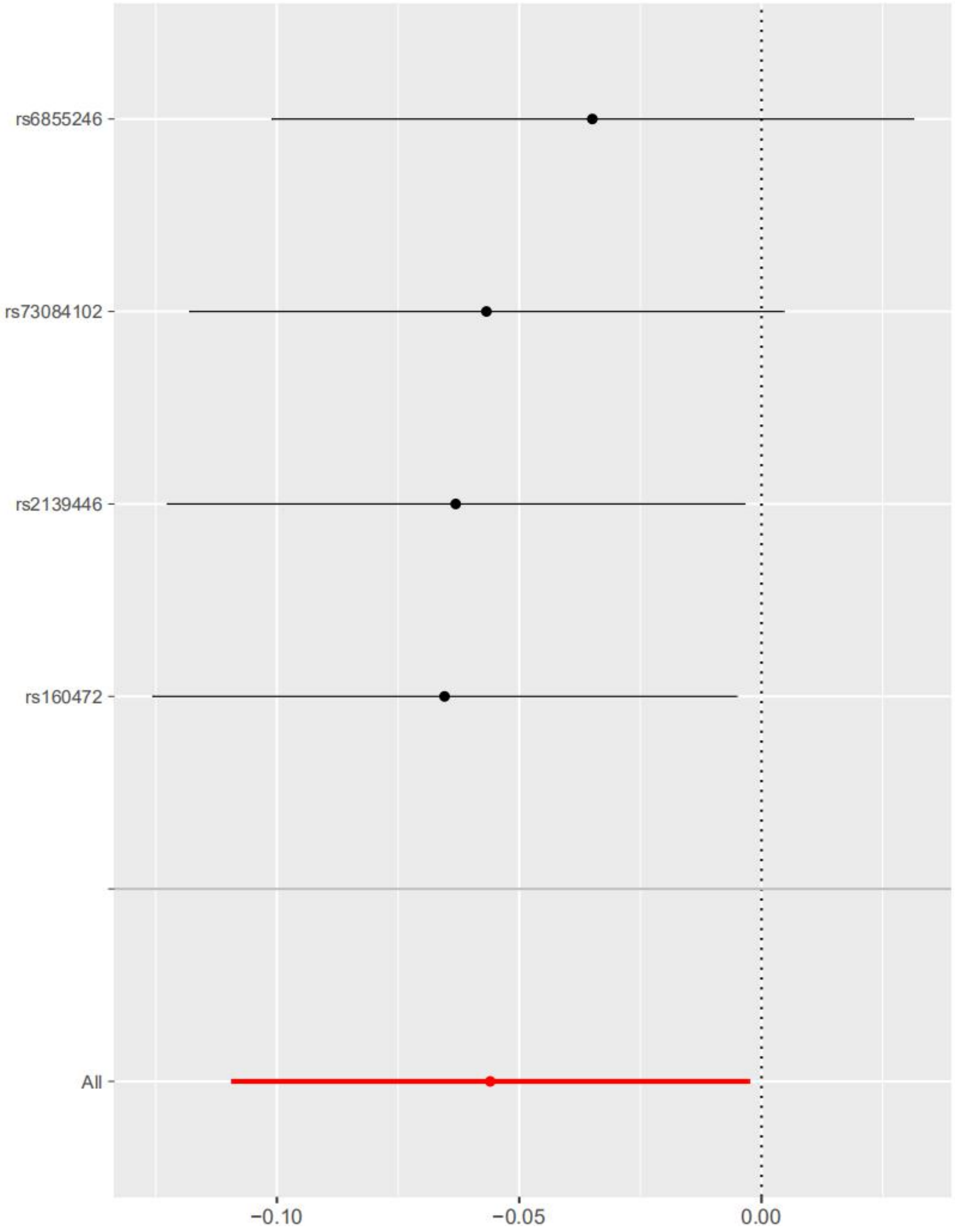


**Figure S2.** Leave-one-out analysis of association between genetically predicted surface of temporal pole and AD.

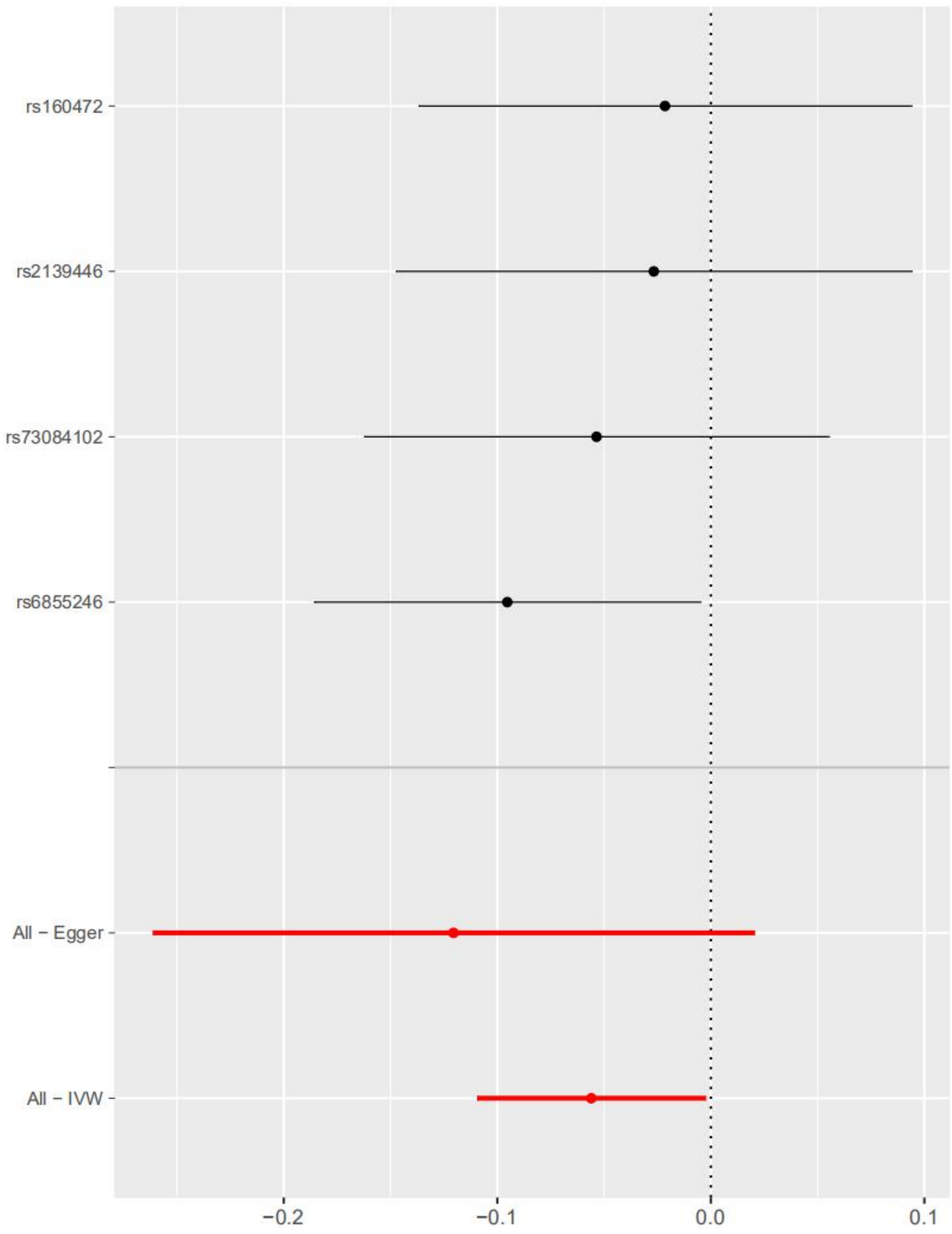


**Figure S3.** Single SNP analysis for individual and combined SNP effects of surface area of temporal pole on AD.

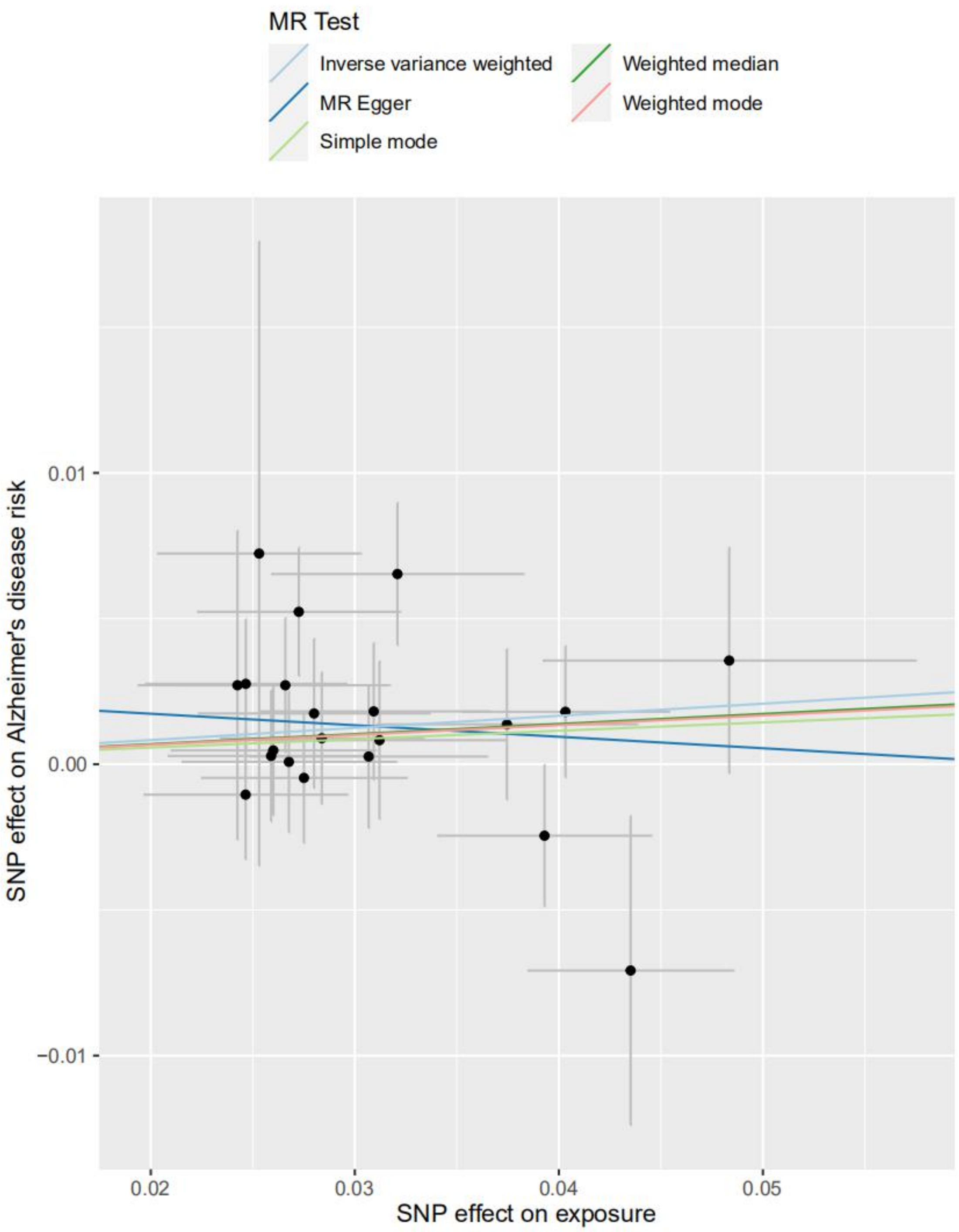


**Figure S4.** Scatterplot of single-nucleotide polymorphism (SNP) associated with surface area of lateral orbitofrontal and AD (vertical and horizontal lines around each SNP show 95% confidence interval).

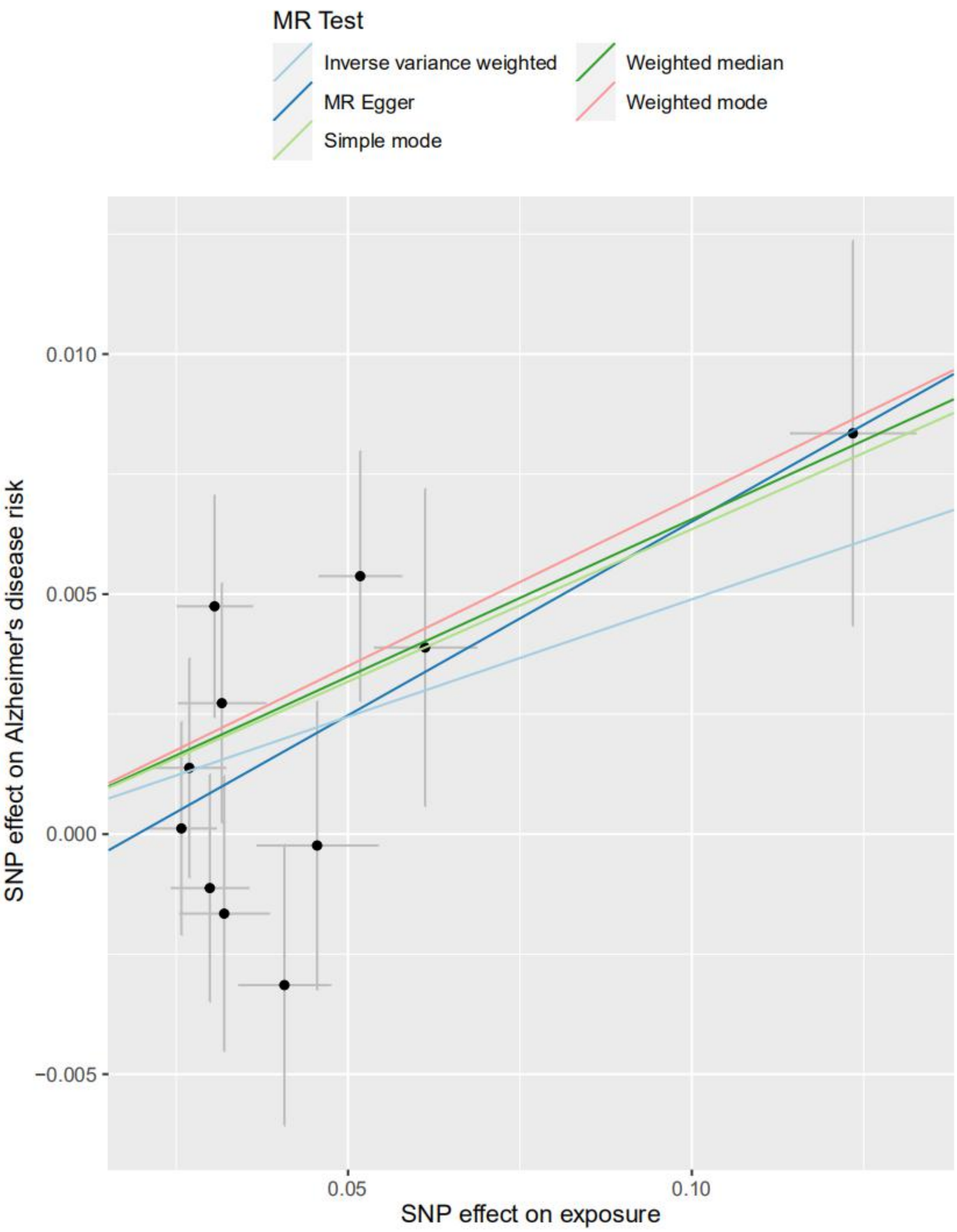


**Figure S5.** Scatterplot of single-nucleotide polymorphism (SNP) associated with surface area of supramarginal and AD (vertical and horizontal lines around each SNP show 95% confidence interval).

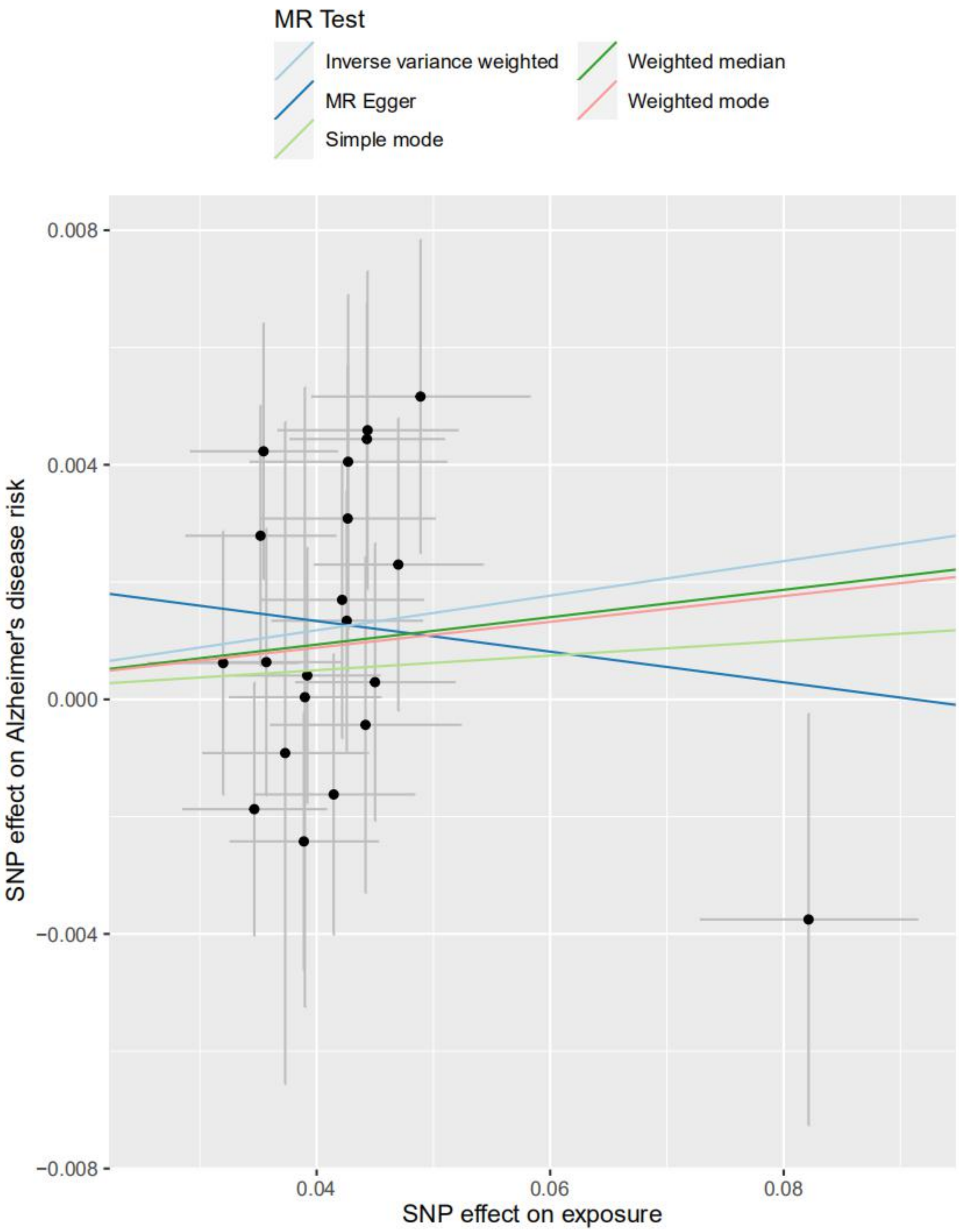


**Figure S6.** Scatterplot of single-nucleotide polymorphism (SNP) associated with surface area of lingual and AD (vertical and horizontal lines around each SNP show 95% confidence interval).

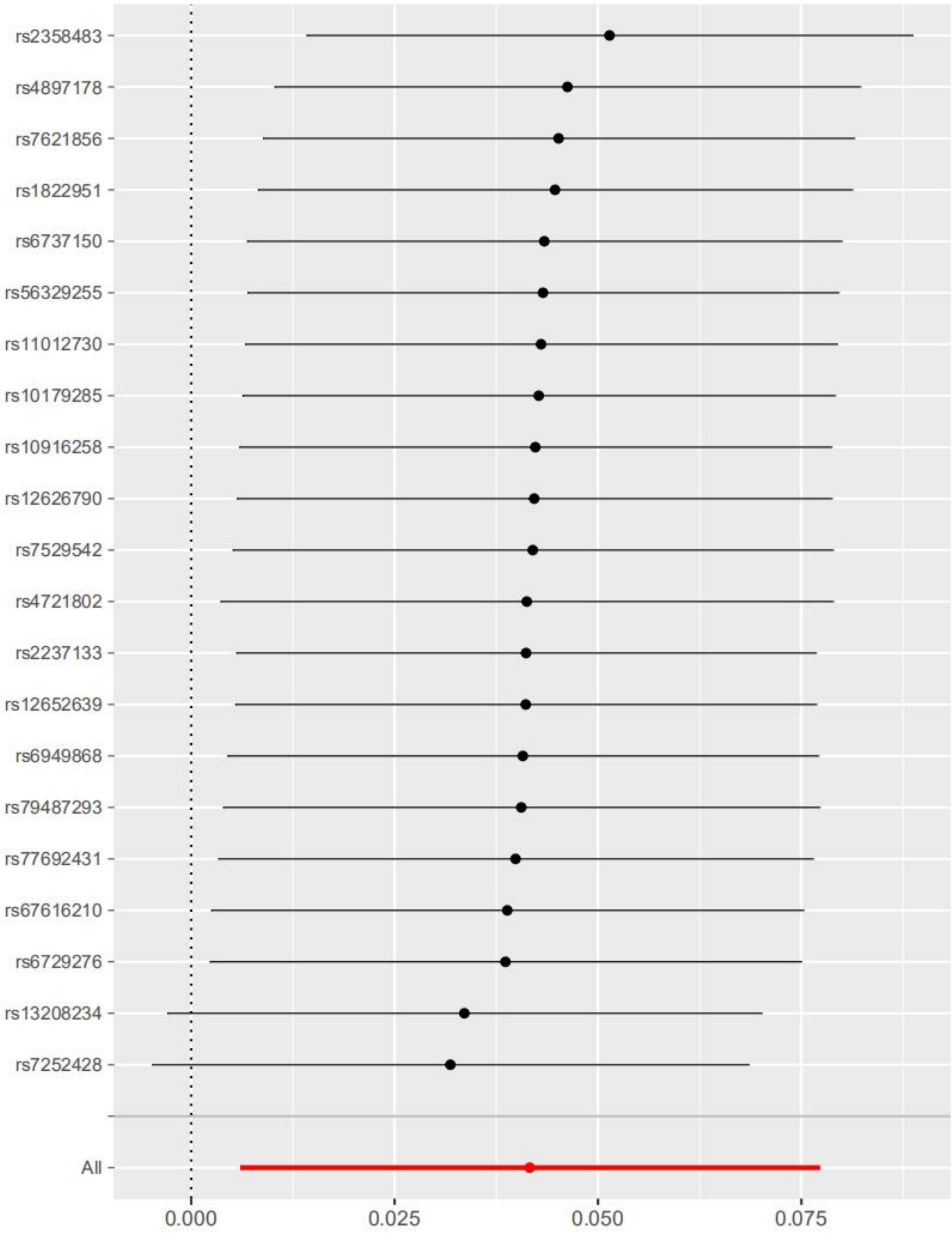


**Figure S7.** Leave-one-out analysis of association between genetically predicted surface area of lateral orbitofrontal and AD.

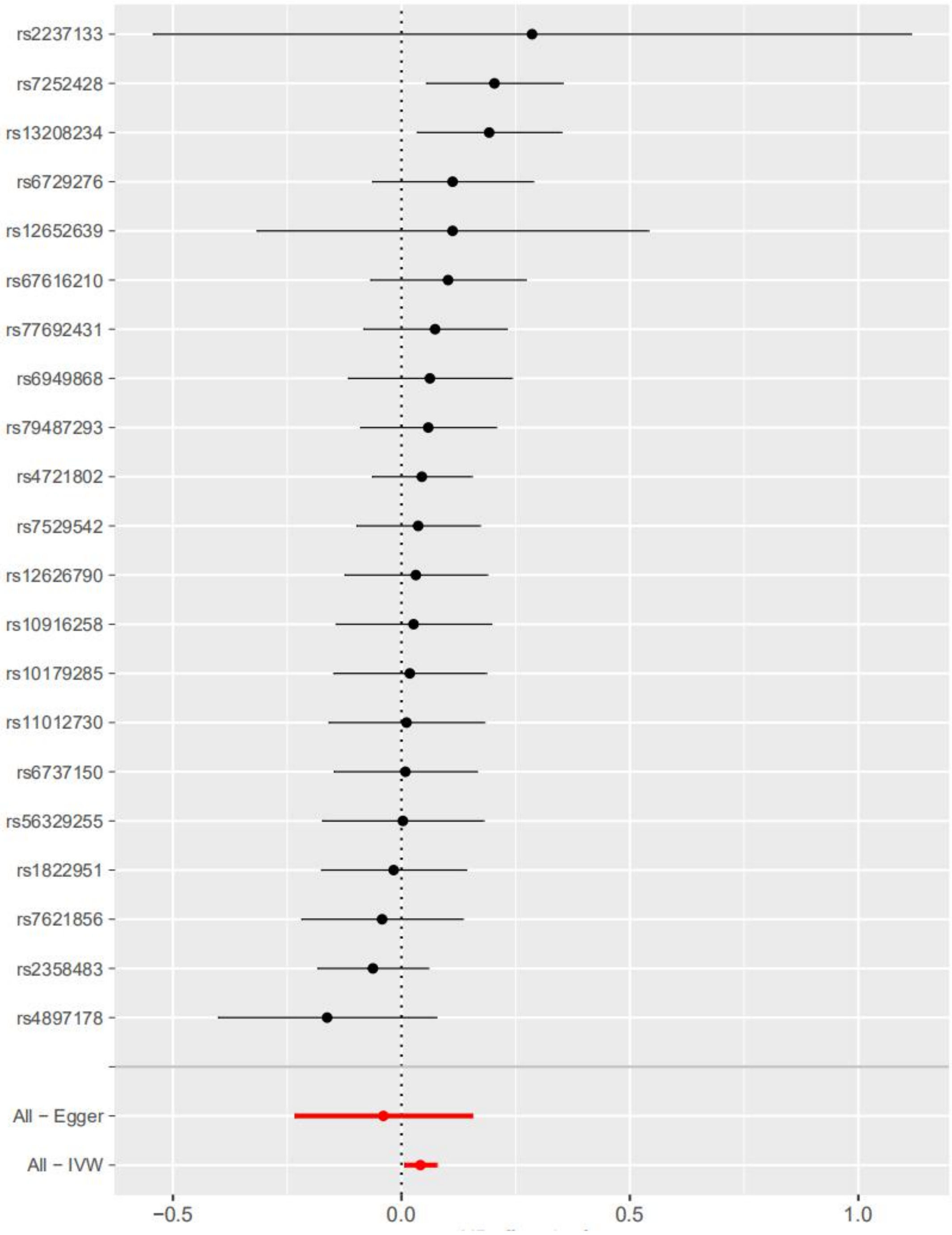


**Figure S8.** Single SNP analysis for individual and combined SNP effects of surface area of lateral orbitofrontal on AD.

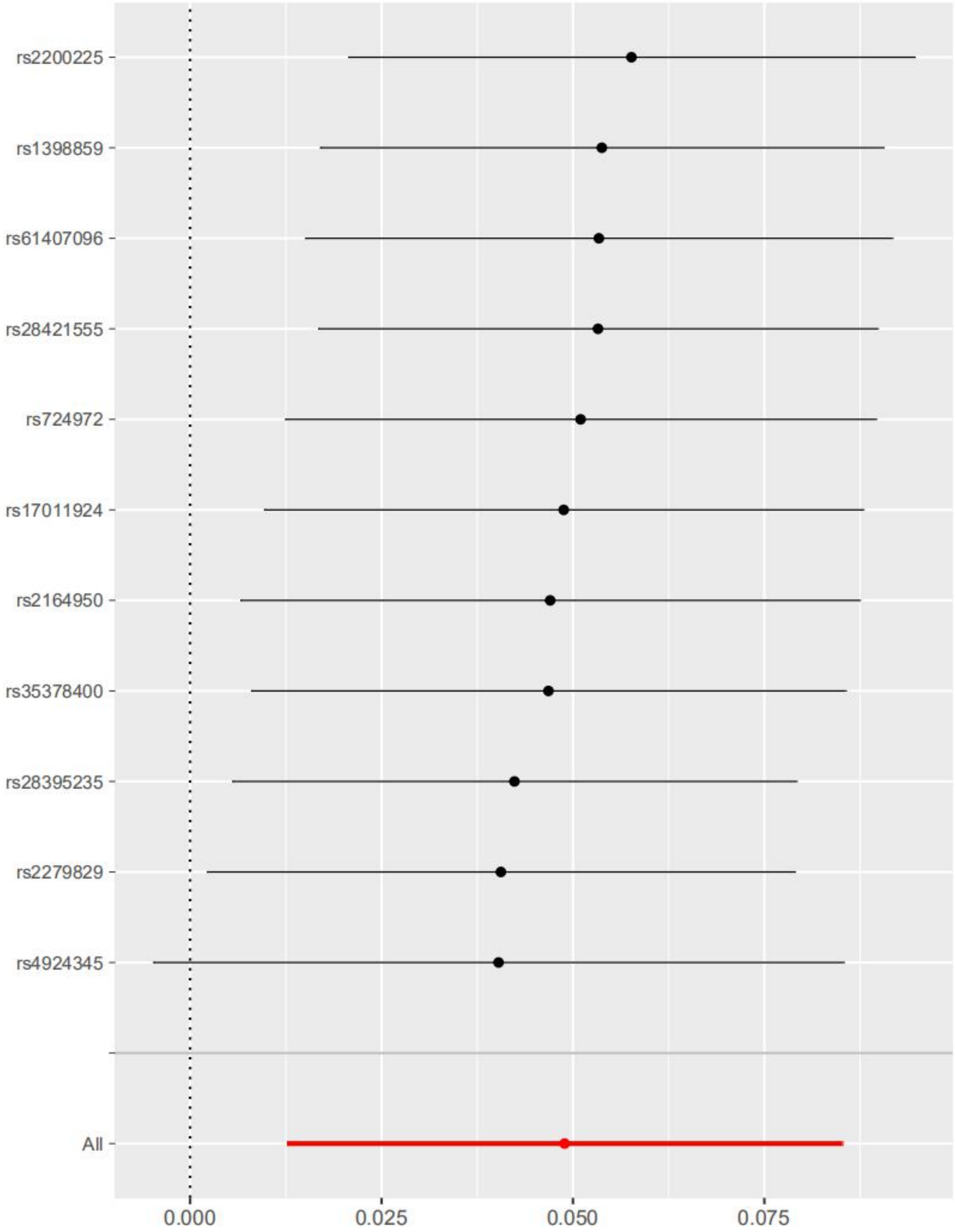


**Figure S9.** Leave-one-out analysis of association between genetically predicted surface area of supramarginal and AD.

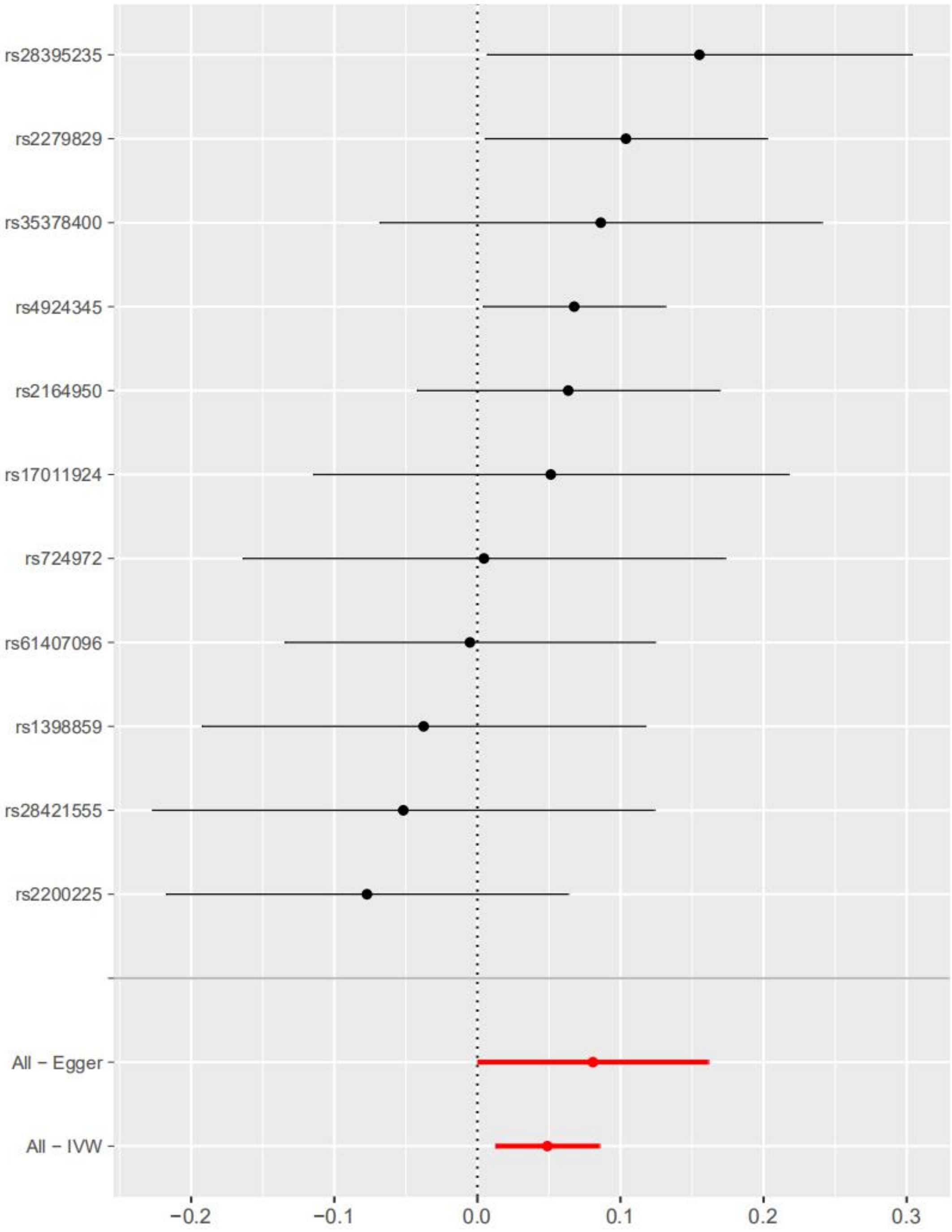


**Figure S10.** Single SNP analysis for individual and combined SNP effects of surface area of supramarginal on AD.

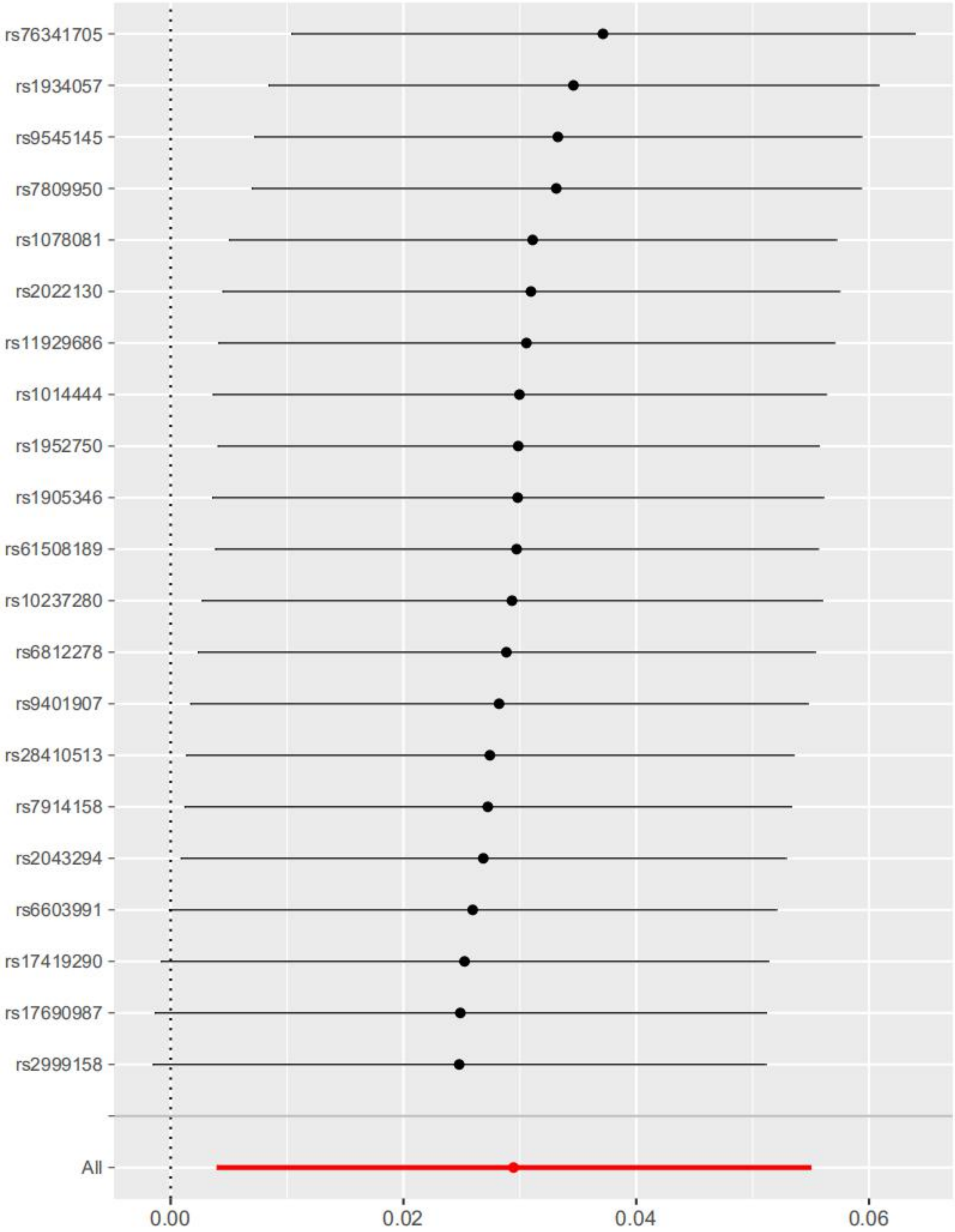


**Figure S11.** Leave-one-out analysis of association between genetically predicted surface area of lingual and AD.

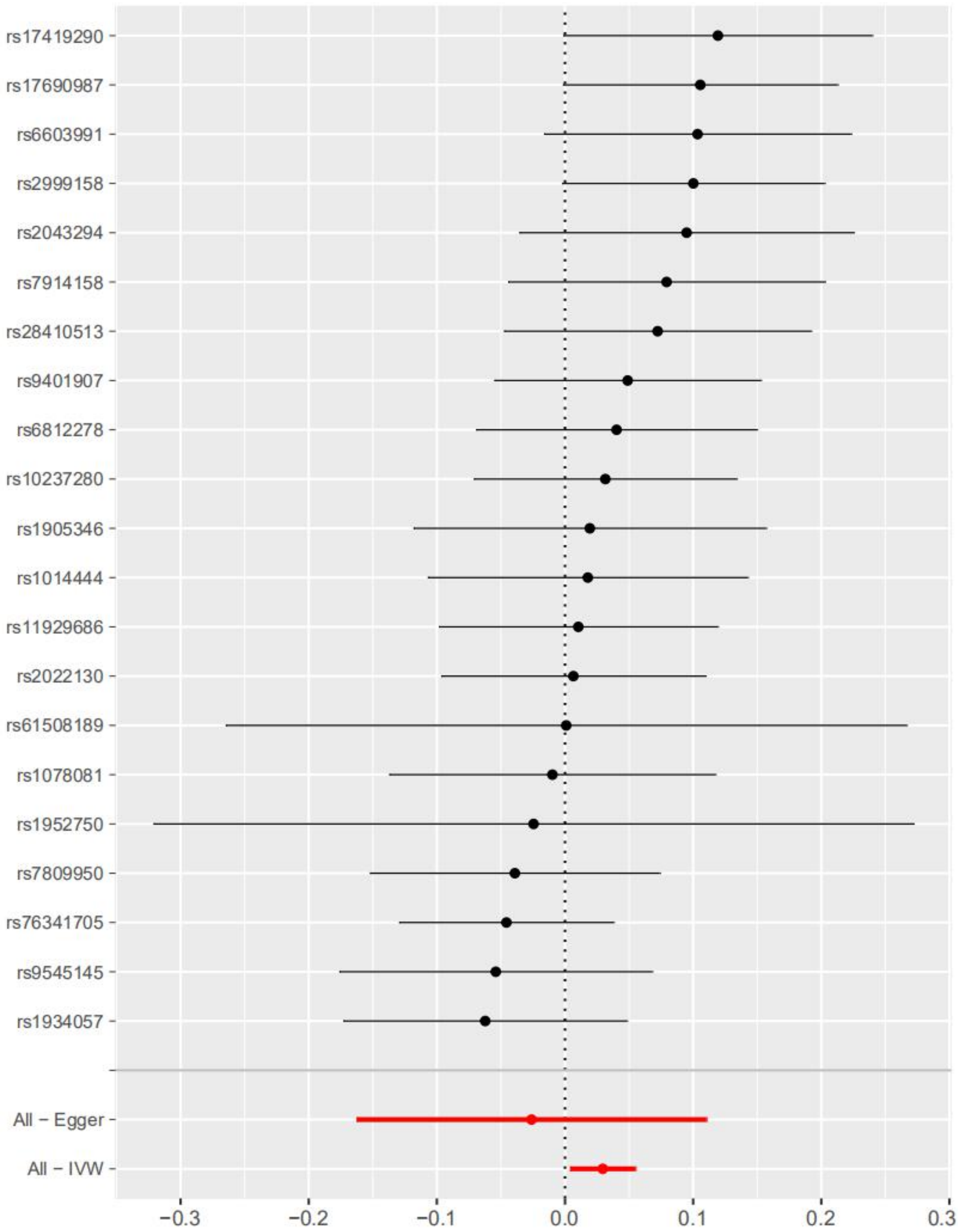


**Figure S12.** Single SNP analysis for individual and combined SNP effects of surface area of lingual on AD.

| Outcome | Lobe | No. of SNPs | OR (95%CI) | P |
|---|---|---|---|---|
| **Global** | | | | |
| Whole cortex | global | 20 | 0.99 (0.96, 1.02) | 0.414 |
| **Regional** | | | | |
| Caudal anterior cingulate | frontal | 5 | 1.02 (0.96, 1.08) | 0.525 |
| Caudal middle frontal | frontal | 5 | 1.06 (0.98, 1.14) | 0.169 |
| Frontal pole | frontal | 6 | 0.96 (0.92, 1.01) | 0.116 |
| Lateral orbitofrontal | frontal | 4 | 0.98 (0.9, 1.06) | 0.554 |
| Medial orbitofrontal | frontal | 3 | 0.94 (0.87, 1.02) | 0.126 |
| Paracentral | frontal | 5 | 1.06 (0.97, 1.16) | 0.175 |
| Pars opercularis | frontal | 9 | 1.02 (0.95, 1.1) | 0.574 |
| Pars orbitalis | frontal | 3 | 0.97 (0.88, 1.07) | 0.556 |
| Pars triangularis | frontal | 4 | 1.02 (0.93, 1.12) | 0.637 |
| Precentral | frontal | 8 | 0.98 (0.91, 1.06) | 0.638 |
| Rostral anterior cingulate | frontal | 7 | 1 (0.93, 1.07) | 0.966 |
| Rostral middle frontal | frontal | 8 | 0.96 (0.86, 1.06) | 0.406 |
| Superior frontal | frontal | 8 | 0.99 (0.92, 1.07) | 0.885 |
| Banks of the superior temporal sulcus | temporal | 5 | 1.02 (0.95, 1.1) | 0.571 |
| Entorhinal | temporal | 4 | 0.96 (0.91, 1.01) | 0.118 |
| Fusiform | temporal | 3 | 1.02 (0.88, 1.18) | 0.774 |
| Inferior temporal | temporal | 2 | 1 (0.78, 1.27) | 0.97 |
| Insula | temporal | 6 | 0.97 (0.91, 1.03) | 0.314 |
| Middle temporal | temporal | 10 | 0.98 (0.93, 1.03) | 0.439 |
| Parahippocampal | temporal | 12 | 1.02 (0.99, 1.06) | 0.134 |
| Superior temporal | temporal | 9 | 1.03 (0.98, 1.09) | 0.227 |
| Temporal pole | temporal | 5 | 1.02 (0.92, 1.12) | 0.736 |
| Transverse temporal | temporal | 4 | 0.99 (0.93, 1.05) | 0.745 |
| Inferior parietal | parietal | 5 | 1.05 (0.96, 1.15) | 0.246 |
| Isthmus cingulate | parietal | 8 | 1.01 (0.97, 1.05) | 0.526 |
| Postcentral | parietal | 15 | 0.99 (0.95, 1.03) | 0.575 |
| Posterior cingulate | parietal | 7 | 0.97 (0.9, 1.05) | 0.44 |
| Precuneus | parietal | 9 | 1.04 (0.97, 1.11) | 0.281 |
| Superior parietal | parietal | 7 | 1.03 (0.96, 1.1) | 0.431 |
| Supramarginal | parietal | 6 | 1.03 (0.92, 1.16) | 0.588 |
| Cuneus | occipital | 5 | 0.93 (0.89, 0.98) | **0.006** |
| Lateral occipital | occipital | 8 | 1 (0.92, 1.1) | 0.956 |
| Lingual | occipital | 8 | 1.03 (0.97, 1.1) | 0.348 |
| Pericalcarine | occipital | 5 | 0.95 (0.88, 1.02) | 0.177 |

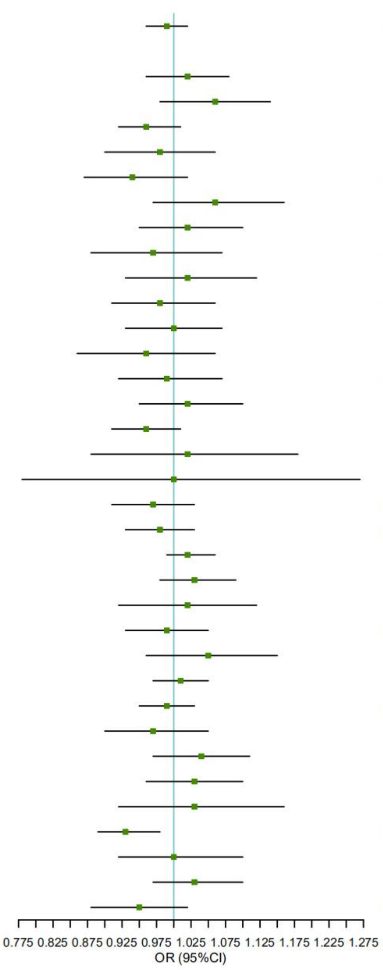


**Figure S13.** Causal effect of genetically predicted cortical thickness on Alzheimer's disease using IVW method.

SNP, single nucleotide polymorphism; OR, odds ratio genetically predicted 1-SD unit increase in the cortical thickness; CI, confidence interval; IVW, inverse variance weighted.

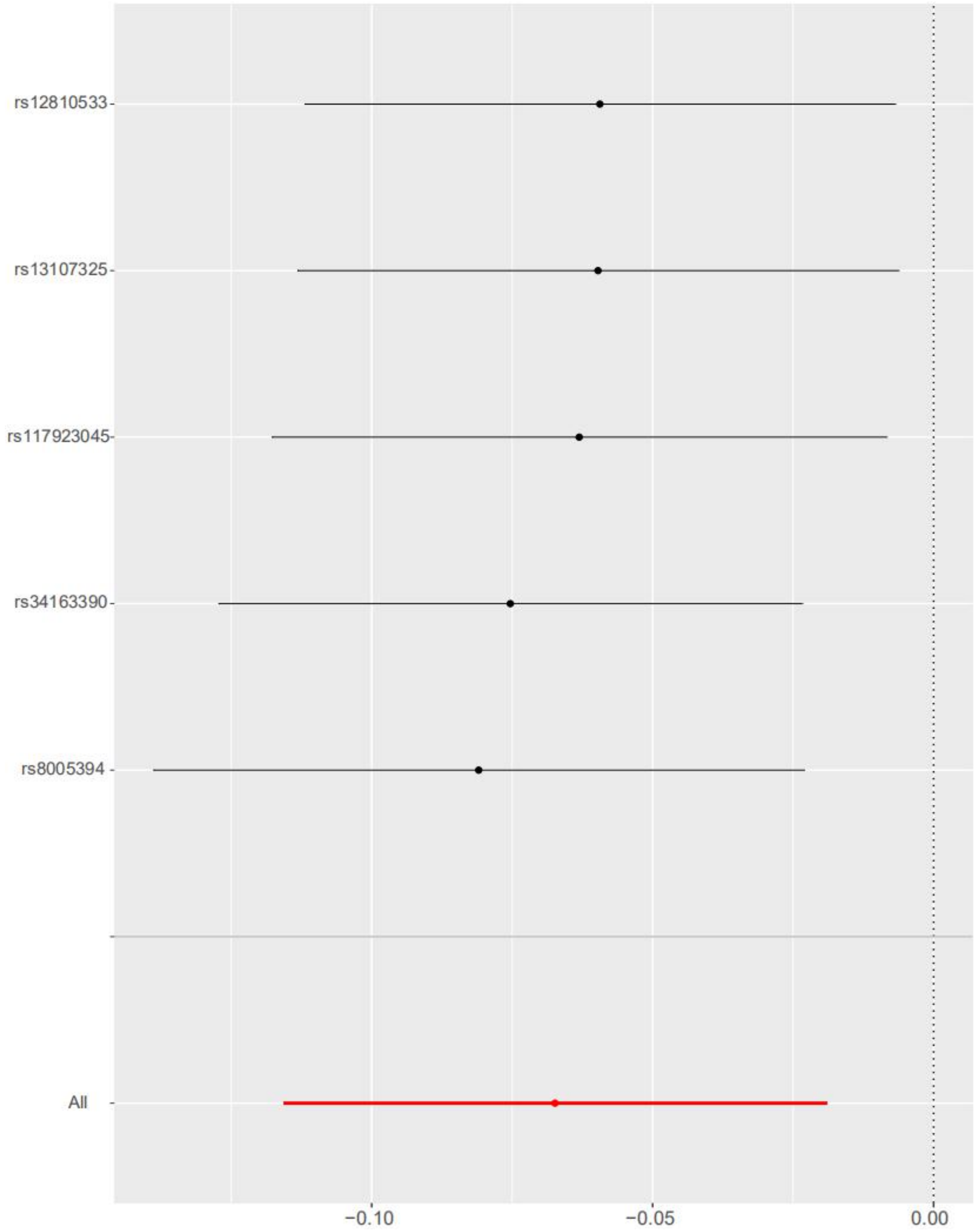


**Figure S14.** Leave-one-out analysis of association between genetically predicted thickness of cuneus and AD.

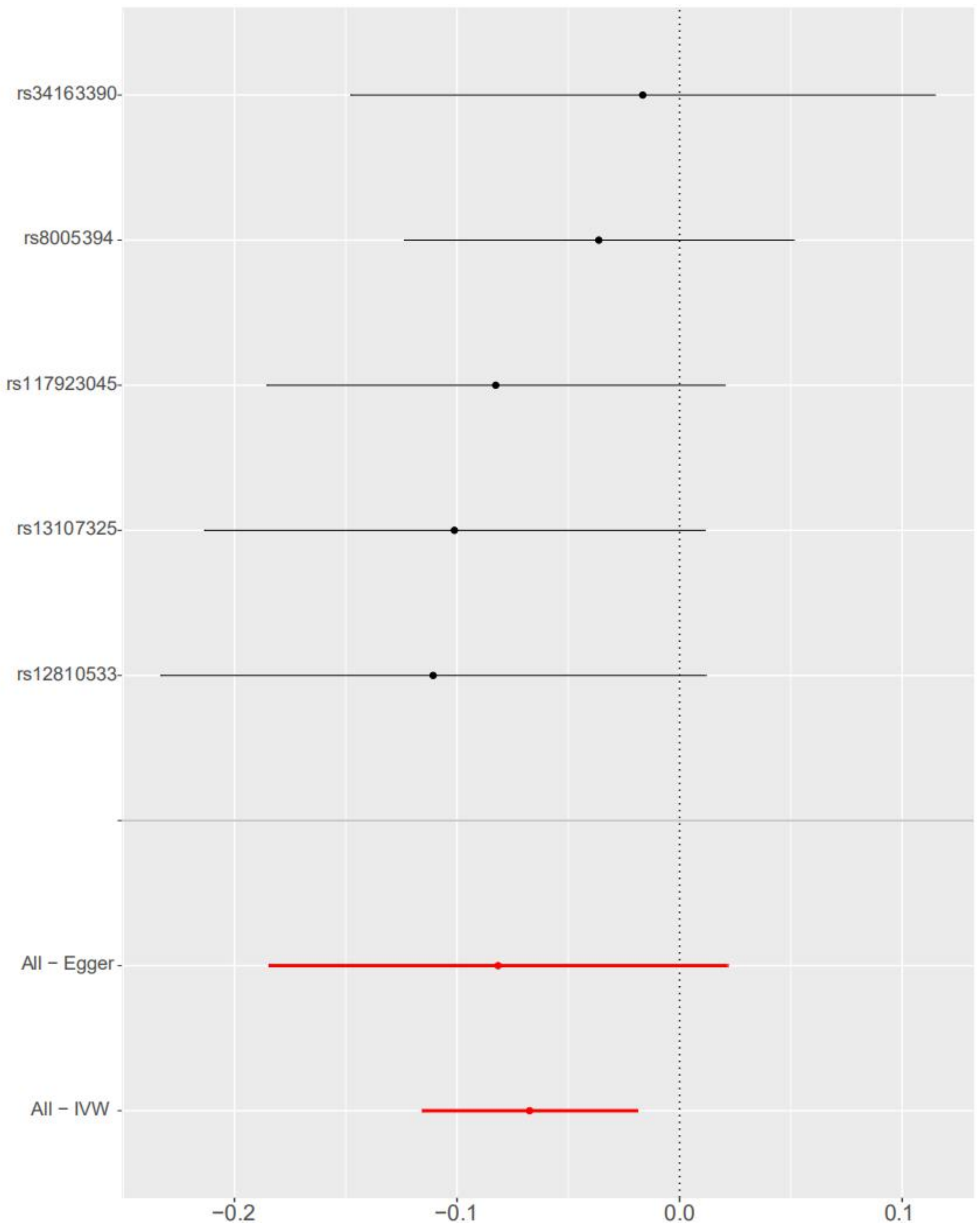


**Figure S15.** Single SNP analysis for individual and combined SNP effects of thickness of cuneus on AD.

| Outcome | Lobe | No. of SNPs | ß (se) | P |
|---|---|---|---|---|
| **Global** | | | | |
| Whole cortex | global | 27 | −789 (984) | 0.423 |
| **Regional** | | | | |
| Caudal anterior cingulate | frontal | 27 | −10.3 (6.39) | 0.106 |
| Caudal middle frontal | frontal | 27 | −8.58 (17.7) | 0.628 |
| Frontal pole | frontal | 27 | 2.08 (1.89) | 0.272 |
| Lateral orbitofrontal | frontal | 27 | −19.5 (11.6) | 0.092 |
| Medial orbitofrontal | frontal | 27 | −5.37 (9.01) | 0.551 |
| Paracentral | frontal | 27 | 4.78 (9.48) | 0.614 |
| Pars opercularis | frontal | 27 | −8.18 (11.1) | 0.463 |
| Pars orbitalis | frontal | 27 | 1.17 (4) | 0.77 |
| Pars triangularis | frontal | 27 | −1.59 (10.3) | 0.878 |
| Precentral | frontal | 27 | −43.4 (21.3) | **0.042** |
| Rostral anterior cingulate | frontal | 27 | −11.7 (6.5) | 0.073 |
| Rostral middle frontal | frontal | 27 | −21.8 (25.4) | 0.391 |
| Superior frontal | frontal | 27 | −2.62 (27.5) | 0.924 |
| Banks of the superior temporal sulcus | temporal | 27 | 0.578 (7.26) | 0.937 |
| Entorhinal | temporal | 27 | −1.59 (3.98) | 0.69 |
| Fusiform | temporal | 27 | −2.91 (16) | 0.855 |
| Inferior temporal | temporal | 27 | 18.7 (19) | 0.324 |
| Insula | temporal | 27 | −1.42 (15.2) | 0.926 |
| Middle temporal | temporal | 27 | 8.13 (20.8) | 0.696 |
| Parahippocampal | temporal | 27 | 8.18 (5.48) | 0.136 |
| Superior temporal | temporal | 27 | −13.6 (17.7) | 0.441 |
| Temporal pole | temporal | 27 | 3.06 (3.03) | 0.313 |
| Transverse temporal | temporal | 27 | 6.01 (3.24) | 0.064 |
| Inferior parietal | parietal | 27 | 28.7 (27.2) | 0.291 |
| Isthmus cingulate | parietal | 27 | −18.5 (7.3) | **0.011** |
| Postcentral | parietal | 27 | −13.5 (18.2) | 0.461 |
| Posterior cingulate | parietal | 27 | −0.083 (8.05) | 0.992 |
| Precuneus | parietal | 27 | −9.91 (18.2) | 0.586 |
| Superior parietal | parietal | 27 | 25.8 (26.1) | 0.323 |
| Supramarginal | parietal | 27 | 24.1 (20.9) | 0.248 |
| Cuneus | occipital | 27 | 23.4 (11.8) | **0.047** |
| Lateral occipital | occipital | 27 | −4.41 (24) | 0.854 |
| Lingual | occipital | 27 | 13.3 (20.3) | 0.513 |
| Pericalcarine | occipital | 27 | 27.3 (12.8) | **0.033** |

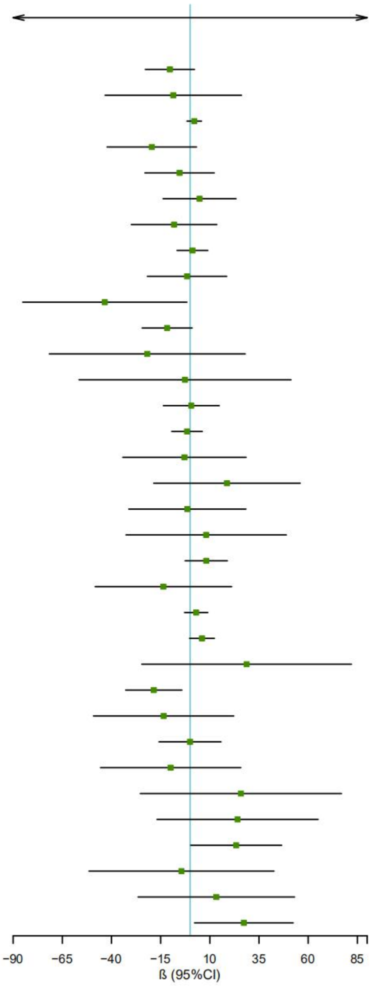


**Figure S16.** Causal effect of genetically predicted Alzheimer's disease on cortical surface area using IVW method.

SNP, single nucleotide polymorphism; β, changes in cortical thickness in population with disease compared with controls; se, standard error; CI, confidence interval; IVW, inverse variance weighted.

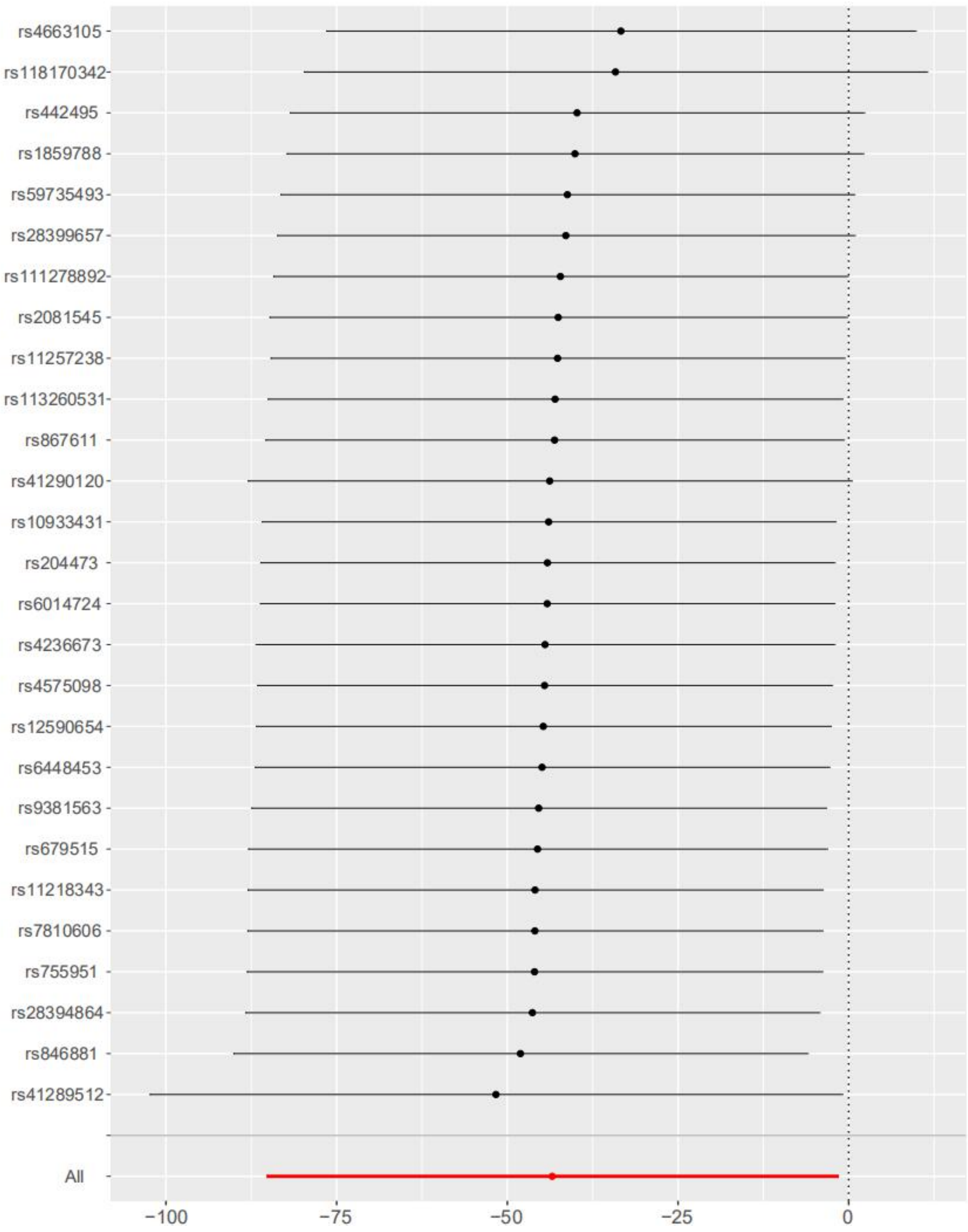


**Figure S17.** Leave-one-out analysis of association between genetically predicted AD and surface area of precentral.

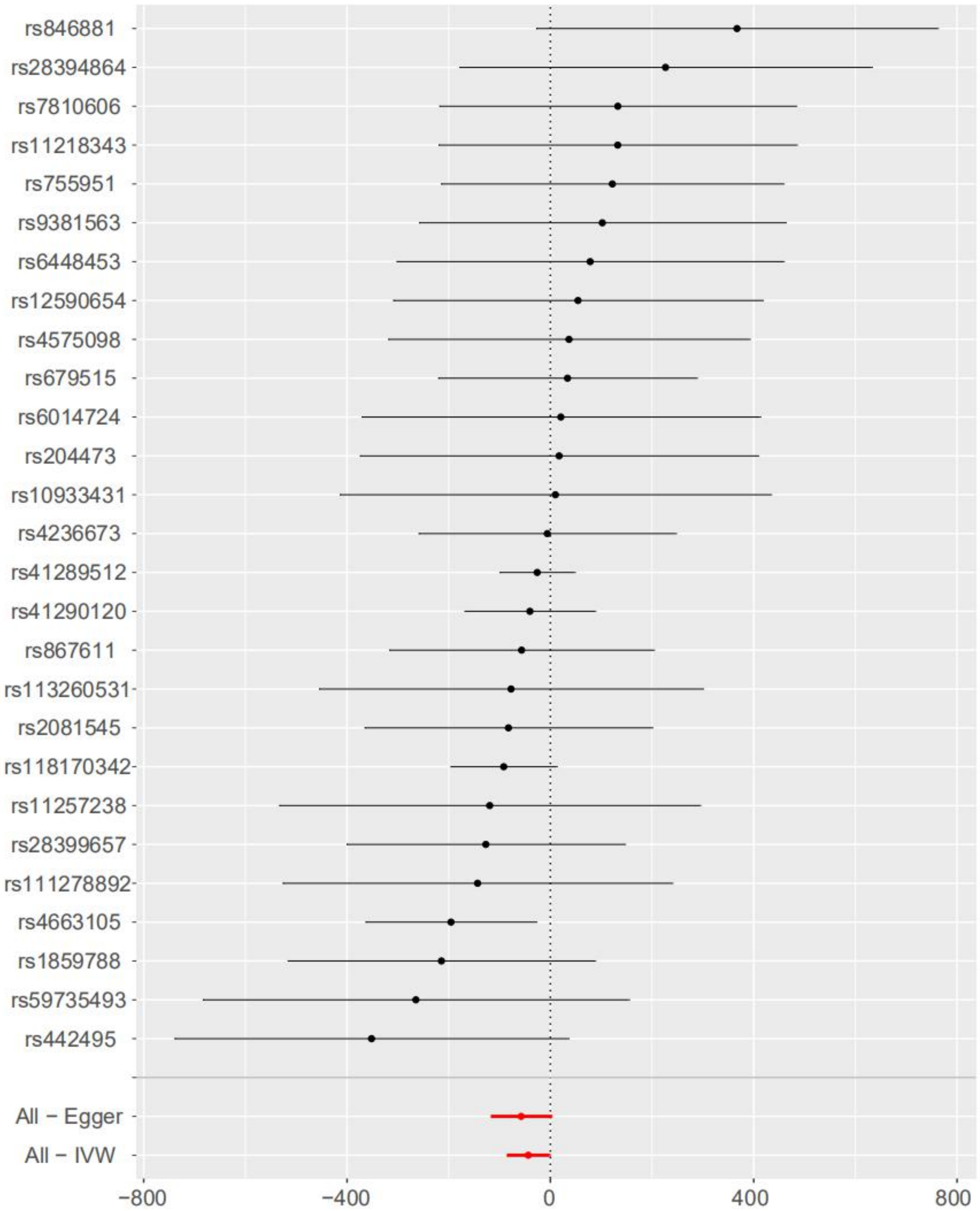


**Figure S18.** Single SNP analysis for individual and combined SNP effects of AD on surface area of precentral.

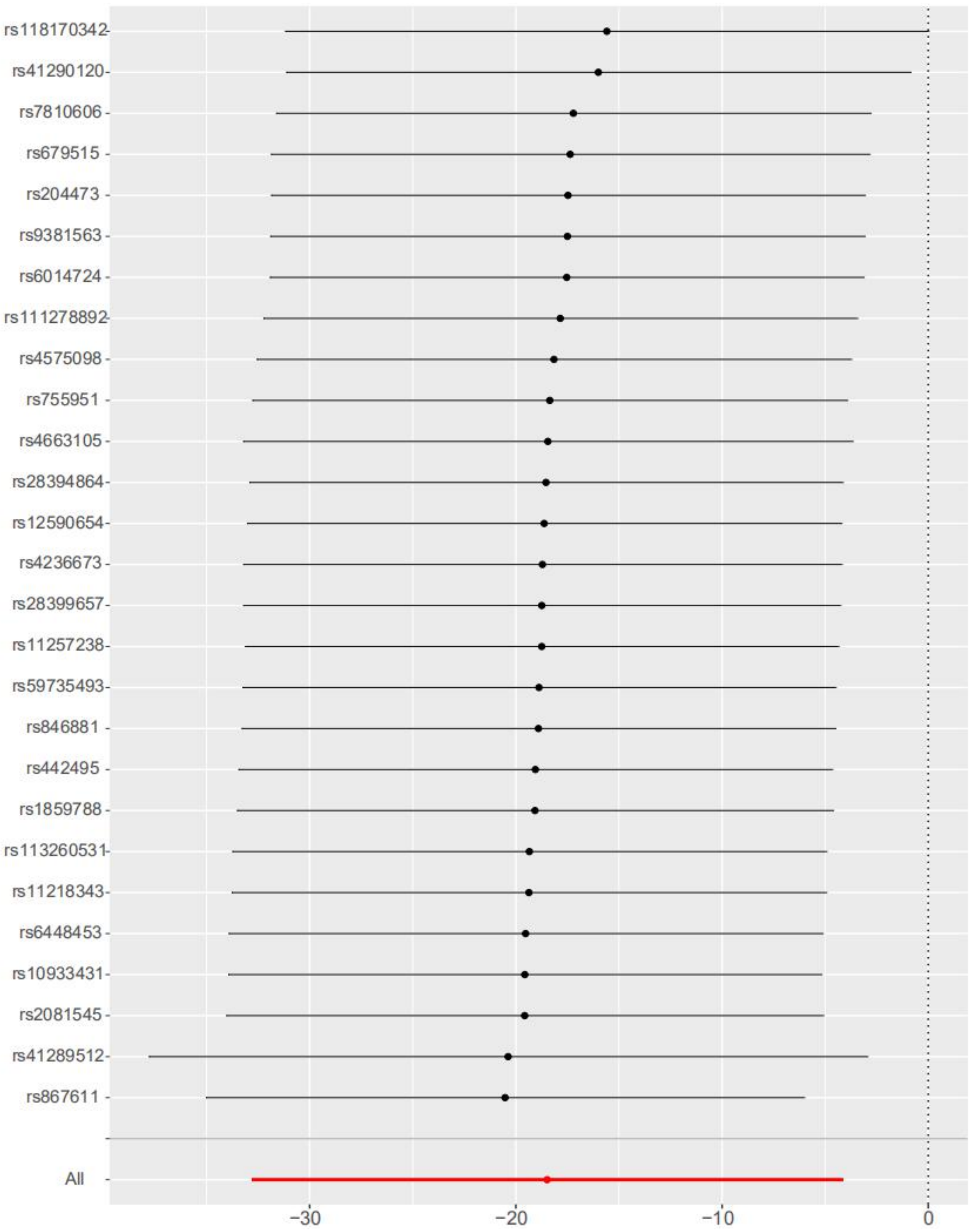


**Figure S19.** Leave-one-out analysis of association between genetically predicted AD and surface area of isthmus cingulate.

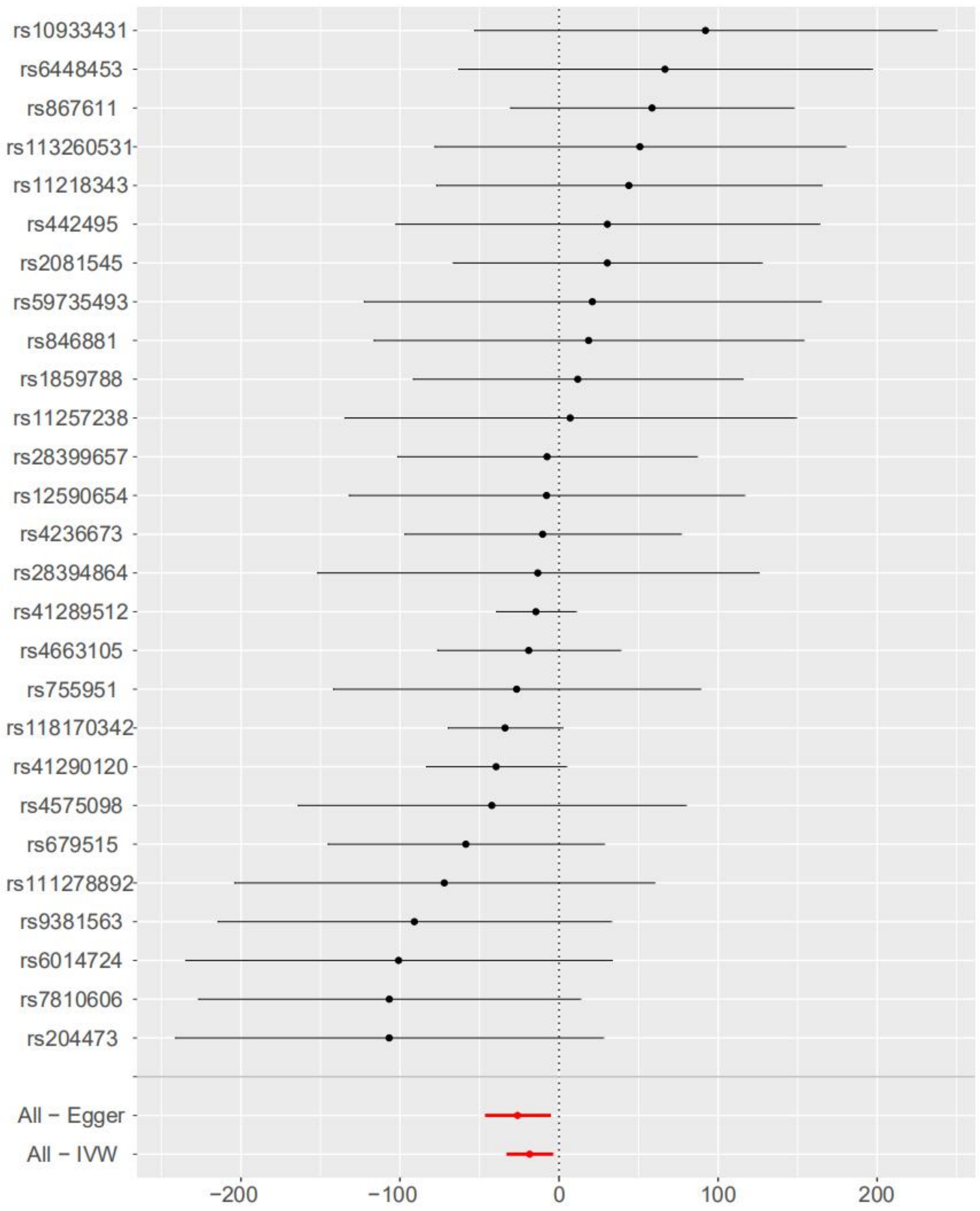


**Figure S20.** Single SNP analysis for individual and combined SNP effects of AD on surface area of isthmus cingulate.

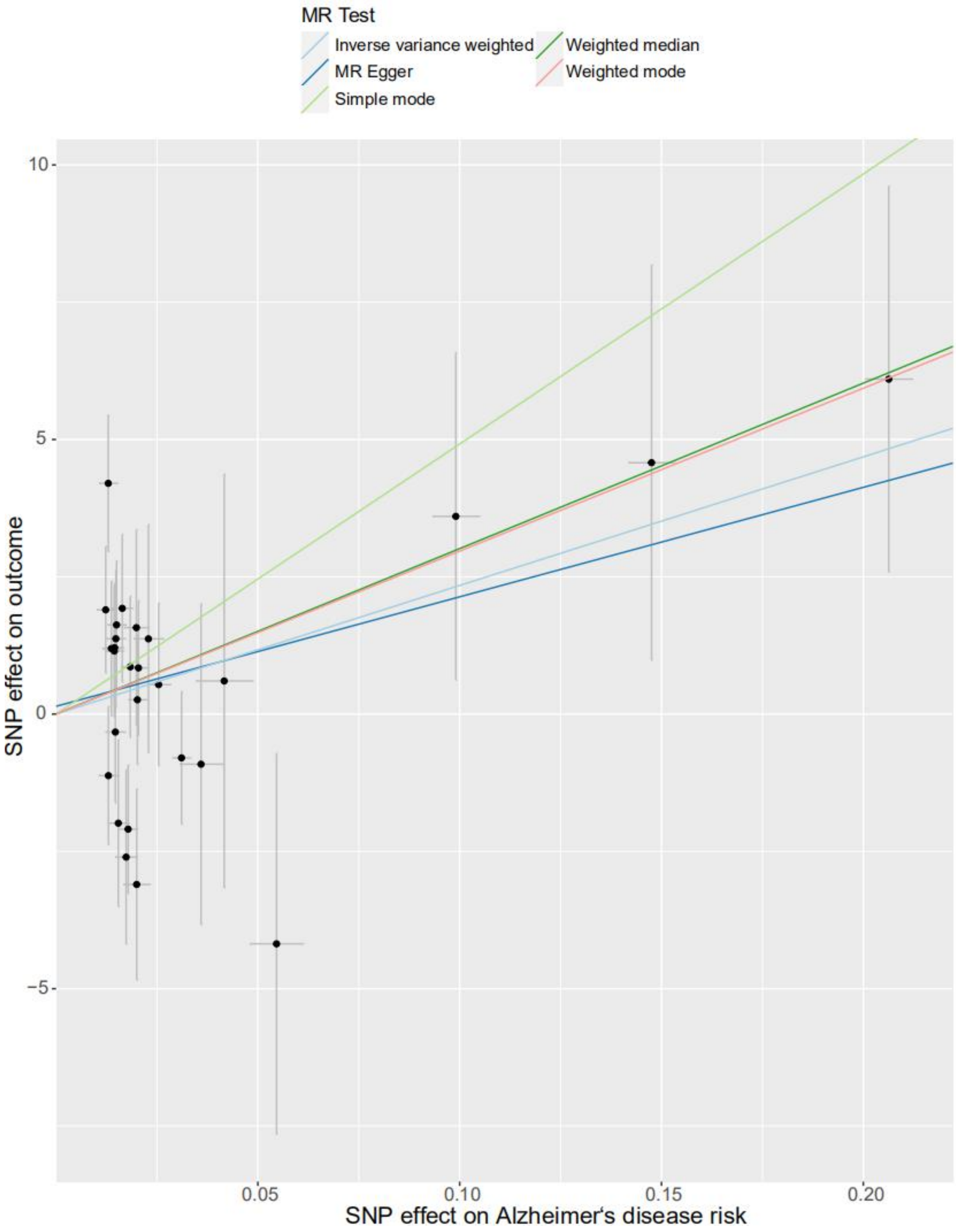


**Figure S21.** Scatterplot of single-nucleotide polymorphism (SNP) associated with AD and surface area of cuneus (vertical and horizontal lines around each SNP show 95% confidence interval).

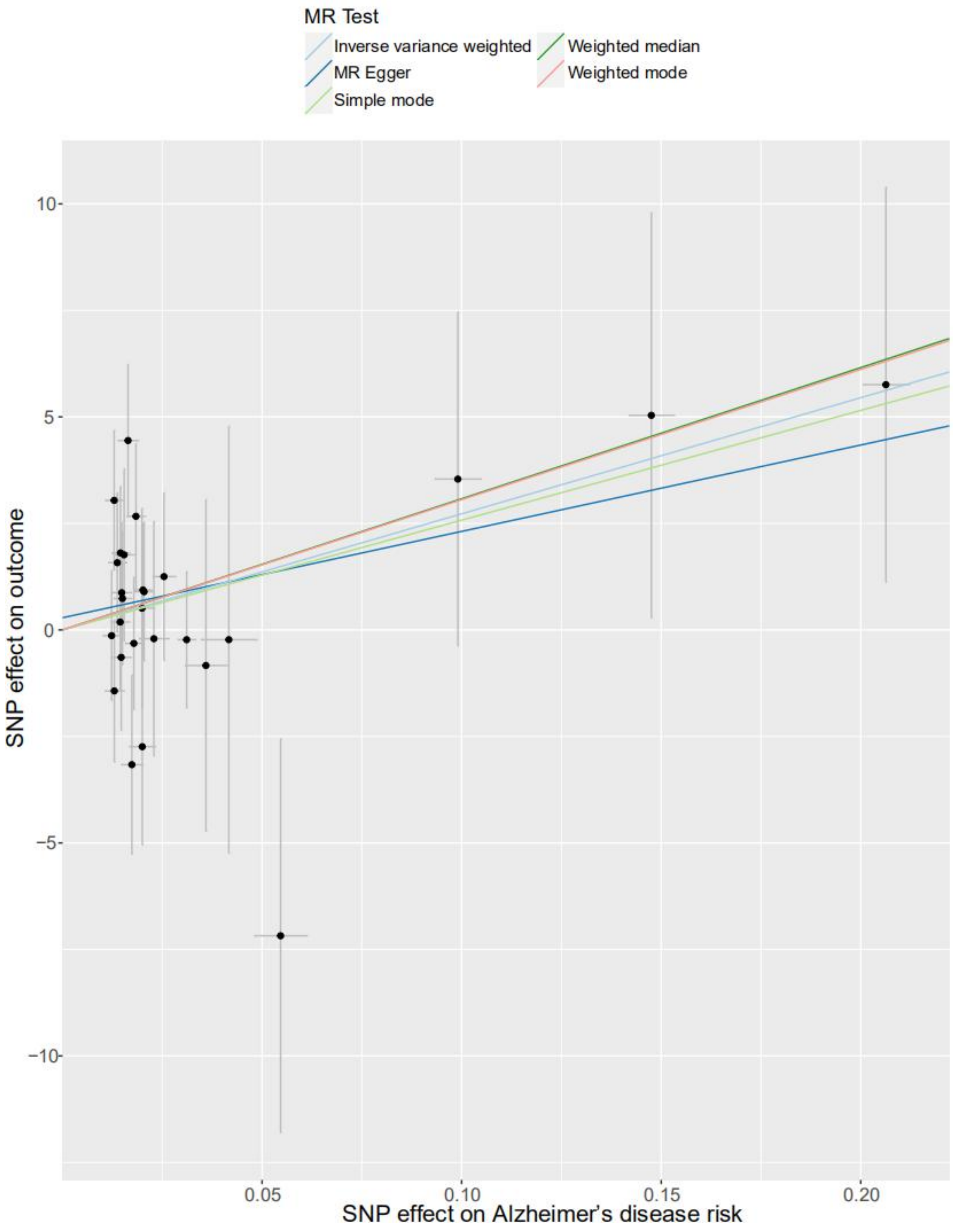


**Figure S22.** Scatterplot of single-nucleotide polymorphism (SNP) associated with AD and surface area of pericalcarine (vertical and horizontal lines around each SNP show 95% confidence interval).

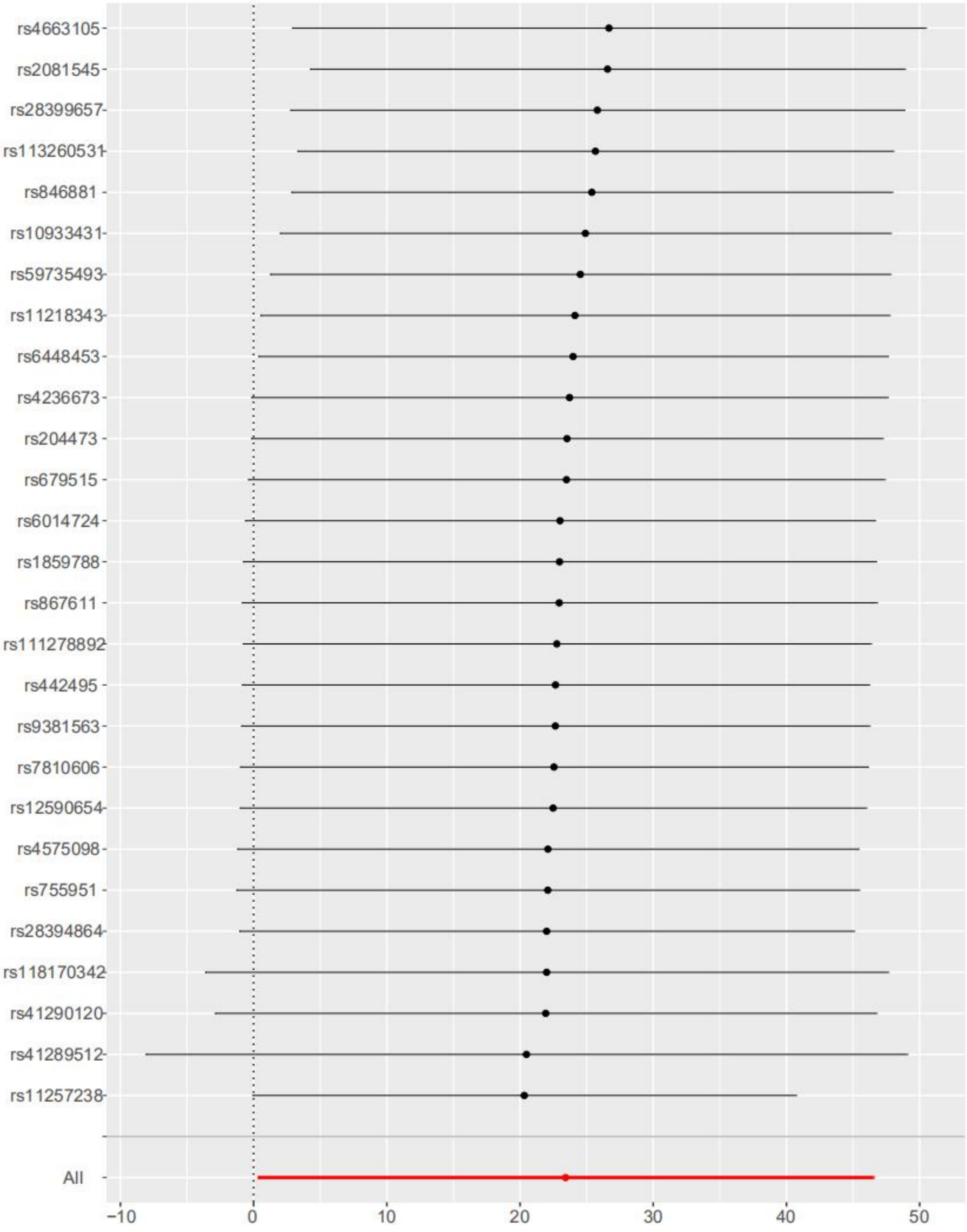


**Figure S23.** Leave-one-out analysis of association between genetically predicted AD and surface area of cuneus.

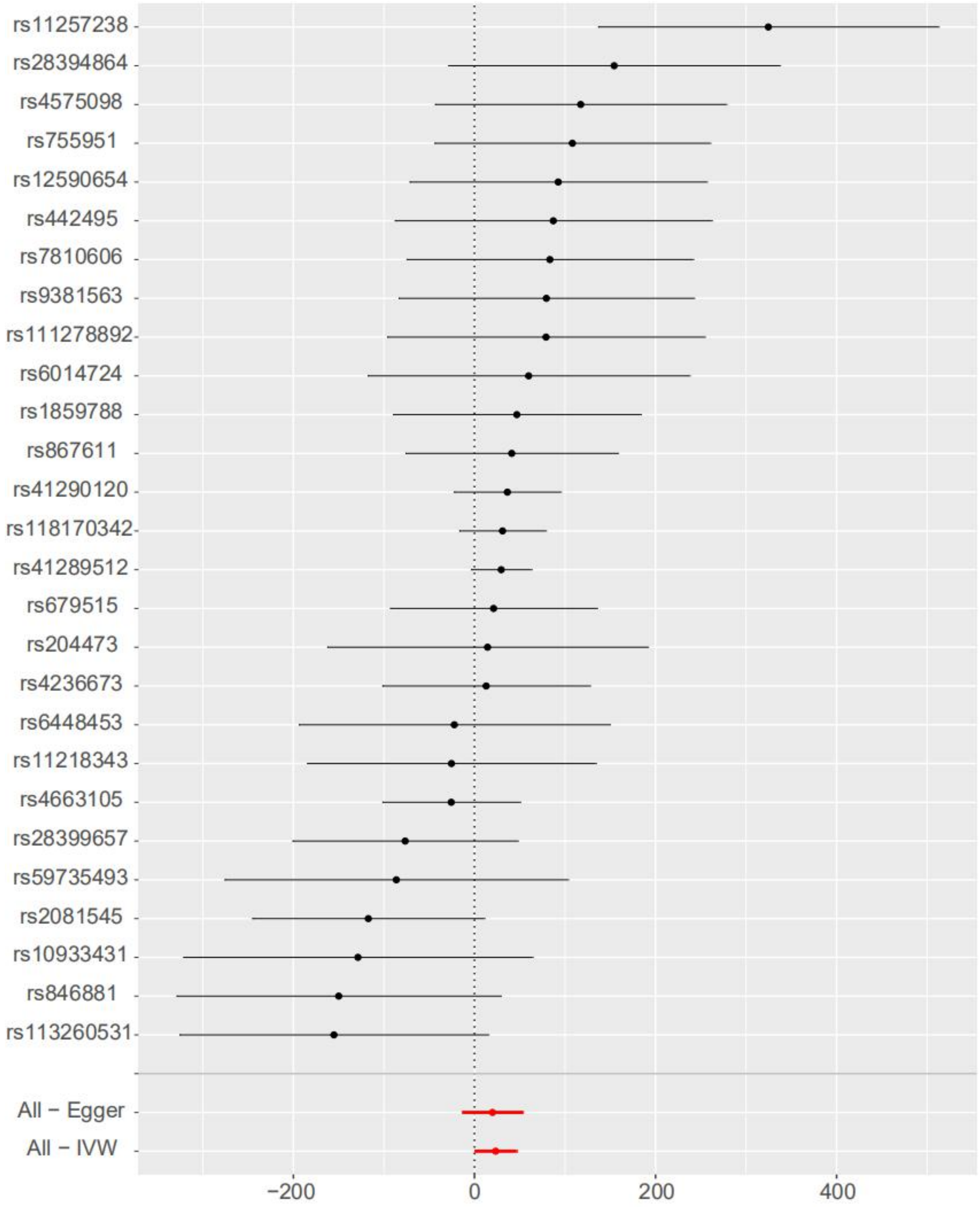


**Figure S24.** Single SNP analysis for individual and combined SNP effects of AD on surface area of cuneus.

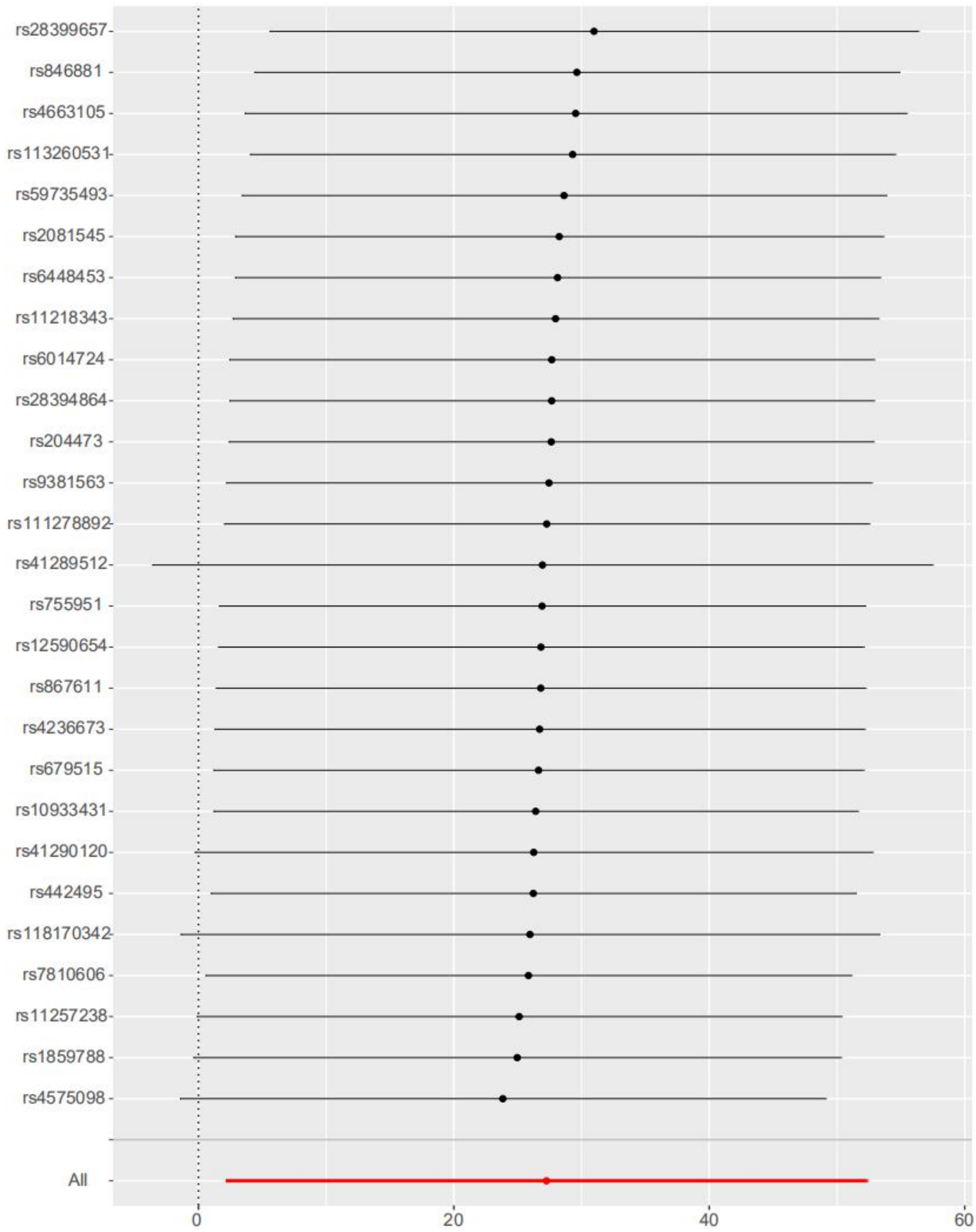


**Figure S25.** Leave-one-out analysis of association between genetically predicted AD and surface area of pericalcarine.

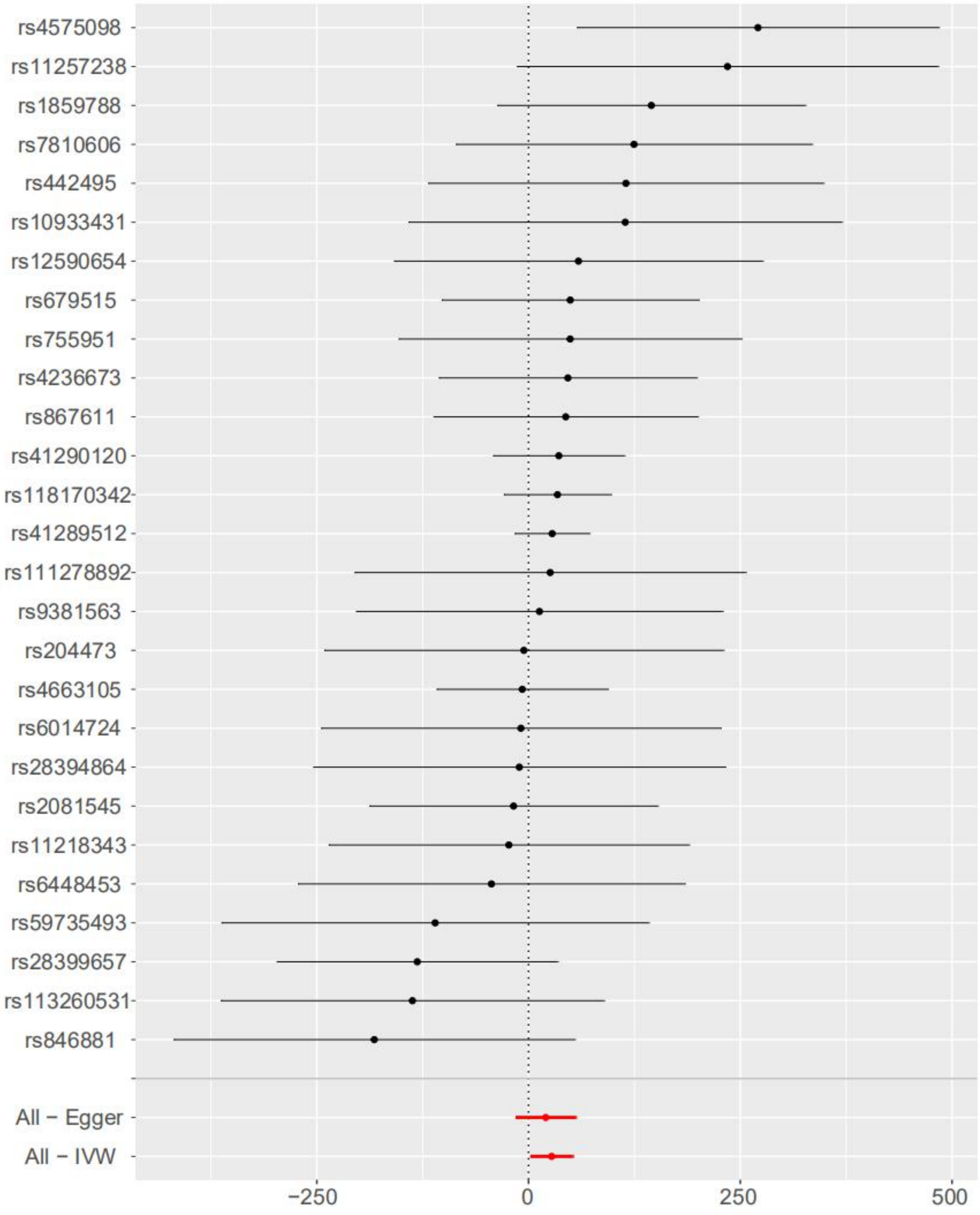


**Figure S26.** Single SNP analysis for individual and combined SNP effects of AD on surface area of pericalcarine.

| Outcome | Lobe | No. of SNPs | ß (se) | P |
|---|---|---|---|---|
| **Global** | | | | |
| Whole cortex | global | 27 | 0.003 (0.006) | 0.634 |
| **Regional** | | | | |
| Caudal anterior cingulate | frontal | 27 | −0.018 (0.013) | 0.174 |
| Caudal middle frontal | frontal | 27 | −0.01 (0.006) | 0.126 |
| Frontal pole | frontal | 27 | 0.002 (0.014) | 0.908 |
| Lateral orbitofrontal | frontal | 27 | 0.002 (0.008) | 0.839 |
| Medial orbitofrontal | frontal | 27 | −0.006 (0.009) | 0.521 |
| Paracentral | frontal | 27 | 0.006 (0.008) | 0.449 |
| Pars opercularis | frontal | 27 | −0.004 (0.007) | 0.586 |
| Pars orbitalis | frontal | 27 | −0.007 (0.01) | 0.438 |
| Pars triangularis | frontal | 27 | −0.002 (0.007) | 0.718 |
| Precentral | frontal | 27 | 0.002 (0.006) | 0.795 |
| Rostral anterior cingulate | frontal | 27 | 0.008 (0.013) | 0.503 |
| Rostral middle frontal | frontal | 27 | −0.004 (0.005) | 0.419 |
| Superior frontal | frontal | 27 | −0.004 (0.006) | 0.431 |
| Banks of the superior temporal sulcus | temporal | 27 | −0.001 (0.008) | 0.938 |
| Entorhinal | temporal | 27 | 0.01 (0.025) | 0.677 |
| Fusiform | temporal | 27 | 0 (0.007) | 0.995 |
| Inferior temporal | temporal | 27 | 0.002 (0.01) | 0.872 |
| Insula | temporal | 27 | −0.006 (0.008) | 0.474 |
| Middle temporal | temporal | 27 | 0.003 (0.007) | 0.634 |
| Parahippocampal | temporal | 27 | −0.032 (0.022) | 0.141 |
| Superior temporal | temporal | 27 | −0.004 (0.007) | 0.592 |
| Temporal pole | temporal | 27 | 0.012 (0.018) | 0.512 |
| Transverse temporal | temporal | 27 | 0.007 (0.012) | 0.543 |
| Inferior parietal | parietal | 27 | −0.004 (0.006) | 0.481 |
| Isthmus cingulate | parietal | 27 | −0.012 (0.011) | 0.275 |
| Postcentral | parietal | 27 | 0.006 (0.007) | 0.363 |
| Posterior cingulate | parietal | 27 | −0.003 (0.009) | 0.749 |
| Precuneus | parietal | 27 | 0.006 (0.005) | 0.25 |
| Superior parietal | parietal | 27 | 0.006 (0.006) | 0.352 |
| Supramarginal | parietal | 27 | 0 (0.005) | 0.948 |
| Cuneus | occipital | 27 | 0.014 (0.007) | **0.05** |
| Lateral occipital | occipital | 27 | 0.018 (0.006) | **0.002** |
| Lingual | occipital | 27 | 0.012 (0.006) | **0.046** |
| Pericalcarine | occipital | 27 | 0.009 (0.007) | 0.247 |

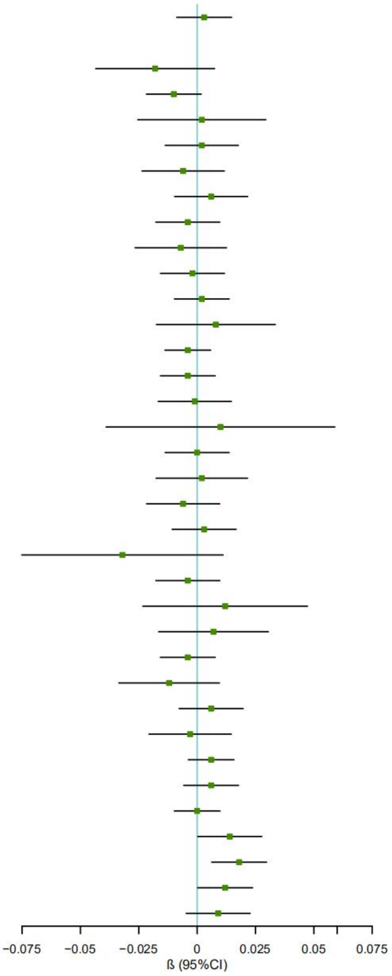


**Figure S27.** Causal effect of genetically predicted Alzheimer's disease on cortical thickness using IVW method.
SNP, single nucleotide polymorphism; β, changes in cortical thickness in population with disease compared with controls; se, standard error; CI, confidence interval; IVW, inverse variance weighted.

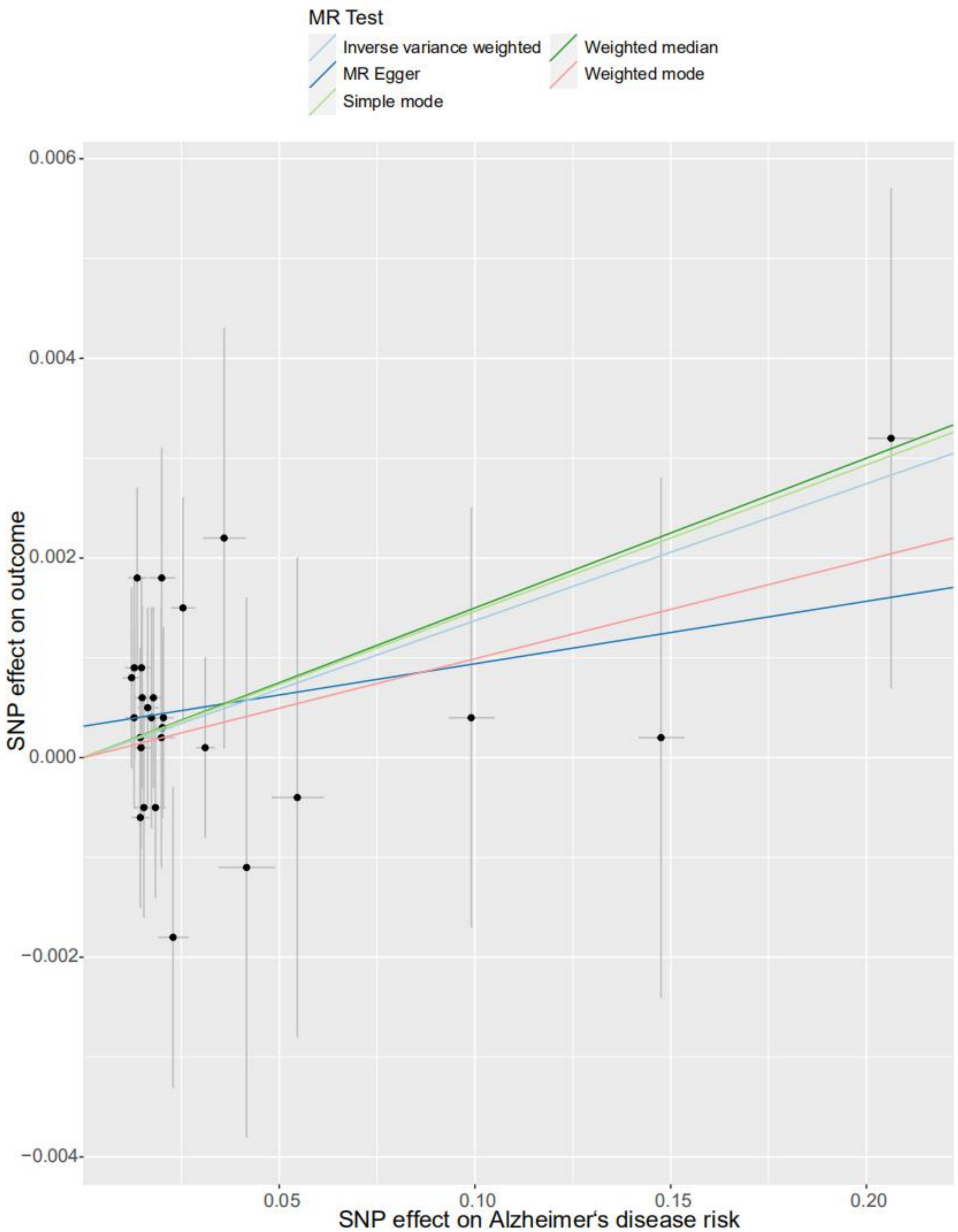


**Figure S28.** Scatterplot of single-nucleotide polymorphism (SNP) associated with AD and thickness of cuneus (vertical and horizontal lines around each SNP show 95% confidence interval).

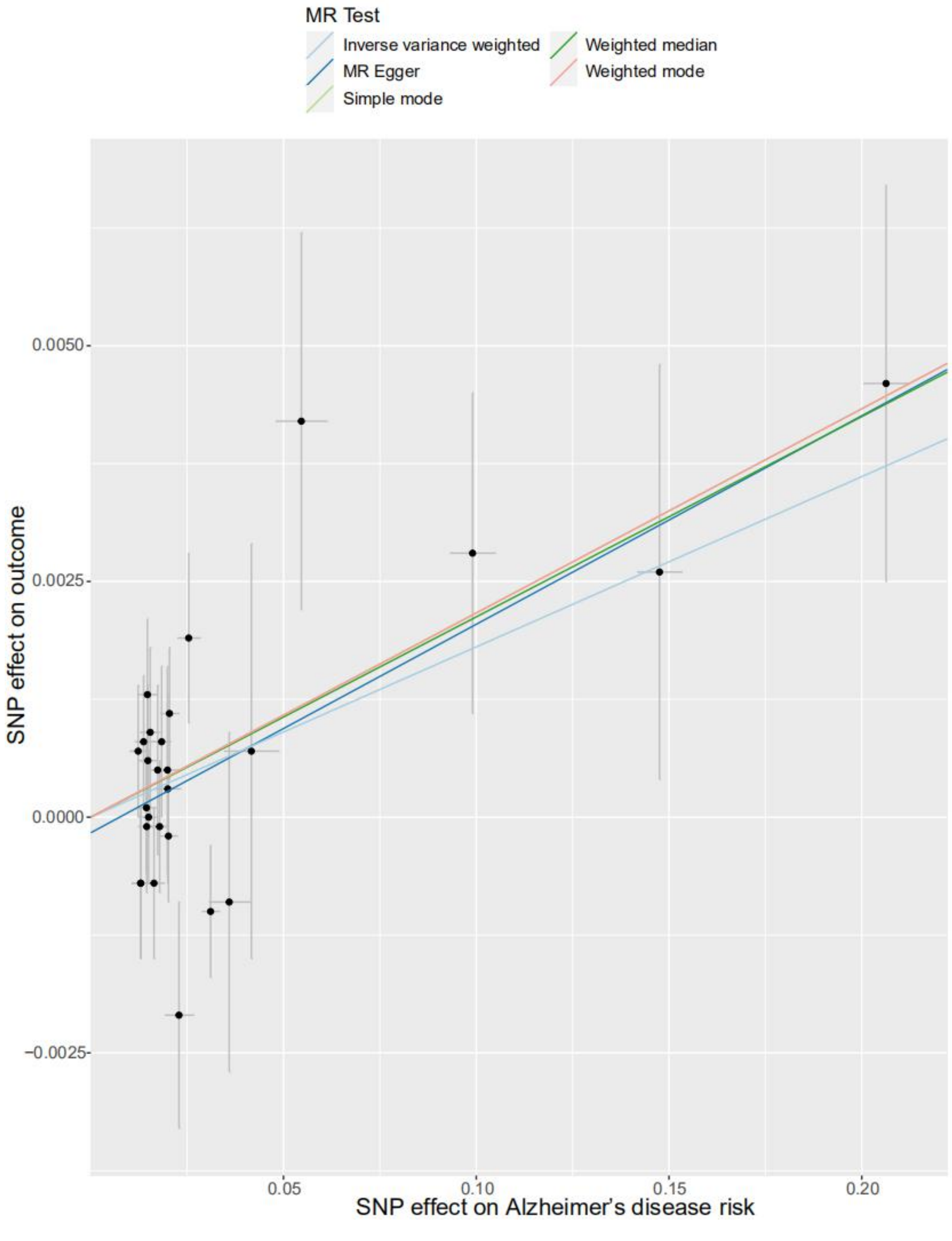


**Figure S29.** Scatterplot of single-nucleotide polymorphism (SNP) associated with AD and thickness of lateral occipital (vertical and horizontal lines around each SNP show 95% confidence interval).

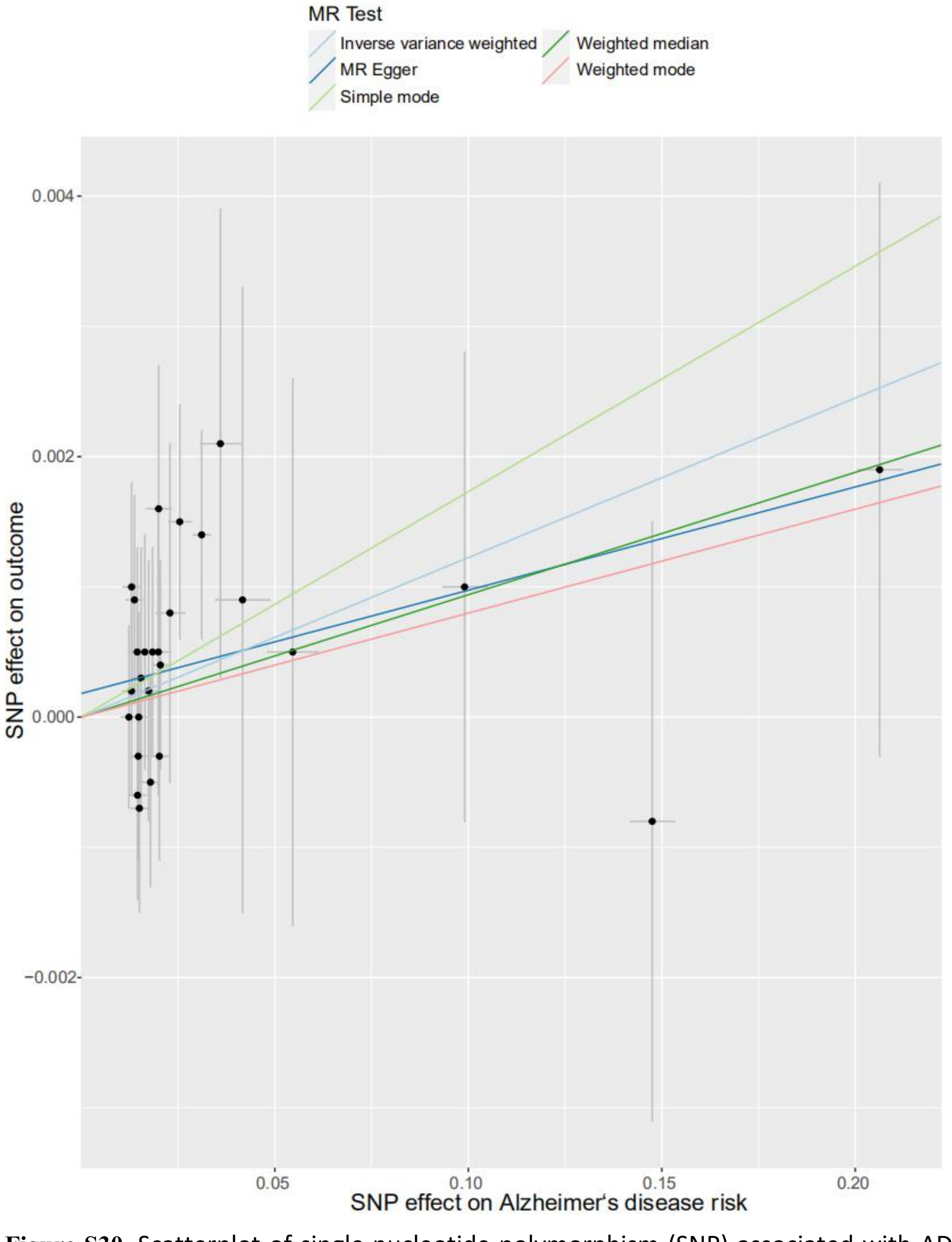


**Figure S30.** Scatterplot of single-nucleotide polymorphism (SNP) associated with AD and thickness of lingual (vertical and horizontal lines around each SNP show 95% confidence interval).

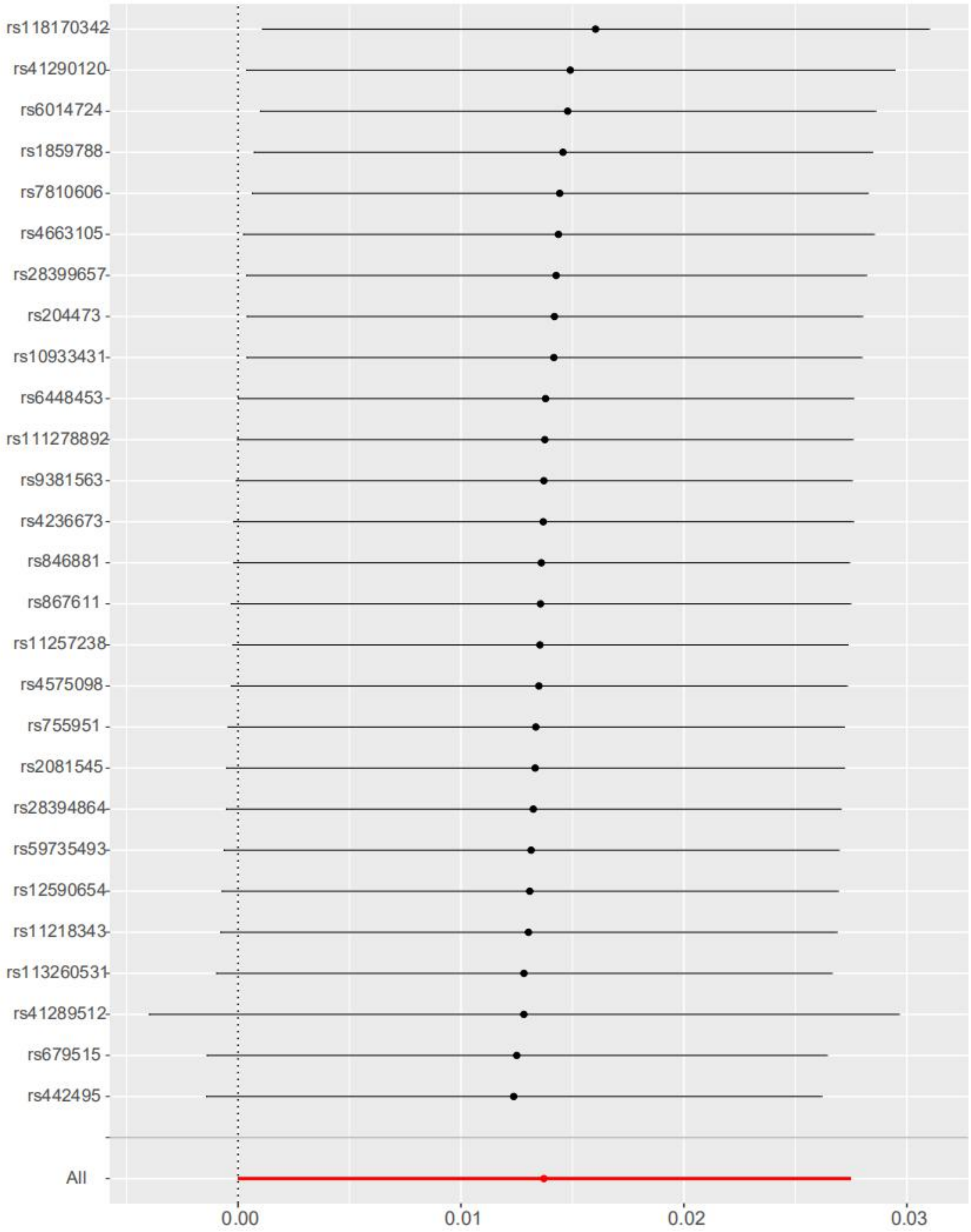


**Figure S31.** Leave-one-out analysis of association between genetically predicted AD and thickness of cuneus.

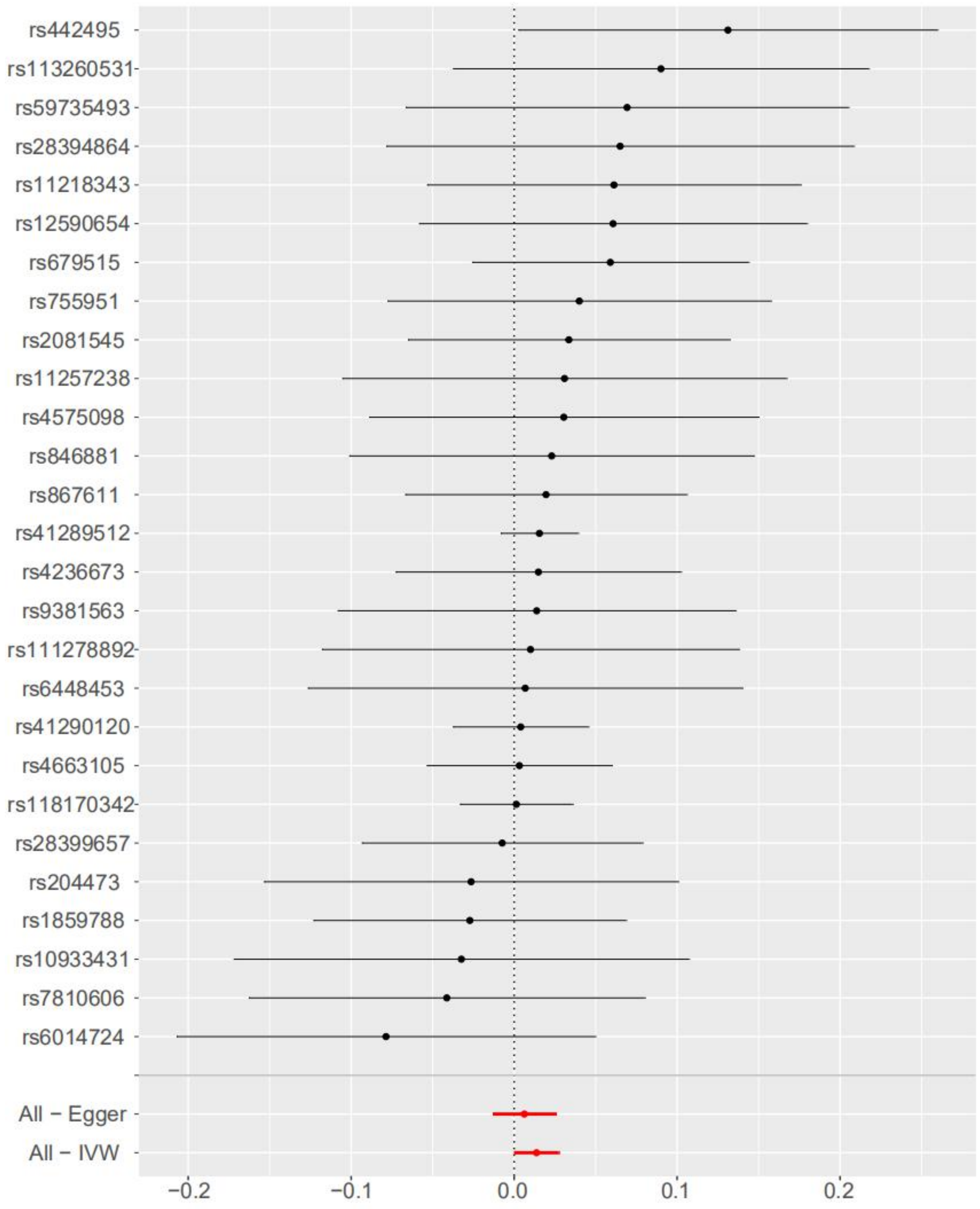


**Figure S32.** Single SNP analysis for individual and combined SNP effects of AD on thickness of cuneus.

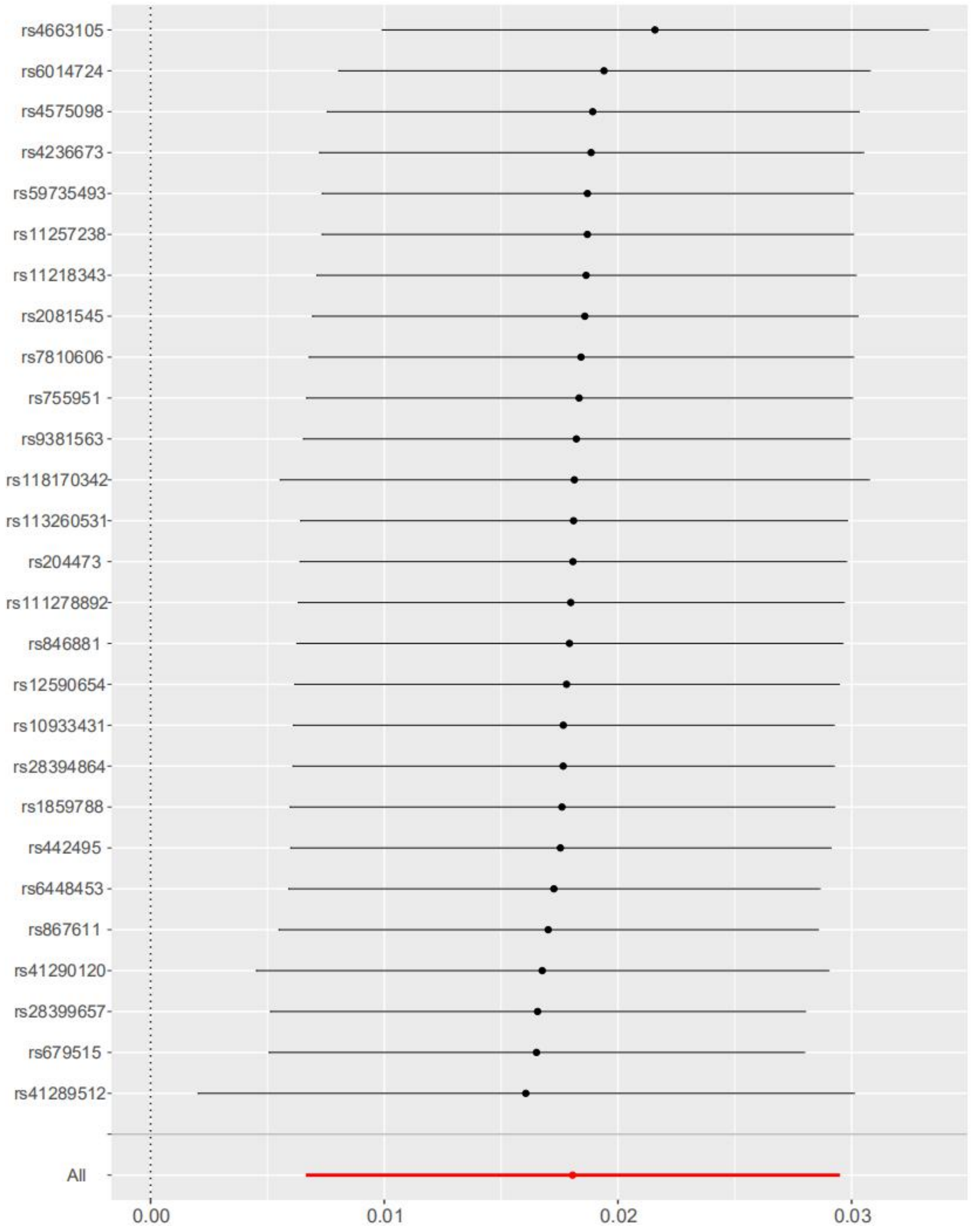


**Figure S33.** Leave-one-out analysis of association between genetically predicted AD and thickness of lateral occipital.

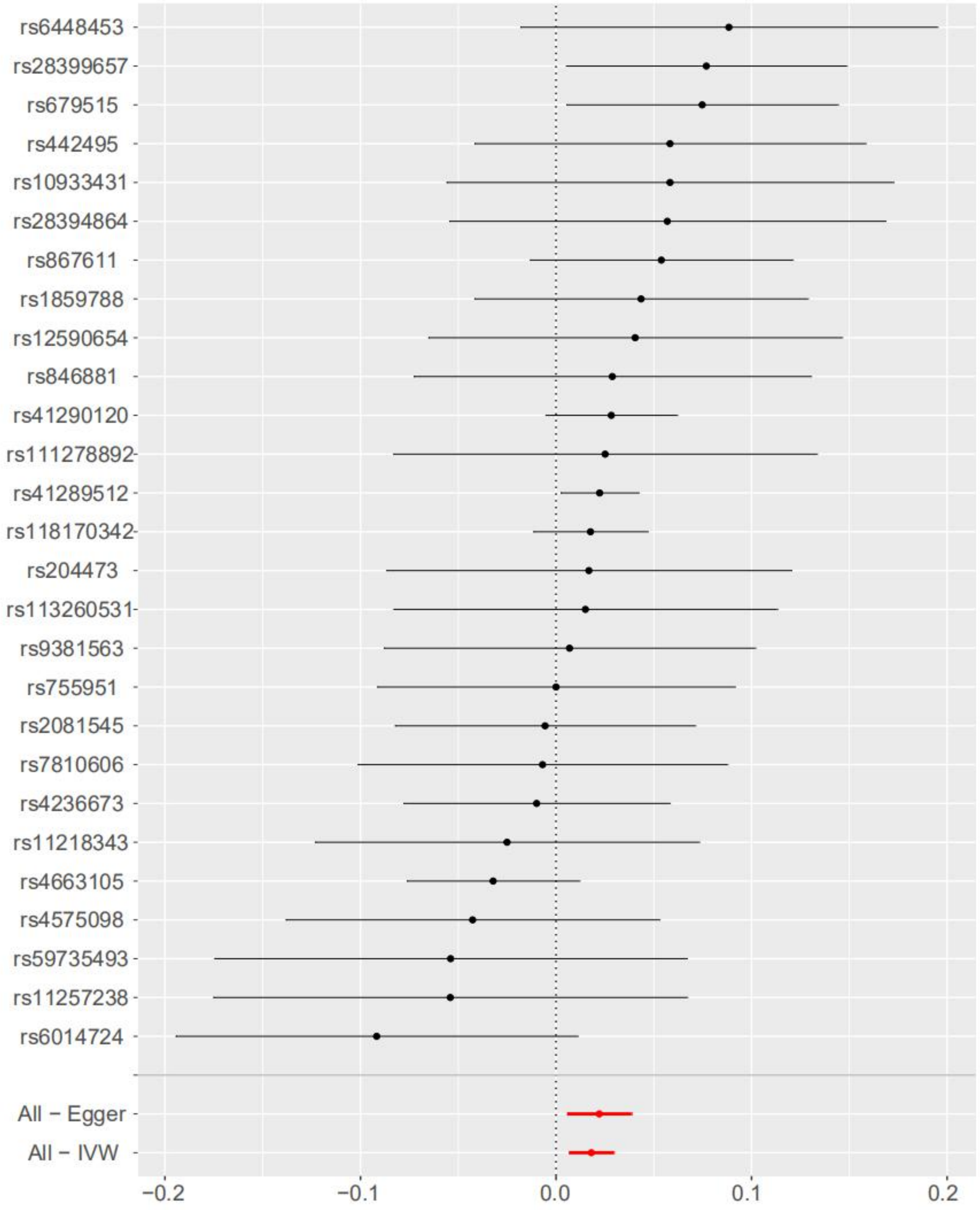


**Figure S34.** Single SNP analysis for individual and combined SNP effects of AD on thickness of lateral occipital.

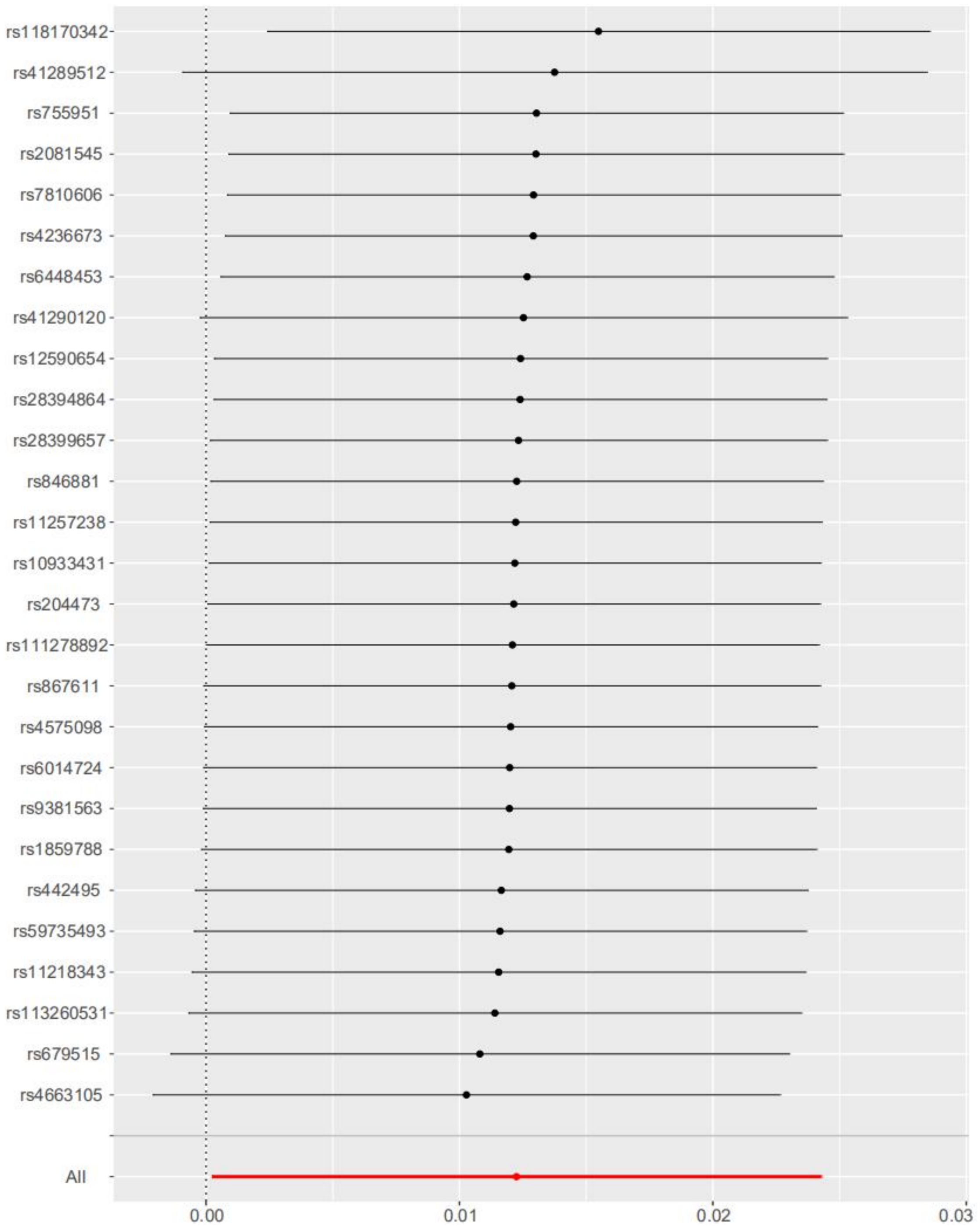


**Figure S35.** Leave-one-out analysis of association between genetically predicted AD and thickness of lingual.

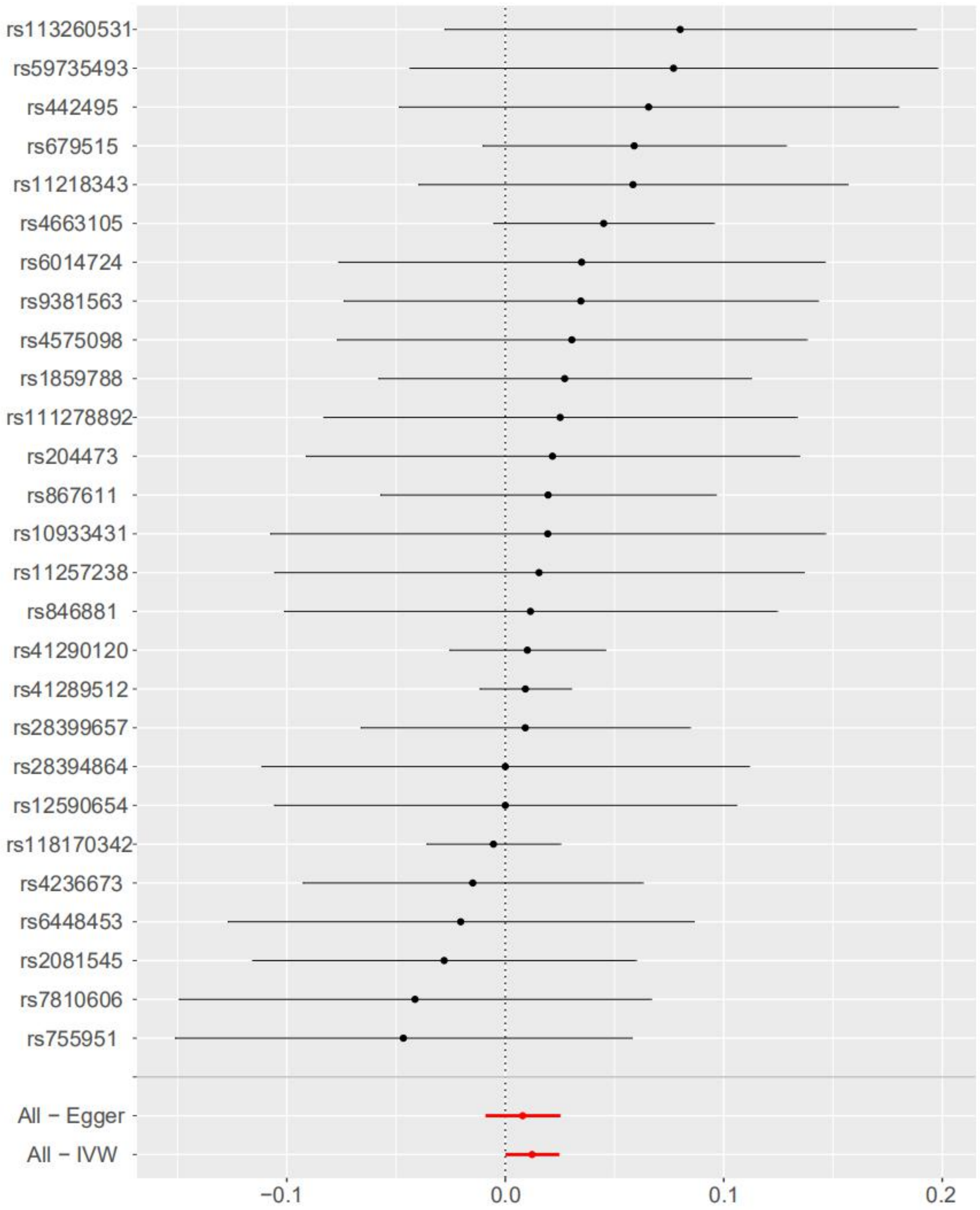


**Figure S36.** Single SNP analysis for individual and combined SNP effects of AD on thickness of lingual.
